# Supplementary figures and images for: JAK1/2 inhibitor ruxolitinib reduces aggregates in cardiac proteinopathy (part 1 of 3)
Source: EMBO Mol Med. 2026 Mar 31;18(5):1836–65. doi: 10.1038/s44321-026-00411-x (PMC13179346; doi:10.1038/s44321-026-00411-x)

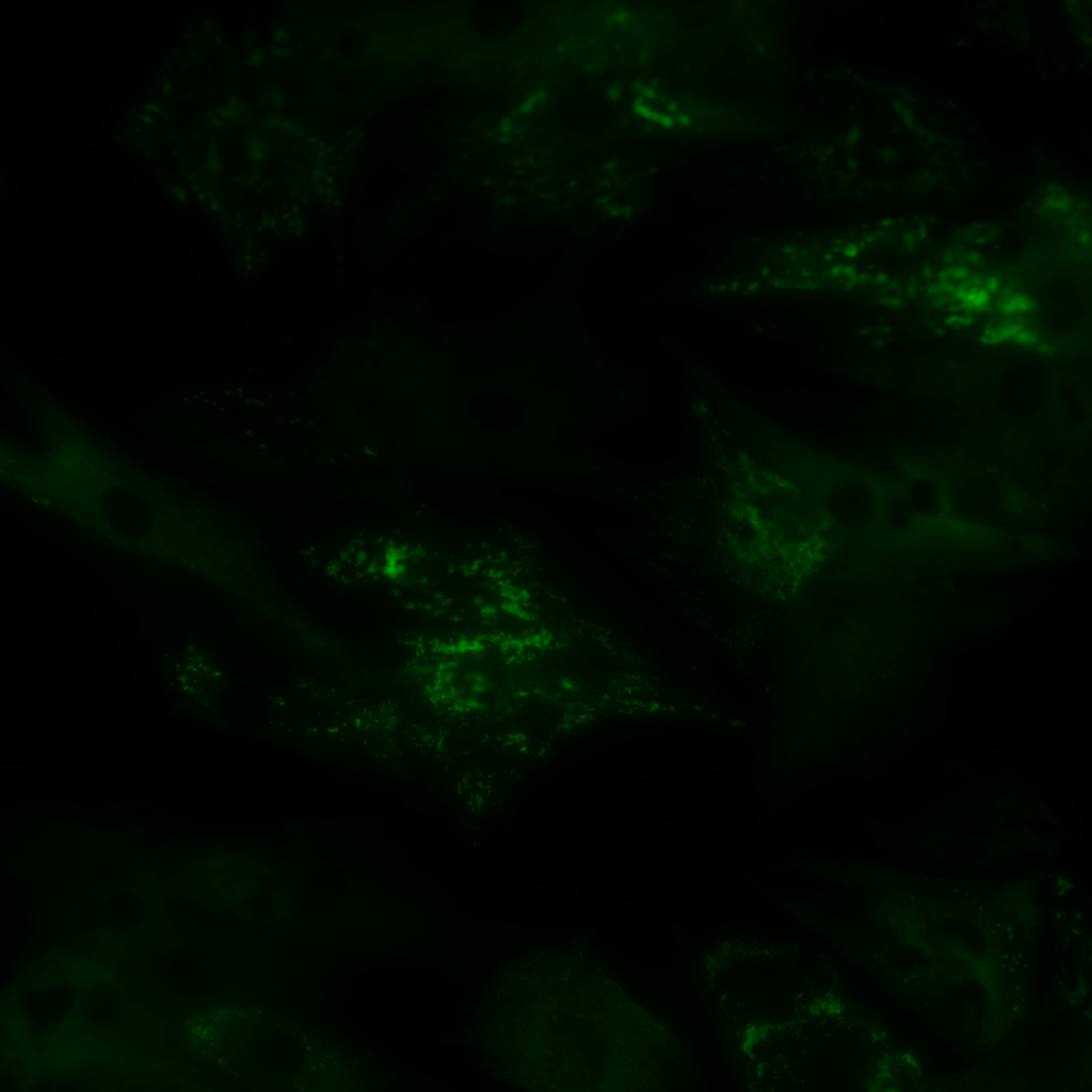

Supplement: Supplementary file 8 — Source data Fig. 1 [file 44321_2026_411_MOESM8_ESM.zip › Figure 1/1A/DMSO 12 h.tif]

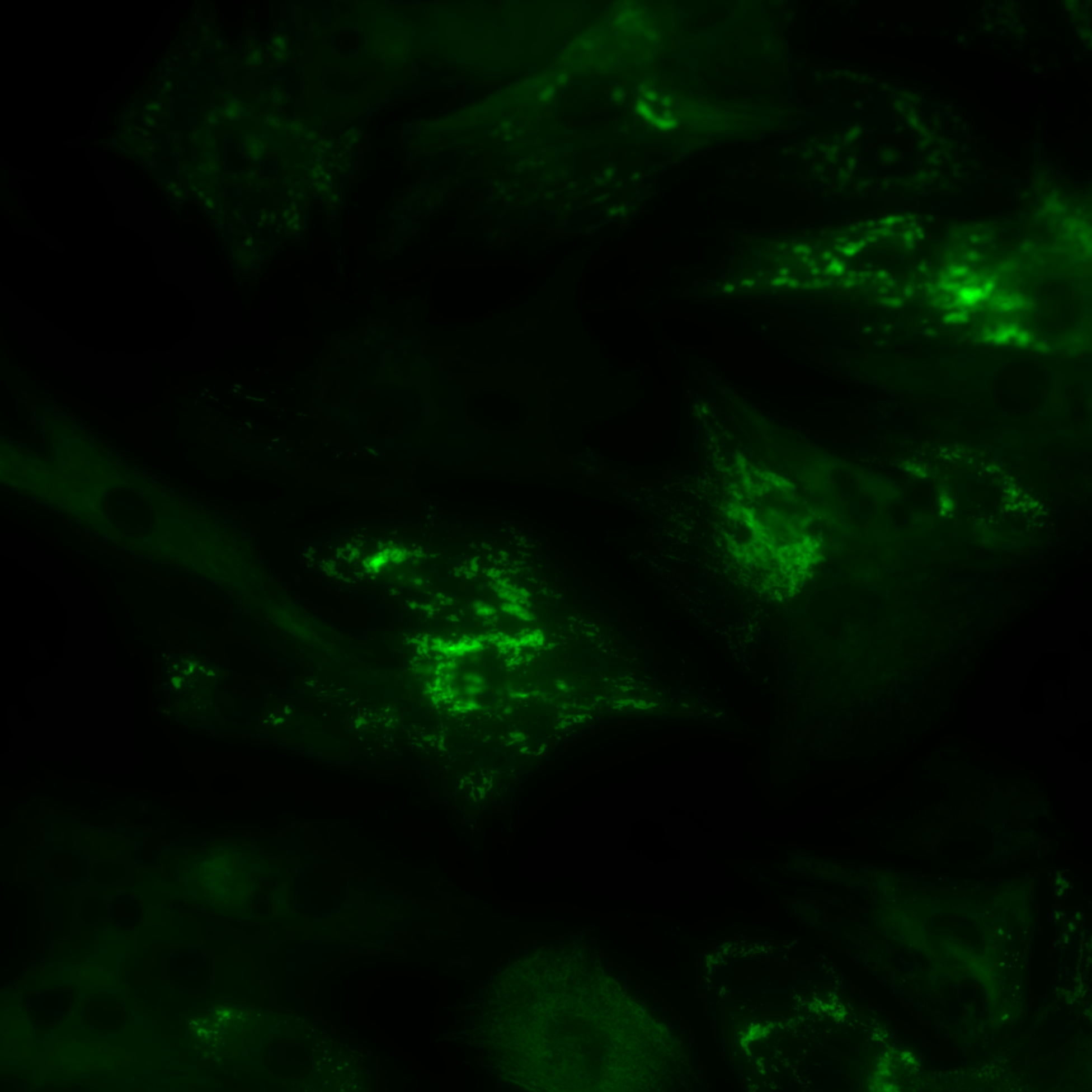

Supplement: Supplementary file 8 — Source data Fig. 1 [file 44321_2026_411_MOESM8_ESM.zip › Figure 1/1A/DMSO 18 h.tif]

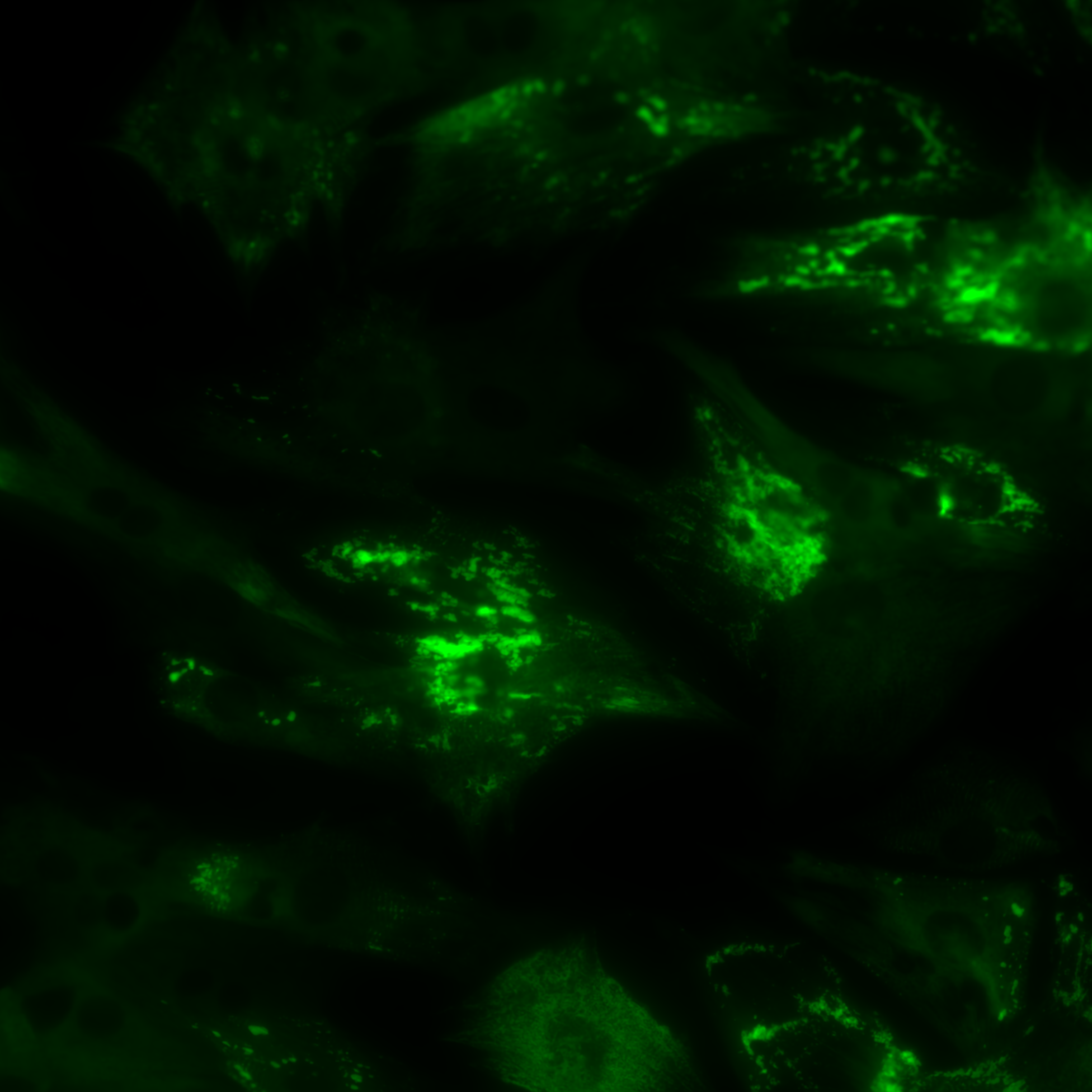

Supplement: Supplementary file 8 — Source data Fig. 1 [file 44321_2026_411_MOESM8_ESM.zip › Figure 1/1A/DMSO 24 h.tif]

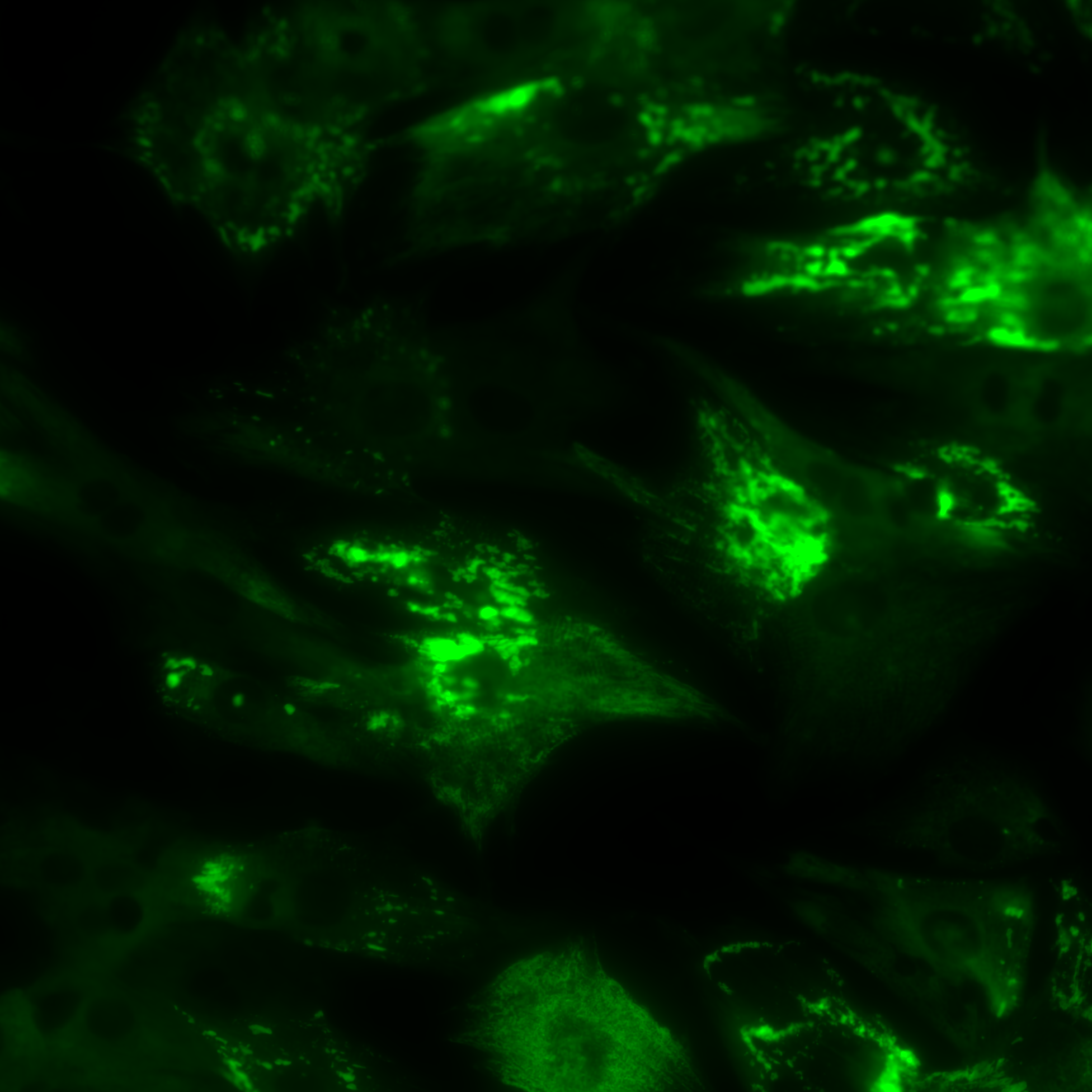

Supplement: Supplementary file 8 — Source data Fig. 1 [file 44321_2026_411_MOESM8_ESM.zip › Figure 1/1A/DMSO 30 h.tif]

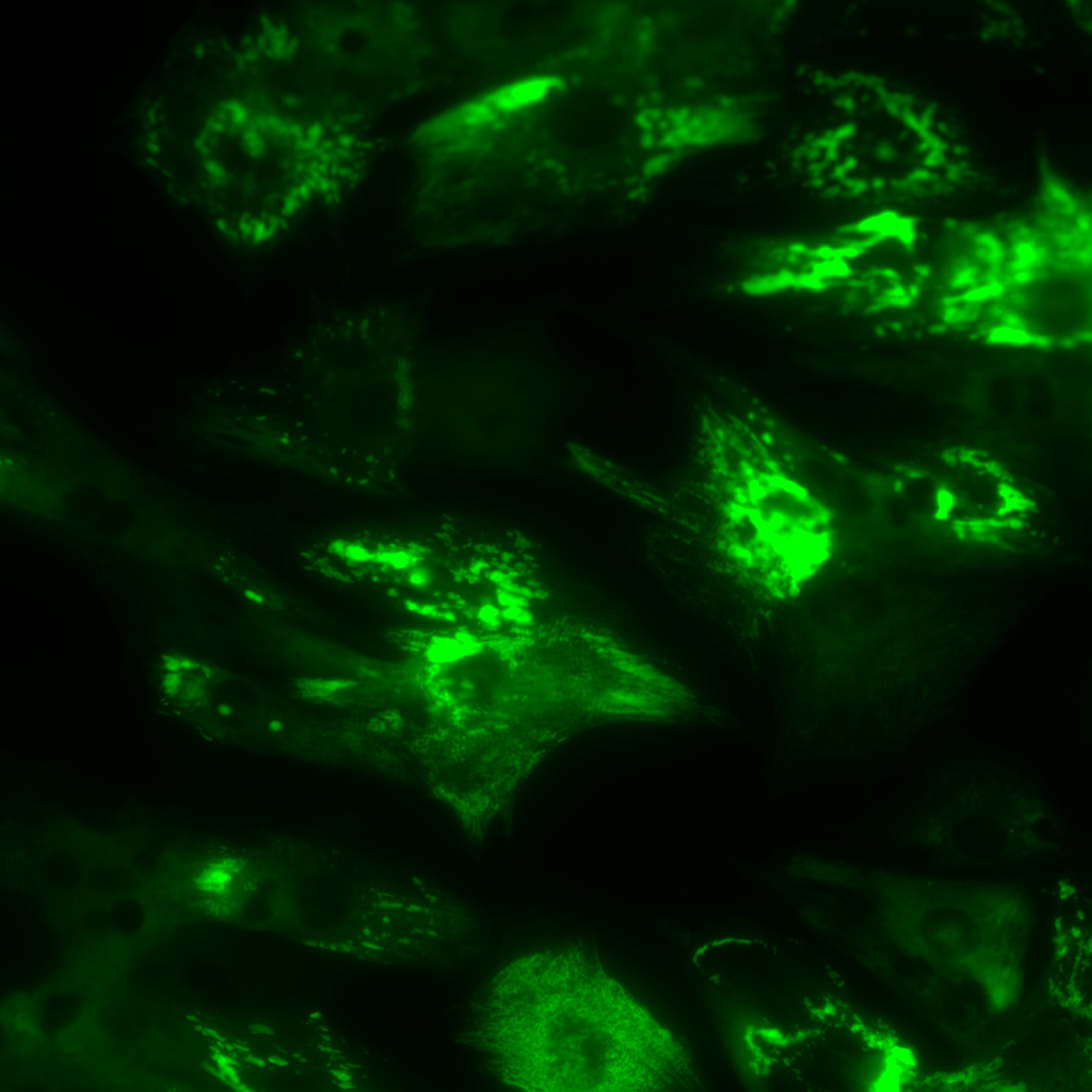

Supplement: Supplementary file 8 — Source data Fig. 1 [file 44321_2026_411_MOESM8_ESM.zip › Figure 1/1A/DMSO 36 h.tif]

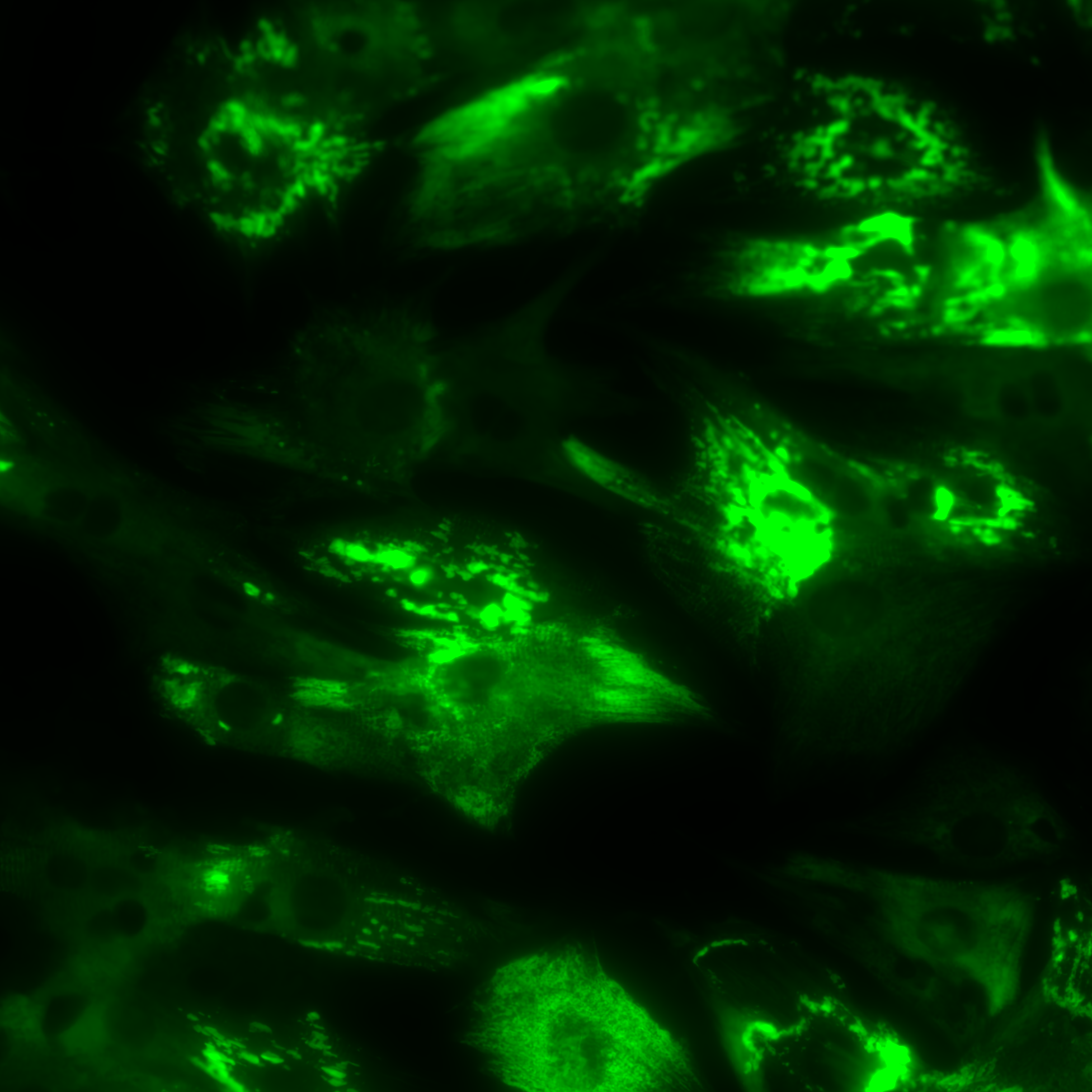

Supplement: Supplementary file 8 — Source data Fig. 1 [file 44321_2026_411_MOESM8_ESM.zip › Figure 1/1A/DMSO 42 h.tif]

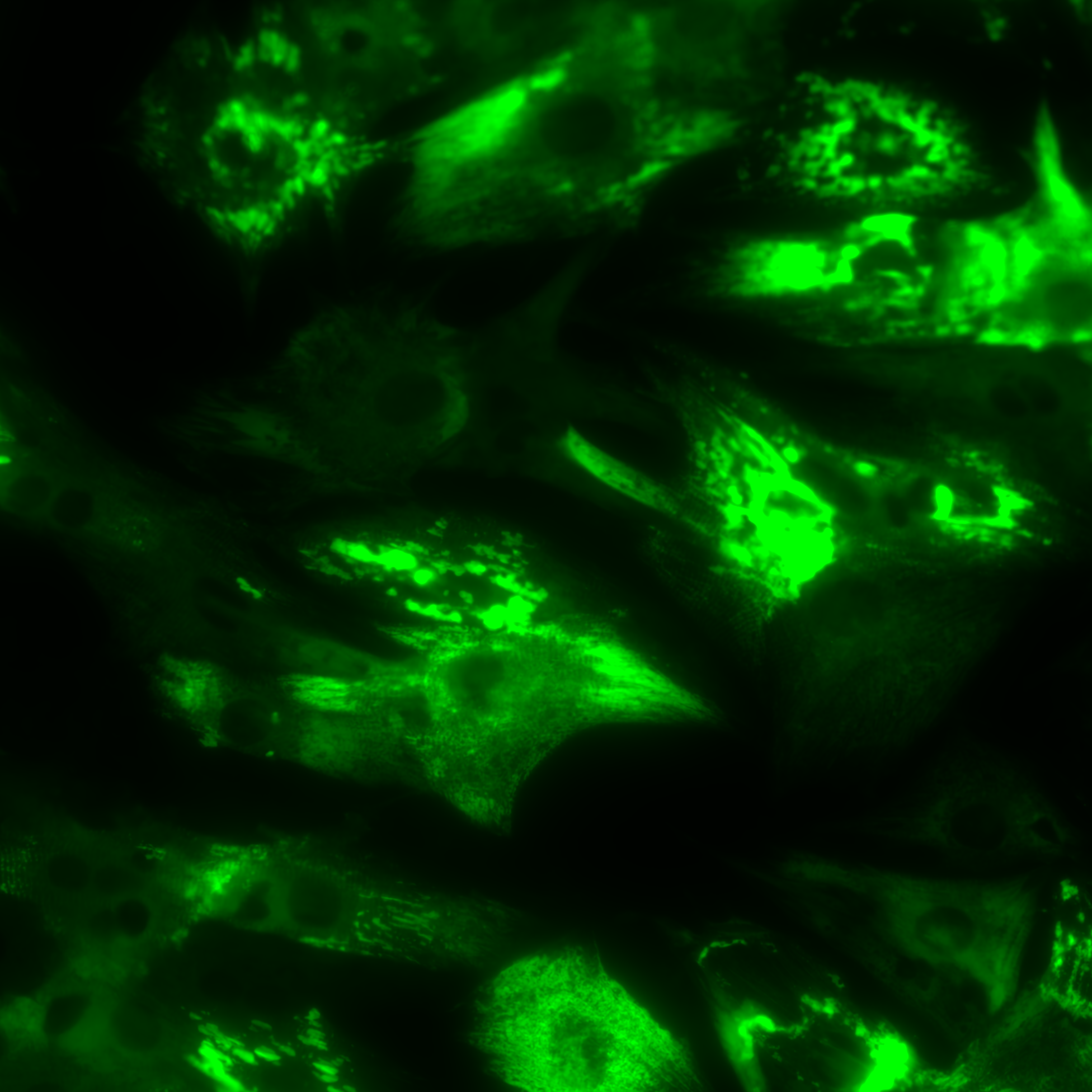

Supplement: Supplementary file 8 — Source data Fig. 1 [file 44321_2026_411_MOESM8_ESM.zip › Figure 1/1A/DMSO 48 h.tif]

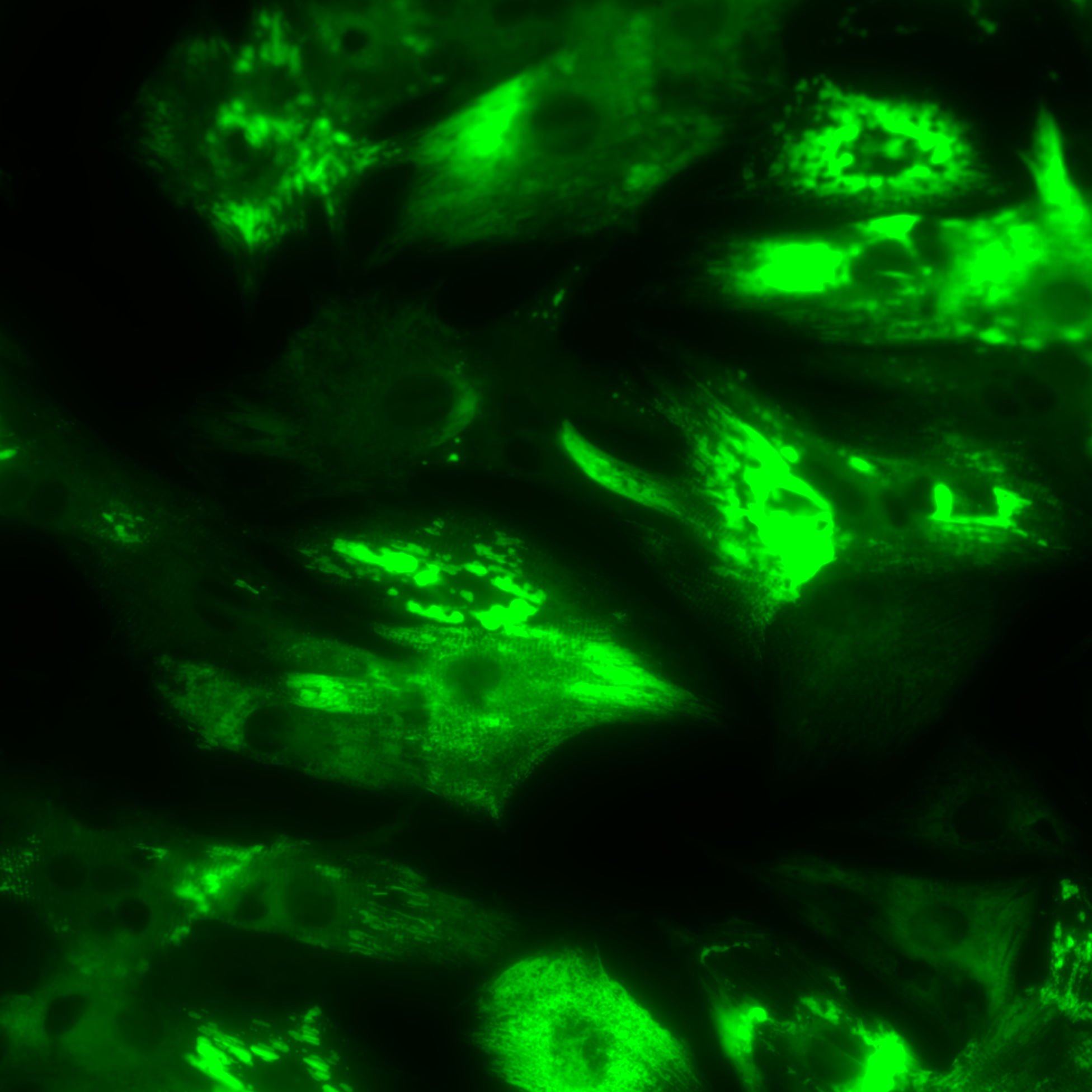

Supplement: Supplementary file 8 — Source data Fig. 1 [file 44321_2026_411_MOESM8_ESM.zip › Figure 1/1A/DMSO 54 h.tif]

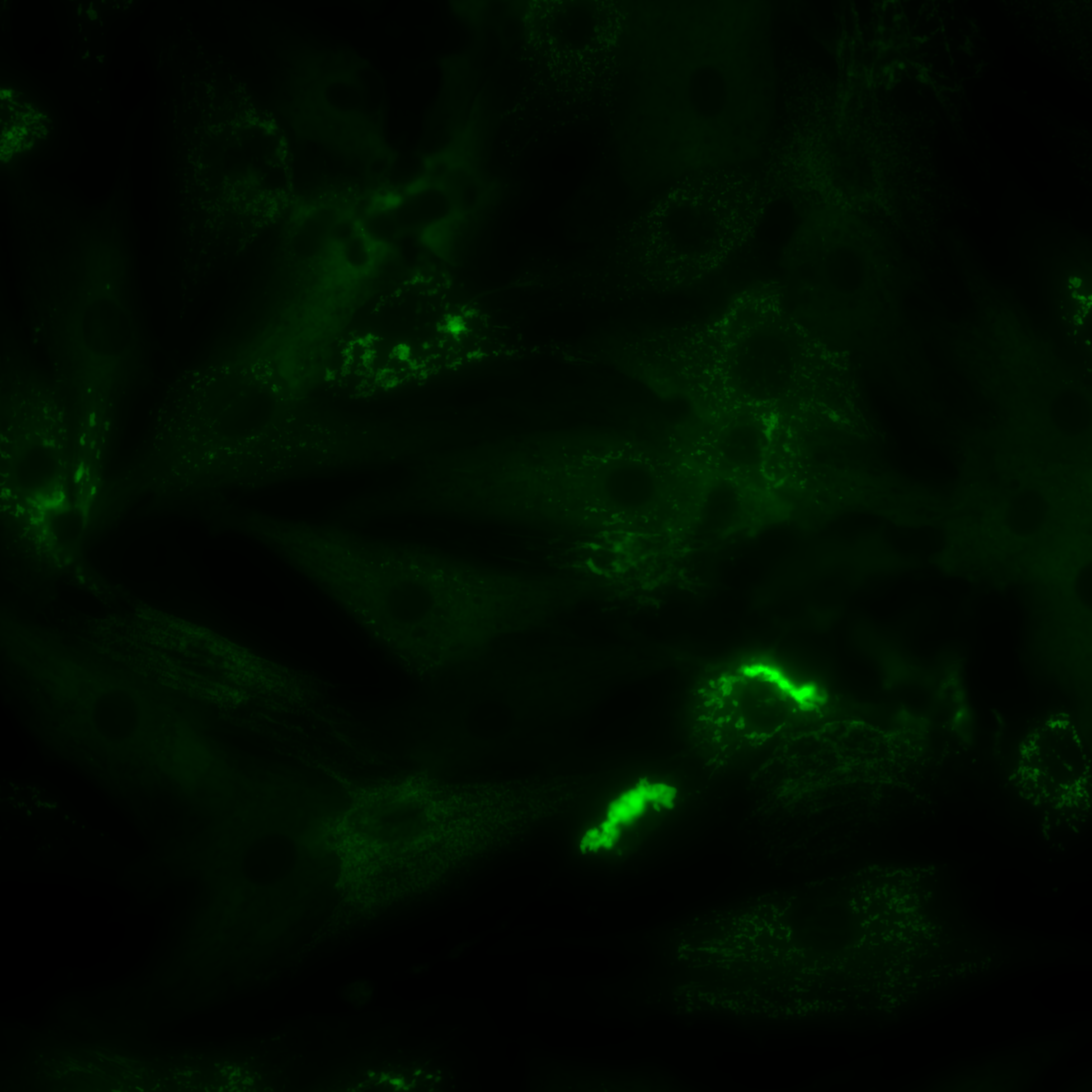

Supplement: Supplementary file 8 — Source data Fig. 1 [file 44321_2026_411_MOESM8_ESM.zip › Figure 1/1A/Ruxo 12 h.tif]

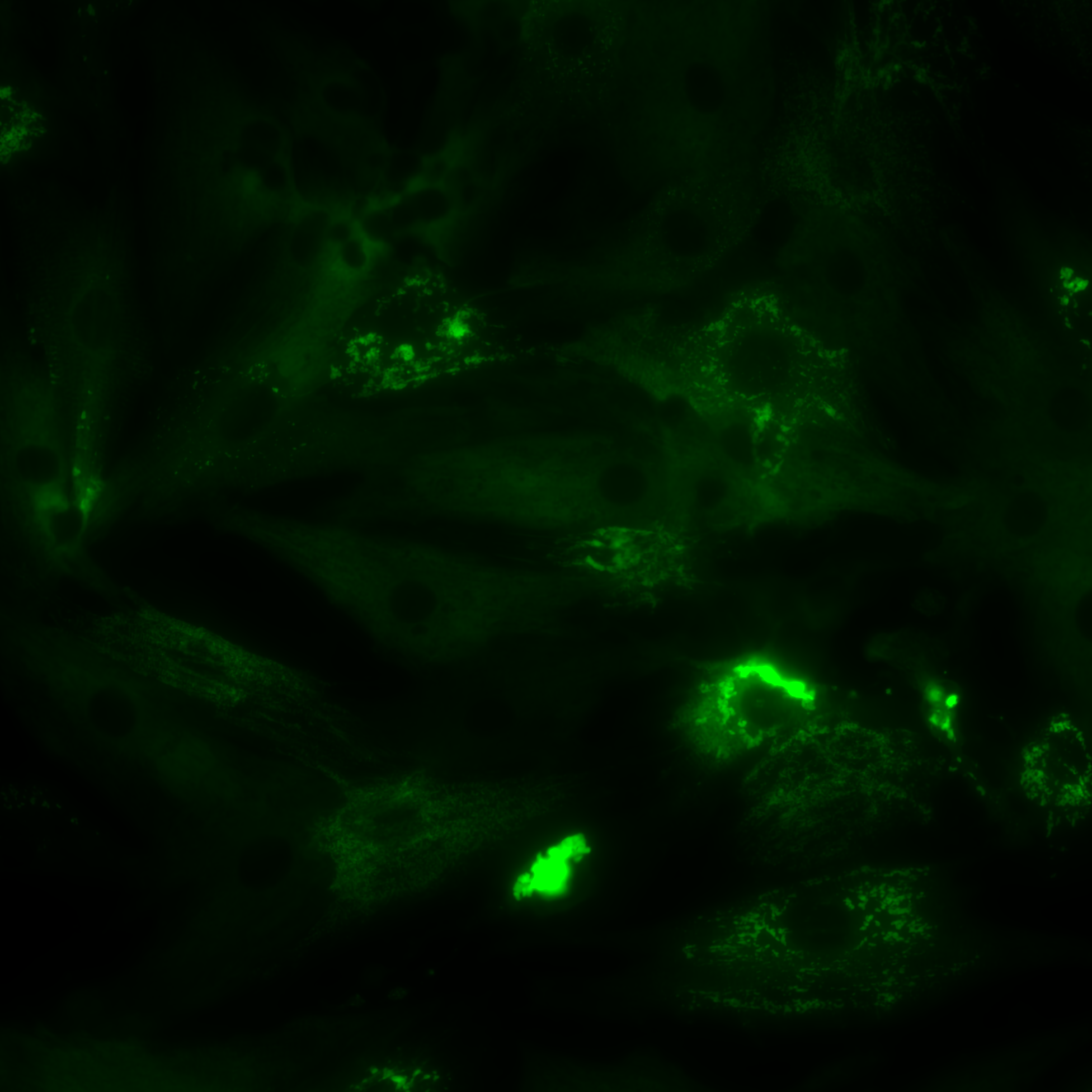

Supplement: Supplementary file 8 — Source data Fig. 1 [file 44321_2026_411_MOESM8_ESM.zip › Figure 1/1A/Ruxo 18 h.tif]

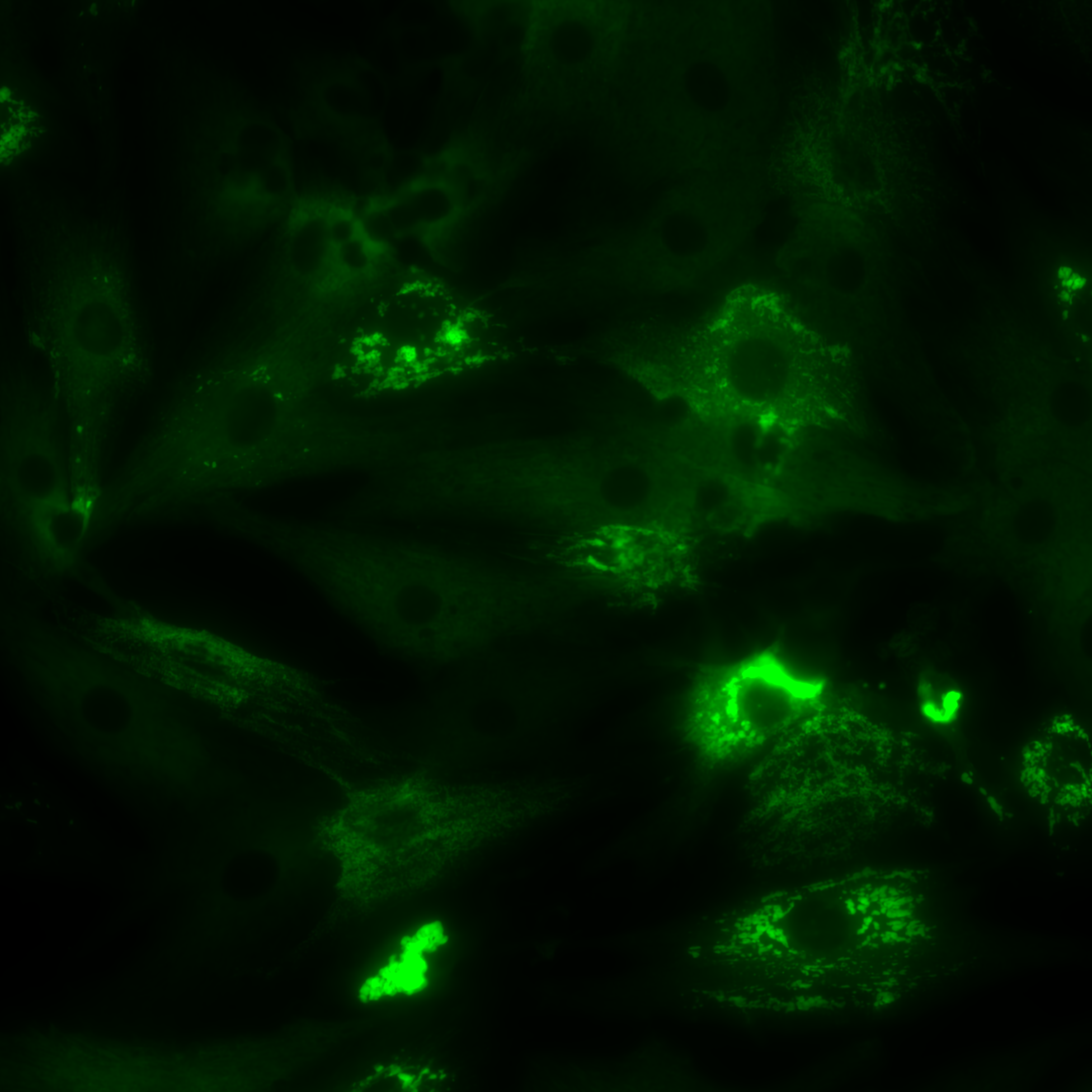

Supplement: Supplementary file 8 — Source data Fig. 1 [file 44321_2026_411_MOESM8_ESM.zip › Figure 1/1A/Ruxo 24 h.tif]

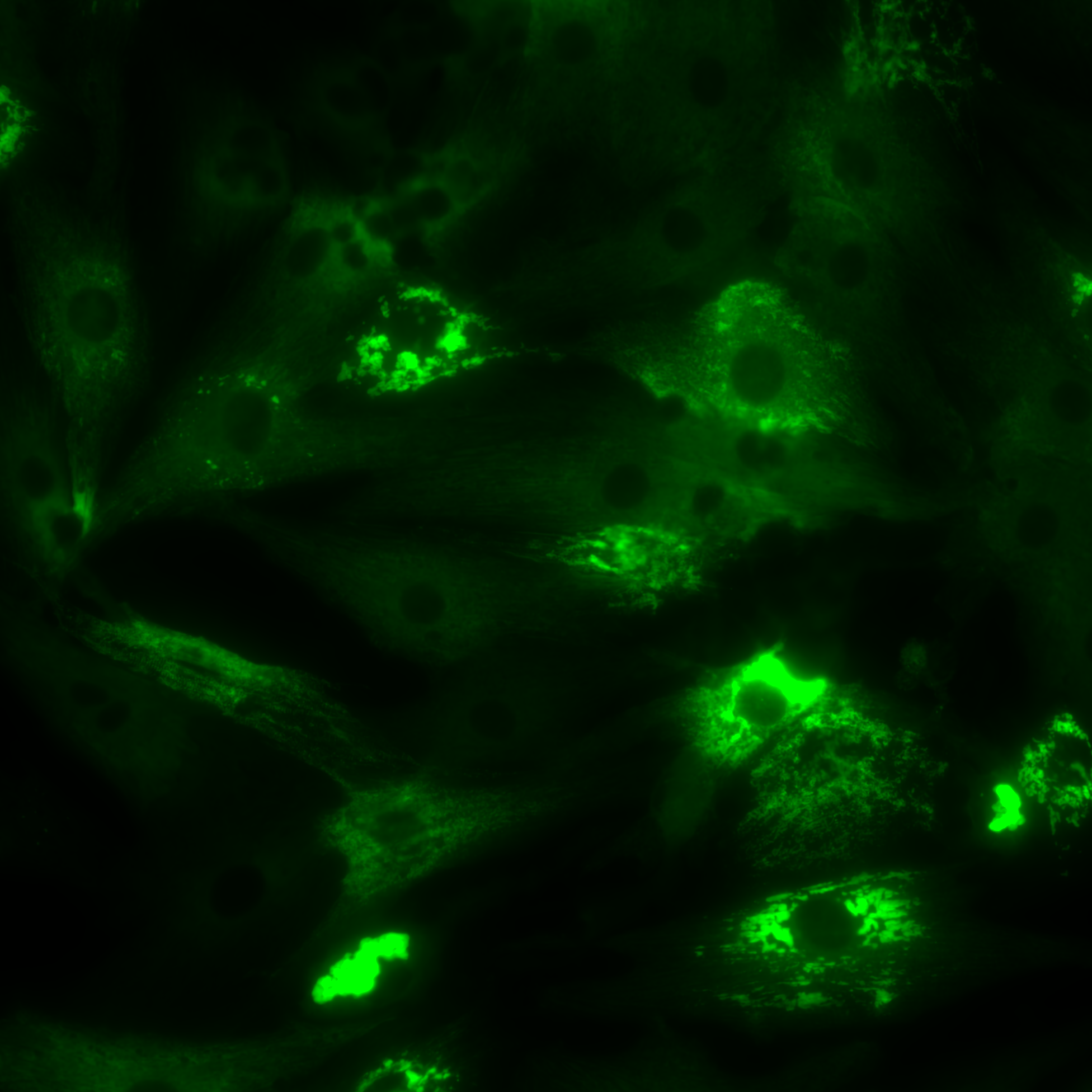

Supplement: Supplementary file 8 — Source data Fig. 1 [file 44321_2026_411_MOESM8_ESM.zip › Figure 1/1A/Ruxo 30 h.tif]

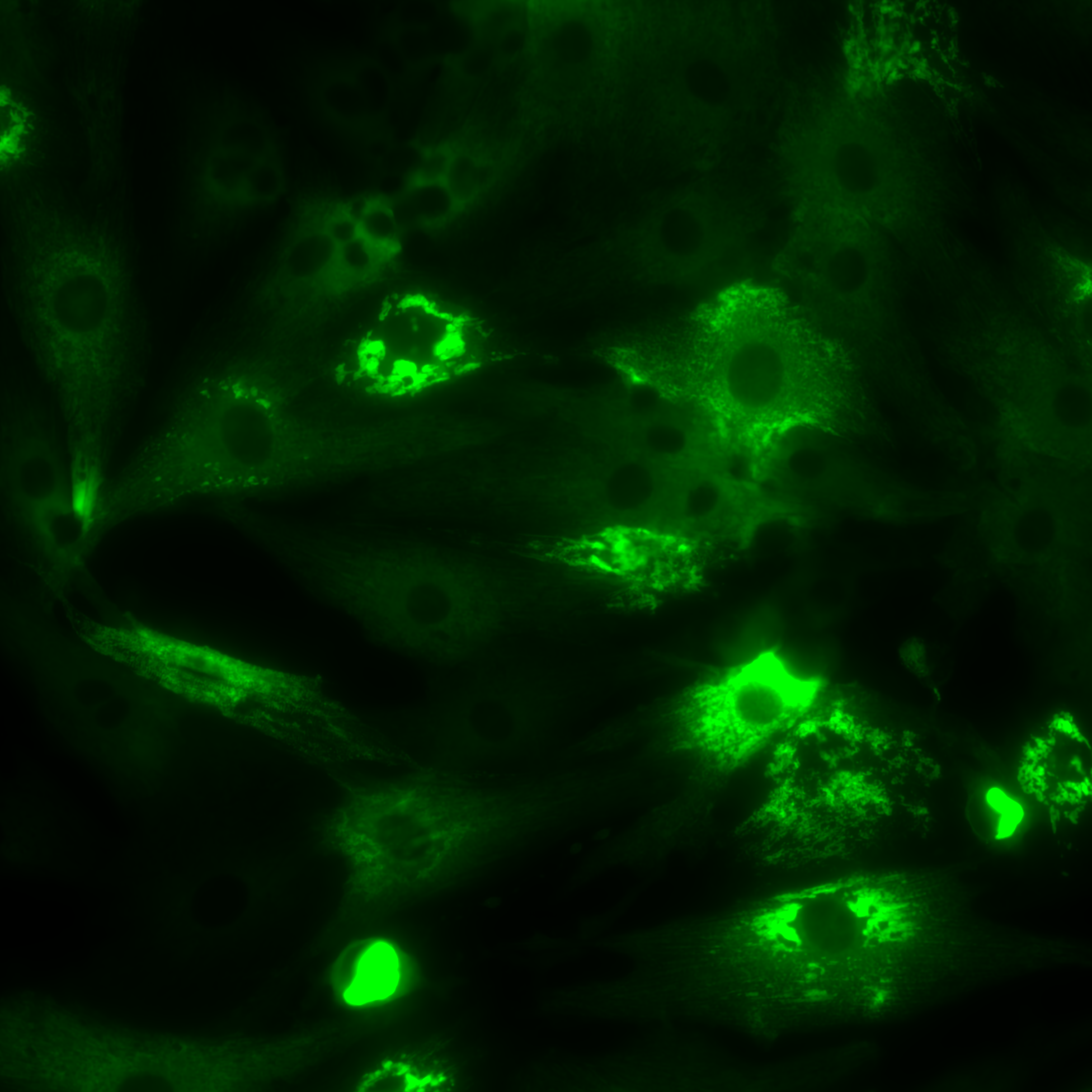

Supplement: Supplementary file 8 — Source data Fig. 1 [file 44321_2026_411_MOESM8_ESM.zip › Figure 1/1A/Ruxo 36 h.tif]

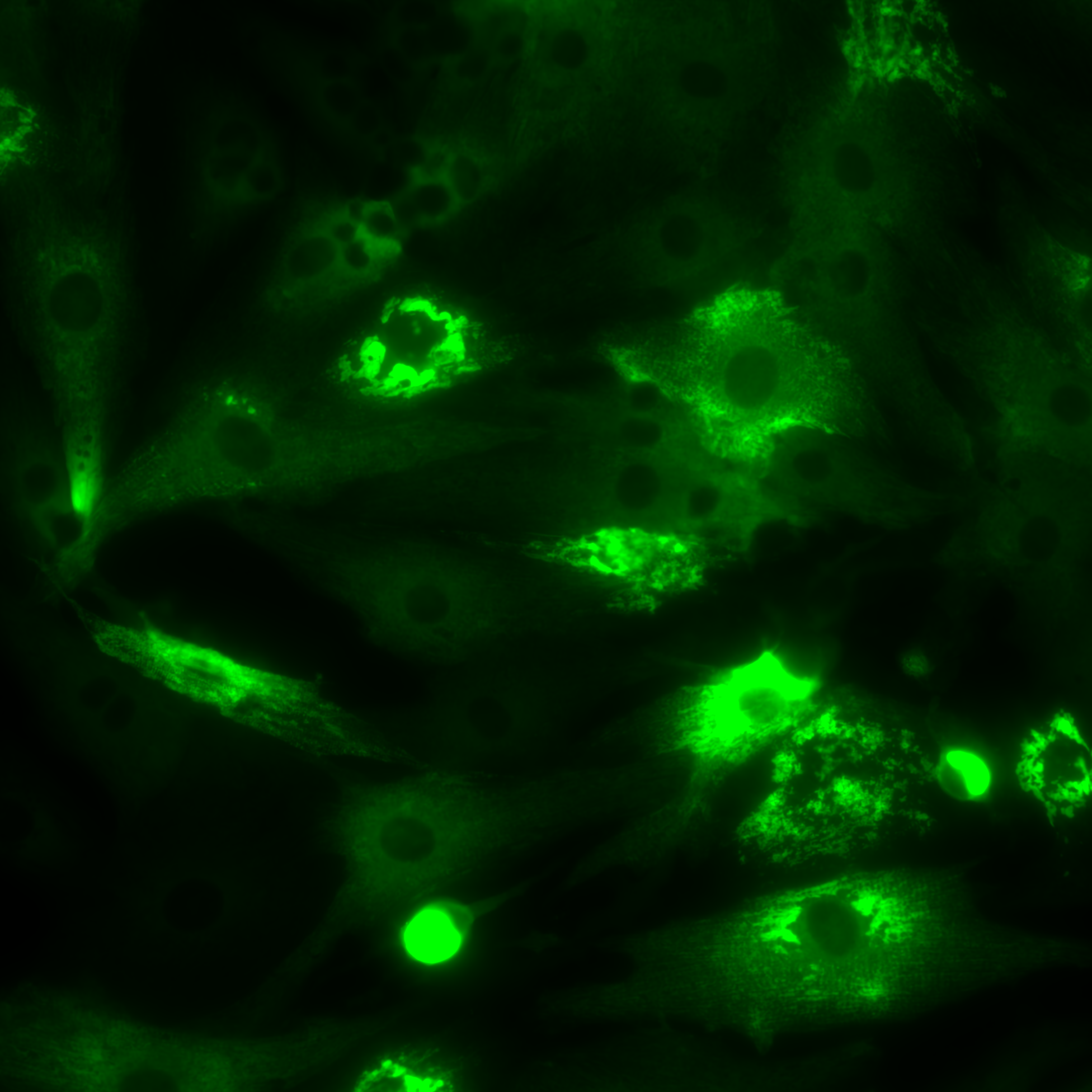

Supplement: Supplementary file 8 — Source data Fig. 1 [file 44321_2026_411_MOESM8_ESM.zip › Figure 1/1A/Ruxo 42 h.tif]

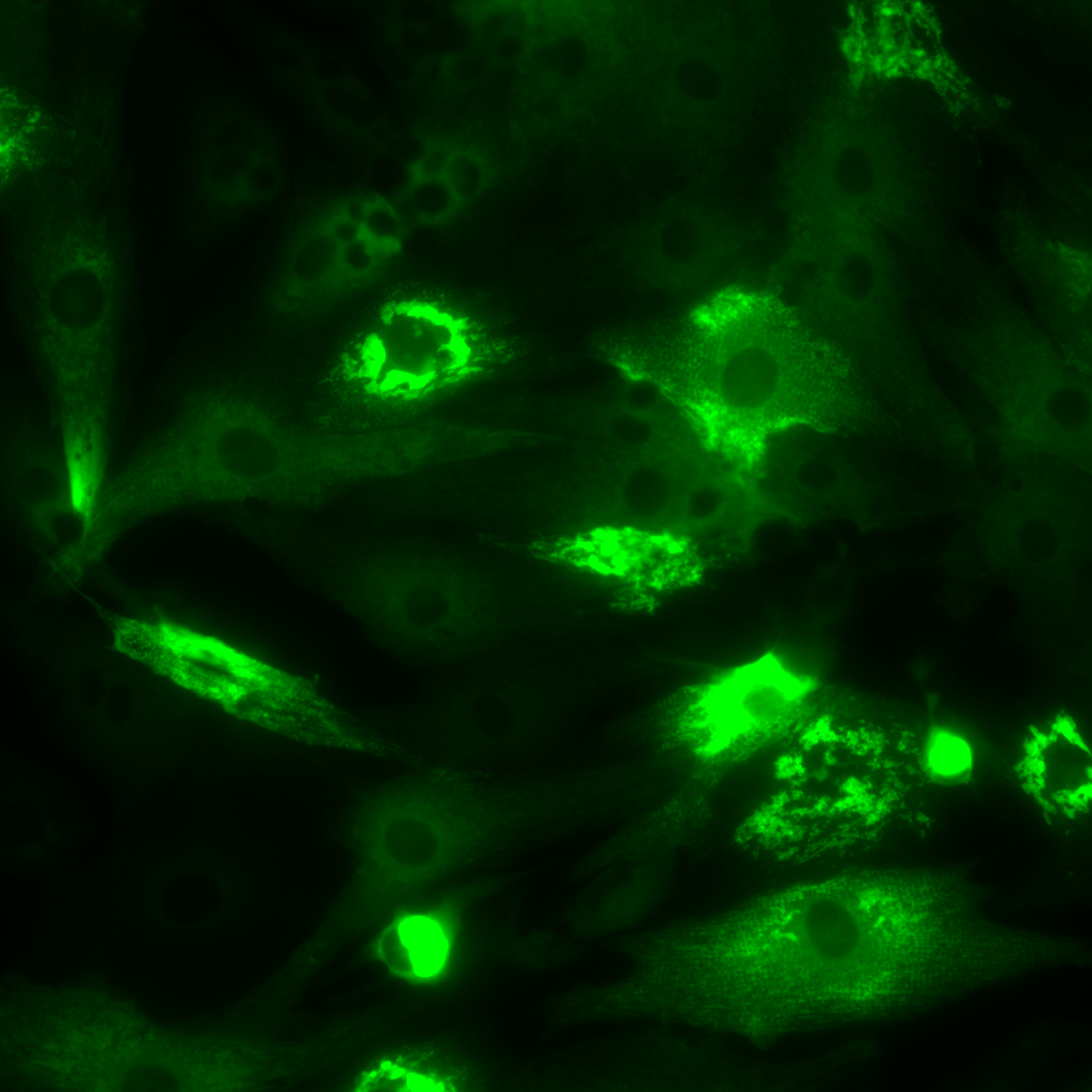

Supplement: Supplementary file 8 — Source data Fig. 1 [file 44321_2026_411_MOESM8_ESM.zip › Figure 1/1A/Ruxo 48 h.tif]

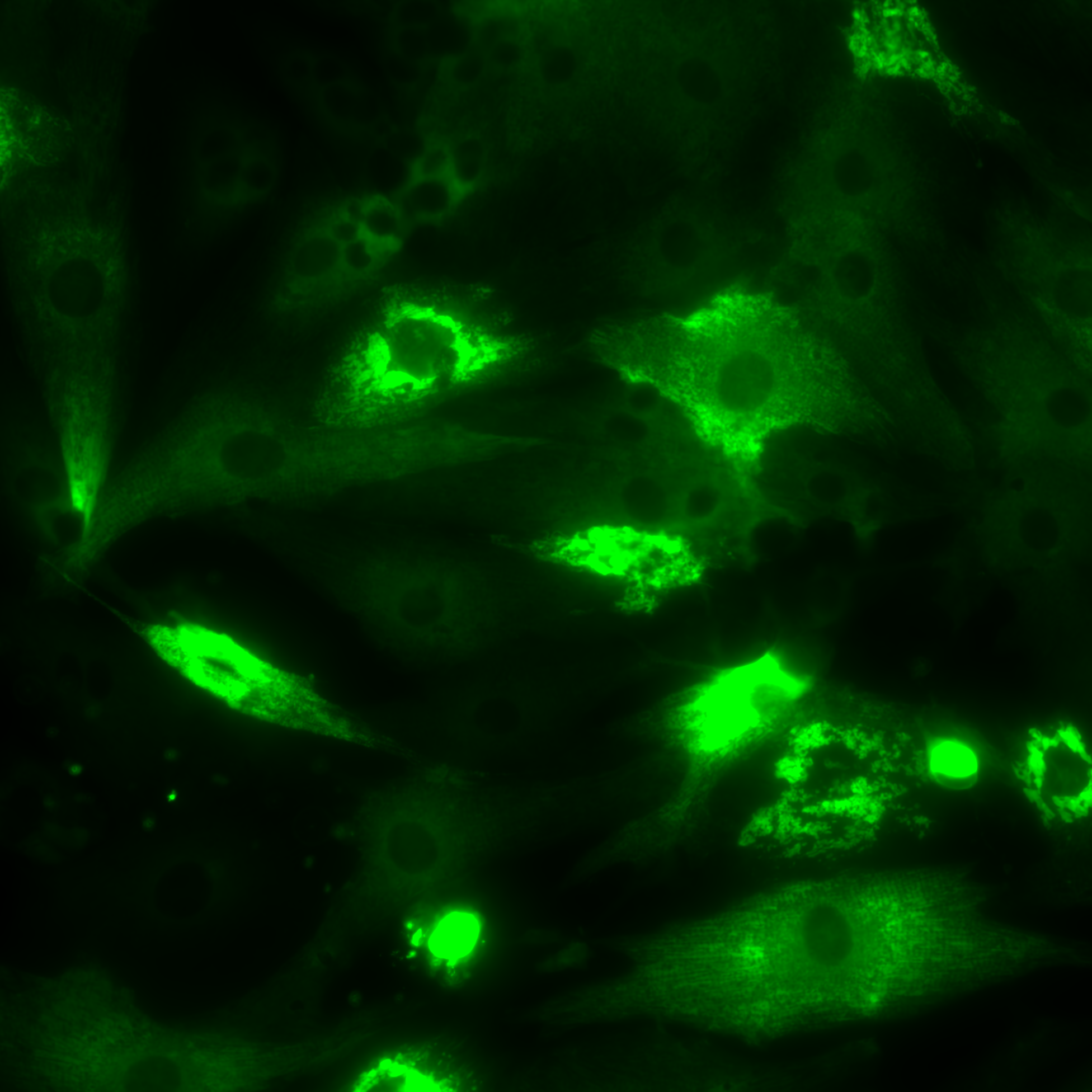

Supplement: Supplementary file 8 — Source data Fig. 1 [file 44321_2026_411_MOESM8_ESM.zip › Figure 1/1A/Ruxo 54 h.tif]

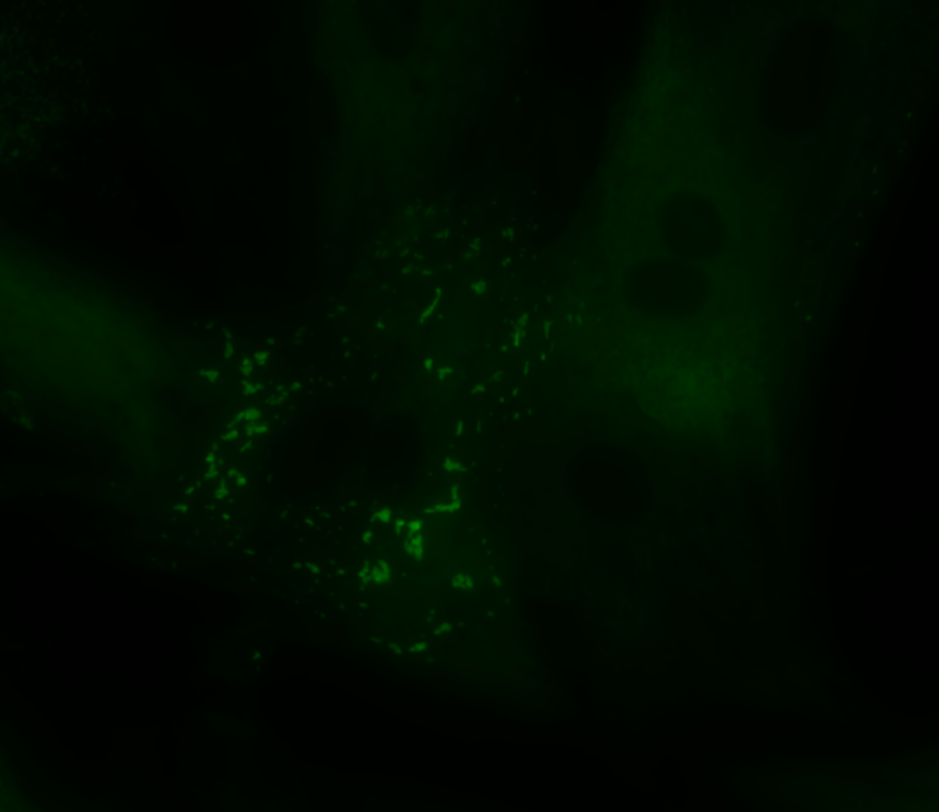

Supplement: Supplementary file 8 — Source data Fig. 1 [file 44321_2026_411_MOESM8_ESM.zip › Figure 1/1B/DMSO 12 h.tif]

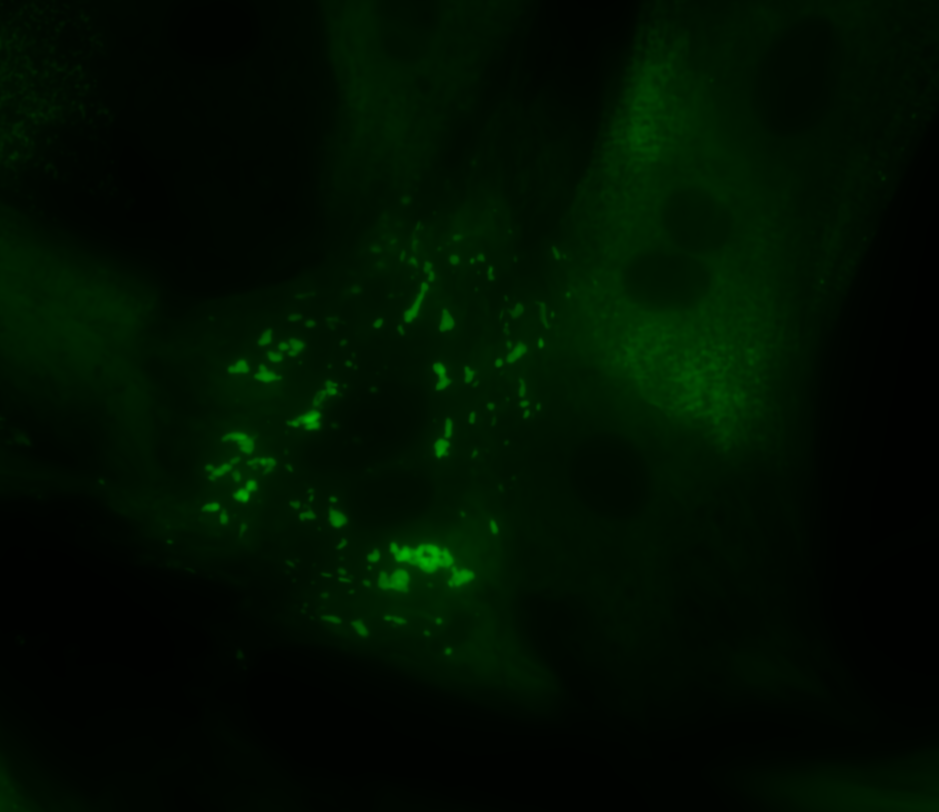

Supplement: Supplementary file 8 — Source data Fig. 1 [file 44321_2026_411_MOESM8_ESM.zip › Figure 1/1B/DMSO 18 h.tif]

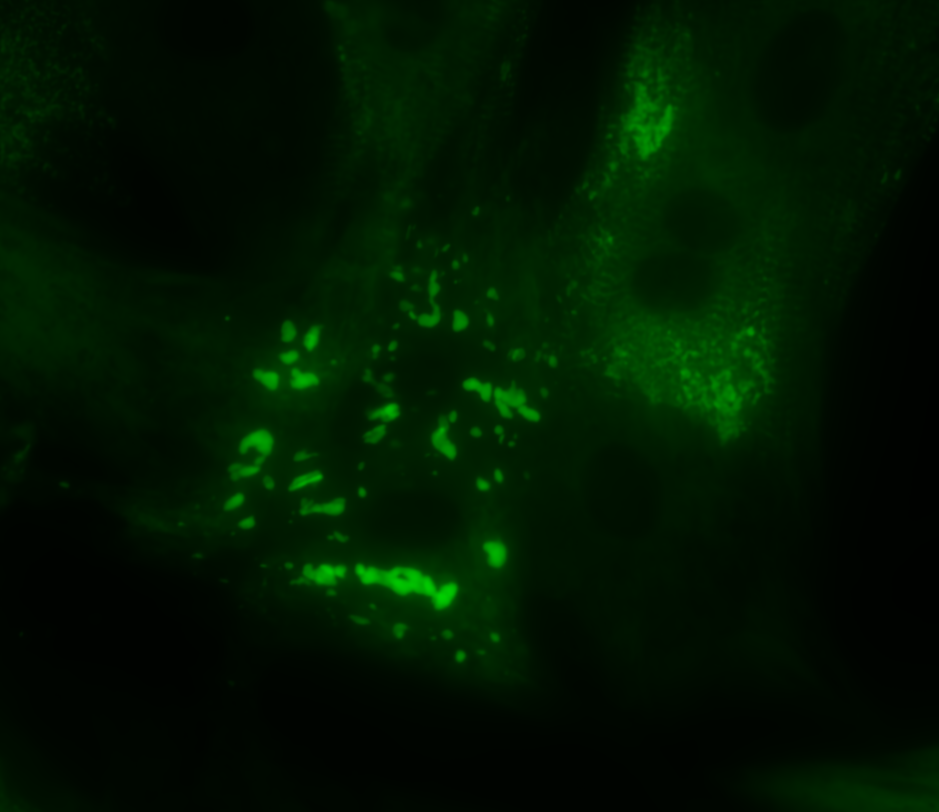

Supplement: Supplementary file 8 — Source data Fig. 1 [file 44321_2026_411_MOESM8_ESM.zip › Figure 1/1B/DMSO 24 h.tif]

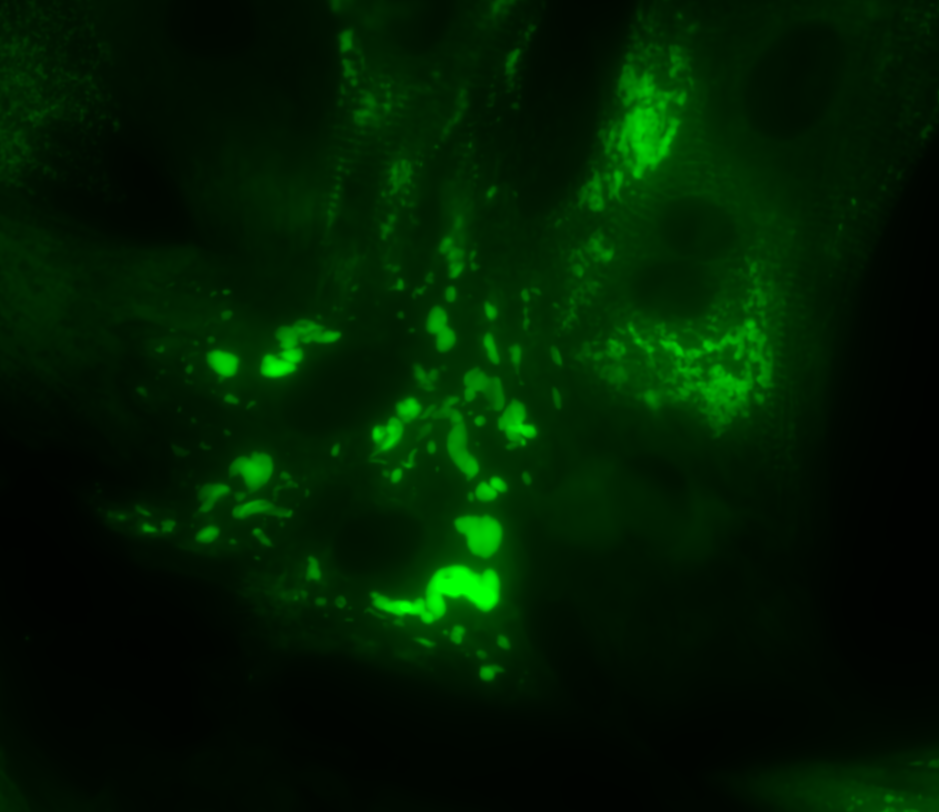

Supplement: Supplementary file 8 — Source data Fig. 1 [file 44321_2026_411_MOESM8_ESM.zip › Figure 1/1B/DMSO 30 h.tif]

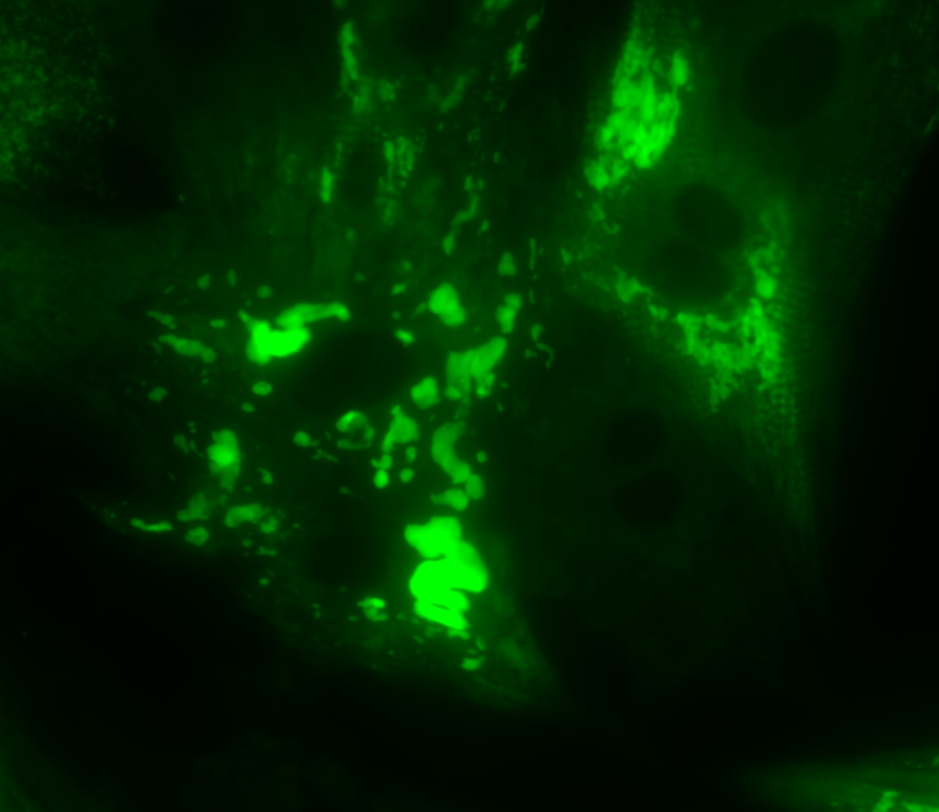

Supplement: Supplementary file 8 — Source data Fig. 1 [file 44321_2026_411_MOESM8_ESM.zip › Figure 1/1B/DMSO 36 h.tif]

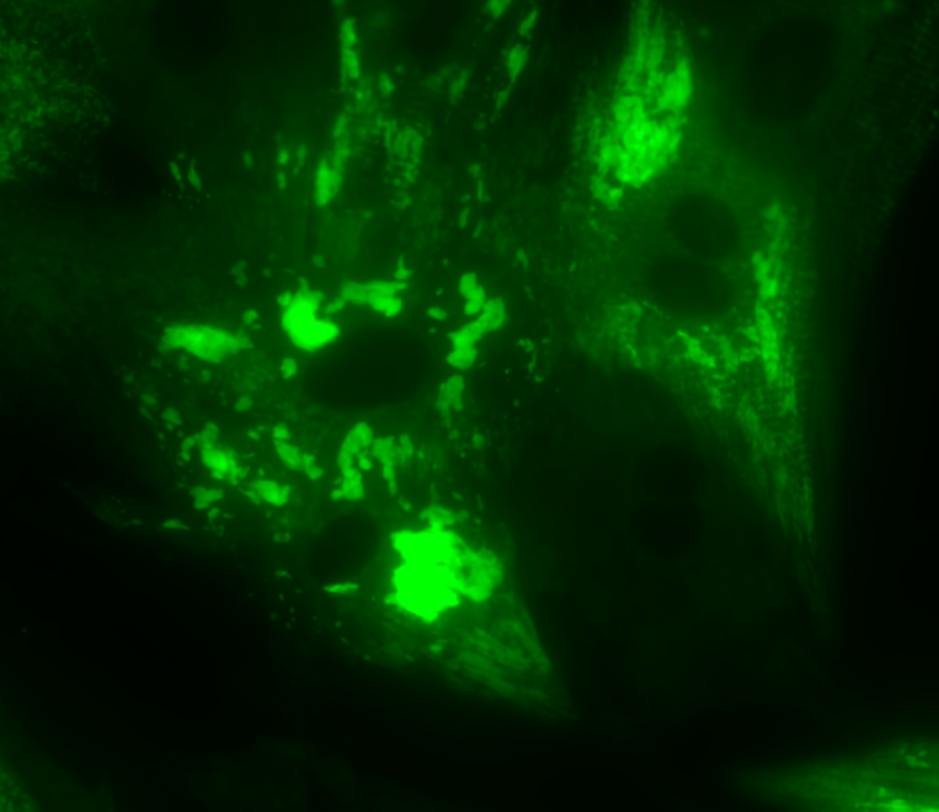

Supplement: Supplementary file 8 — Source data Fig. 1 [file 44321_2026_411_MOESM8_ESM.zip › Figure 1/1B/DMSO 42 h.tif]

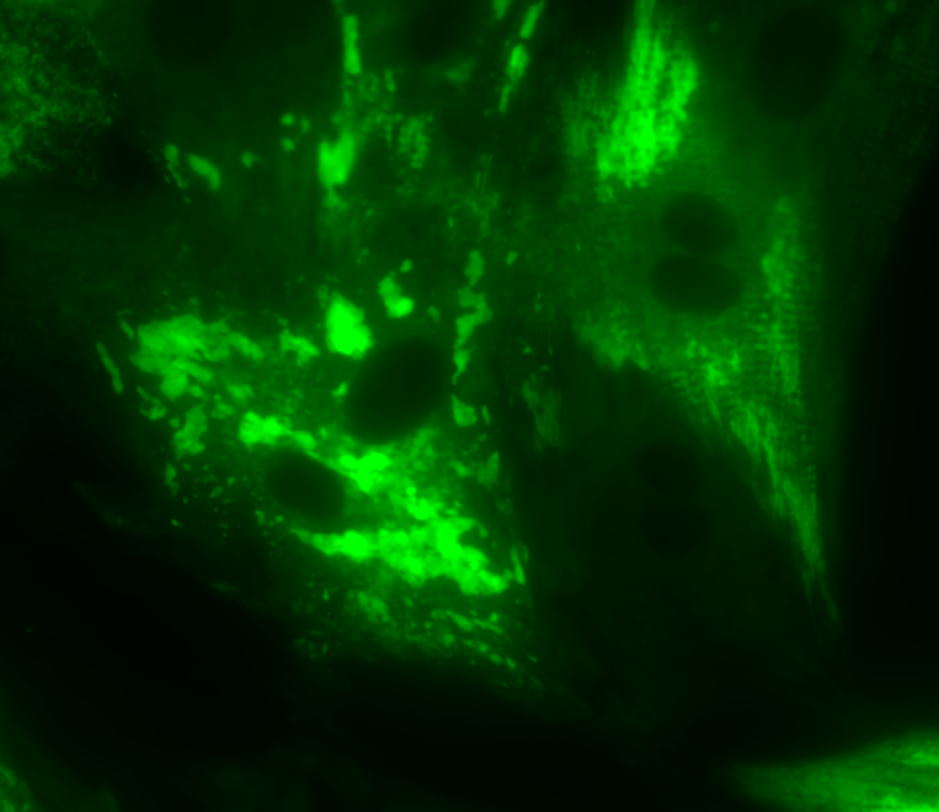

Supplement: Supplementary file 8 — Source data Fig. 1 [file 44321_2026_411_MOESM8_ESM.zip › Figure 1/1B/DMSO 48 h.tif]

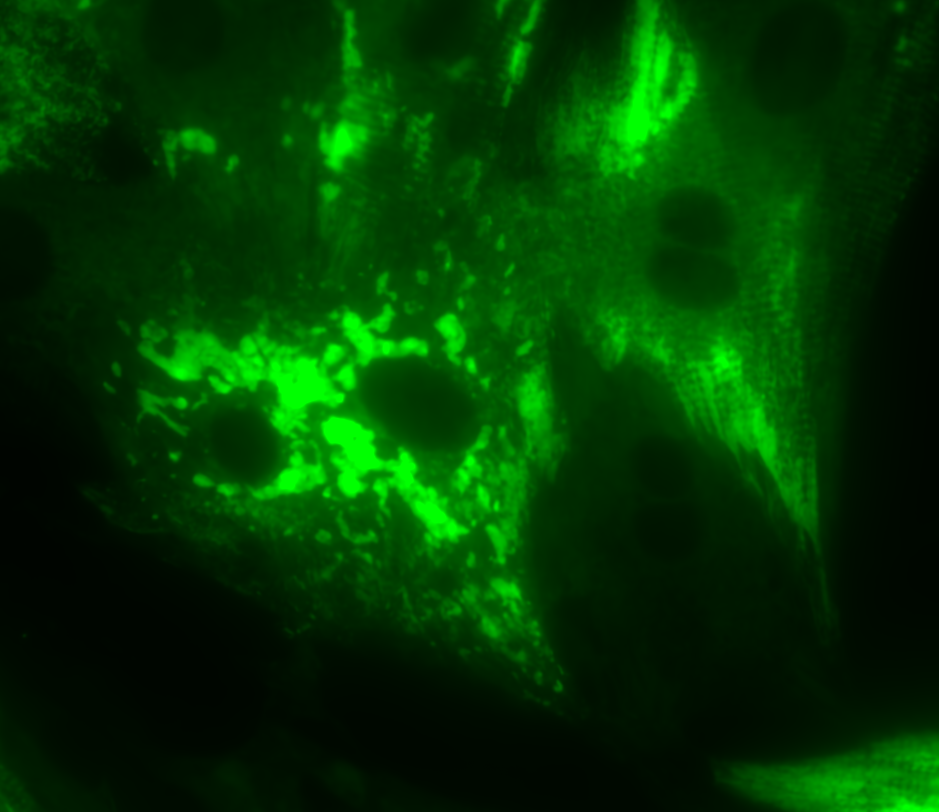

Supplement: Supplementary file 8 — Source data Fig. 1 [file 44321_2026_411_MOESM8_ESM.zip › Figure 1/1B/DMSO 54 h.tif]

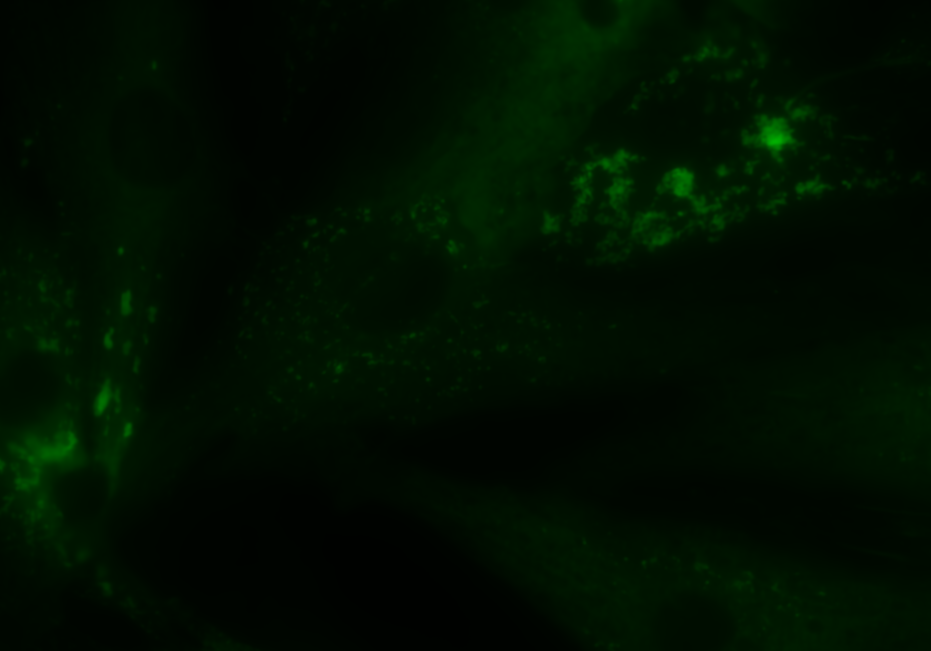

Supplement: Supplementary file 8 — Source data Fig. 1 [file 44321_2026_411_MOESM8_ESM.zip › Figure 1/1B/Ruxo 12 h.tif]

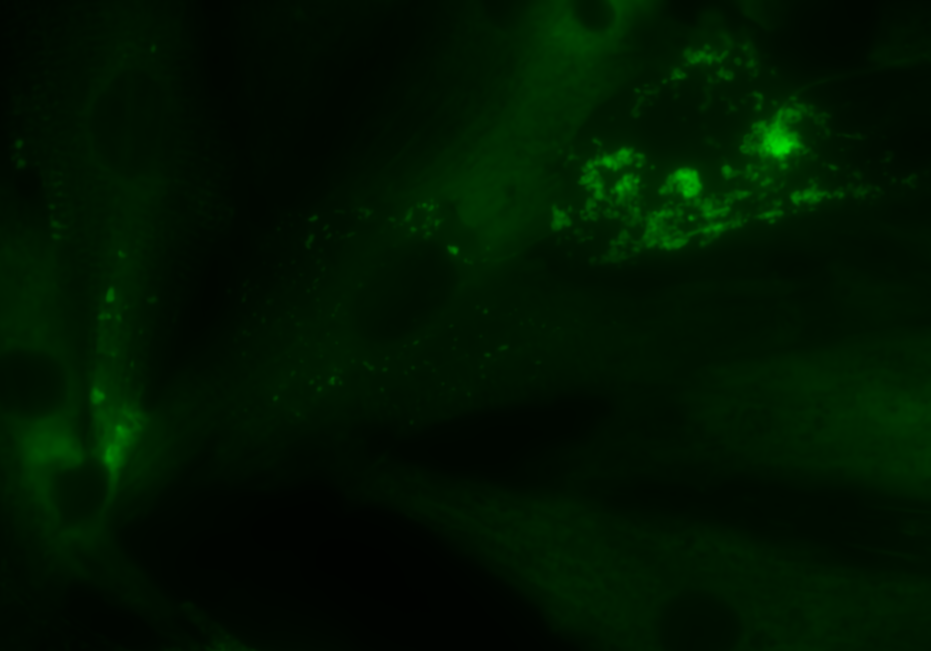

Supplement: Supplementary file 8 — Source data Fig. 1 [file 44321_2026_411_MOESM8_ESM.zip › Figure 1/1B/Ruxo 18 h.tif]

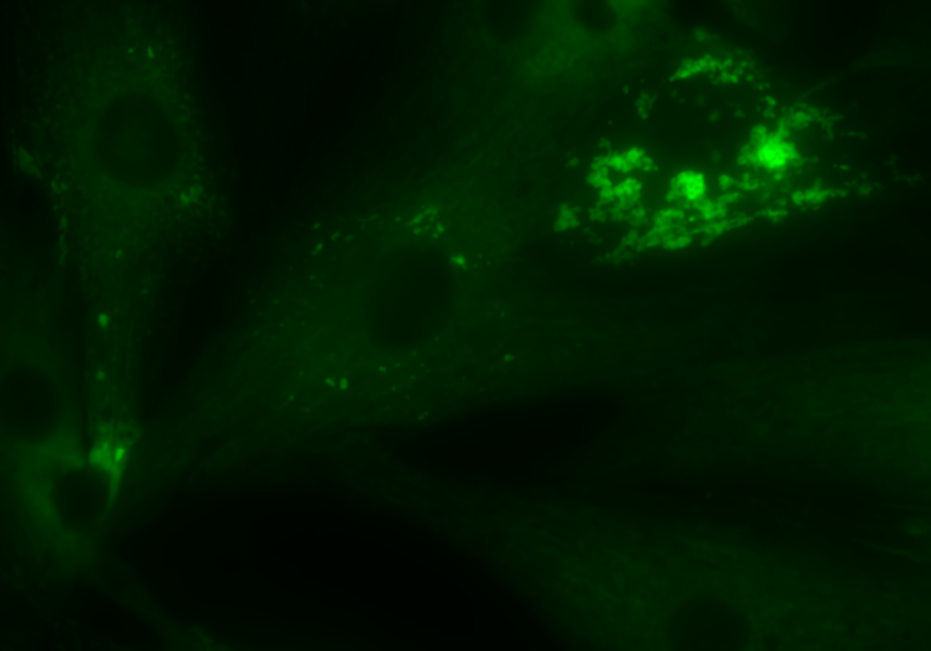

Supplement: Supplementary file 8 — Source data Fig. 1 [file 44321_2026_411_MOESM8_ESM.zip › Figure 1/1B/Ruxo 24 h.tif]

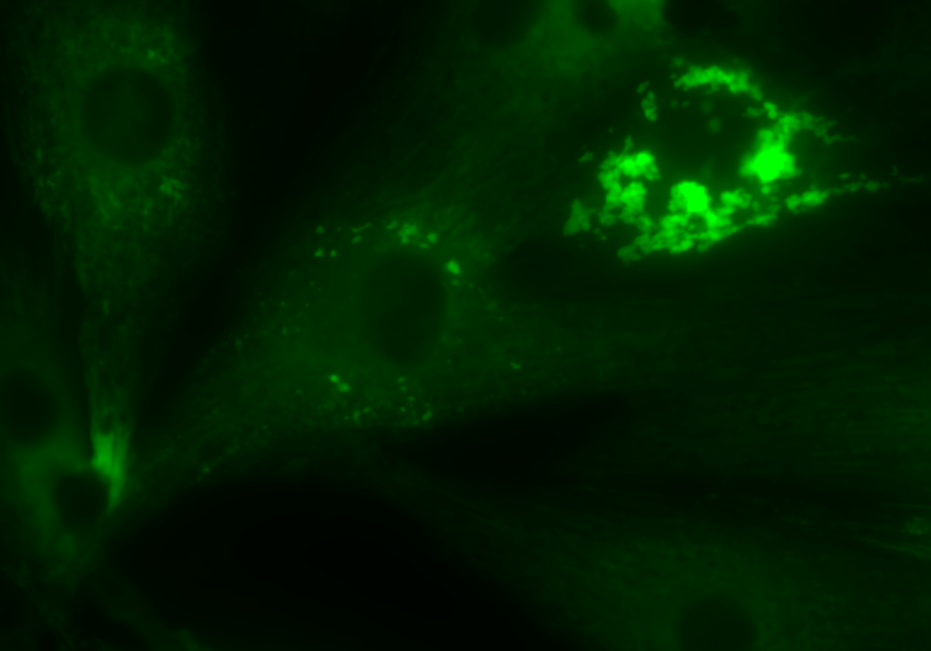

Supplement: Supplementary file 8 — Source data Fig. 1 [file 44321_2026_411_MOESM8_ESM.zip › Figure 1/1B/Ruxo 30 h.tif]

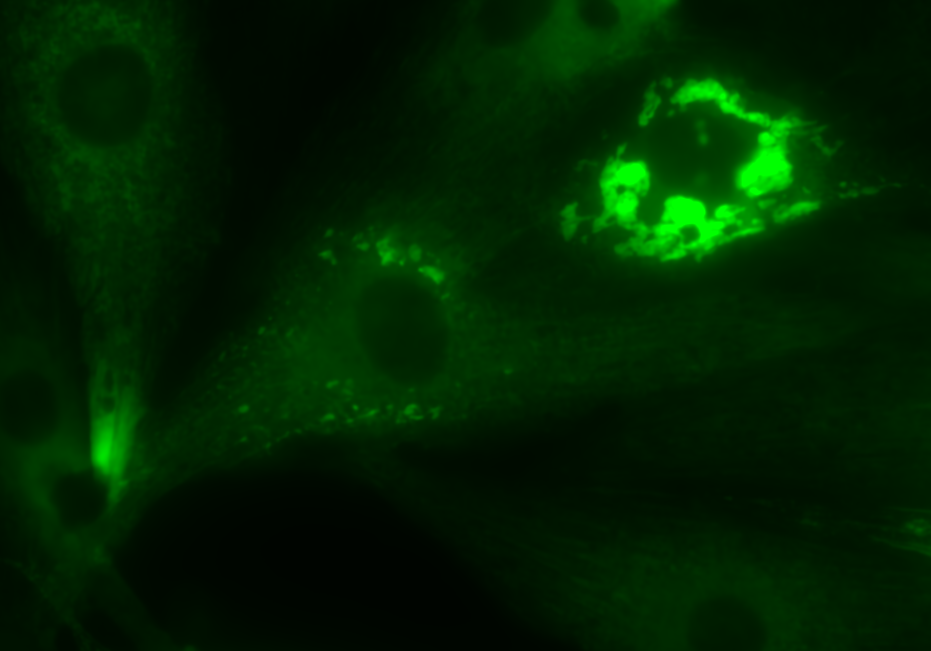

Supplement: Supplementary file 8 — Source data Fig. 1 [file 44321_2026_411_MOESM8_ESM.zip › Figure 1/1B/Ruxo 36 h.tif]

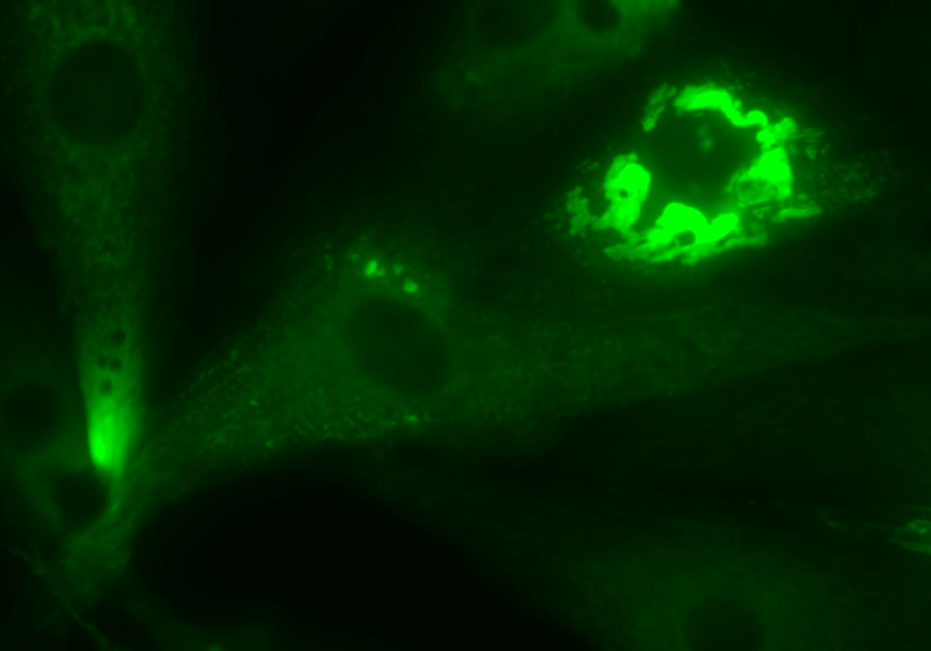

Supplement: Supplementary file 8 — Source data Fig. 1 [file 44321_2026_411_MOESM8_ESM.zip › Figure 1/1B/Ruxo 42 h.tif]

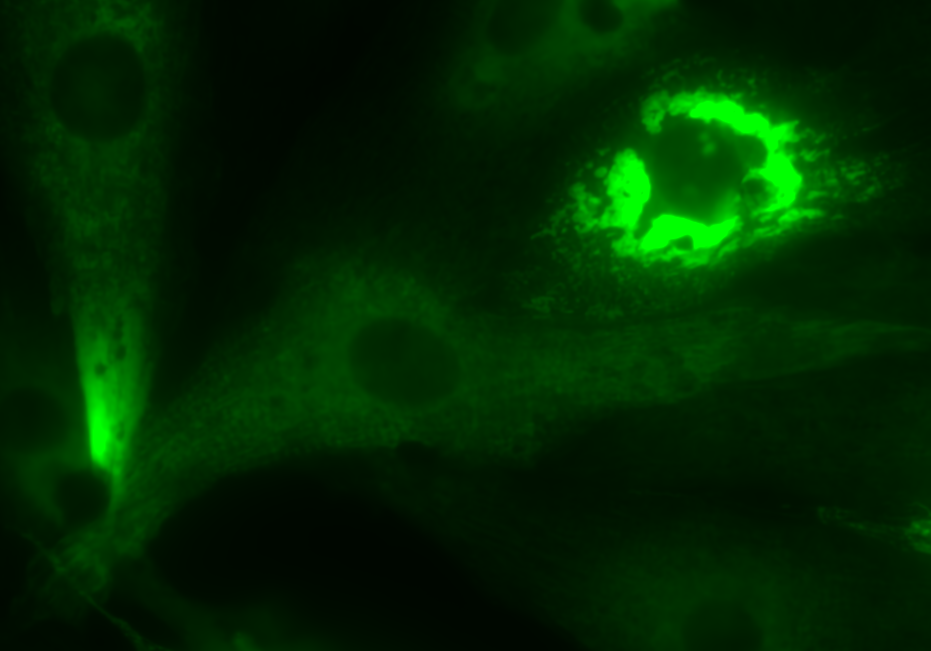

Supplement: Supplementary file 8 — Source data Fig. 1 [file 44321_2026_411_MOESM8_ESM.zip › Figure 1/1B/Ruxo 48 h.tif]

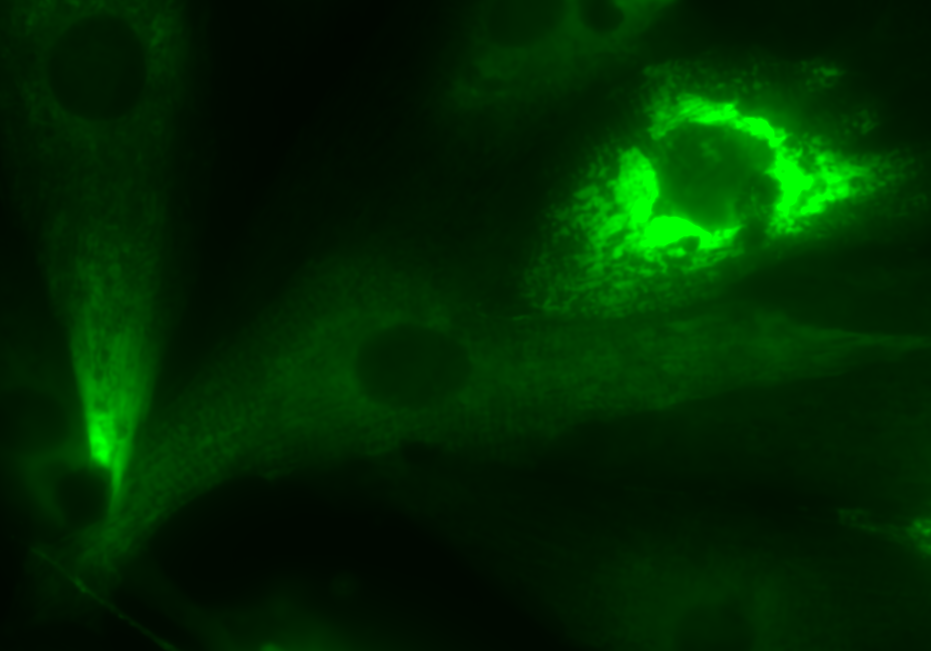

Supplement: Supplementary file 8 — Source data Fig. 1 [file 44321_2026_411_MOESM8_ESM.zip › Figure 1/1B/Ruxo 54 h.tif]

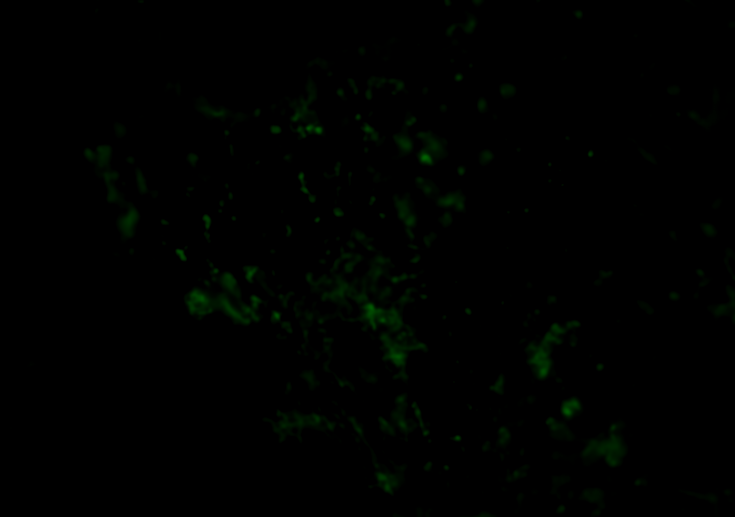

Supplement: Supplementary file 9 — Source data Fig. 2 [file 44321_2026_411_MOESM9_ESM.zip › Figure 2/2A/DMSO 0 h.tif]

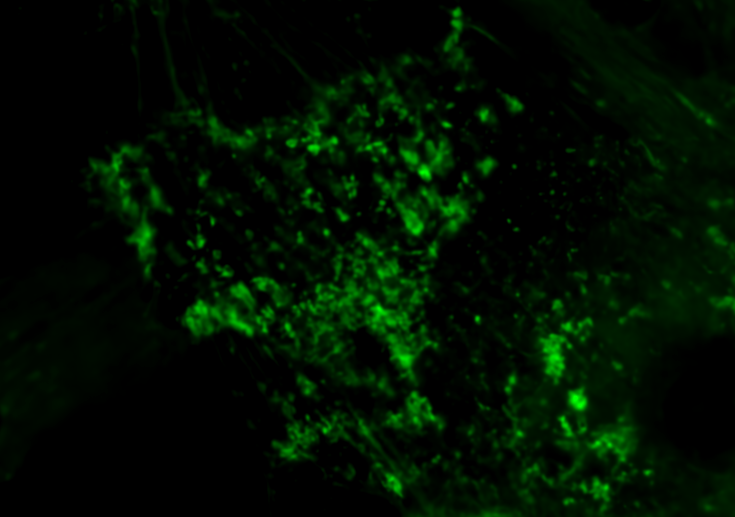

Supplement: Supplementary file 9 — Source data Fig. 2 [file 44321_2026_411_MOESM9_ESM.zip › Figure 2/2A/DMSO 12 h.tif]

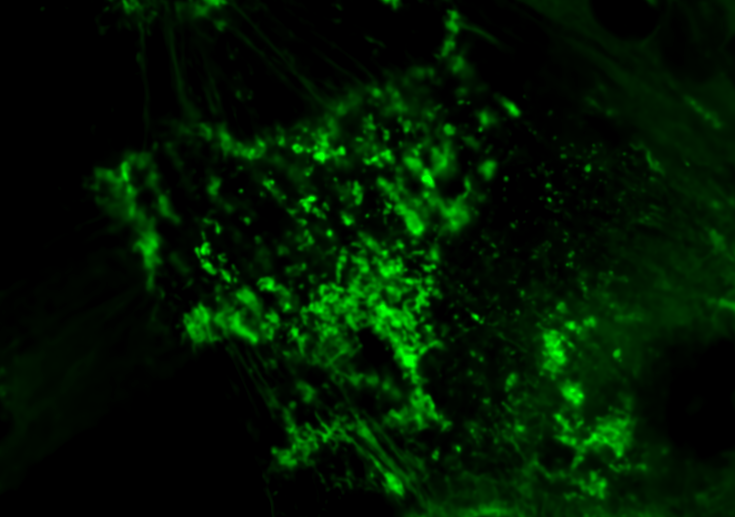

Supplement: Supplementary file 9 — Source data Fig. 2 [file 44321_2026_411_MOESM9_ESM.zip › Figure 2/2A/DMSO 18 h.tif]

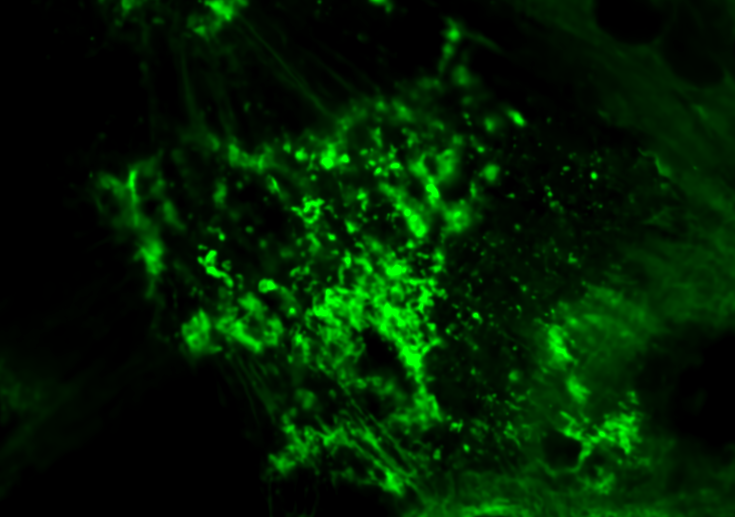

Supplement: Supplementary file 9 — Source data Fig. 2 [file 44321_2026_411_MOESM9_ESM.zip › Figure 2/2A/DMSO 24 h.tif]

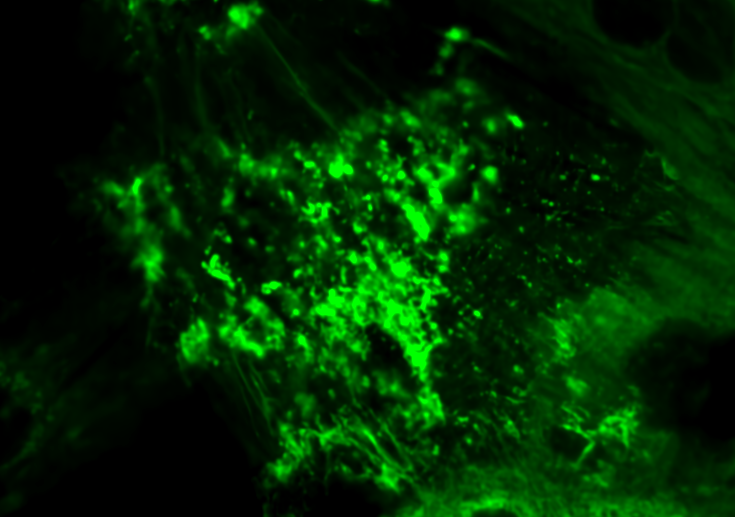

Supplement: Supplementary file 9 — Source data Fig. 2 [file 44321_2026_411_MOESM9_ESM.zip › Figure 2/2A/DMSO 30 h.tif]

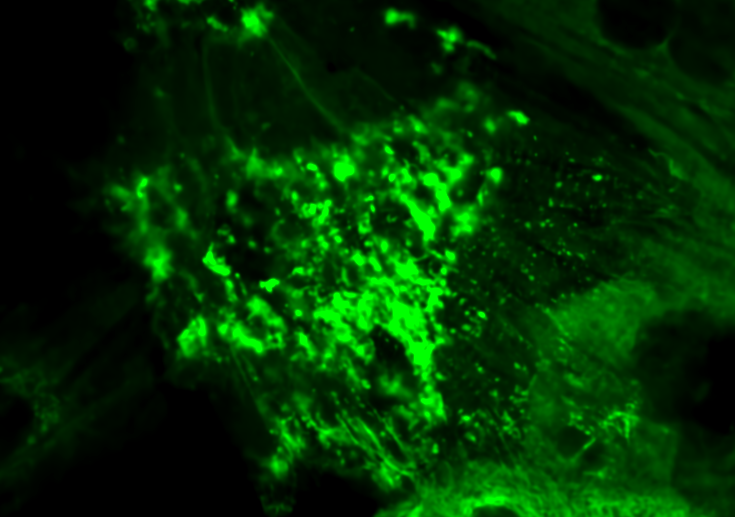

Supplement: Supplementary file 9 — Source data Fig. 2 [file 44321_2026_411_MOESM9_ESM.zip › Figure 2/2A/DMSO 36 h.tif]

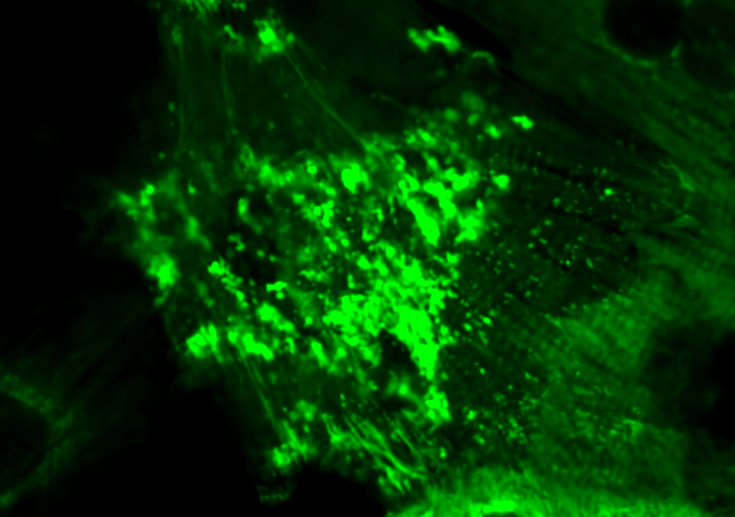

Supplement: Supplementary file 9 — Source data Fig. 2 [file 44321_2026_411_MOESM9_ESM.zip › Figure 2/2A/DMSO 42 h.tif]

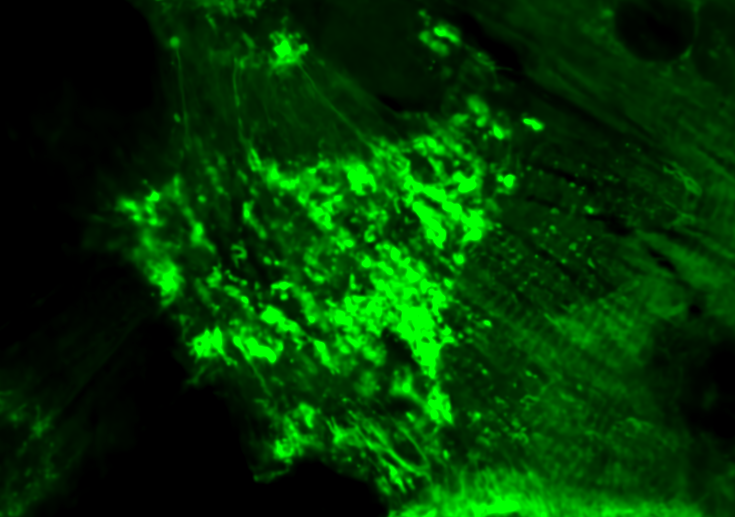

Supplement: Supplementary file 9 — Source data Fig. 2 [file 44321_2026_411_MOESM9_ESM.zip › Figure 2/2A/DMSO 48 h.tif]

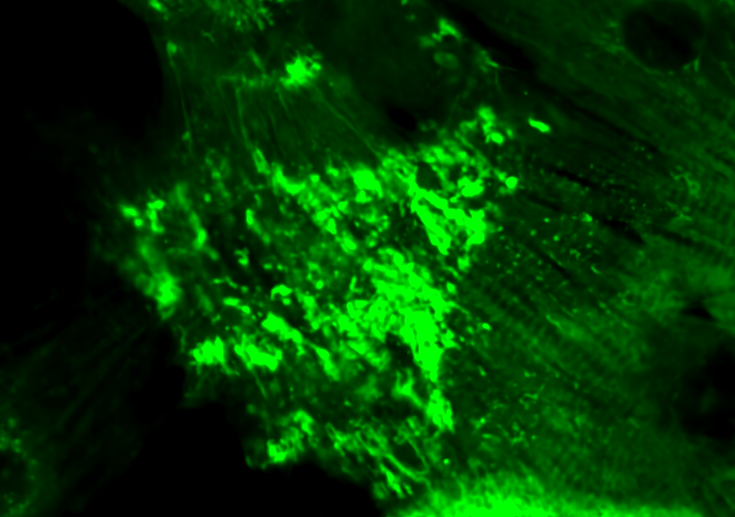

Supplement: Supplementary file 9 — Source data Fig. 2 [file 44321_2026_411_MOESM9_ESM.zip › Figure 2/2A/DMSO 54 h.tif]

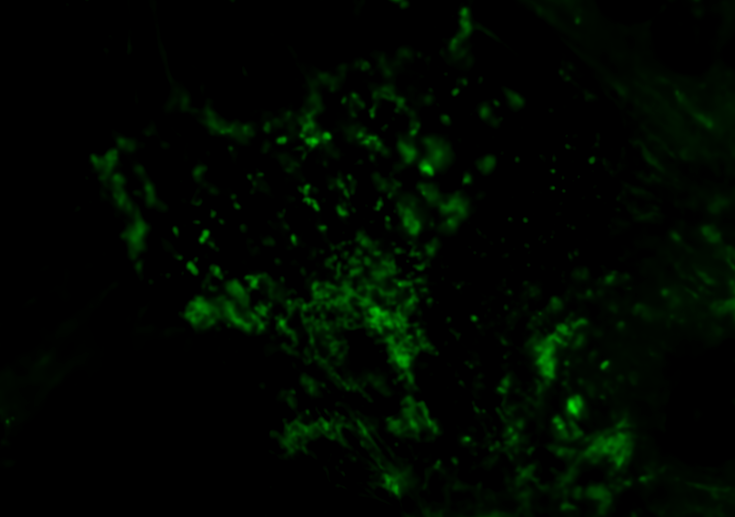

Supplement: Supplementary file 9 — Source data Fig. 2 [file 44321_2026_411_MOESM9_ESM.zip › Figure 2/2A/DMSO 6 h.tif]

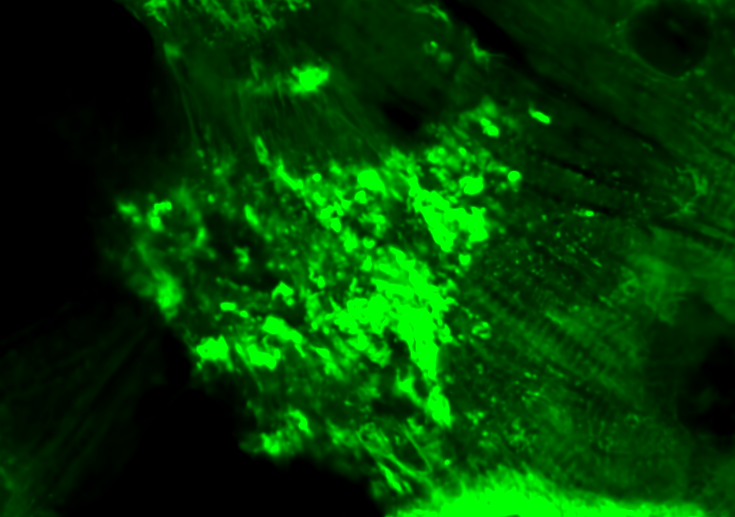

Supplement: Supplementary file 9 — Source data Fig. 2 [file 44321_2026_411_MOESM9_ESM.zip › Figure 2/2A/DMSO 60 h.tif]

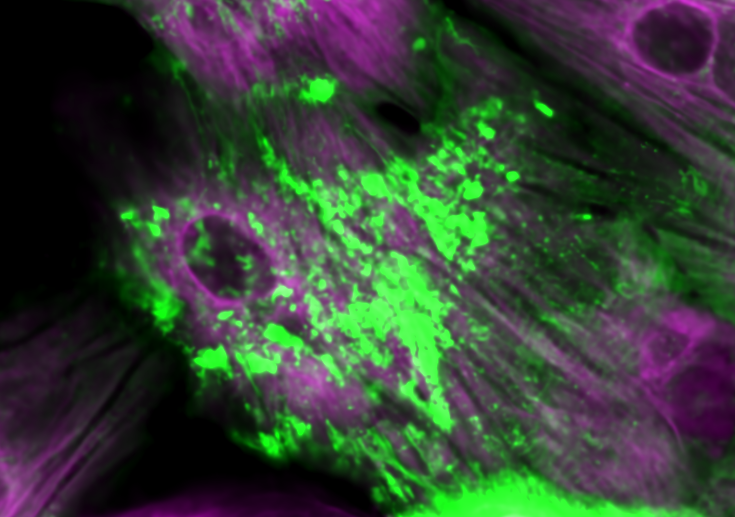

Supplement: Supplementary file 9 — Source data Fig. 2 [file 44321_2026_411_MOESM9_ESM.zip › Figure 2/2A/DMSO 66 h.tif]

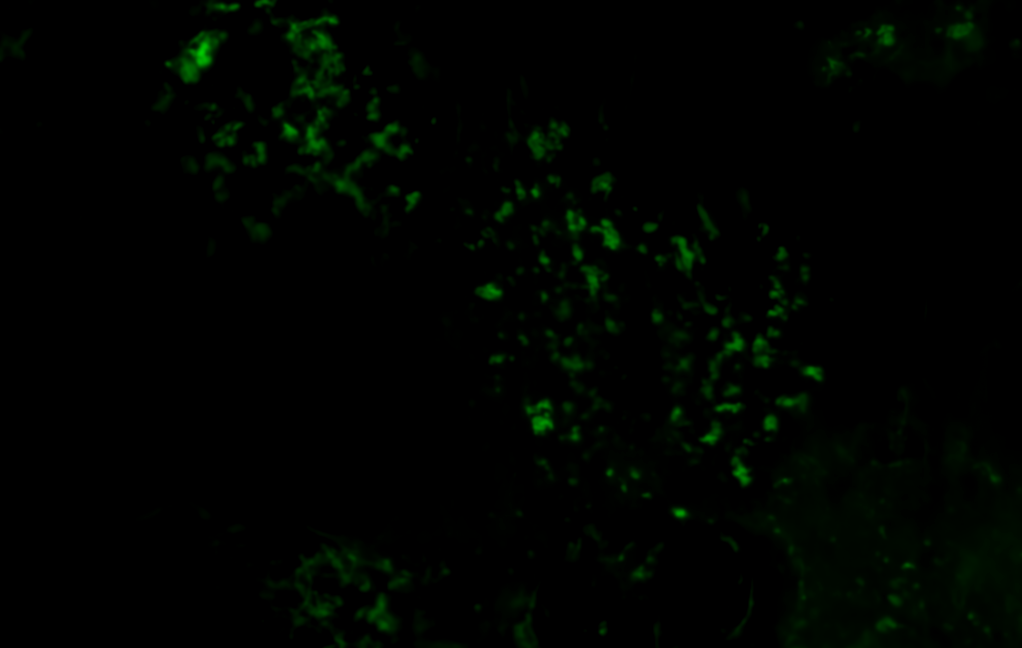

Supplement: Supplementary file 9 — Source data Fig. 2 [file 44321_2026_411_MOESM9_ESM.zip › Figure 2/2A/Ruxolitinib 0 h.tif]

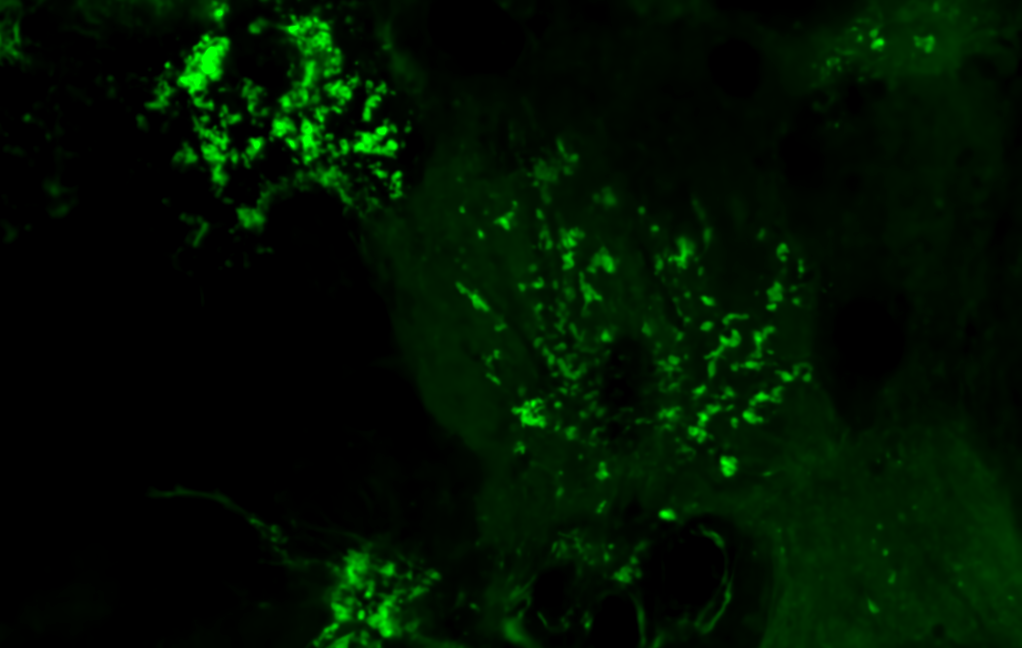

Supplement: Supplementary file 9 — Source data Fig. 2 [file 44321_2026_411_MOESM9_ESM.zip › Figure 2/2A/Ruxolitinib 12 h.tif]

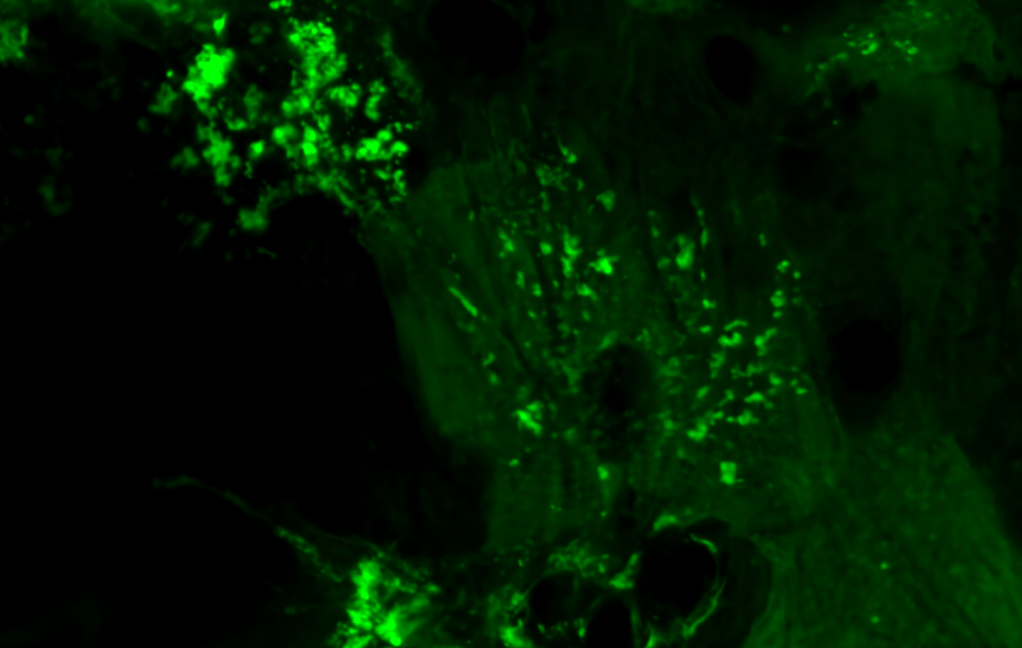

Supplement: Supplementary file 9 — Source data Fig. 2 [file 44321_2026_411_MOESM9_ESM.zip › Figure 2/2A/Ruxolitinib 18 h.tif]

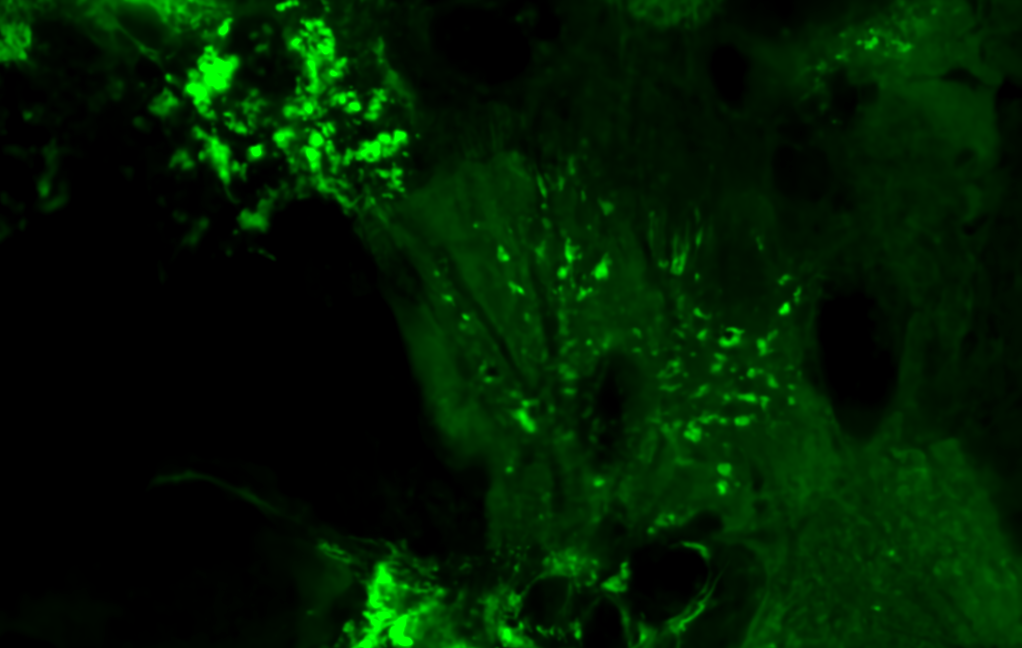

Supplement: Supplementary file 9 — Source data Fig. 2 [file 44321_2026_411_MOESM9_ESM.zip › Figure 2/2A/Ruxolitinib 24 h.tif]

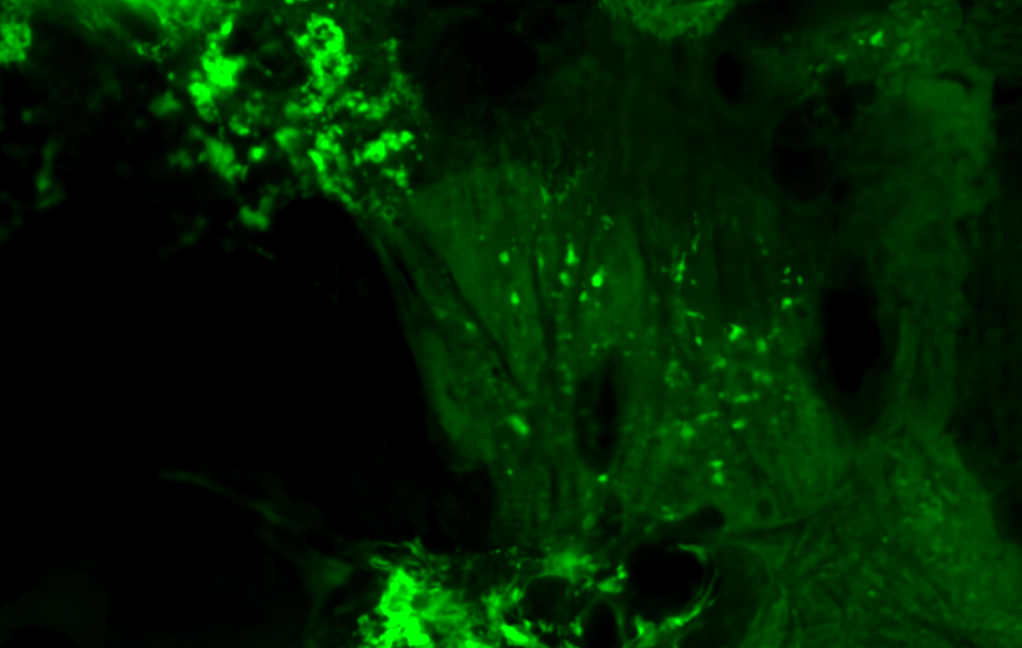

Supplement: Supplementary file 9 — Source data Fig. 2 [file 44321_2026_411_MOESM9_ESM.zip › Figure 2/2A/Ruxolitinib 30 h.tif]

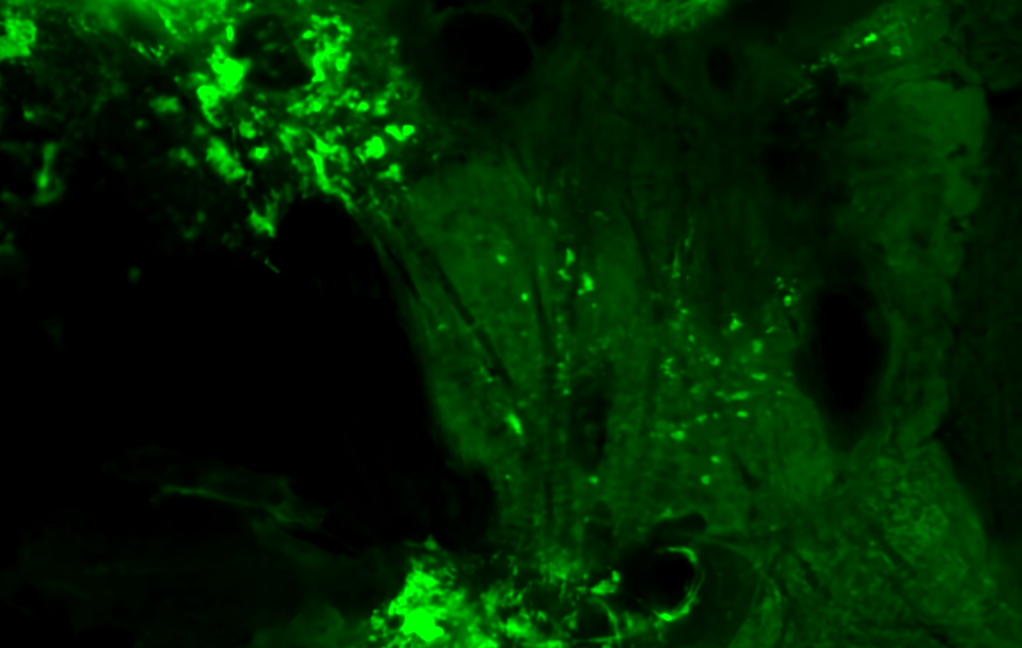

Supplement: Supplementary file 9 — Source data Fig. 2 [file 44321_2026_411_MOESM9_ESM.zip › Figure 2/2A/Ruxolitinib 36 h.tif]

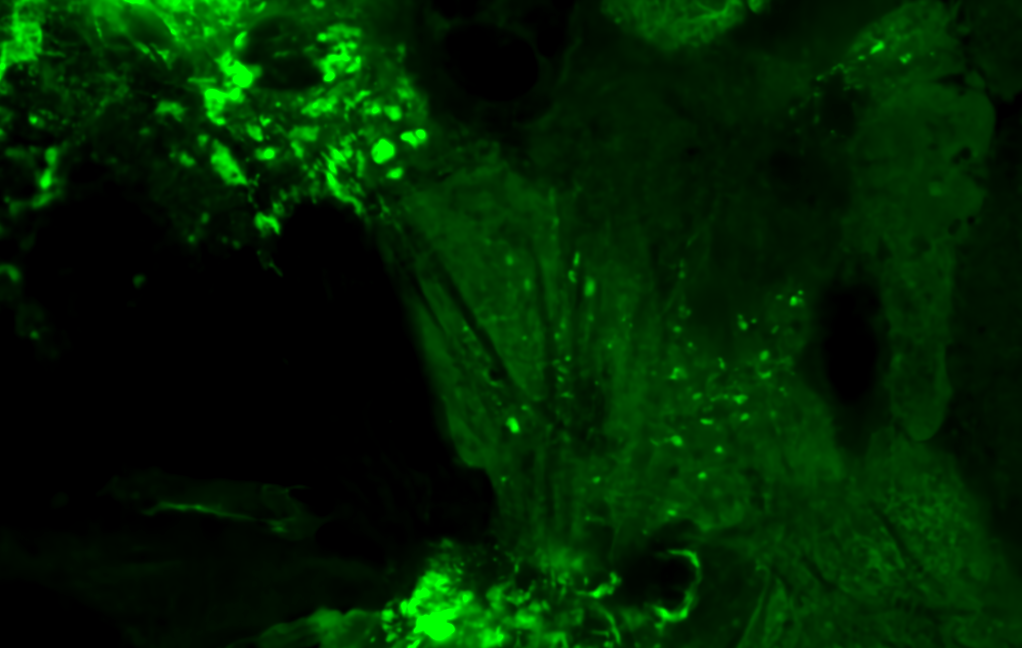

Supplement: Supplementary file 9 — Source data Fig. 2 [file 44321_2026_411_MOESM9_ESM.zip › Figure 2/2A/Ruxolitinib 42 h.tif]

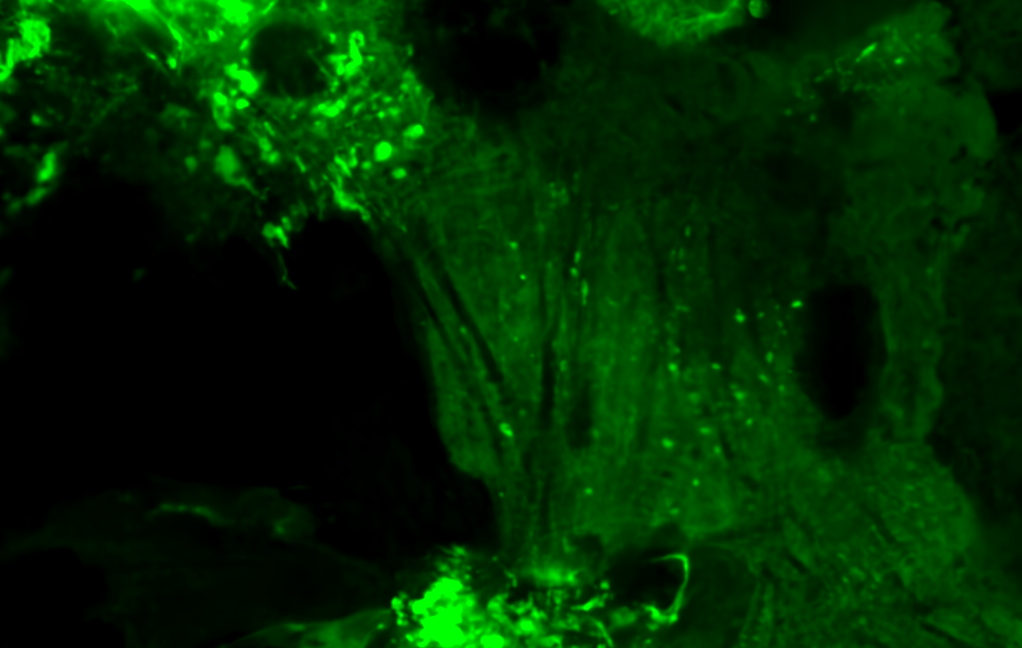

Supplement: Supplementary file 9 — Source data Fig. 2 [file 44321_2026_411_MOESM9_ESM.zip › Figure 2/2A/Ruxolitinib 48 h.tif]

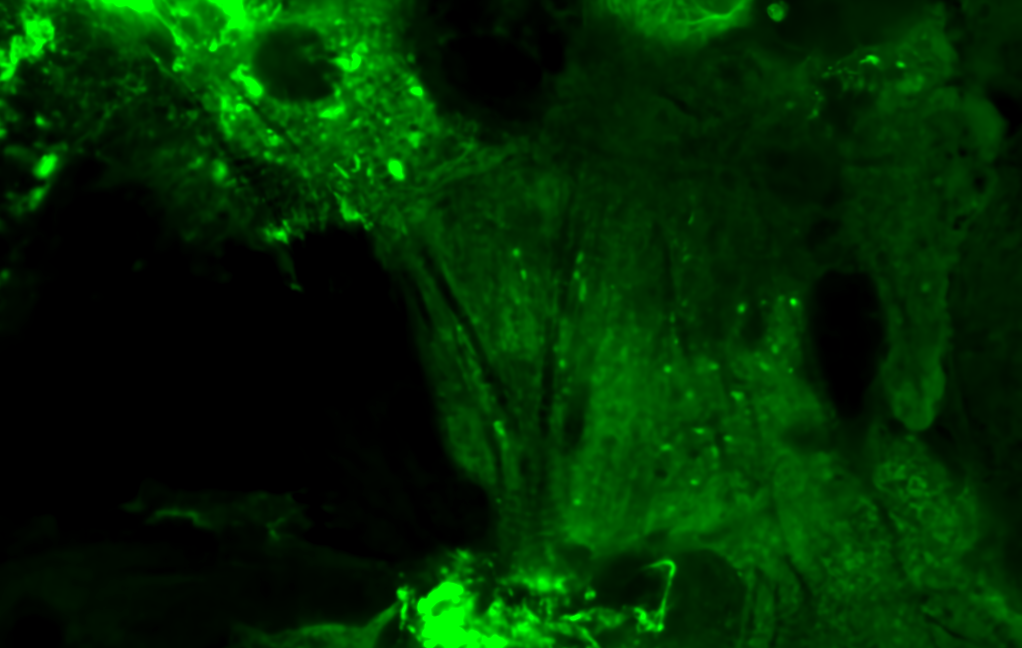

Supplement: Supplementary file 9 — Source data Fig. 2 [file 44321_2026_411_MOESM9_ESM.zip › Figure 2/2A/Ruxolitinib 54 h.tif]

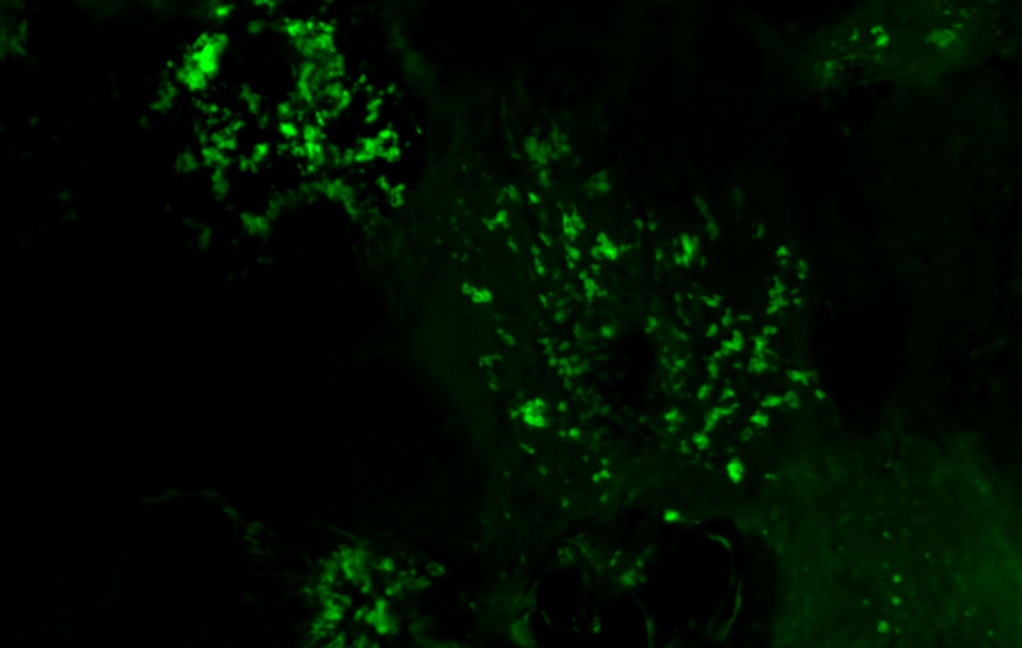

Supplement: Supplementary file 9 — Source data Fig. 2 [file 44321_2026_411_MOESM9_ESM.zip › Figure 2/2A/Ruxolitinib 6 h.tif]

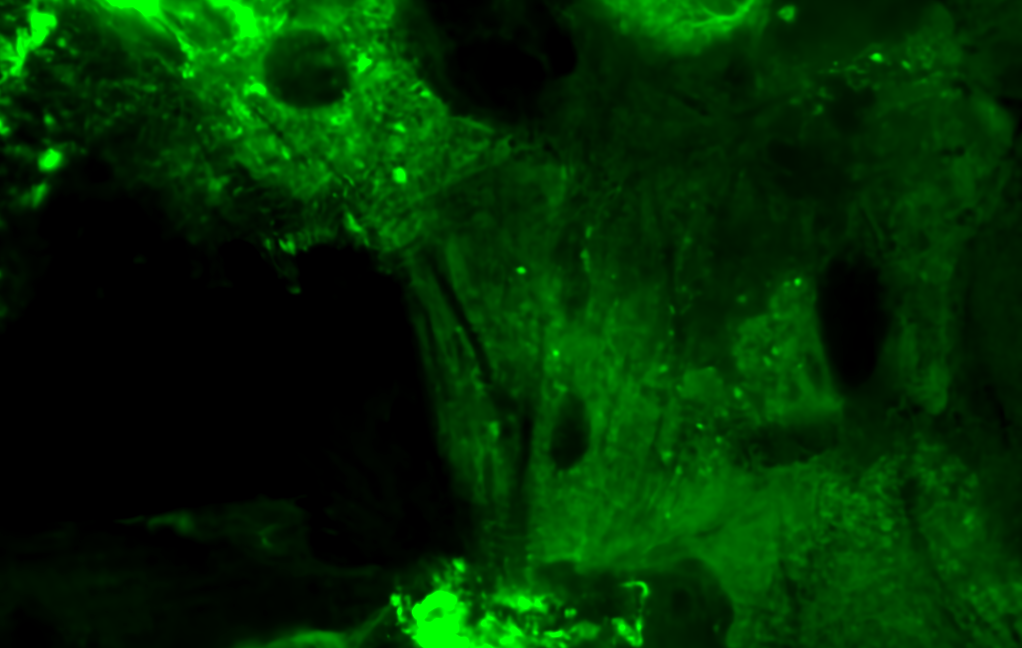

Supplement: Supplementary file 9 — Source data Fig. 2 [file 44321_2026_411_MOESM9_ESM.zip › Figure 2/2A/Ruxolitinib 60 h.tif]

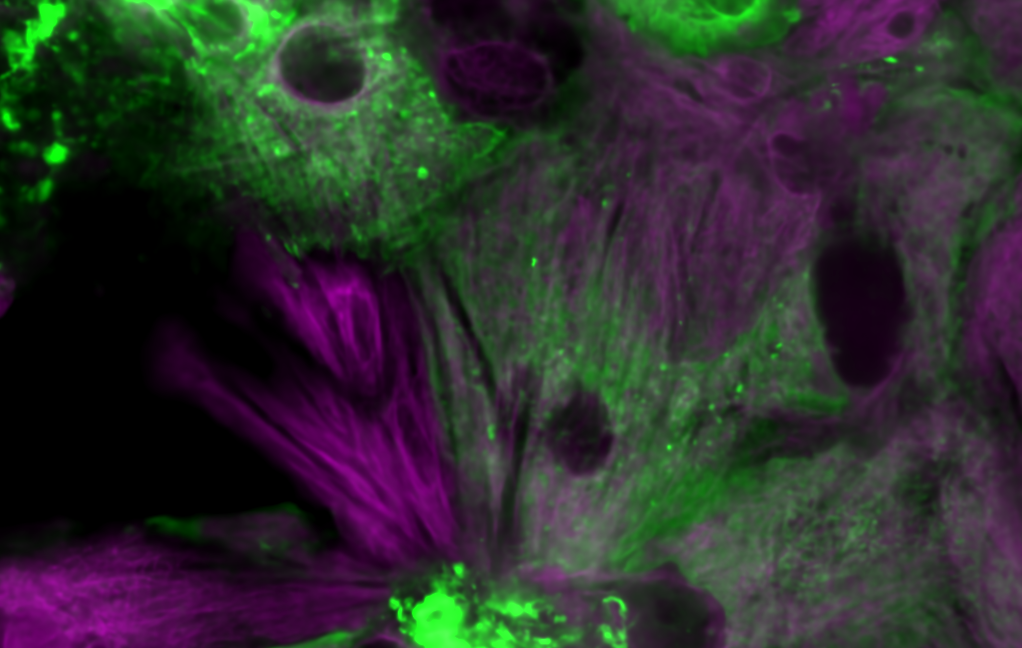

Supplement: Supplementary file 9 — Source data Fig. 2 [file 44321_2026_411_MOESM9_ESM.zip › Figure 2/2A/Ruxolitinib 66 h.tif]

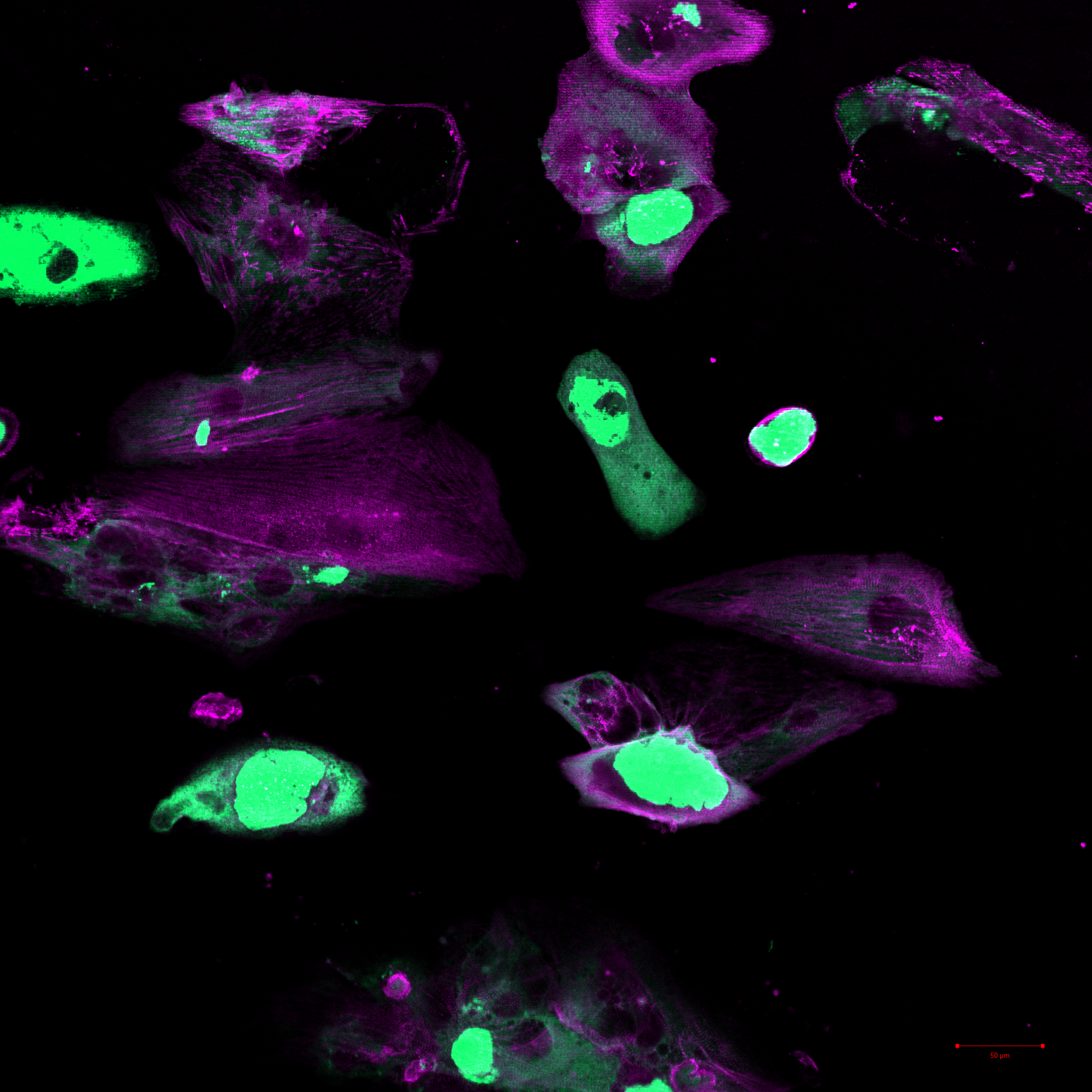

Supplement: Supplementary file 9 — Source data Fig. 2 [file 44321_2026_411_MOESM9_ESM.zip › Figure 2/2C/DMSO-I.tif]

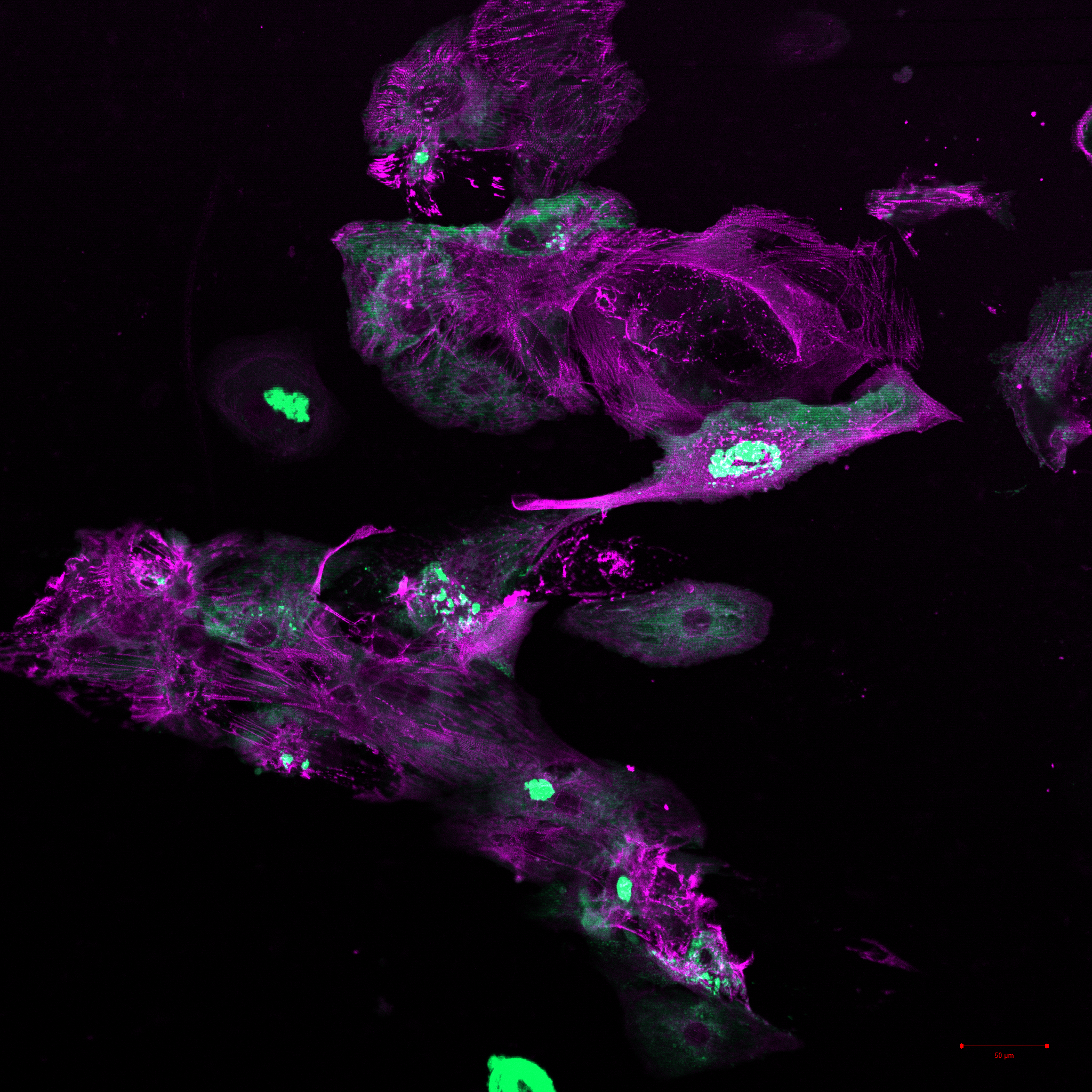

Supplement: Supplementary file 9 — Source data Fig. 2 [file 44321_2026_411_MOESM9_ESM.zip › Figure 2/2C/DMSO-II.tif]

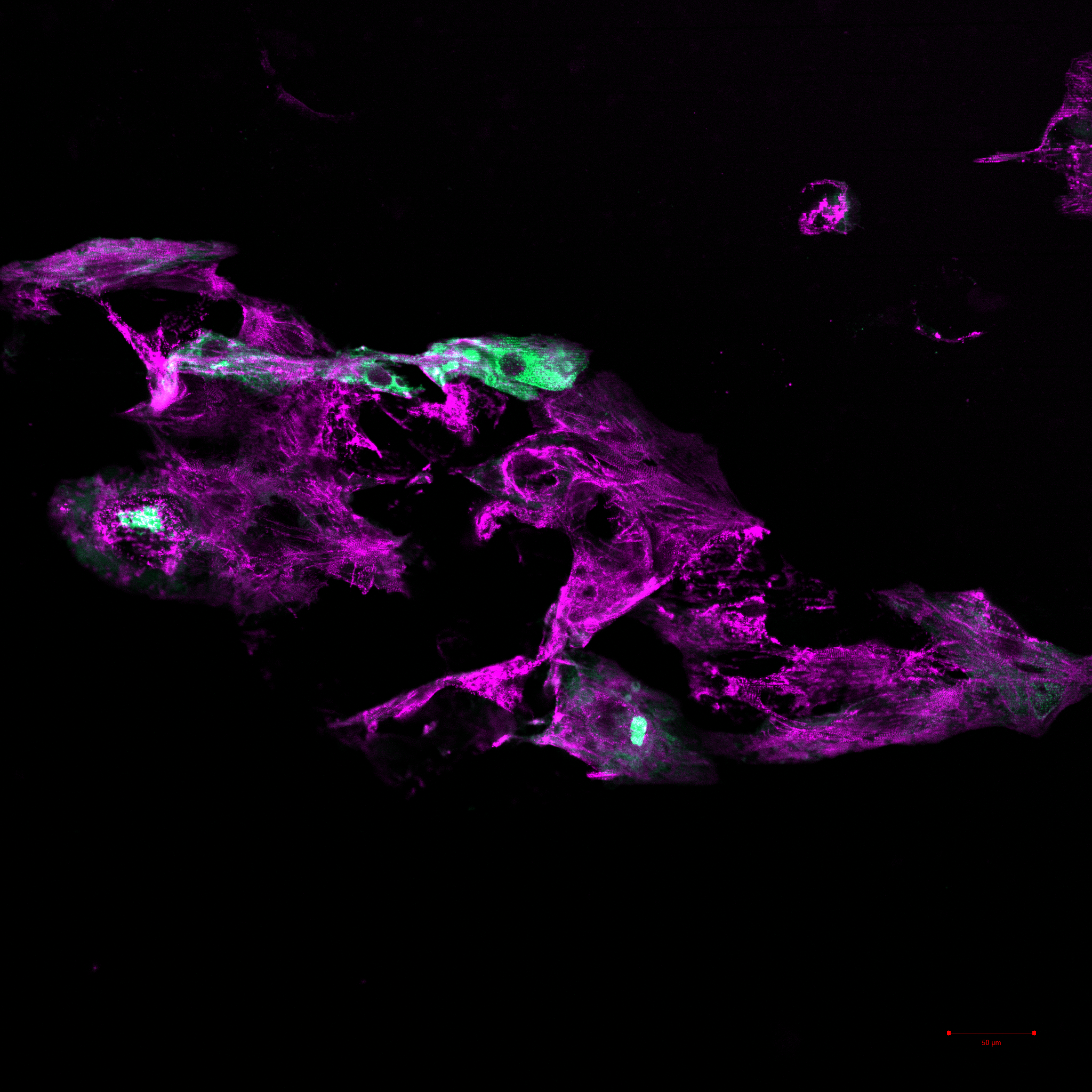

Supplement: Supplementary file 9 — Source data Fig. 2 [file 44321_2026_411_MOESM9_ESM.zip › Figure 2/2C/Filgotinib.tif]

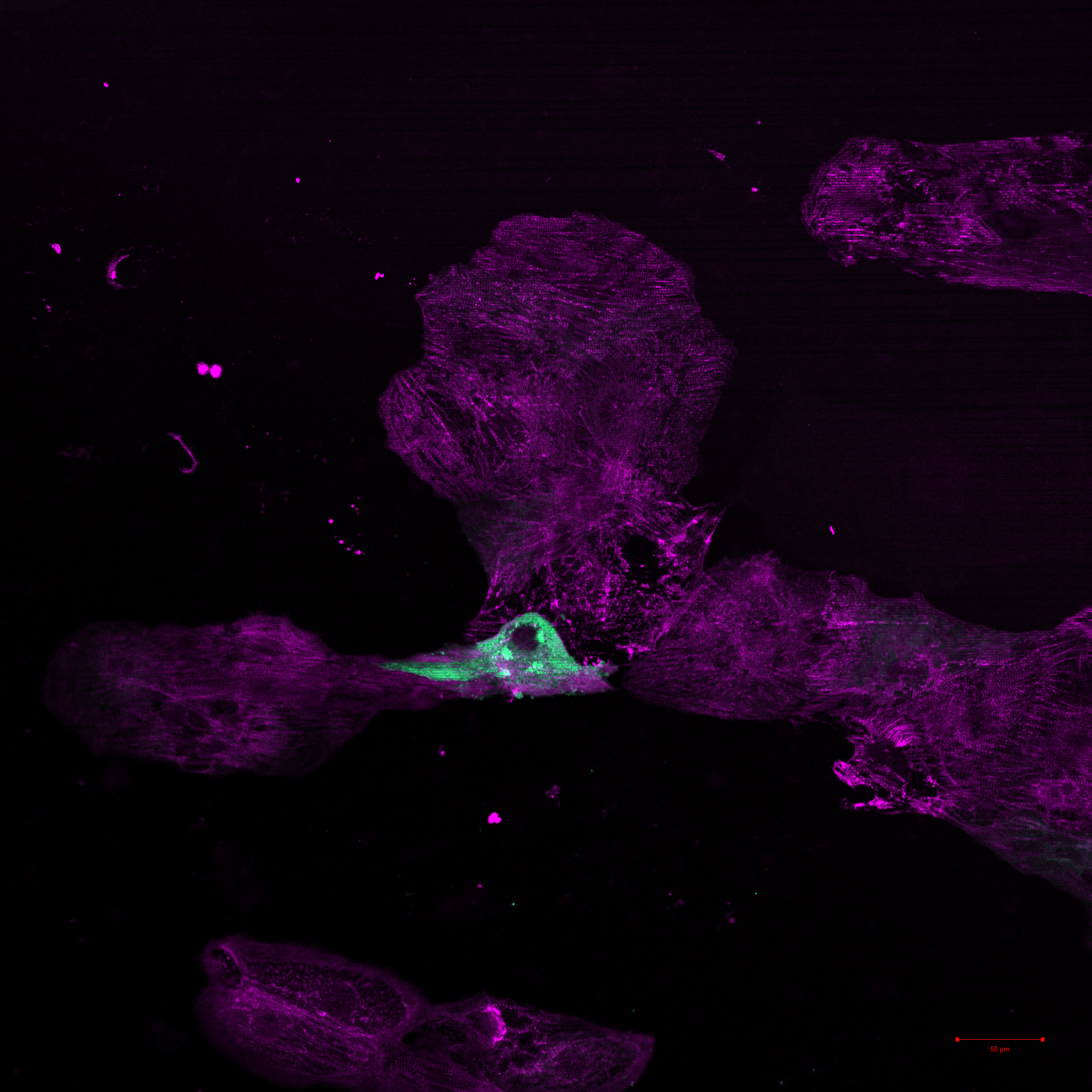

Supplement: Supplementary file 9 — Source data Fig. 2 [file 44321_2026_411_MOESM9_ESM.zip › Figure 2/2C/Ruxolitinib.tif]

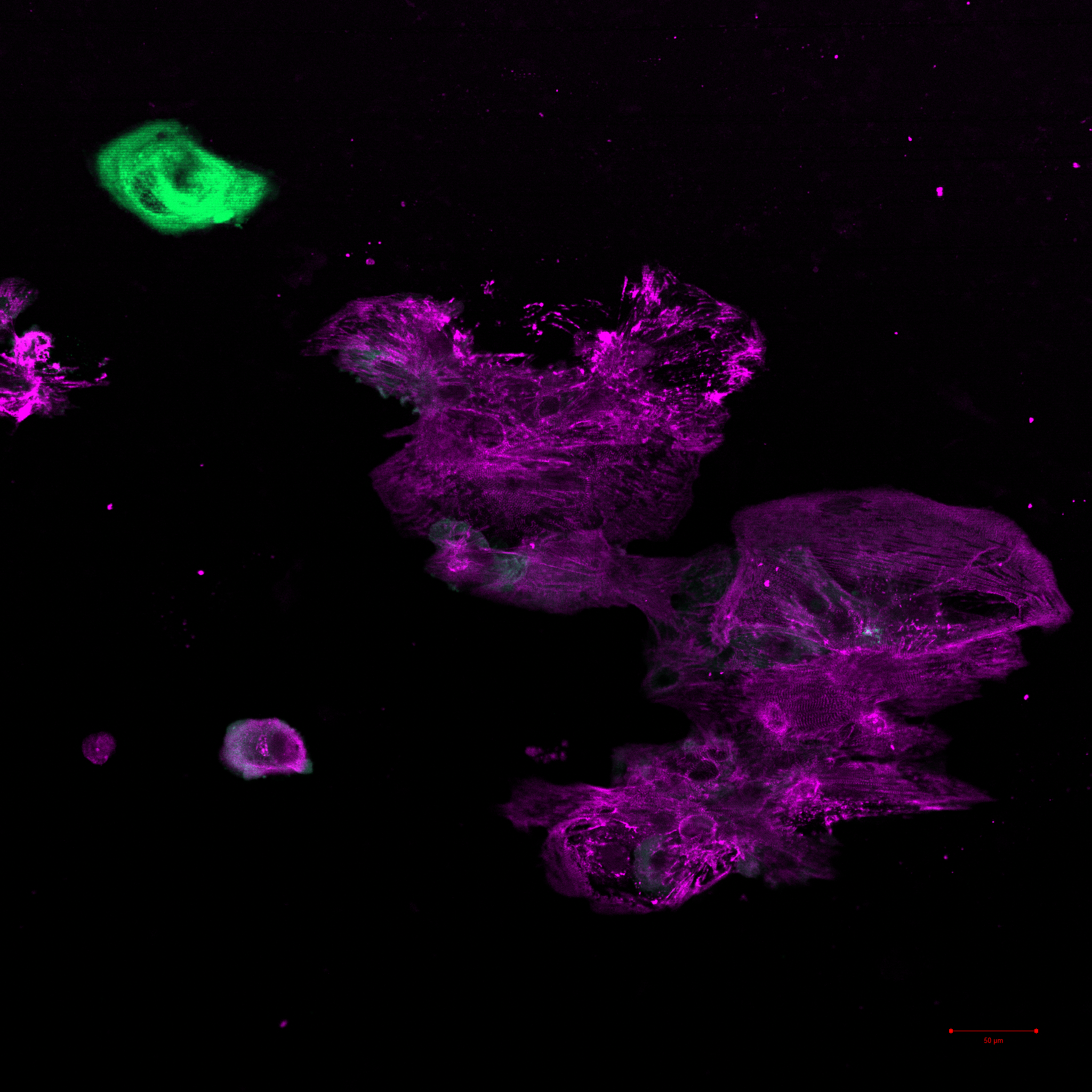

Supplement: Supplementary file 9 — Source data Fig. 2 [file 44321_2026_411_MOESM9_ESM.zip › Figure 2/2C/Solcitinib.tif]

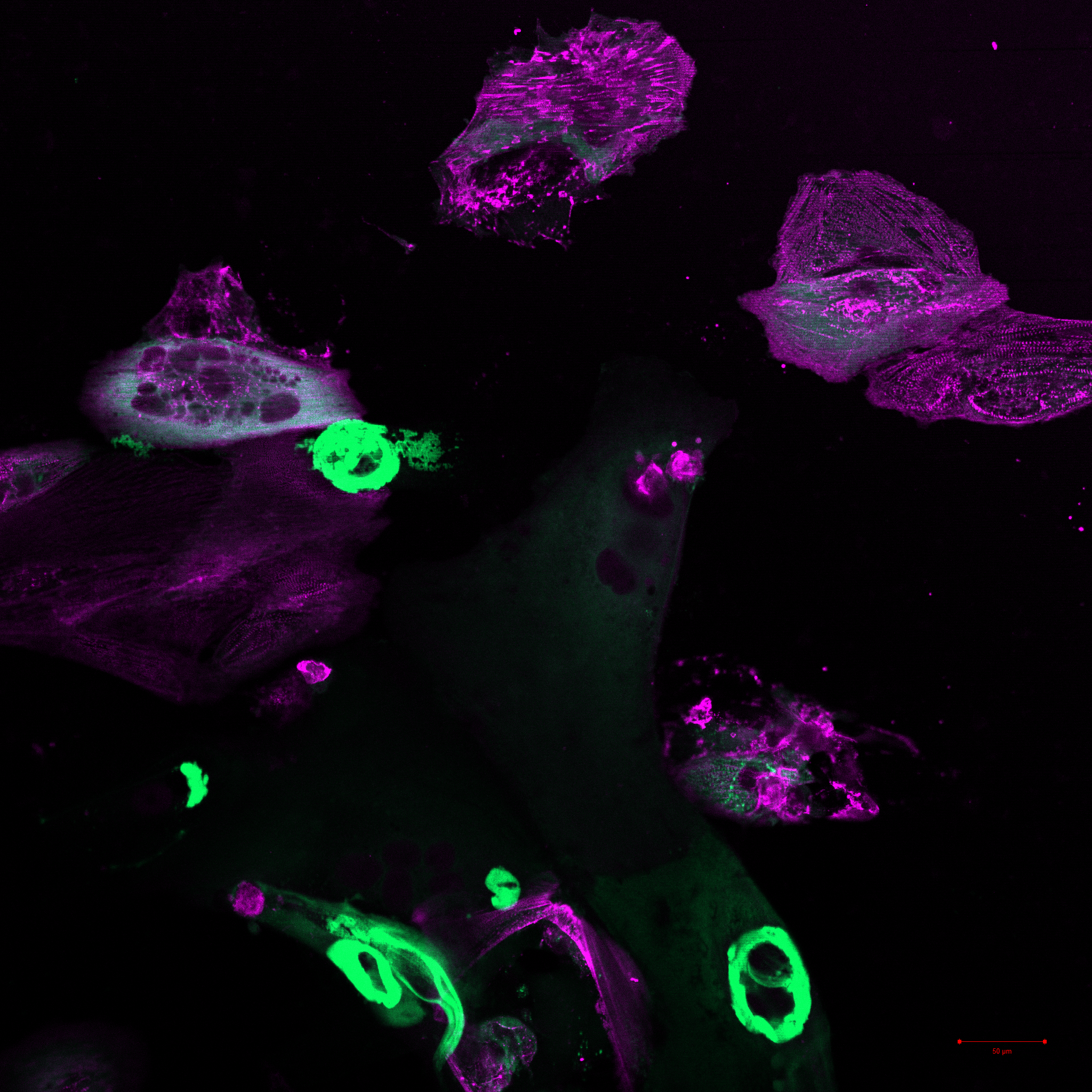

Supplement: Supplementary file 9 — Source data Fig. 2 [file 44321_2026_411_MOESM9_ESM.zip › Figure 2/2C/Upadacitinib.tif]

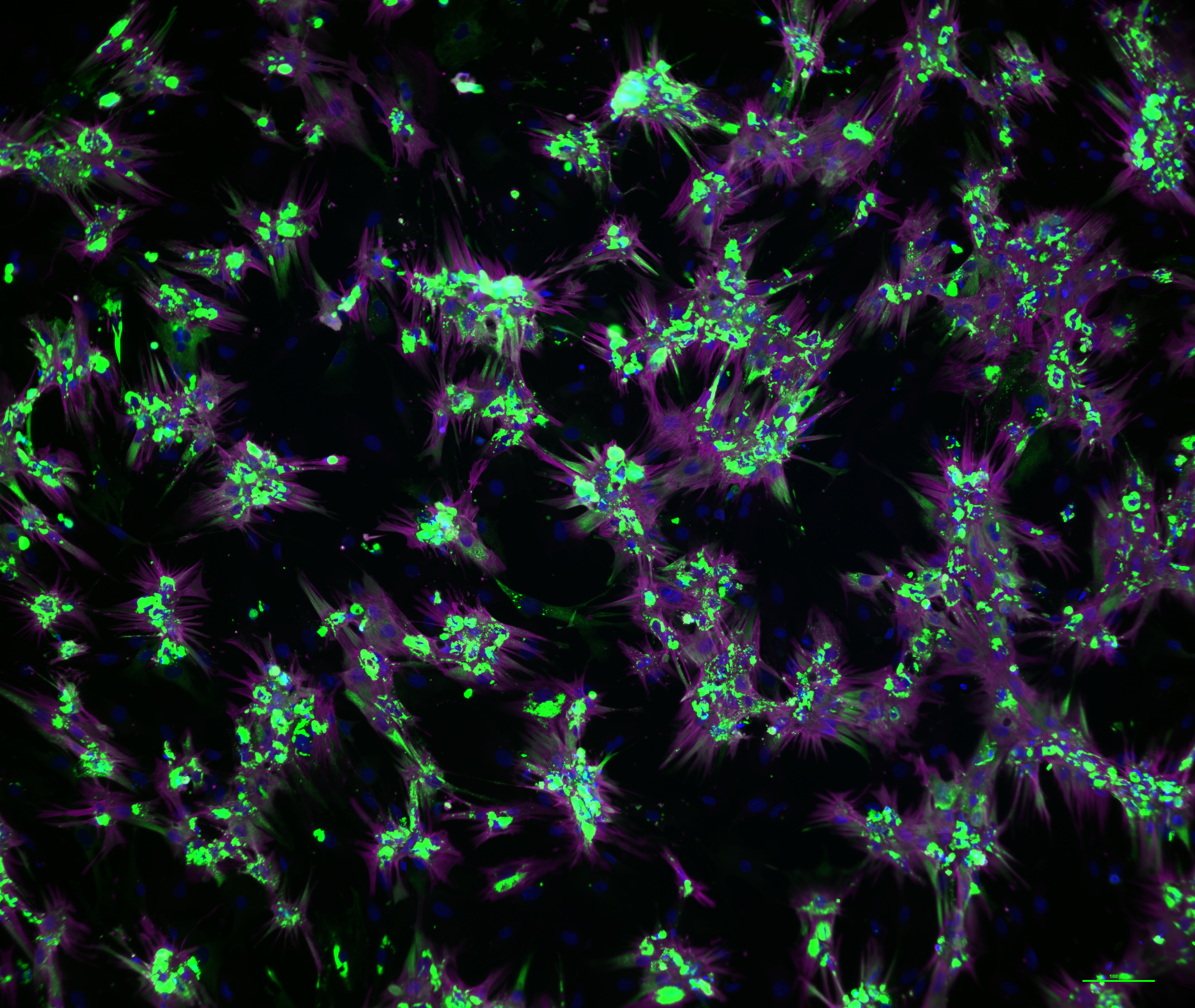

Supplement: Supplementary file 10 — Source data Fig. 3 [file 44321_2026_411_MOESM10_ESM.zip › Figure 3/3A/DMSO_RGB.tif]

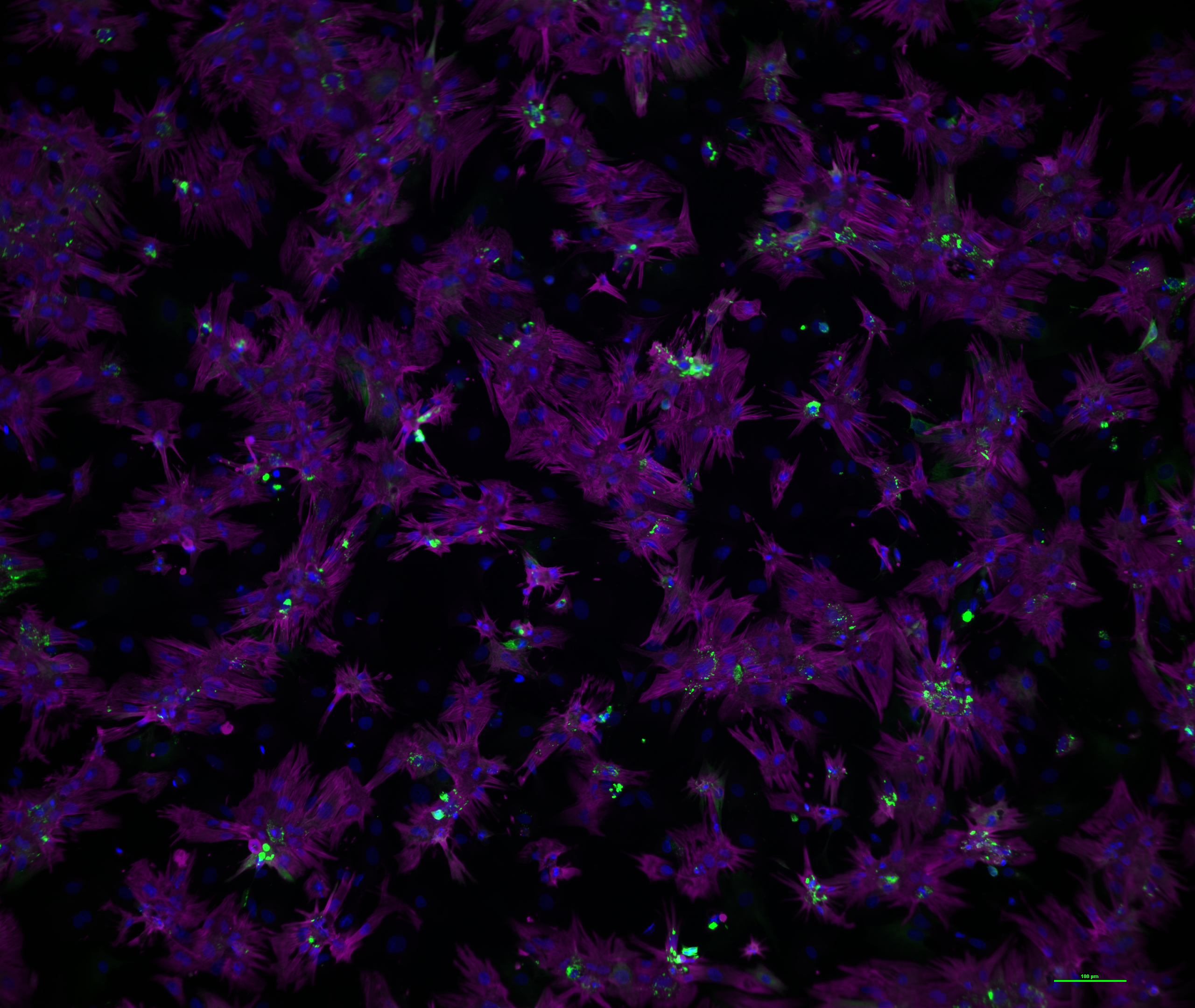

Supplement: Supplementary file 10 — Source data Fig. 3 [file 44321_2026_411_MOESM10_ESM.zip › Figure 3/3A/Ruxo_RGB.tif]

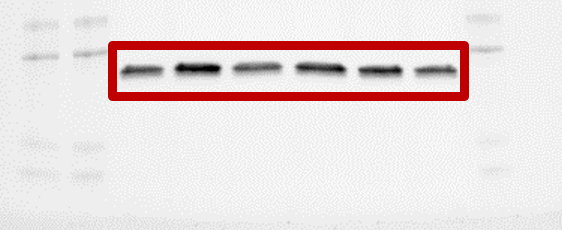

Supplement: Supplementary file 10 — Source data Fig. 3 [file 44321_2026_411_MOESM10_ESM.zip › Figure 3/3A/western GAPDH 1 - JAK1.tif]

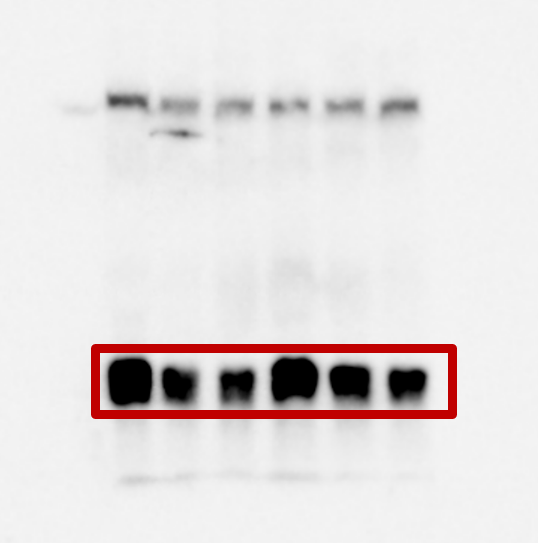

Supplement: Supplementary file 10 — Source data Fig. 3 [file 44321_2026_411_MOESM10_ESM.zip › Figure 3/3A/western GAPDH 2 - JAK2.tif]

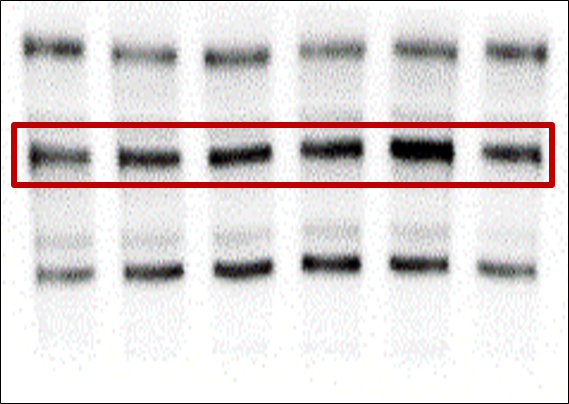

Supplement: Supplementary file 10 — Source data Fig. 3 [file 44321_2026_411_MOESM10_ESM.zip › Figure 3/3A/western JAK1.tiff]

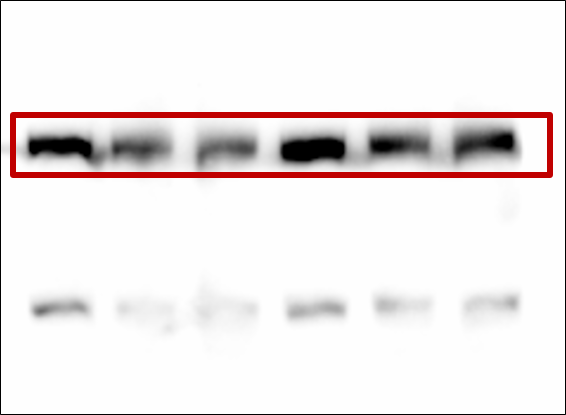

Supplement: Supplementary file 10 — Source data Fig. 3 [file 44321_2026_411_MOESM10_ESM.zip › Figure 3/3A/western JAK2.tiff]

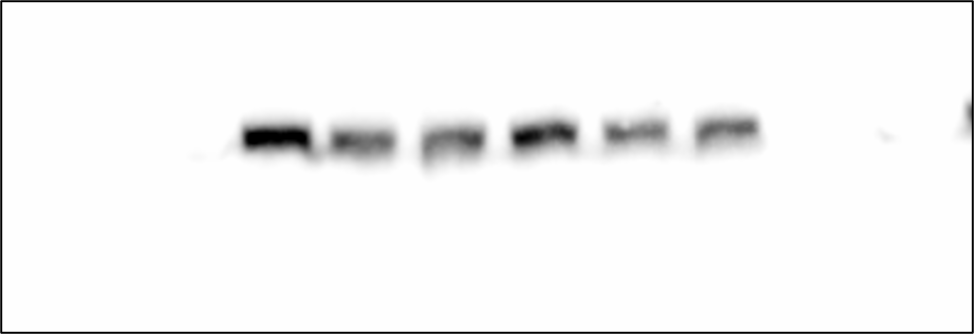

Supplement: Supplementary file 10 — Source data Fig. 3 [file 44321_2026_411_MOESM10_ESM.zip › Figure 3/3A/western STAT3.tif]

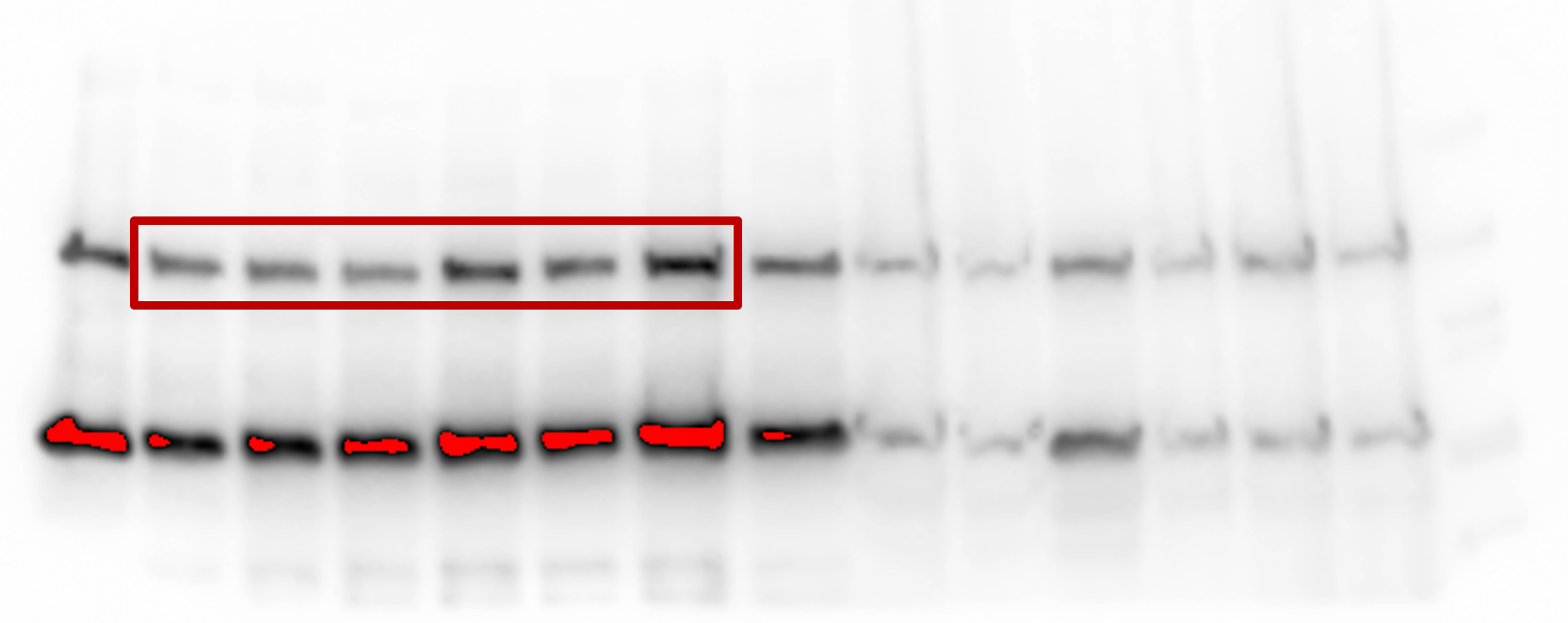

Supplement: Supplementary file 10 — Source data Fig. 3 [file 44321_2026_411_MOESM10_ESM.zip › Figure 3/3B/western ACTN2.png]

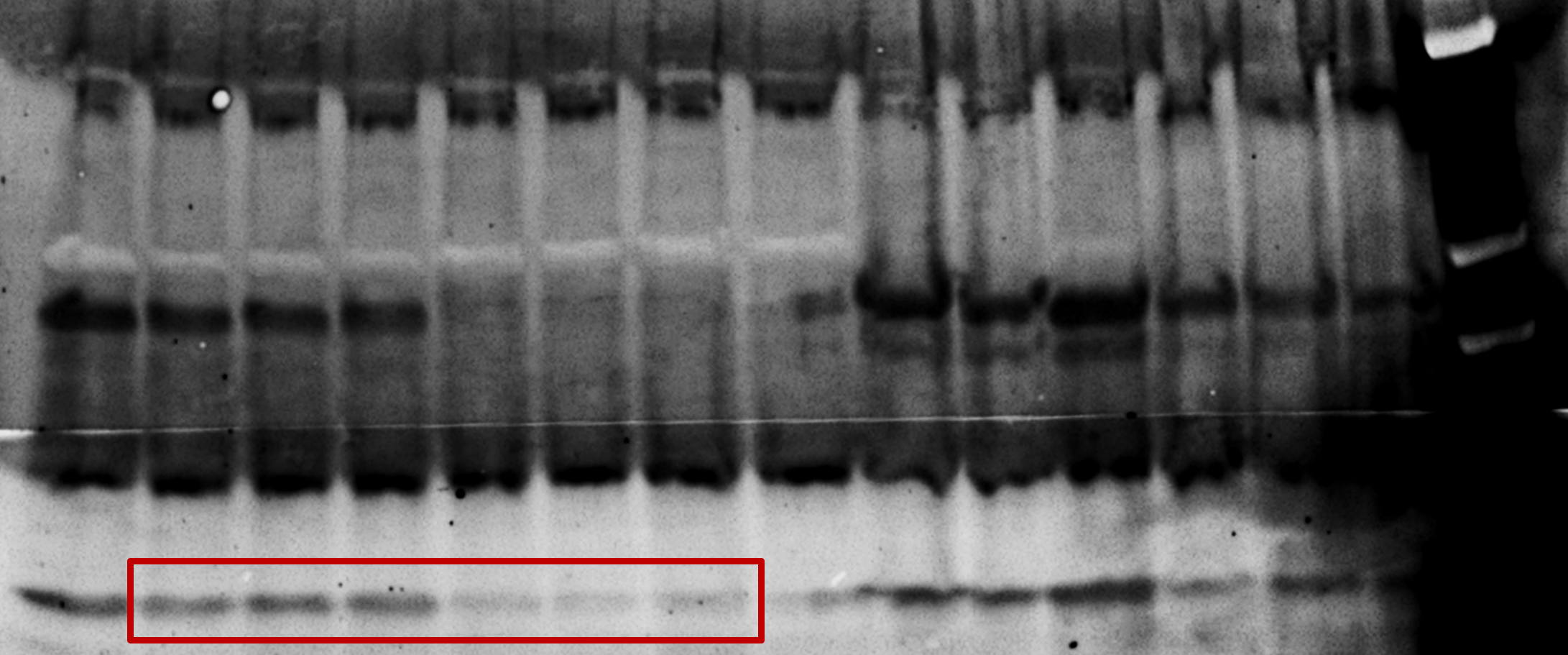

Supplement: Supplementary file 10 — Source data Fig. 3 [file 44321_2026_411_MOESM10_ESM.zip › Figure 3/3B/western CRYAB 1.tif]

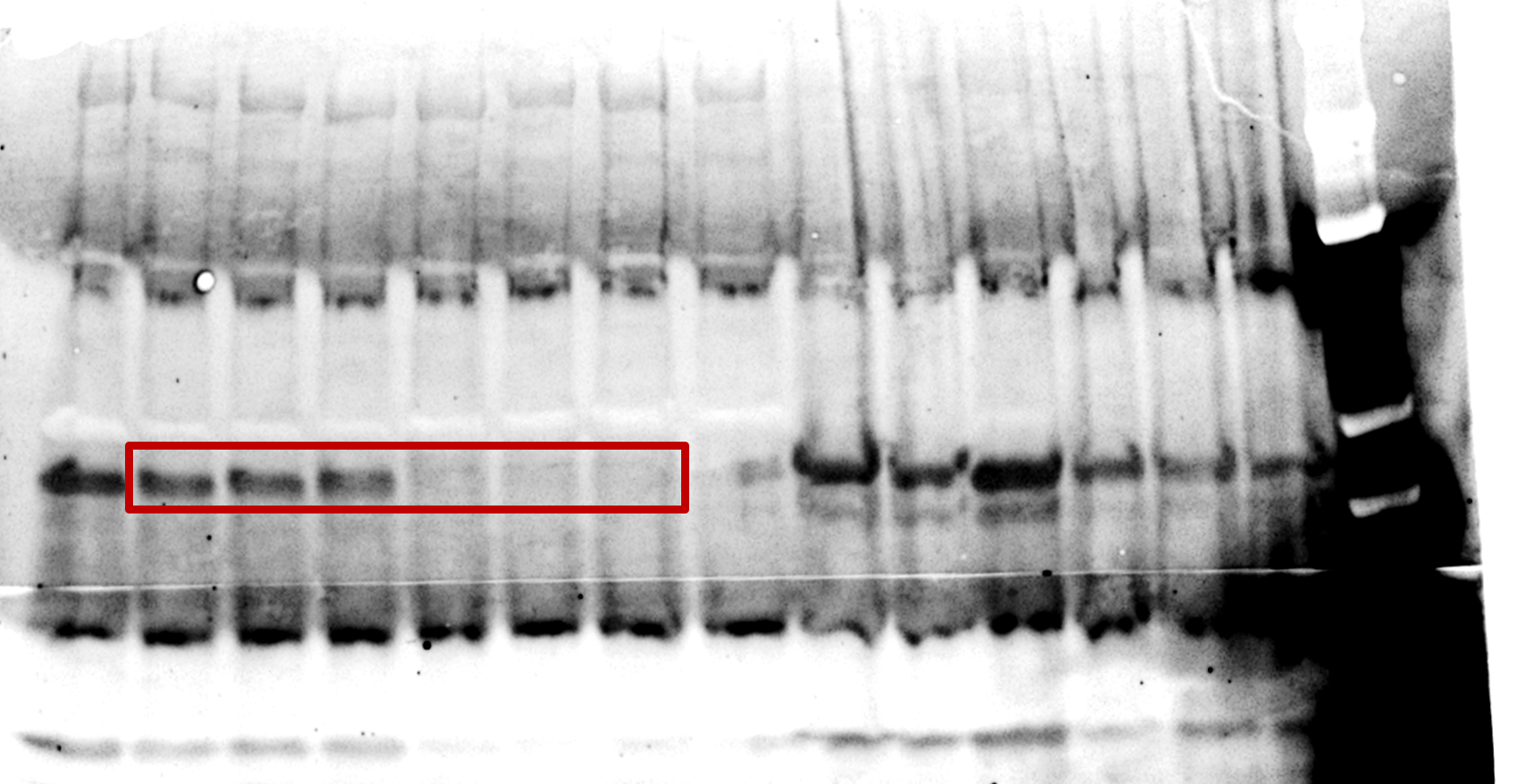

Supplement: Supplementary file 10 — Source data Fig. 3 [file 44321_2026_411_MOESM10_ESM.zip › Figure 3/3B/western CRYAB-GFP 1.png]

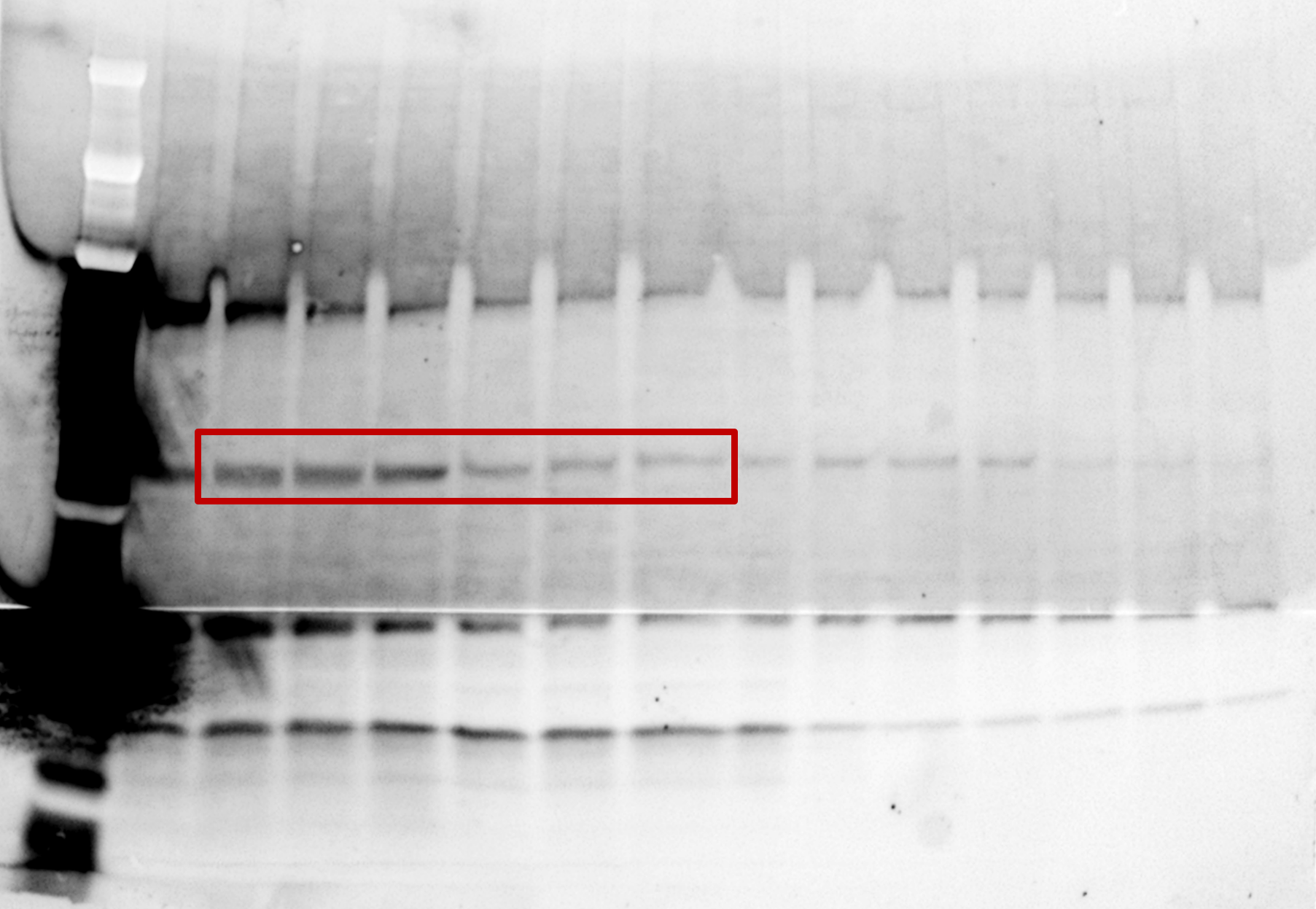

Supplement: Supplementary file 10 — Source data Fig. 3 [file 44321_2026_411_MOESM10_ESM.zip › Figure 3/3B/western CRYAB-GFP.tif]

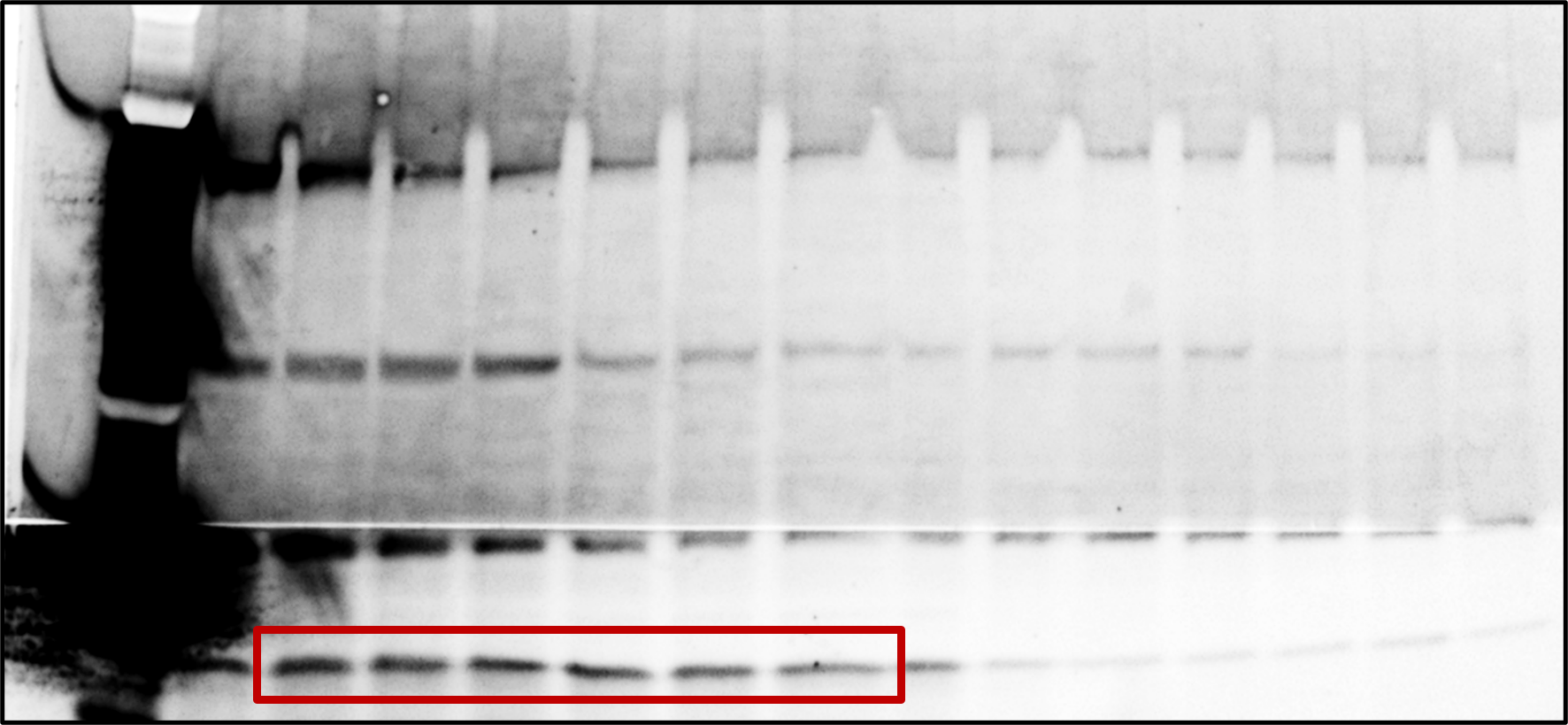

Supplement: Supplementary file 10 — Source data Fig. 3 [file 44321_2026_411_MOESM10_ESM.zip › Figure 3/3B/western CRYAB.tif]

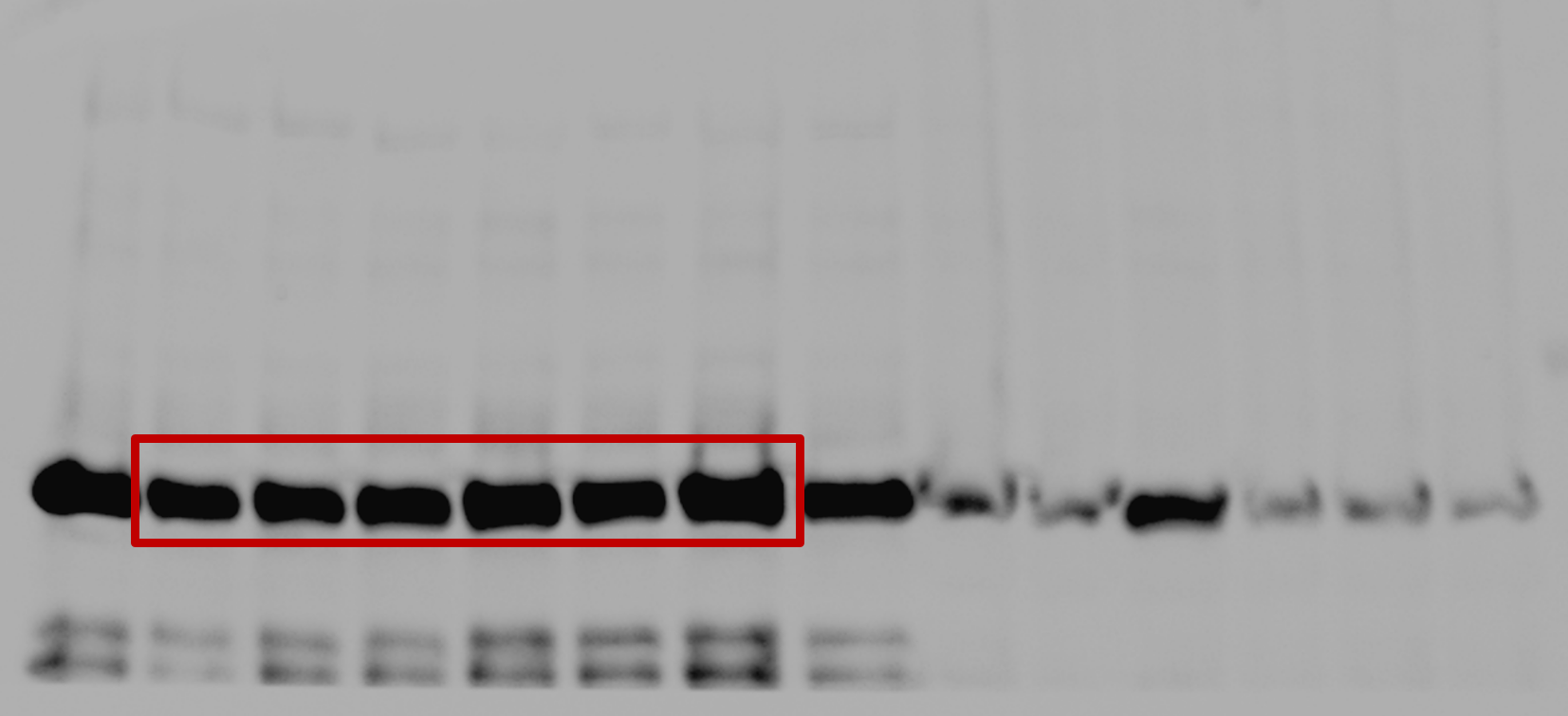

Supplement: Supplementary file 10 — Source data Fig. 3 [file 44321_2026_411_MOESM10_ESM.zip › Figure 3/3B/western DES 1.tif]

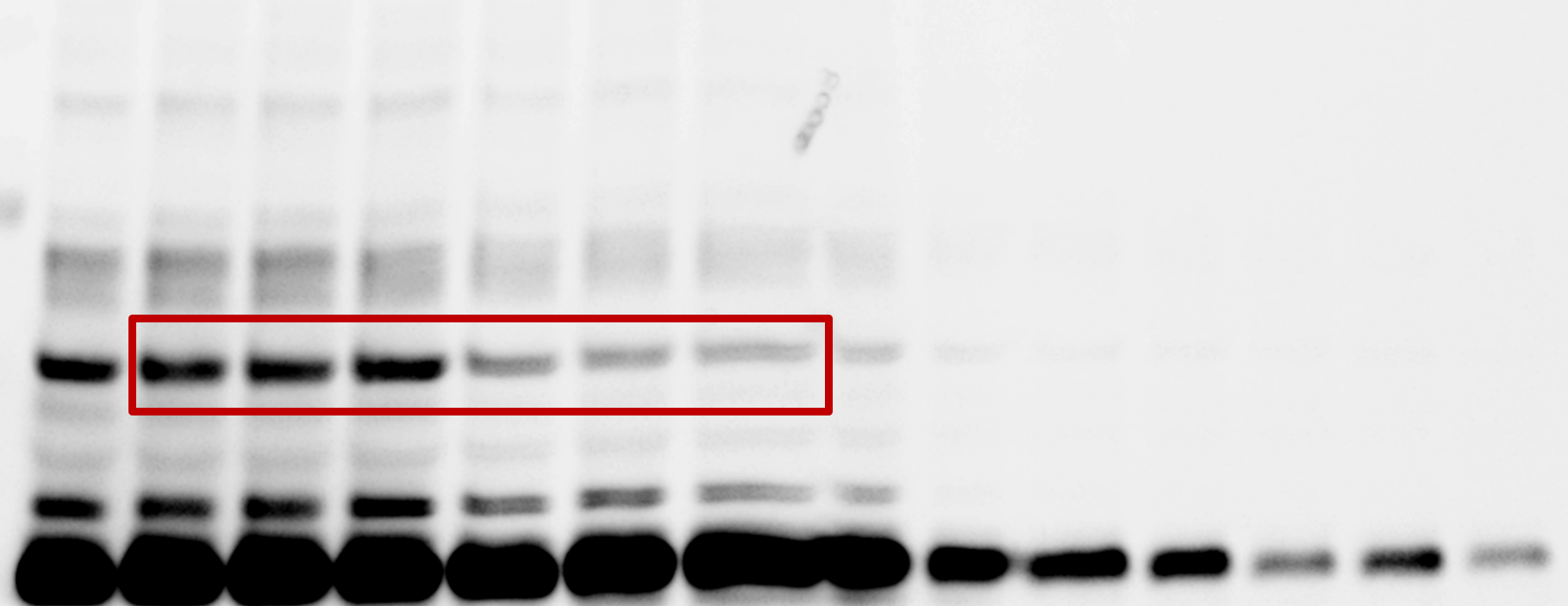

Supplement: Supplementary file 10 — Source data Fig. 3 [file 44321_2026_411_MOESM10_ESM.zip › Figure 3/3B/western DES.tif]

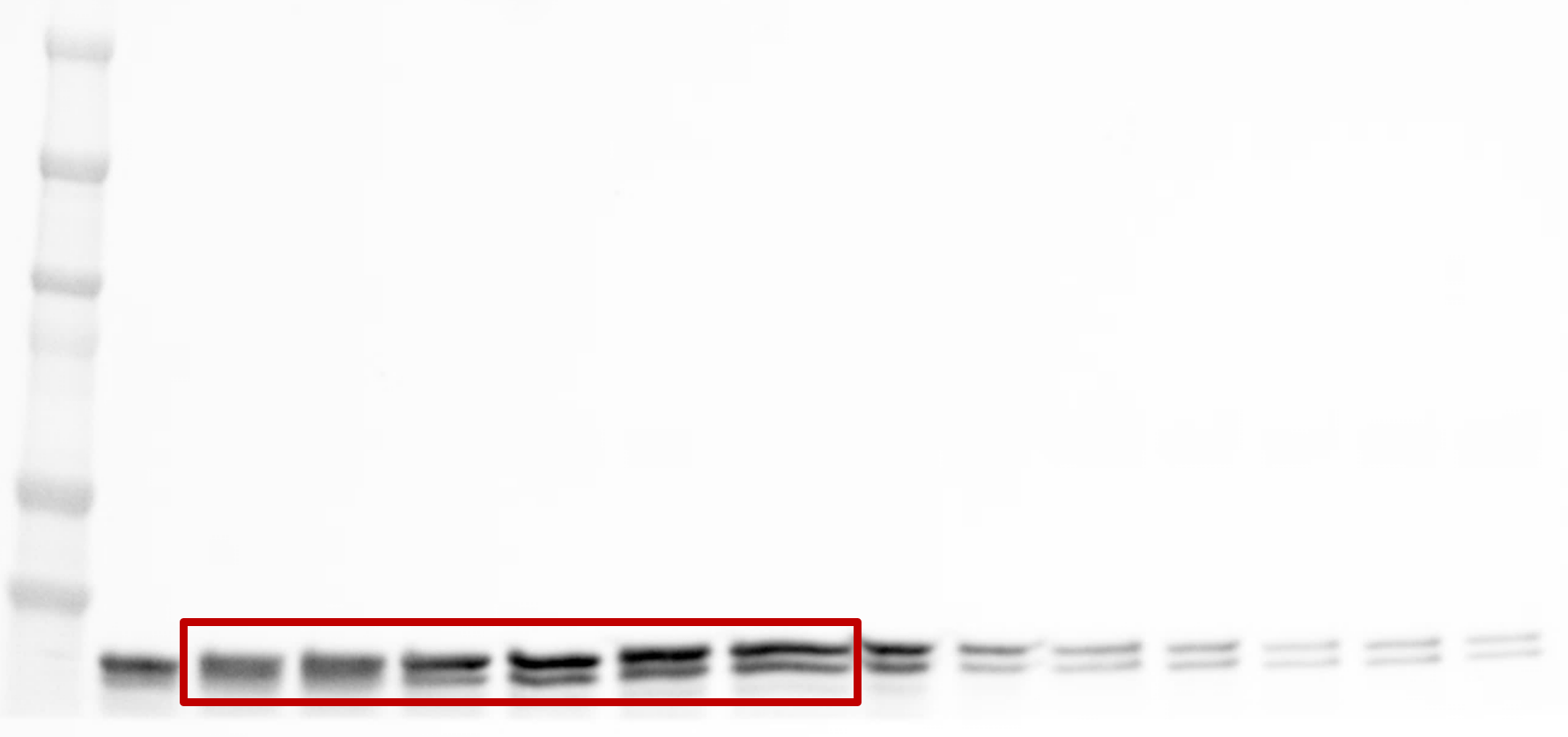

Supplement: Supplementary file 10 — Source data Fig. 3 [file 44321_2026_411_MOESM10_ESM.zip › Figure 3/3B/western GAPDH.tif]

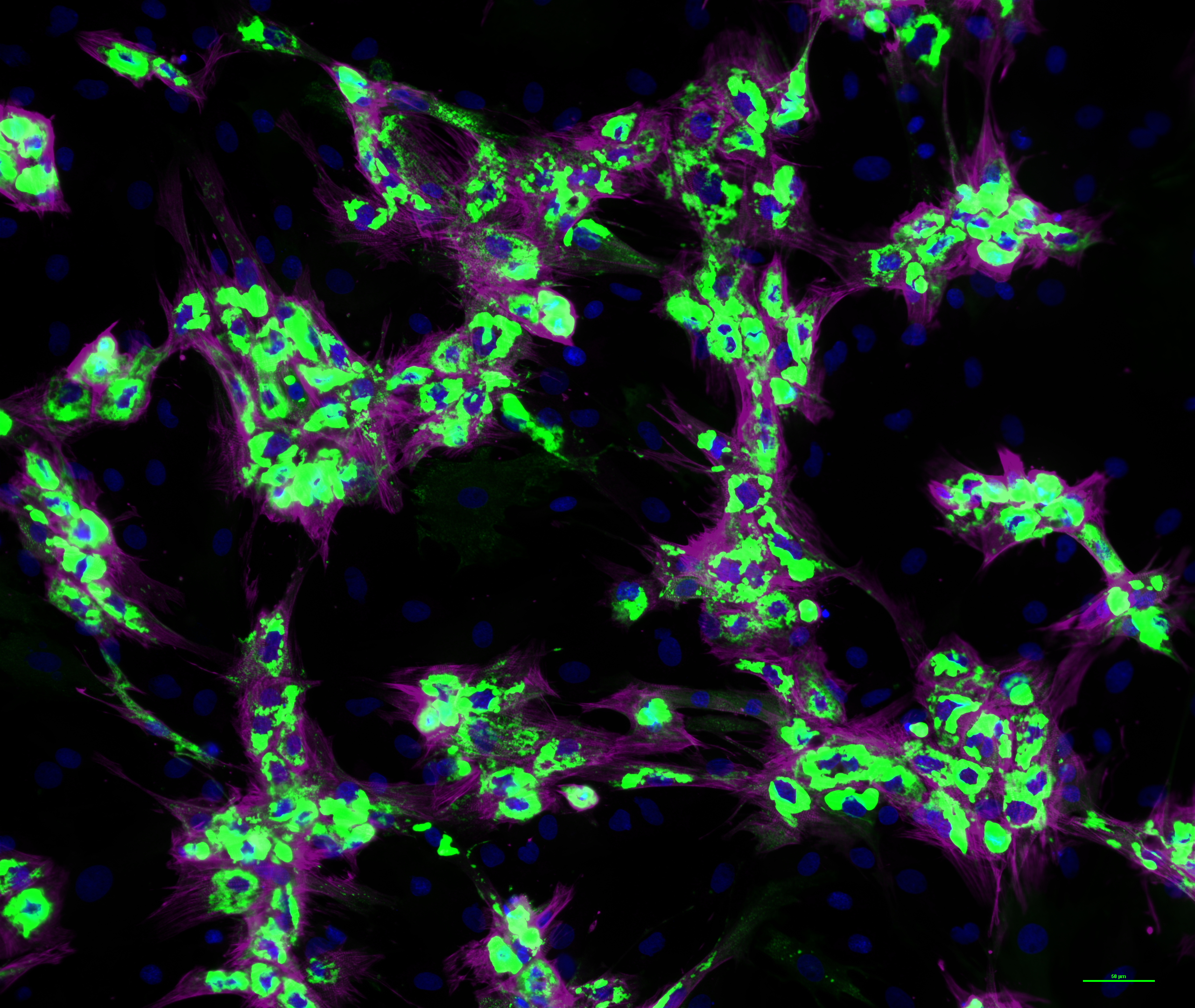

Supplement: Supplementary file 10 — Source data Fig. 3 [file 44321_2026_411_MOESM10_ESM.zip › Figure 3/3C/Scr_RGB.tif]

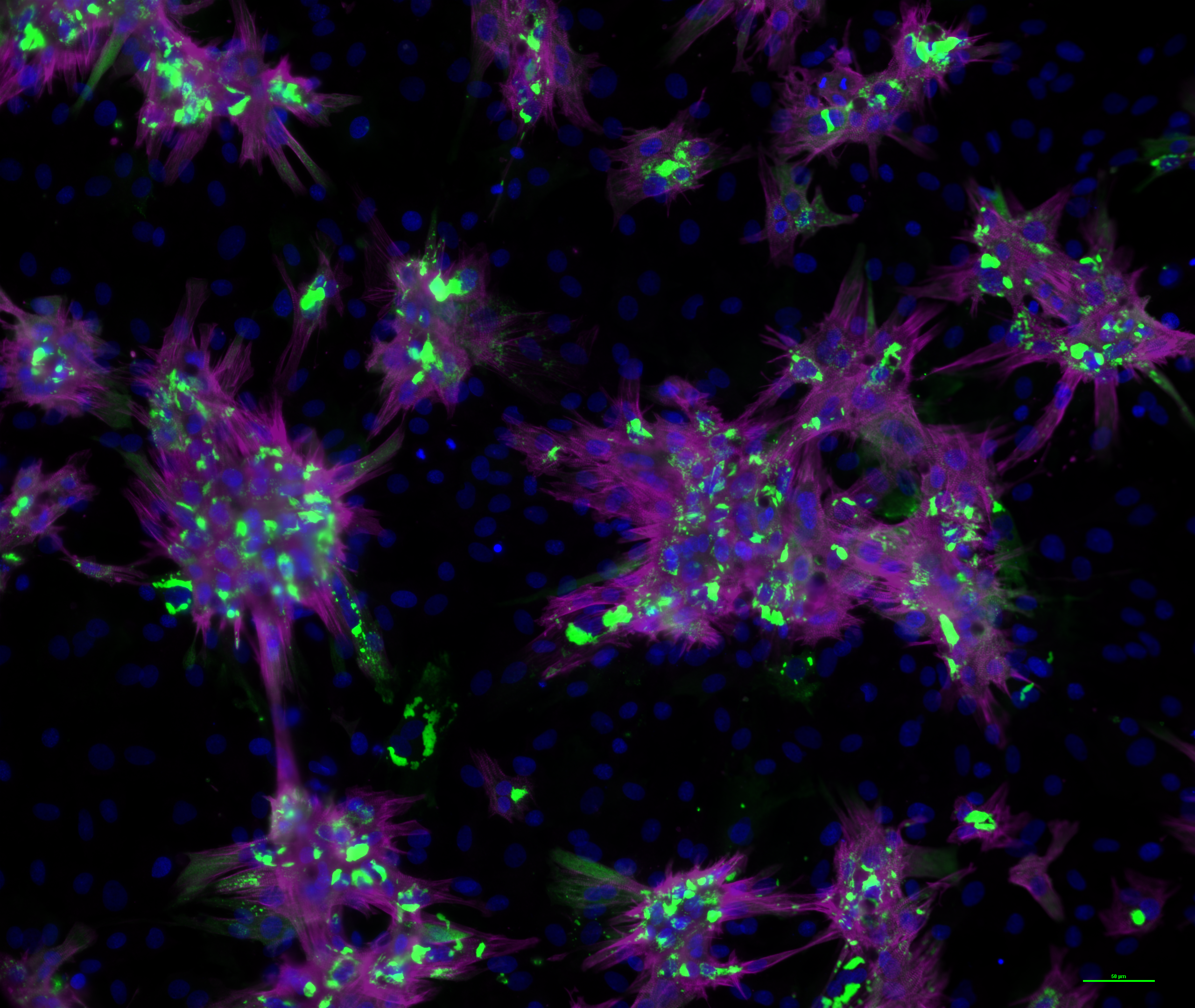

Supplement: Supplementary file 10 — Source data Fig. 3 [file 44321_2026_411_MOESM10_ESM.zip › Figure 3/3C/siJak1_RGB.tif]

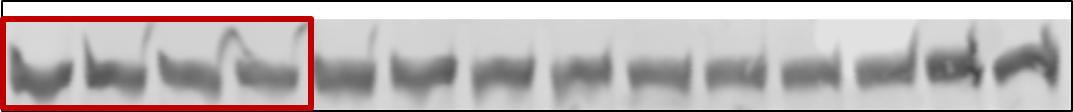

Supplement: Supplementary file 10 — Source data Fig. 3 [file 44321_2026_411_MOESM10_ESM.zip › Figure 3/3C/western ACTN2 2.tif]

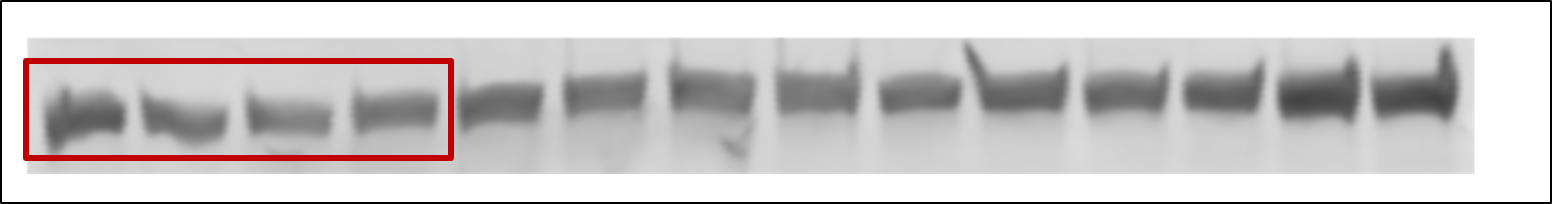

Supplement: Supplementary file 10 — Source data Fig. 3 [file 44321_2026_411_MOESM10_ESM.zip › Figure 3/3C/western ACTN2.tif]

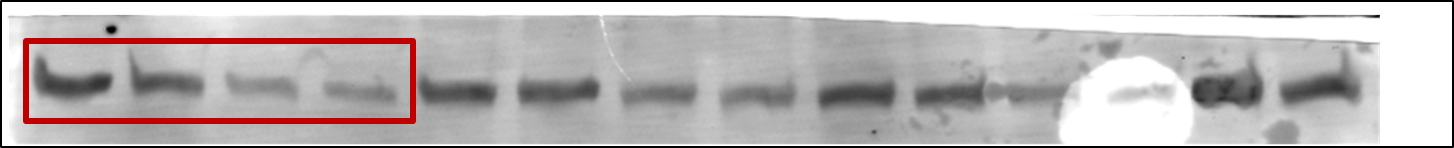

Supplement: Supplementary file 10 — Source data Fig. 3 [file 44321_2026_411_MOESM10_ESM.zip › Figure 3/3C/western JAK1 2.tif]

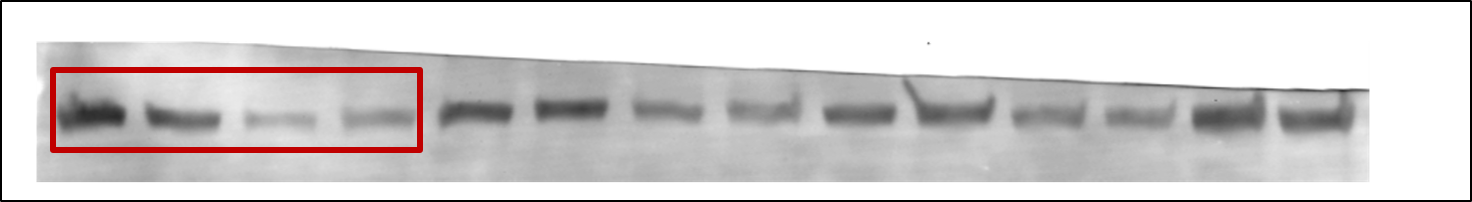

Supplement: Supplementary file 10 — Source data Fig. 3 [file 44321_2026_411_MOESM10_ESM.zip › Figure 3/3C/western JAK1.tif]

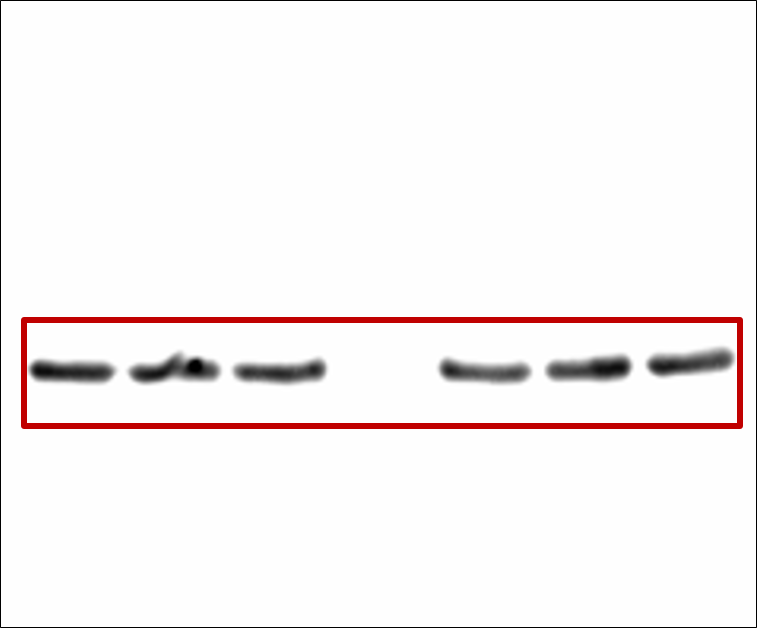

Supplement: Supplementary file 10 — Source data Fig. 3 [file 44321_2026_411_MOESM10_ESM.zip › Figure 3/3D/GAPDH.tif]

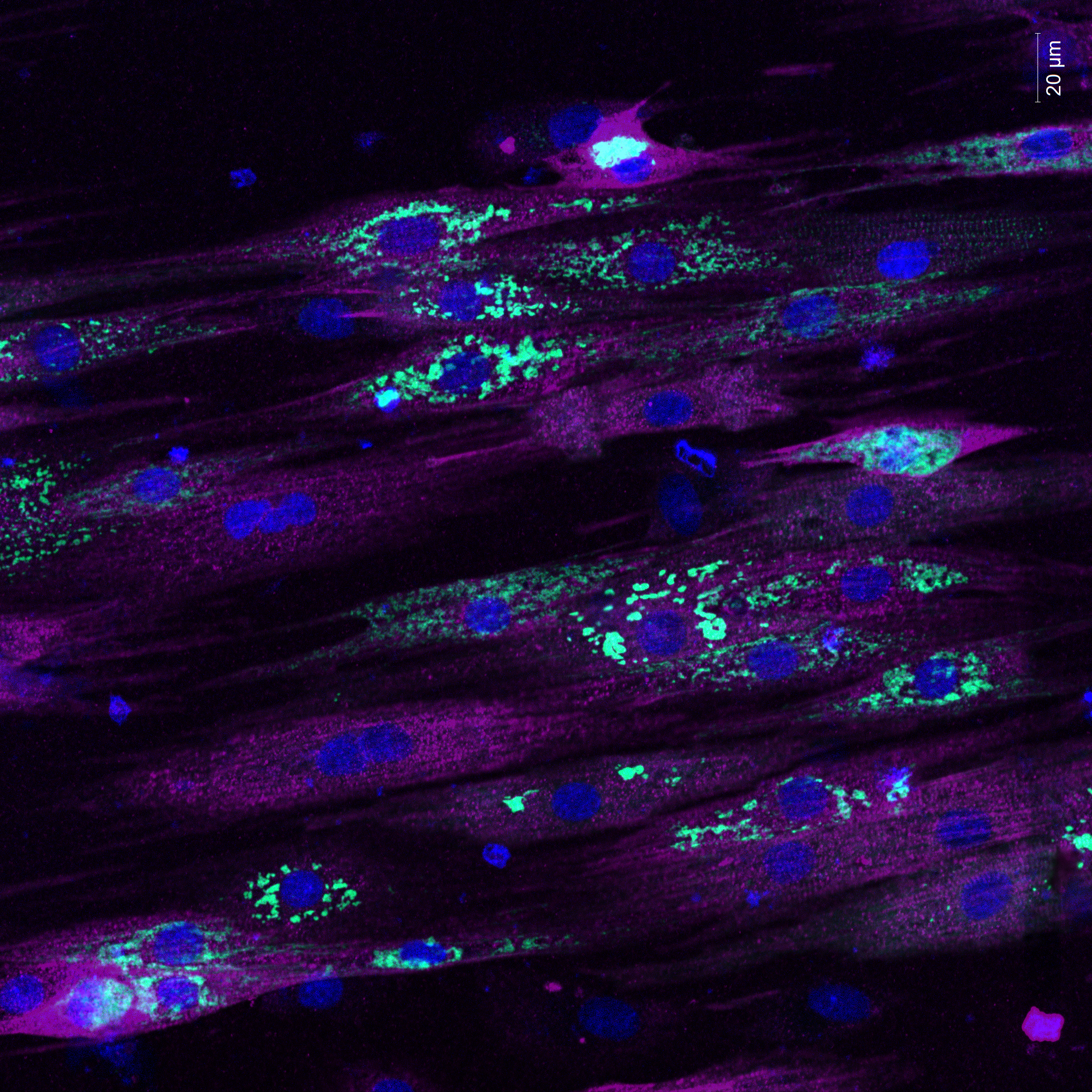

Supplement: Supplementary file 10 — Source data Fig. 3 [file 44321_2026_411_MOESM10_ESM.zip › Figure 3/3D/Scr_RGB.tif]

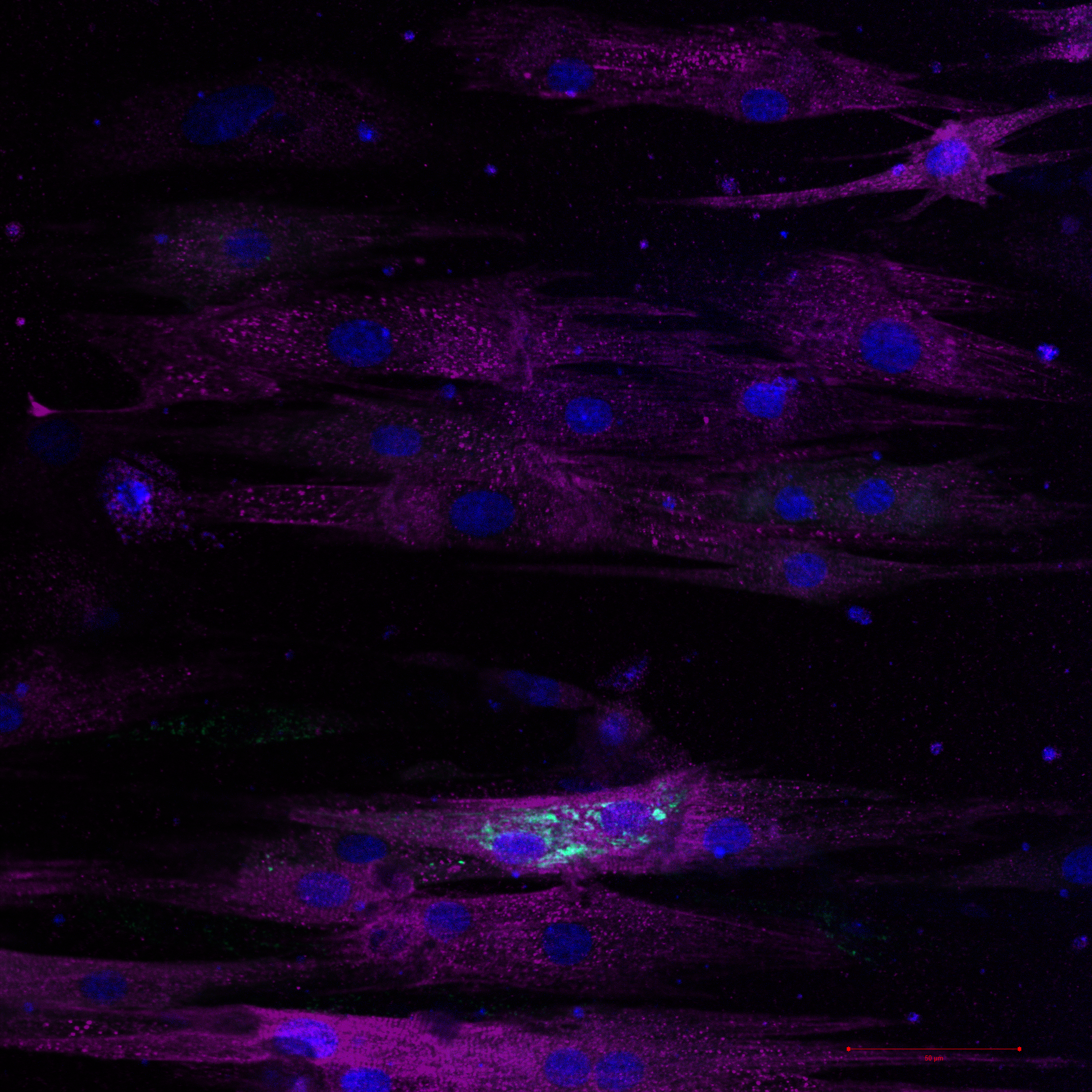

Supplement: Supplementary file 10 — Source data Fig. 3 [file 44321_2026_411_MOESM10_ESM.zip › Figure 3/3D/siStat3_RGB.tif]

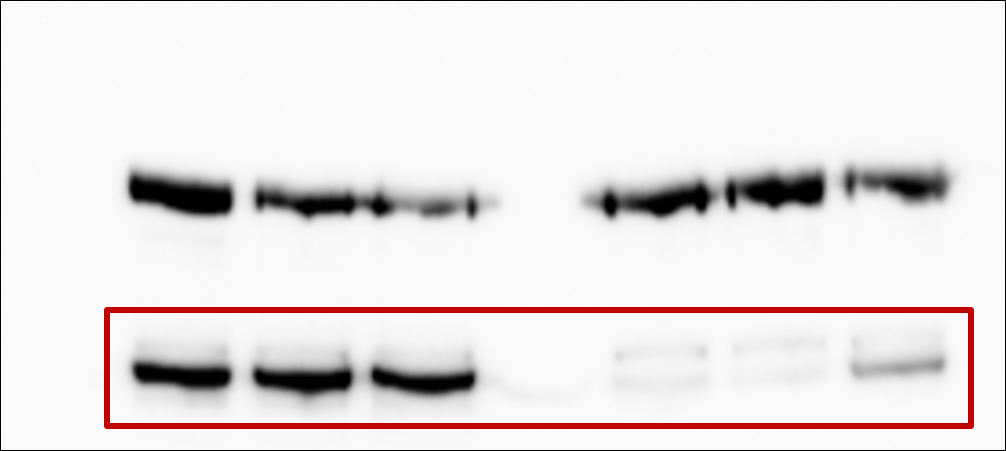

Supplement: Supplementary file 10 — Source data Fig. 3 [file 44321_2026_411_MOESM10_ESM.zip › Figure 3/3D/STAT3.tif]

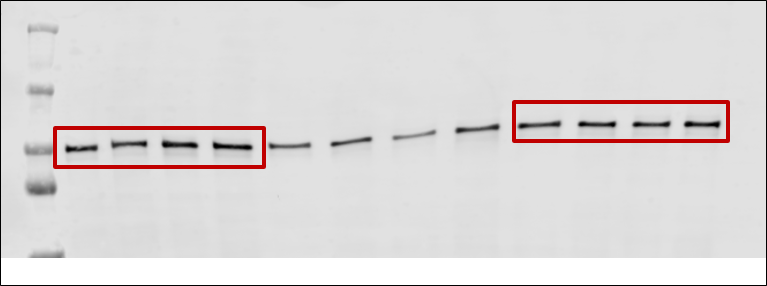

Supplement: Supplementary file 11 — Source data Fig. 4 [file 44321_2026_411_MOESM11_ESM.zip › Figure 4/4A/western ACTN2 a-actinin2.png]

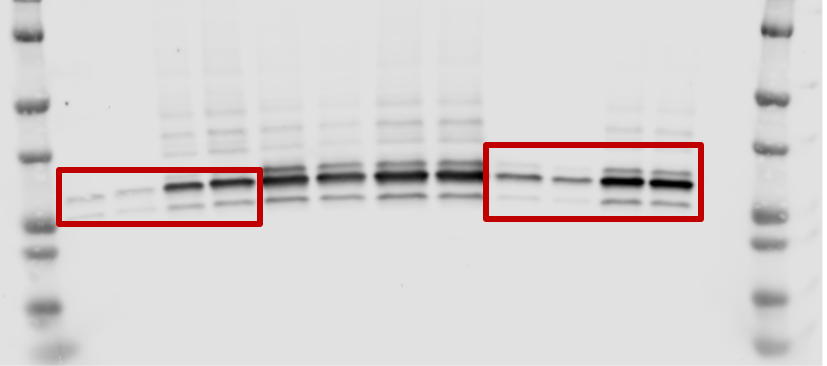

Supplement: Supplementary file 11 — Source data Fig. 4 [file 44321_2026_411_MOESM11_ESM.zip › Figure 4/4A/western GFPu .tif]

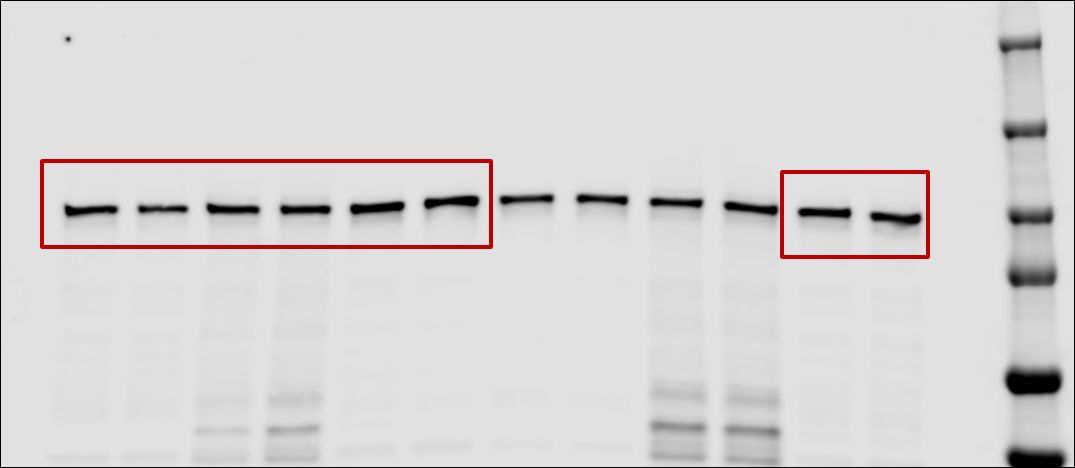

Supplement: Supplementary file 11 — Source data Fig. 4 [file 44321_2026_411_MOESM11_ESM.zip › Figure 4/4B/western ACTN2.tif]

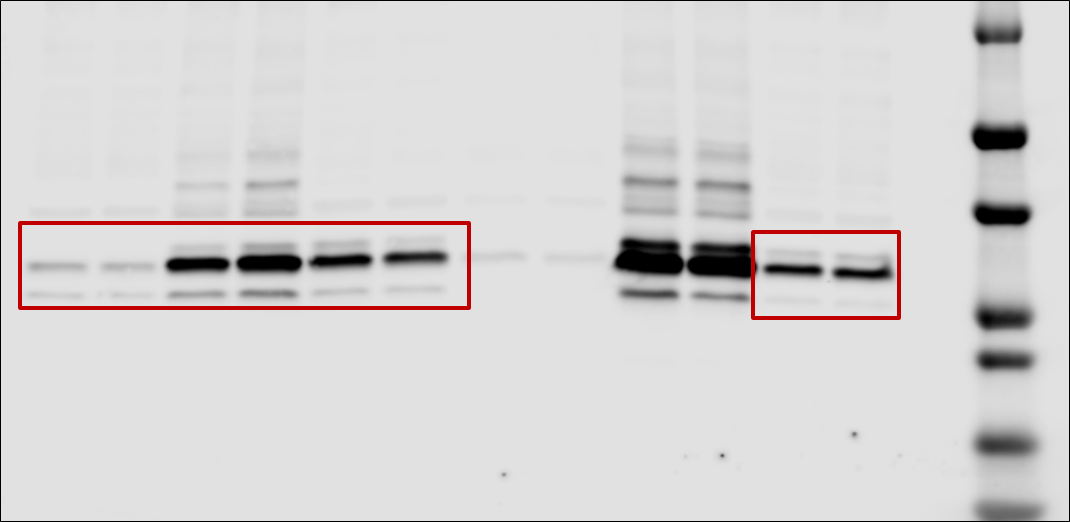

Supplement: Supplementary file 11 — Source data Fig. 4 [file 44321_2026_411_MOESM11_ESM.zip › Figure 4/4B/western GFPu.tif]

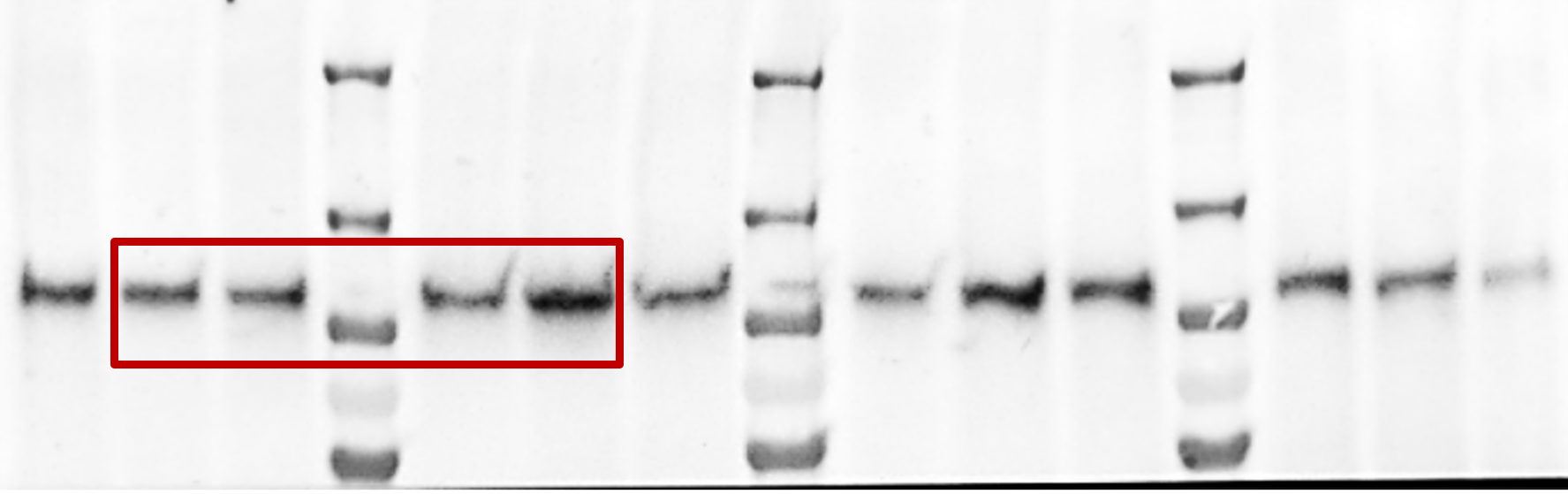

Supplement: Supplementary file 11 — Source data Fig. 4 [file 44321_2026_411_MOESM11_ESM.zip › Figure 4/4C/western ACTN2.png]

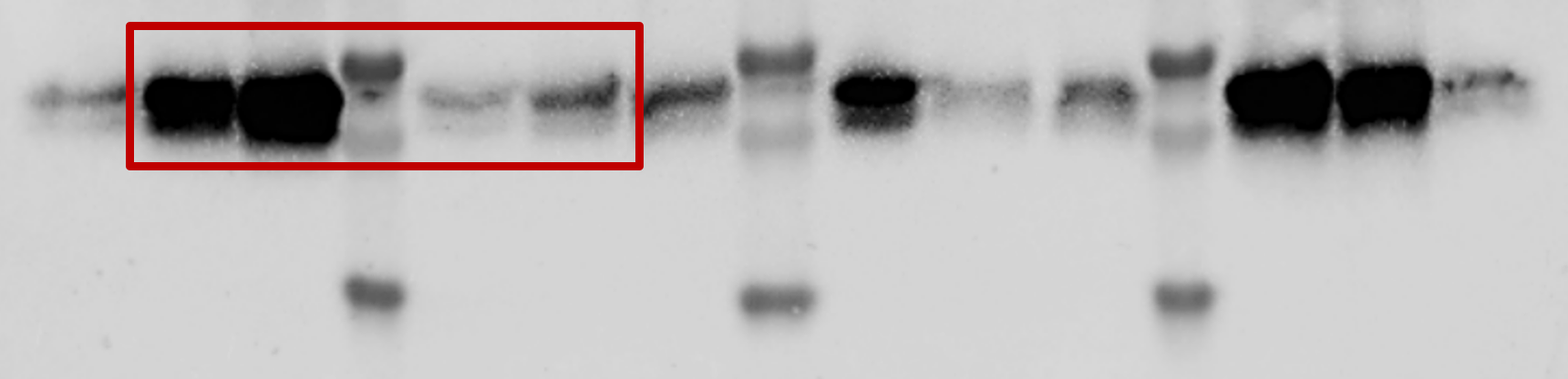

Supplement: Supplementary file 11 — Source data Fig. 4 [file 44321_2026_411_MOESM11_ESM.zip › Figure 4/4C/western GFPu.tif]

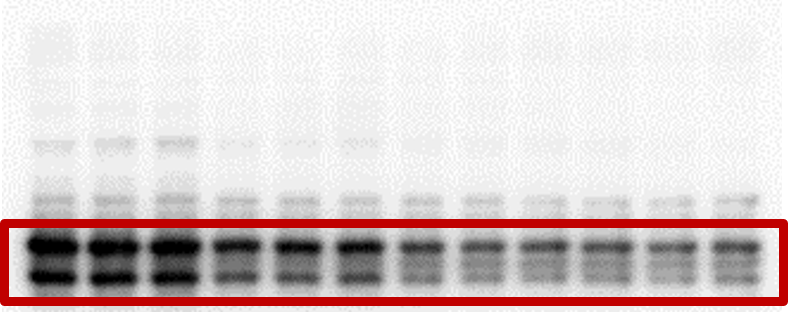

Supplement: Supplementary file 11 — Source data Fig. 4 [file 44321_2026_411_MOESM11_ESM.zip › Figure 4/4D/western GFPu.tif]

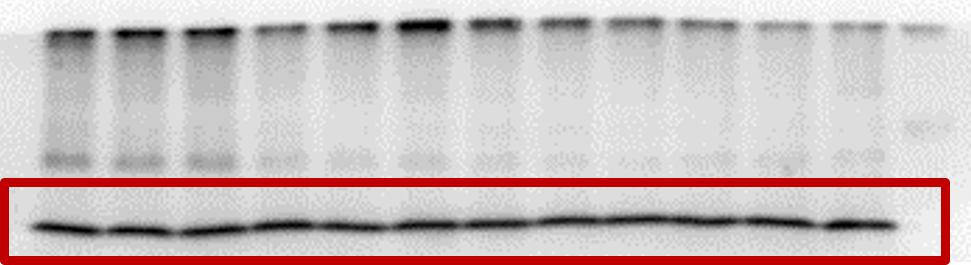

Supplement: Supplementary file 11 — Source data Fig. 4 [file 44321_2026_411_MOESM11_ESM.zip › Figure 4/4D/western H3.tif]

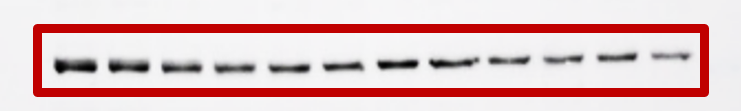

Supplement: Supplementary file 11 — Source data Fig. 4 [file 44321_2026_411_MOESM11_ESM.zip › Figure 4/4E/western a-tubulin.tif]

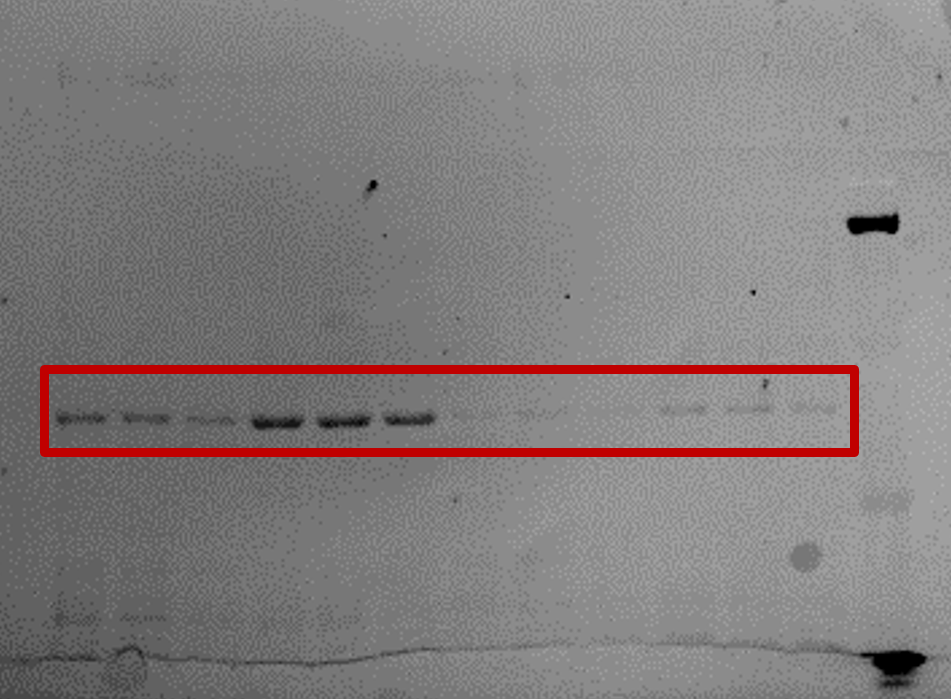

Supplement: Supplementary file 11 — Source data Fig. 4 [file 44321_2026_411_MOESM11_ESM.zip › Figure 4/4E/western CRYAB-GFP.tif]

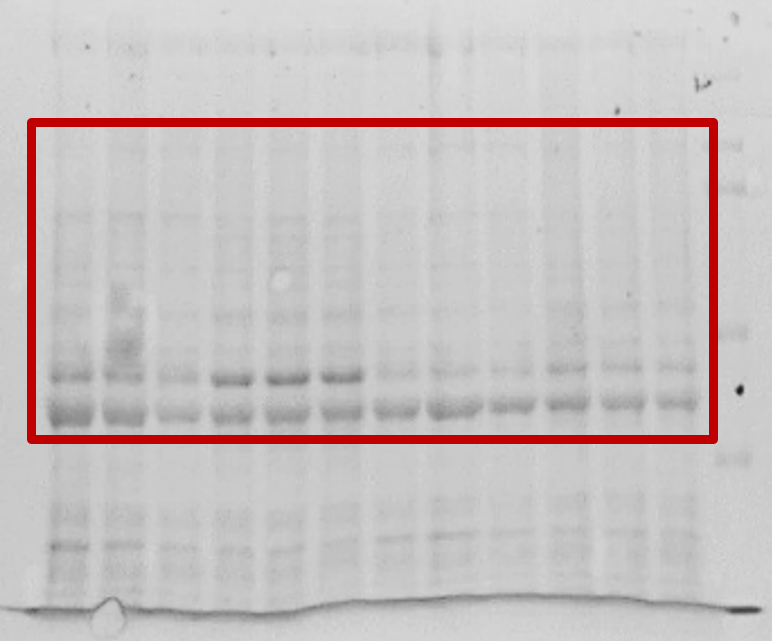

Supplement: Supplementary file 11 — Source data Fig. 4 [file 44321_2026_411_MOESM11_ESM.zip › Figure 4/4E/western Ponceau 1.tif]

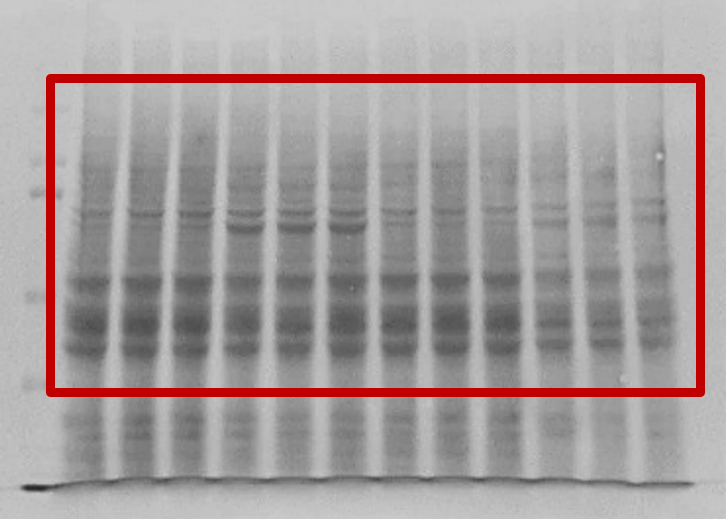

Supplement: Supplementary file 11 — Source data Fig. 4 [file 44321_2026_411_MOESM11_ESM.zip › Figure 4/4E/western Ponceau.tif]

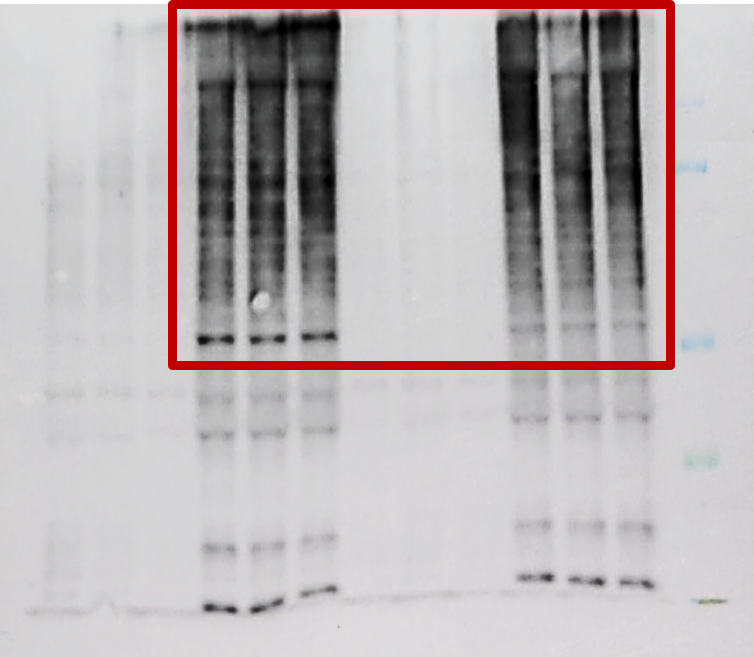

Supplement: Supplementary file 11 — Source data Fig. 4 [file 44321_2026_411_MOESM11_ESM.zip › Figure 4/4E/western Ubiquitin 1.tif]
