# Supplementary figures and images for: JAK1/2 inhibitor ruxolitinib reduces aggregates in cardiac proteinopathy (part 2 of 3)
Source: EMBO Mol Med. 2026 Mar 31;18(5):1836–65. doi: 10.1038/s44321-026-00411-x (PMC13179346; doi:10.1038/s44321-026-00411-x)

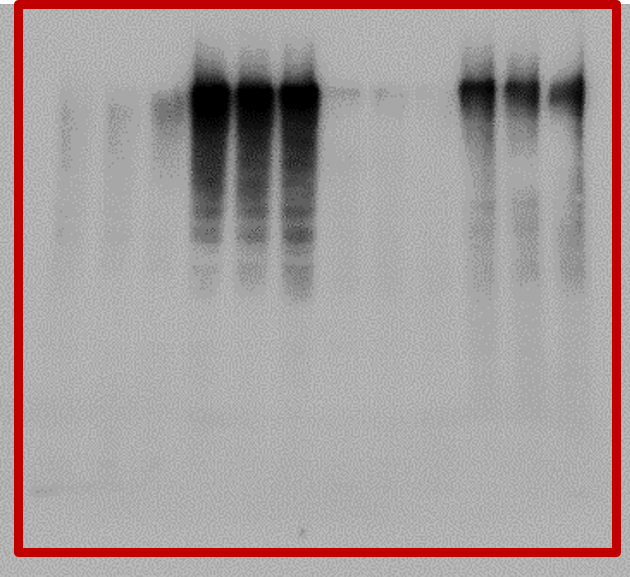

Supplement: Supplementary file 11 — Source data Fig. 4 [file 44321_2026_411_MOESM11_ESM.zip › Figure 4/4E/western Ubiquitin.tif]

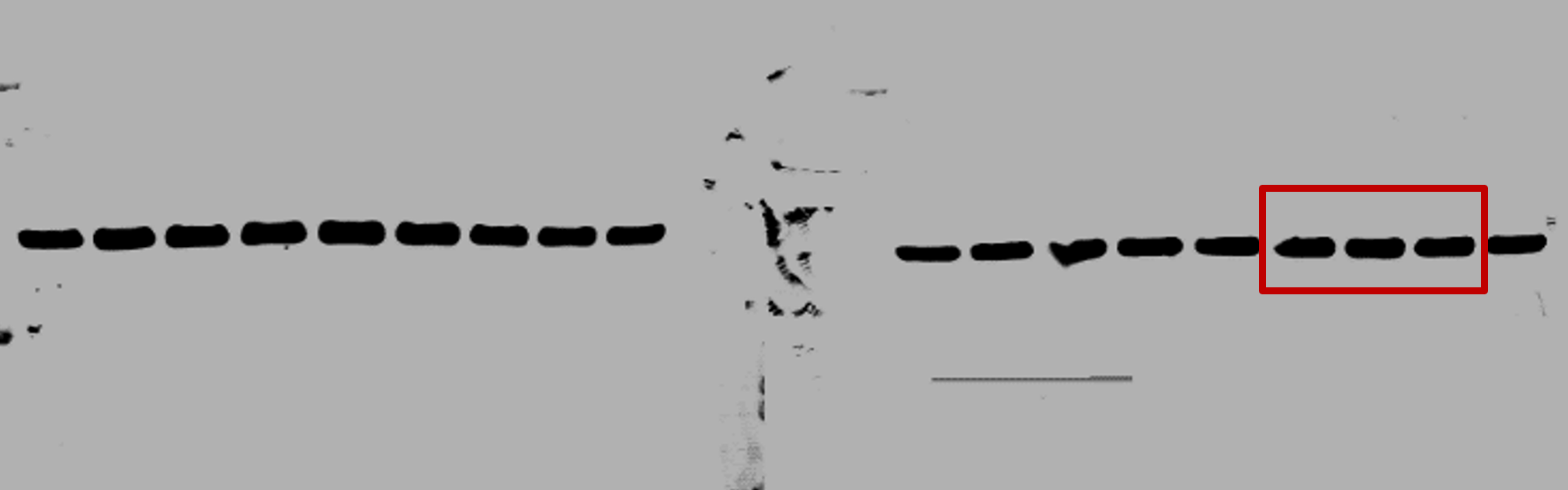

Supplement: Supplementary file 12 — Source data Fig. 5 [file 44321_2026_411_MOESM12_ESM.zip › Figure 5/5A/western GAPDH 1.tif]

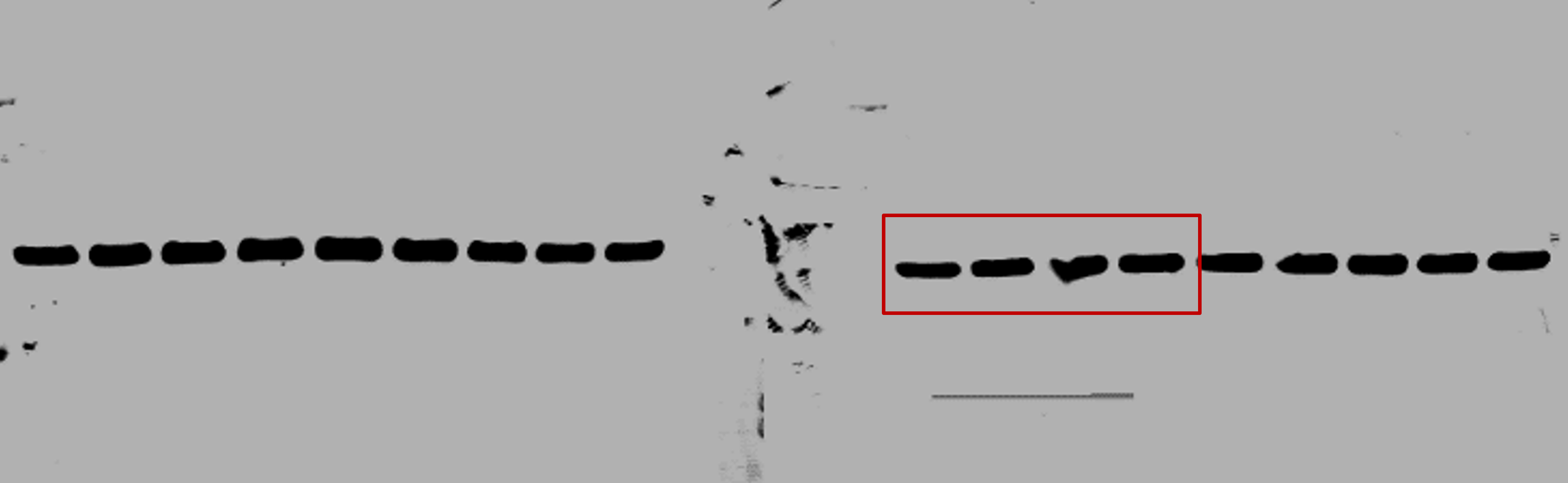

Supplement: Supplementary file 12 — Source data Fig. 5 [file 44321_2026_411_MOESM12_ESM.zip › Figure 5/5A/western GAPDH.tif]

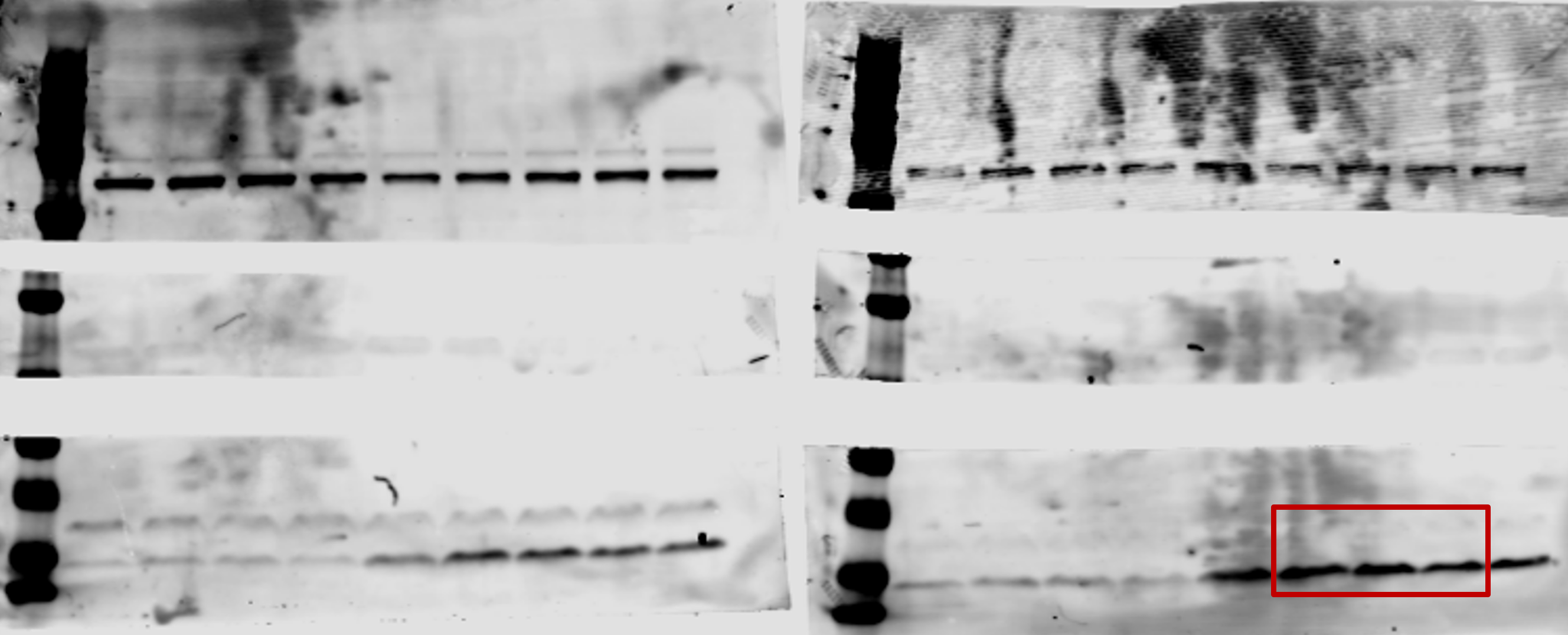

Supplement: Supplementary file 12 — Source data Fig. 5 [file 44321_2026_411_MOESM12_ESM.zip › Figure 5/5A/western LC3-I, LC3-II 1.tif]

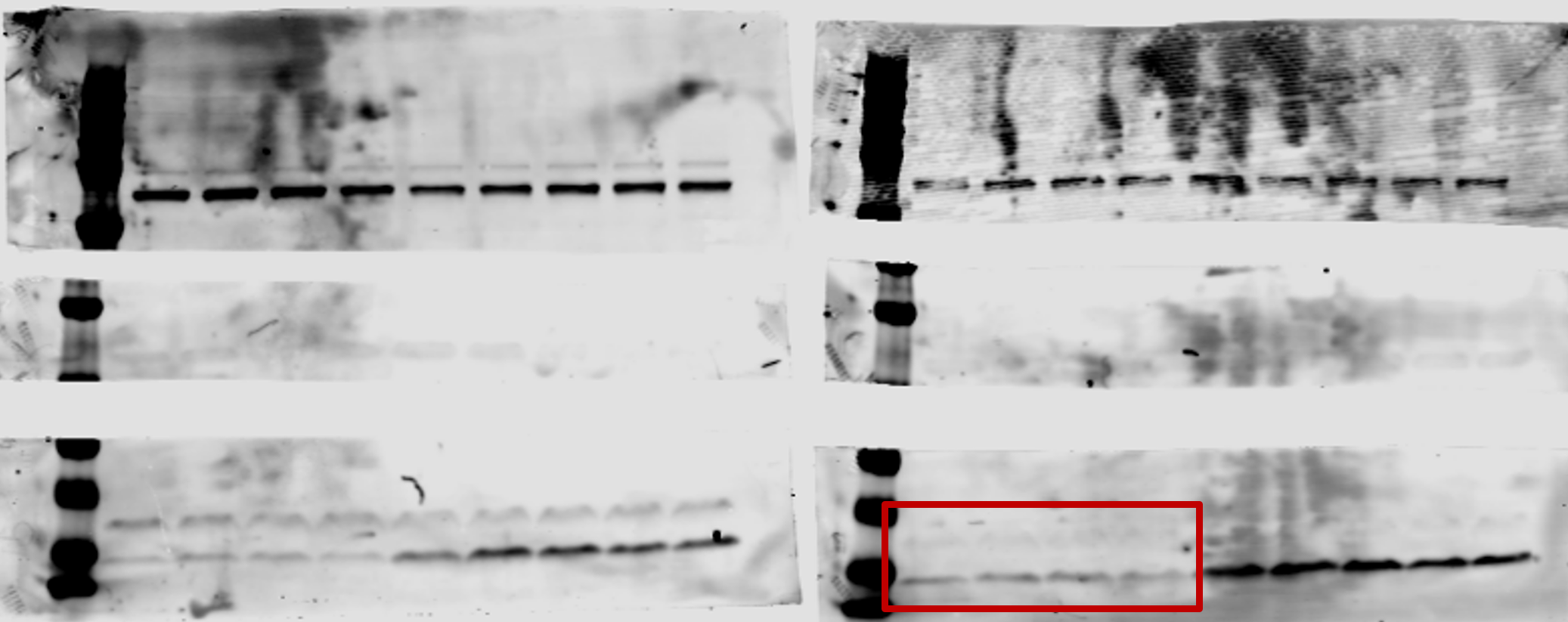

Supplement: Supplementary file 12 — Source data Fig. 5 [file 44321_2026_411_MOESM12_ESM.zip › Figure 5/5A/western LC3-I, LC3-II.tif]

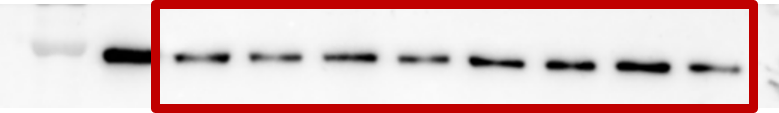

Supplement: Supplementary file 12 — Source data Fig. 5 [file 44321_2026_411_MOESM12_ESM.zip › Figure 5/5B/western GAPDH.tif]

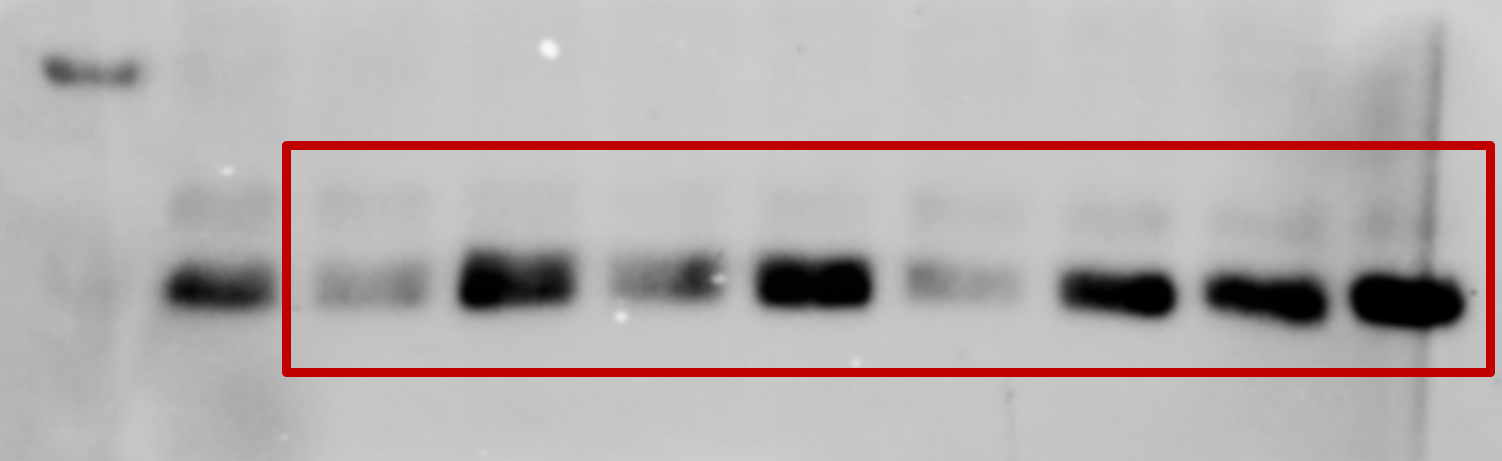

Supplement: Supplementary file 12 — Source data Fig. 5 [file 44321_2026_411_MOESM12_ESM.zip › Figure 5/5B/western LC3-I, LC3-II.tif]

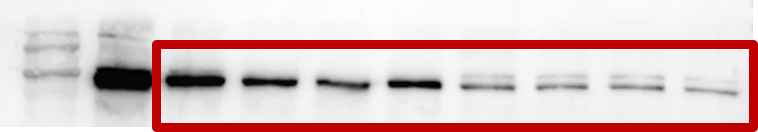

Supplement: Supplementary file 12 — Source data Fig. 5 [file 44321_2026_411_MOESM12_ESM.zip › Figure 5/5B/western STAT3.tif]

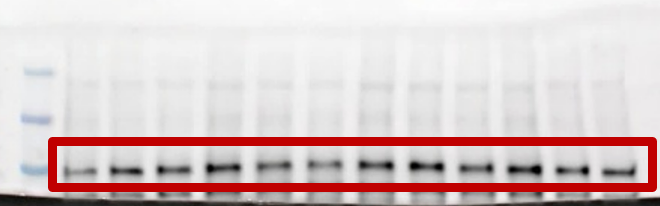

Supplement: Supplementary file 12 — Source data Fig. 5 [file 44321_2026_411_MOESM12_ESM.zip › Figure 5/5C/western ACTN2 1.tif]

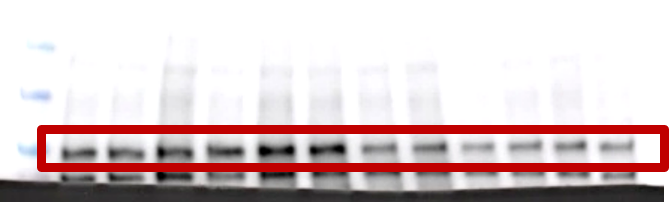

Supplement: Supplementary file 12 — Source data Fig. 5 [file 44321_2026_411_MOESM12_ESM.zip › Figure 5/5C/western ACTN2.tif]

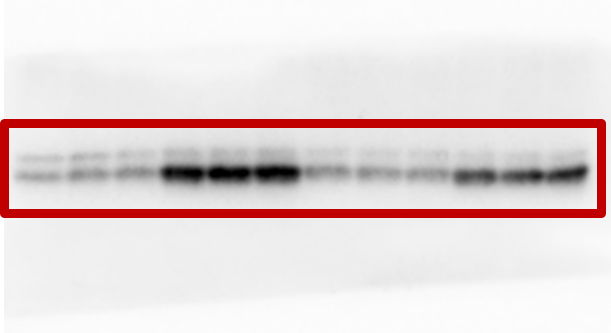

Supplement: Supplementary file 12 — Source data Fig. 5 [file 44321_2026_411_MOESM12_ESM.zip › Figure 5/5C/western LC3-I, LC3-II 1.tif]

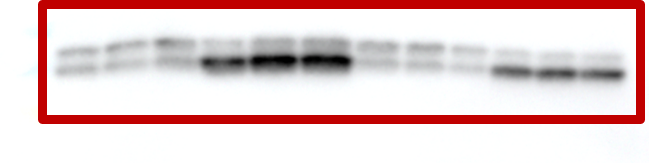

Supplement: Supplementary file 12 — Source data Fig. 5 [file 44321_2026_411_MOESM12_ESM.zip › Figure 5/5C/western LC3-I, LC3-II.tif]

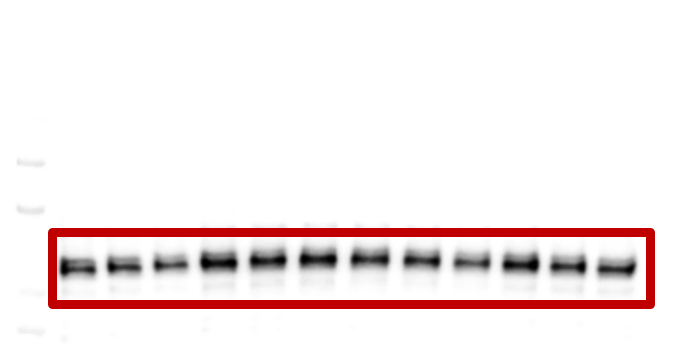

Supplement: Supplementary file 12 — Source data Fig. 5 [file 44321_2026_411_MOESM12_ESM.zip › Figure 5/5C/western p62 1.tif]

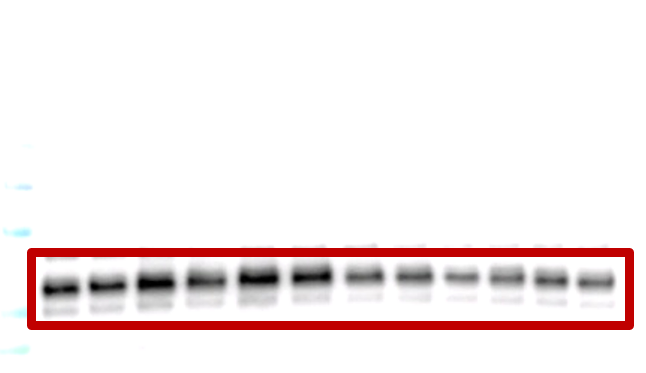

Supplement: Supplementary file 12 — Source data Fig. 5 [file 44321_2026_411_MOESM12_ESM.zip › Figure 5/5C/western p62.tif]

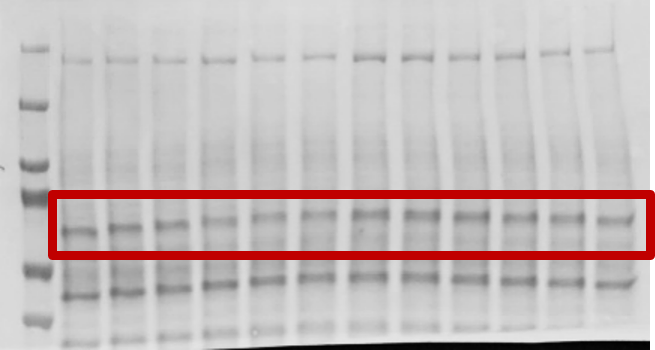

Supplement: Supplementary file 12 — Source data Fig. 5 [file 44321_2026_411_MOESM12_ESM.zip › Figure 5/5C/western Ponceau 1.tif]

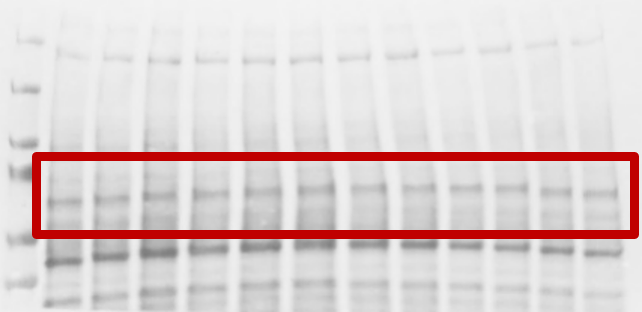

Supplement: Supplementary file 12 — Source data Fig. 5 [file 44321_2026_411_MOESM12_ESM.zip › Figure 5/5C/western Ponceau.tif]

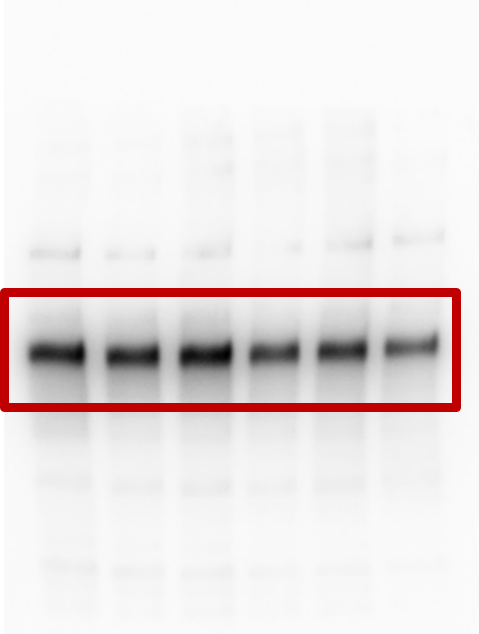

Supplement: Supplementary file 12 — Source data Fig. 5 [file 44321_2026_411_MOESM12_ESM.zip › Figure 5/5D/western BAG3.tif]

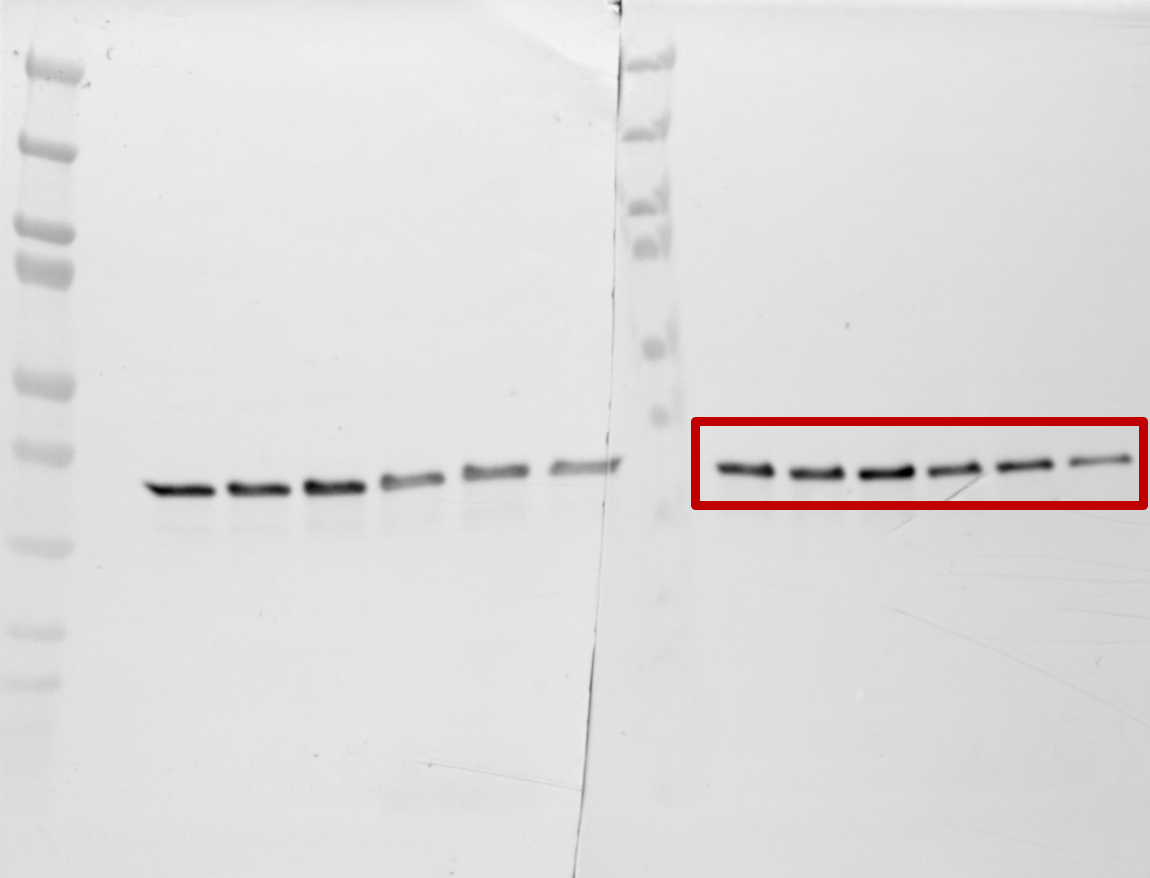

Supplement: Supplementary file 12 — Source data Fig. 5 [file 44321_2026_411_MOESM12_ESM.zip › Figure 5/5D/western GAPDH 1.tif]

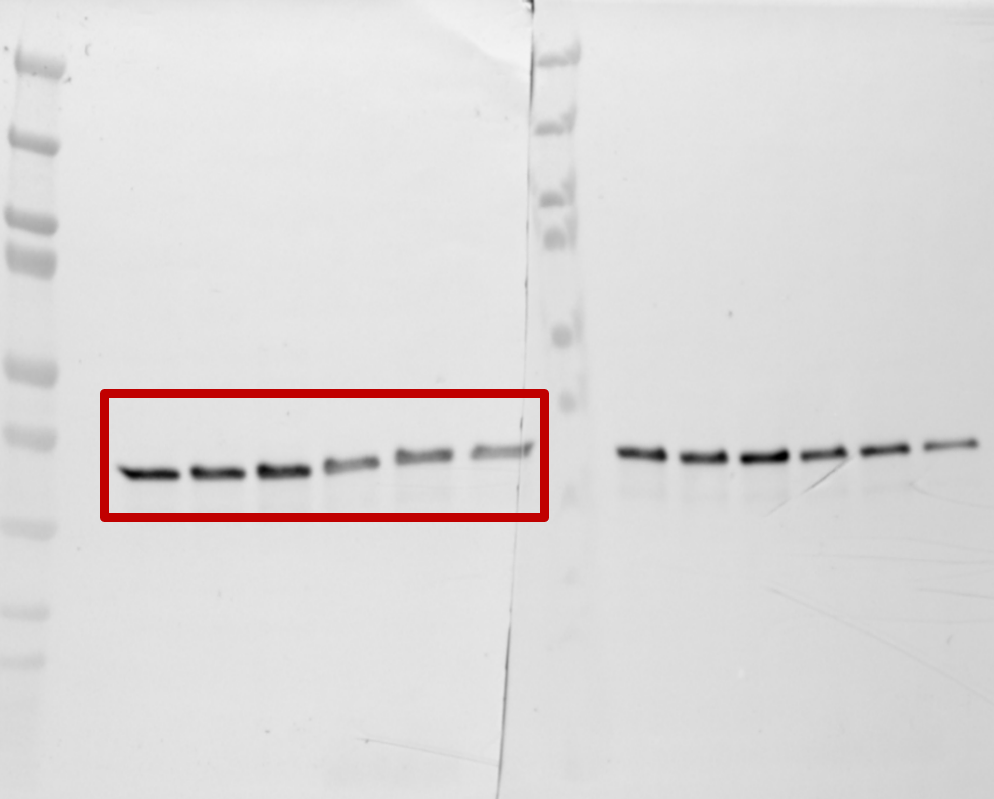

Supplement: Supplementary file 12 — Source data Fig. 5 [file 44321_2026_411_MOESM12_ESM.zip › Figure 5/5D/western GAPDH.tif]

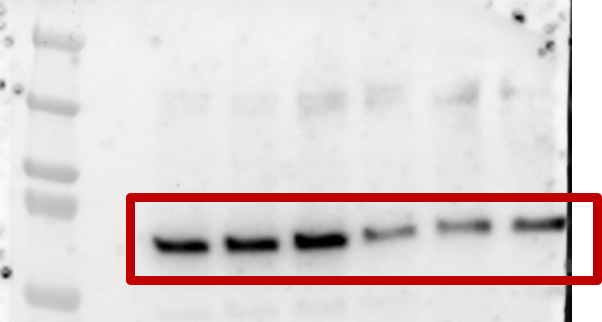

Supplement: Supplementary file 12 — Source data Fig. 5 [file 44321_2026_411_MOESM12_ESM.zip › Figure 5/5D/western HSC70.tif]

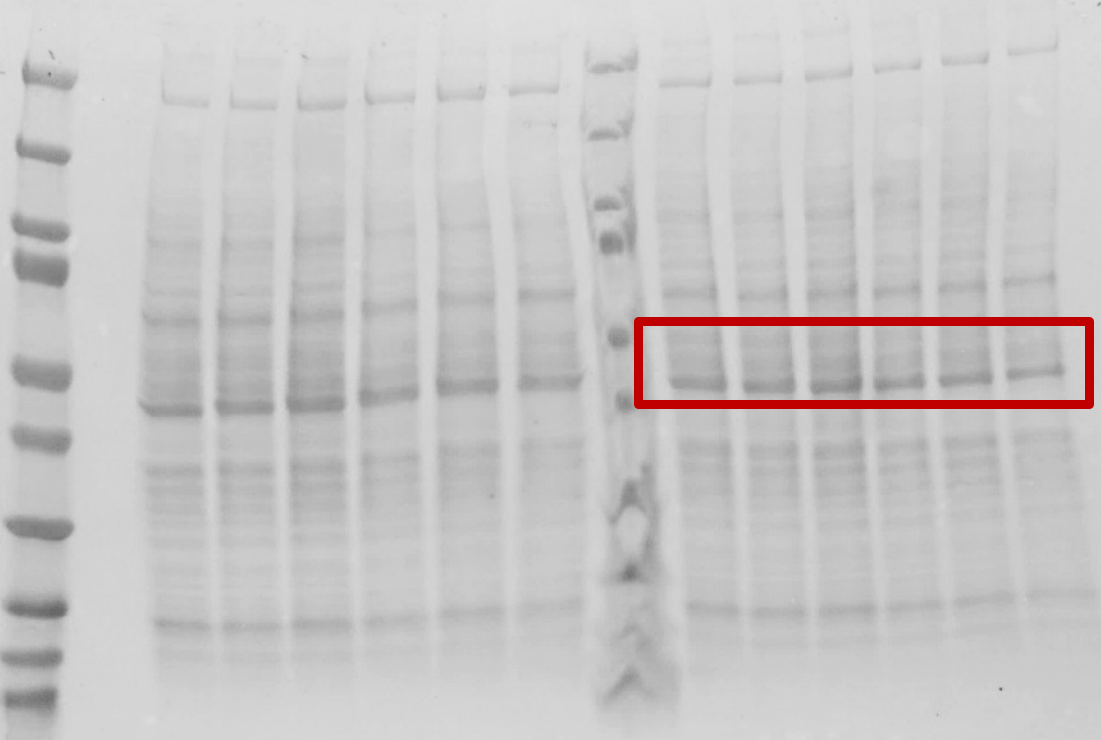

Supplement: Supplementary file 12 — Source data Fig. 5 [file 44321_2026_411_MOESM12_ESM.zip › Figure 5/5D/western Ponceau 1.tif]

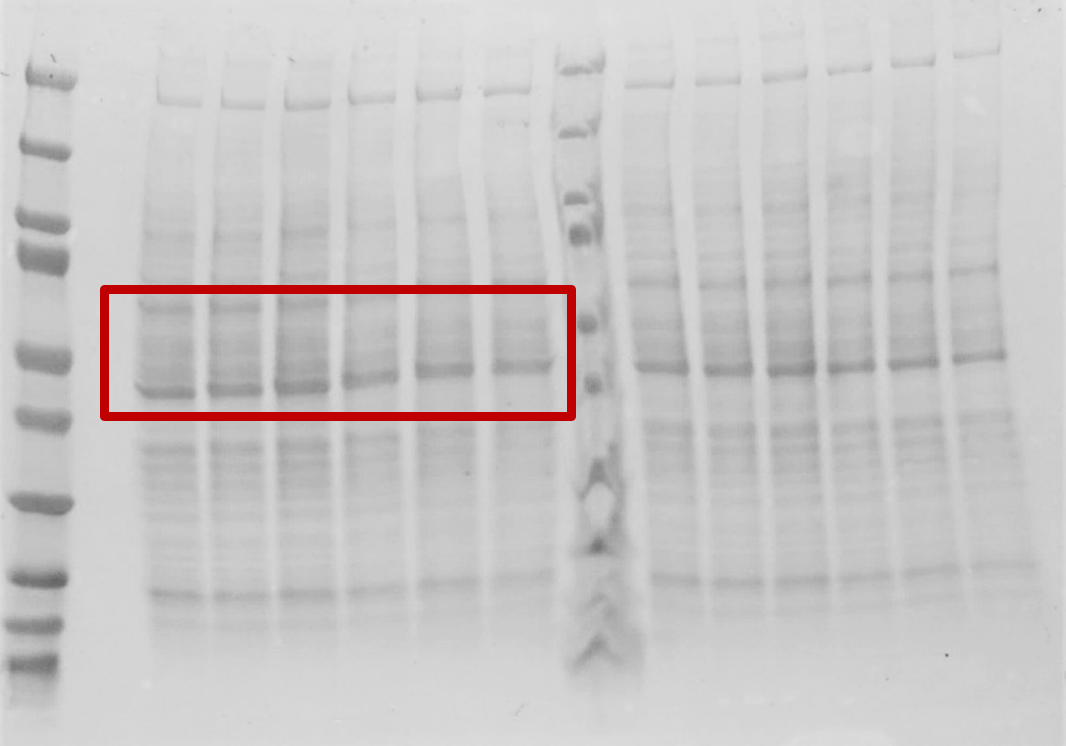

Supplement: Supplementary file 12 — Source data Fig. 5 [file 44321_2026_411_MOESM12_ESM.zip › Figure 5/5D/western Ponceau.tif]

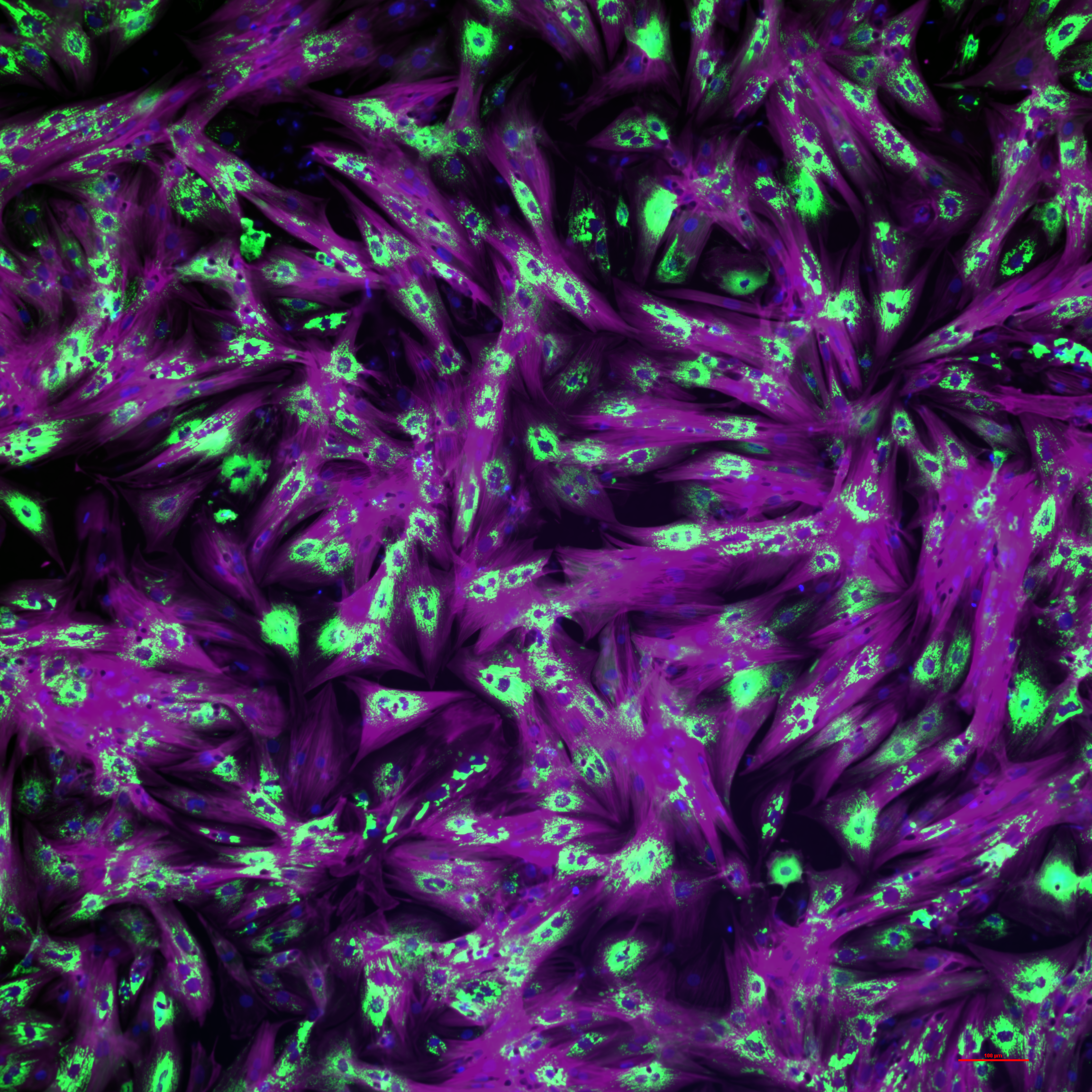

Supplement: Supplementary file 13 — Source data Fig. 6 [file 44321_2026_411_MOESM13_ESM.zip › Figure 6/6A/DMSO Scr.tif]

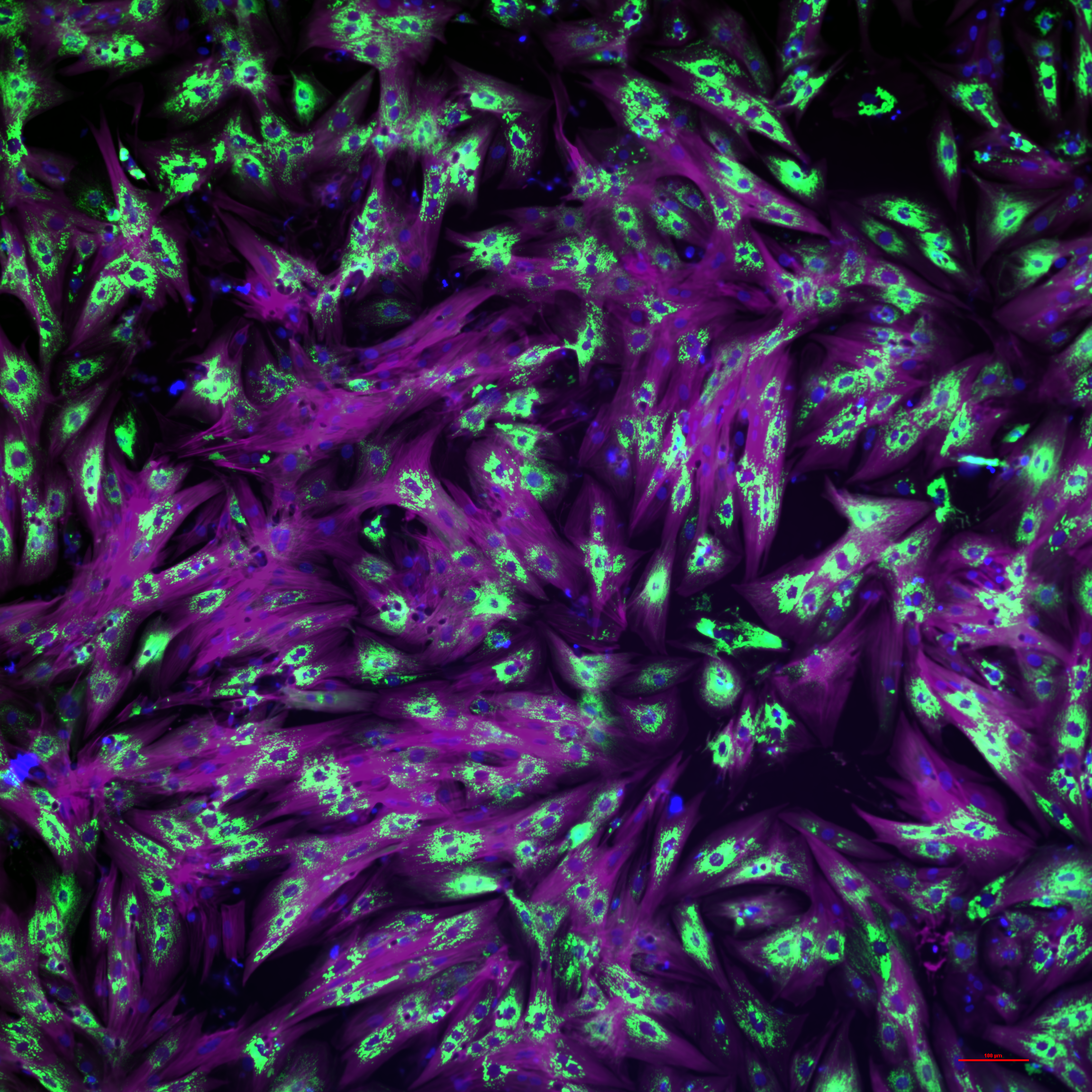

Supplement: Supplementary file 13 — Source data Fig. 6 [file 44321_2026_411_MOESM13_ESM.zip › Figure 6/6A/DMSO siAsb2.tif]

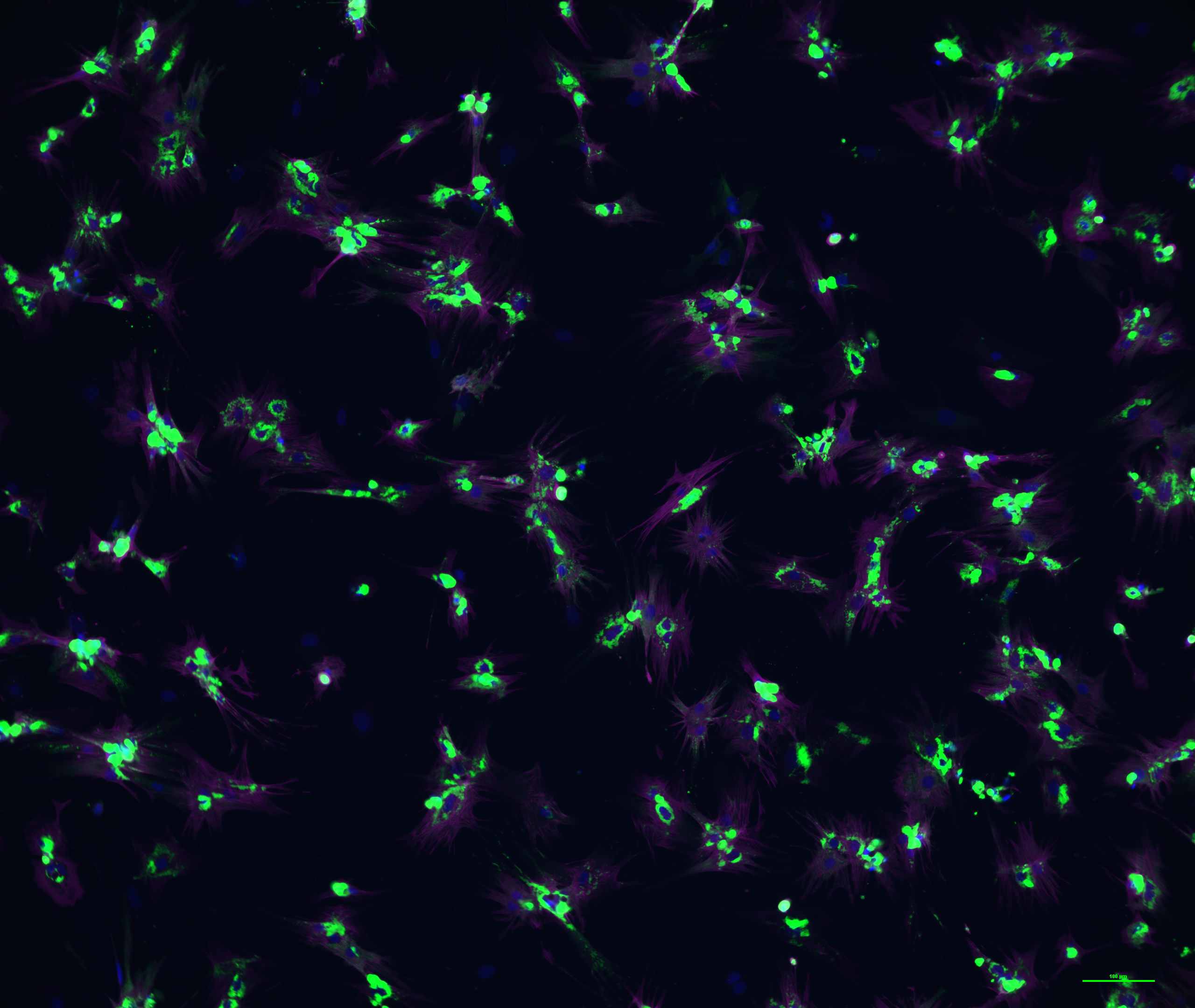

Supplement: Supplementary file 13 — Source data Fig. 6 [file 44321_2026_411_MOESM13_ESM.zip › Figure 6/6A/DMSO.tif]

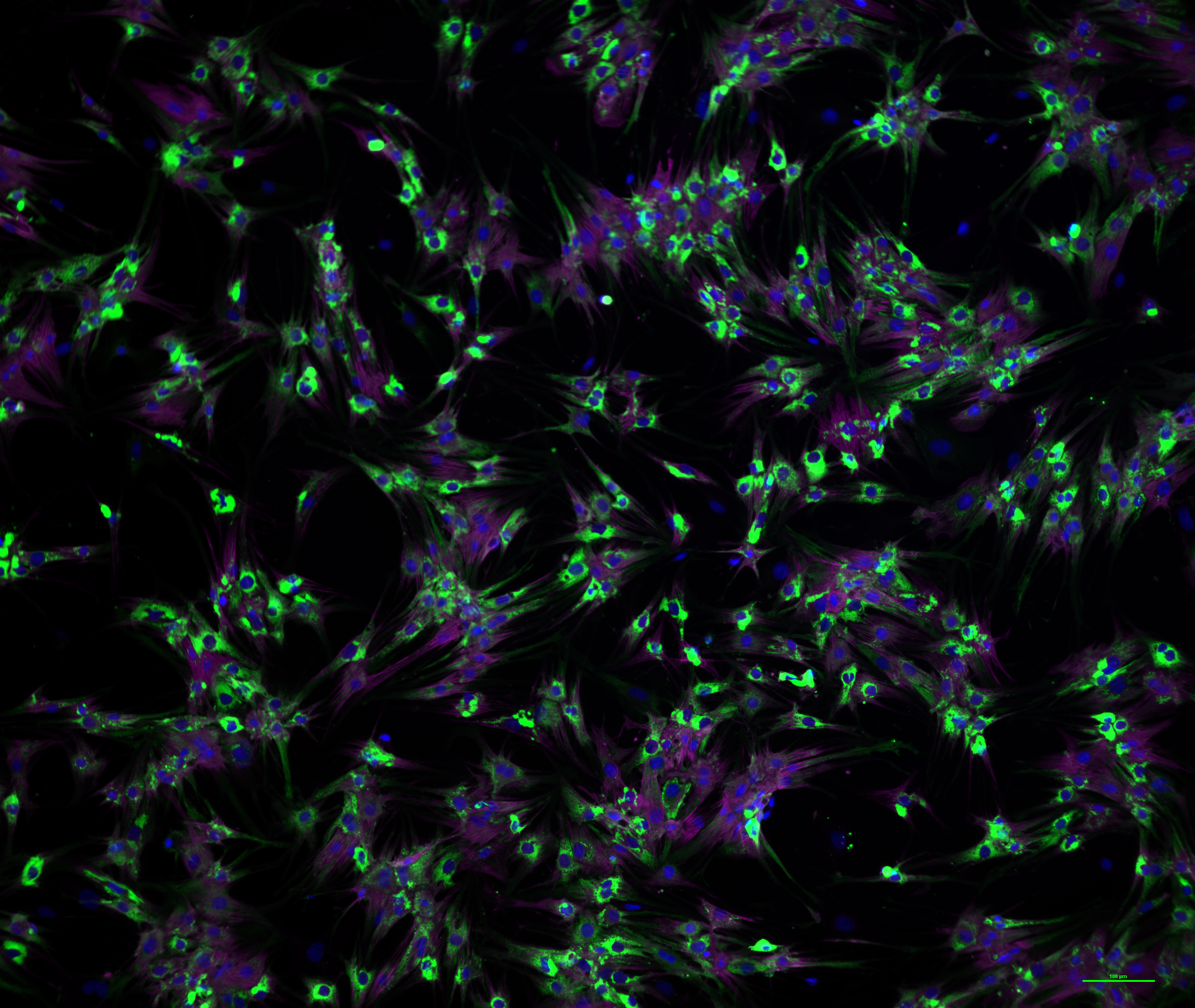

Supplement: Supplementary file 13 — Source data Fig. 6 [file 44321_2026_411_MOESM13_ESM.zip › Figure 6/6A/DMSO_siPsmd1.tif]

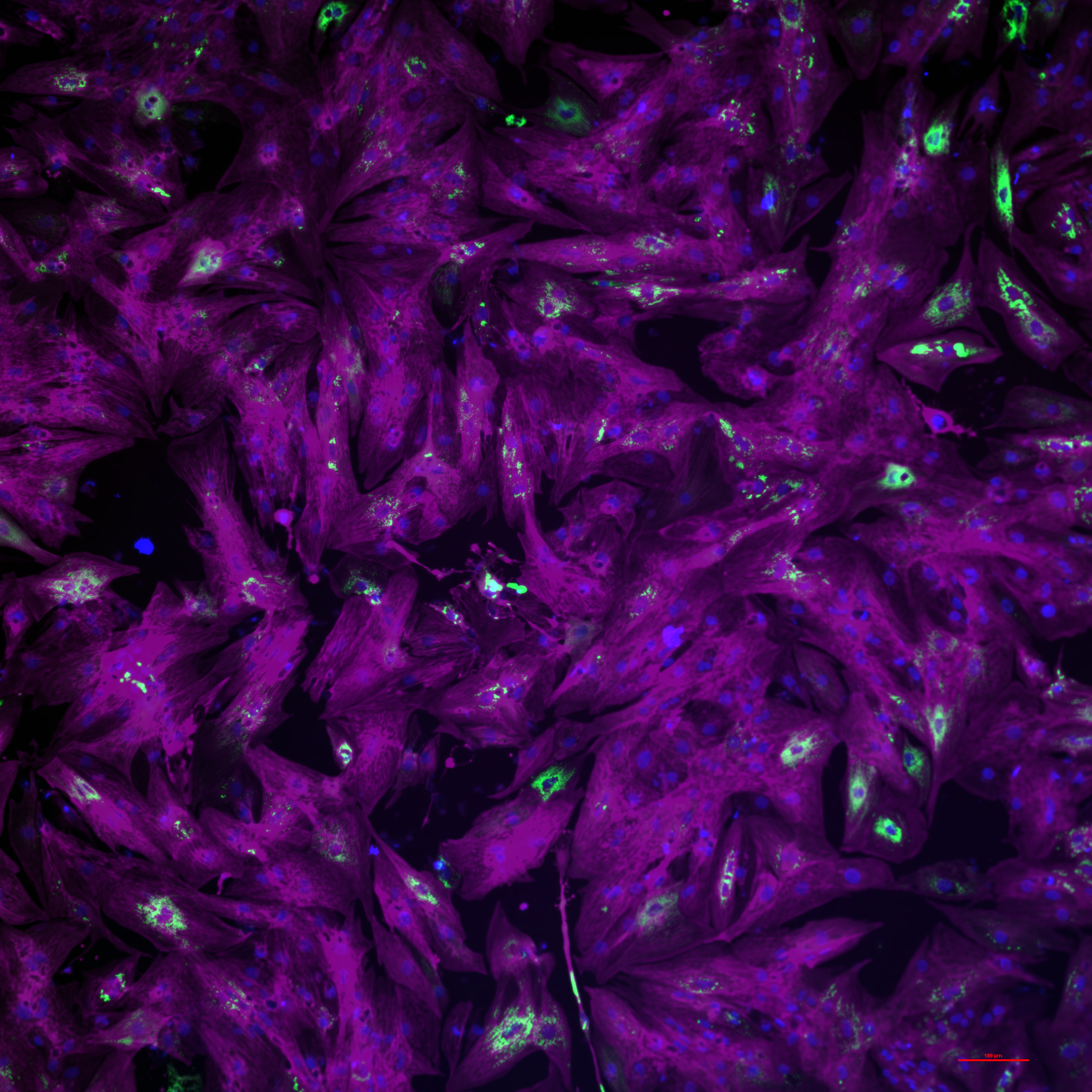

Supplement: Supplementary file 13 — Source data Fig. 6 [file 44321_2026_411_MOESM13_ESM.zip › Figure 6/6A/Ruxo Scr.tif]

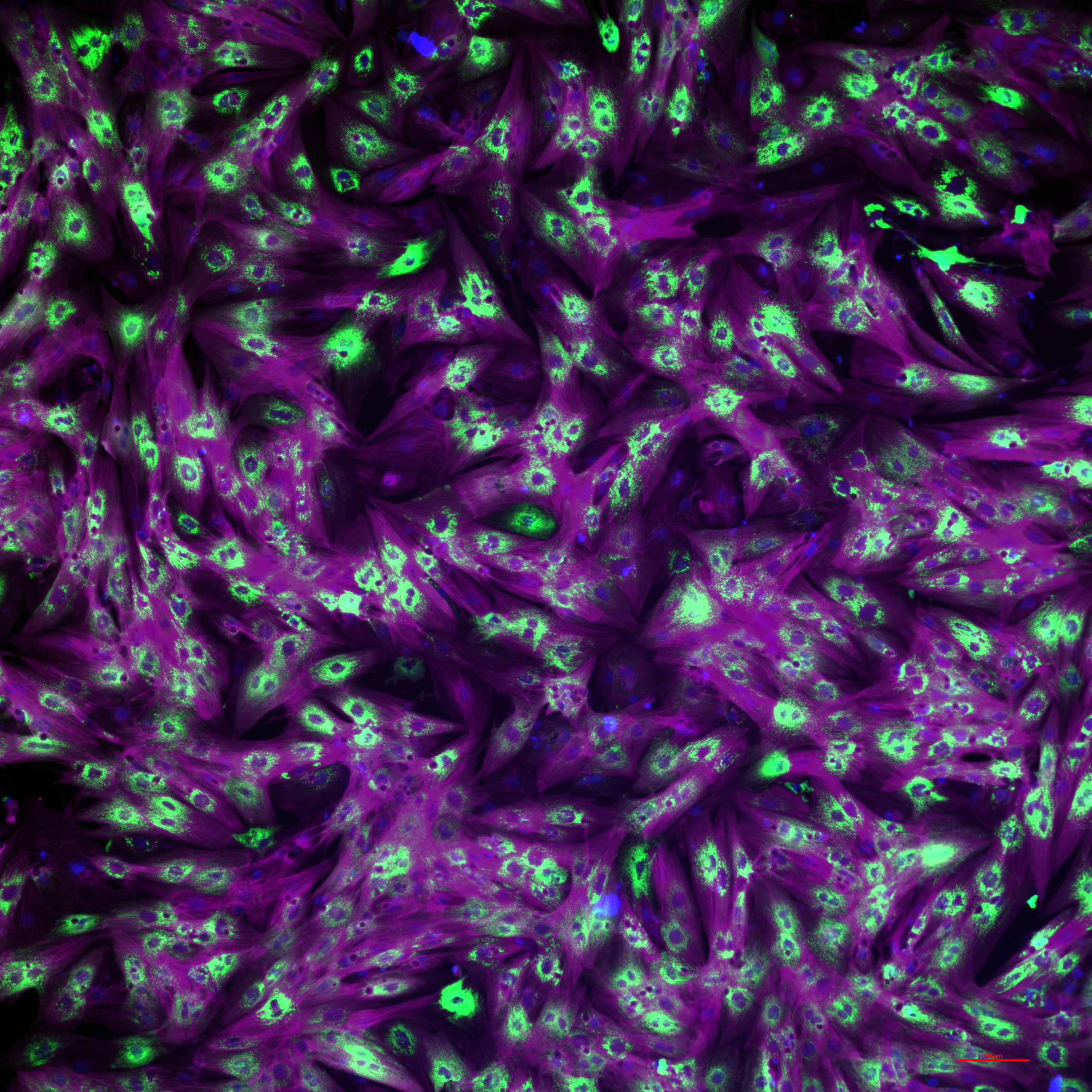

Supplement: Supplementary file 13 — Source data Fig. 6 [file 44321_2026_411_MOESM13_ESM.zip › Figure 6/6A/Ruxo siAsb2.tif]

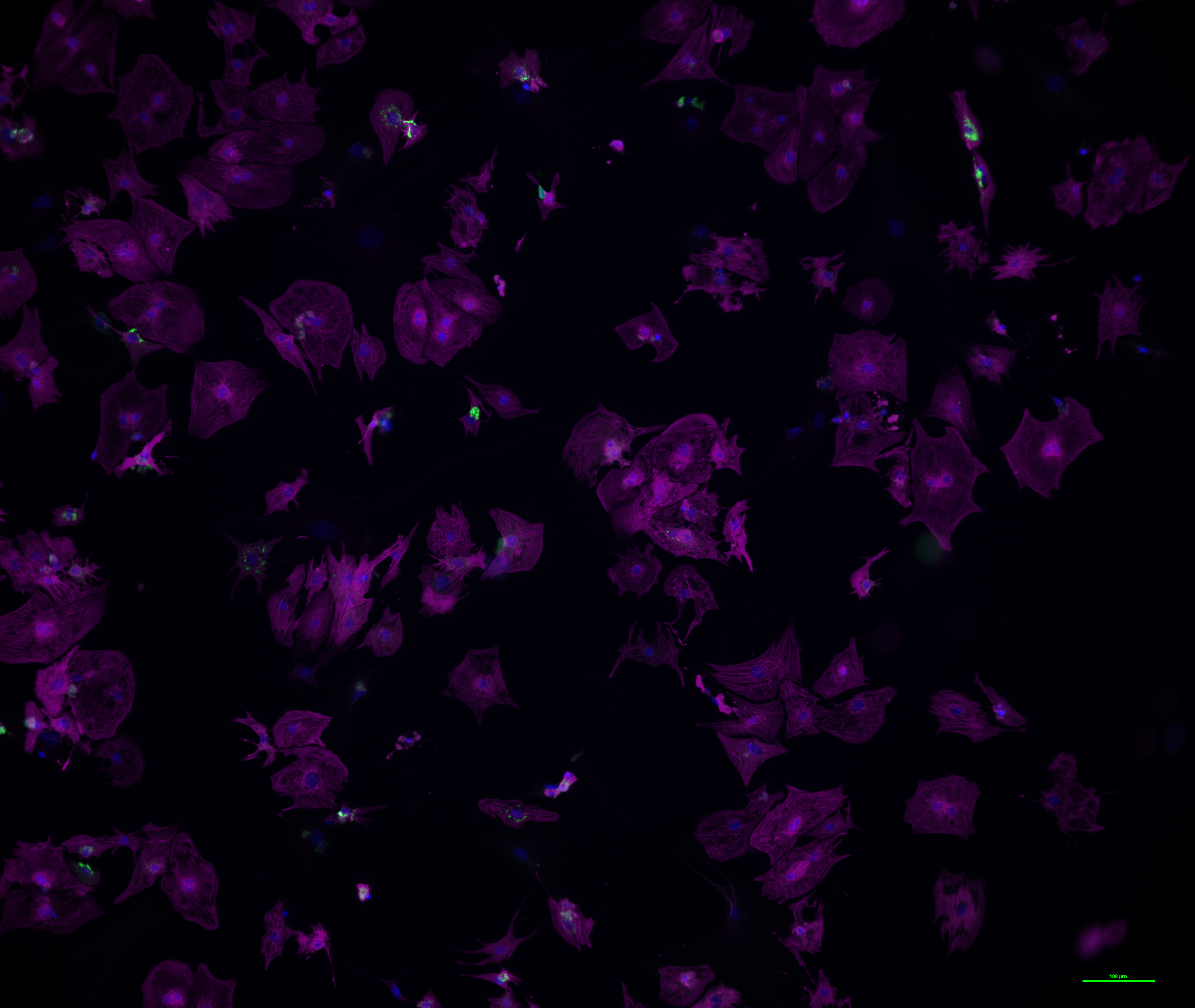

Supplement: Supplementary file 13 — Source data Fig. 6 [file 44321_2026_411_MOESM13_ESM.zip › Figure 6/6A/Ruxo.tif]

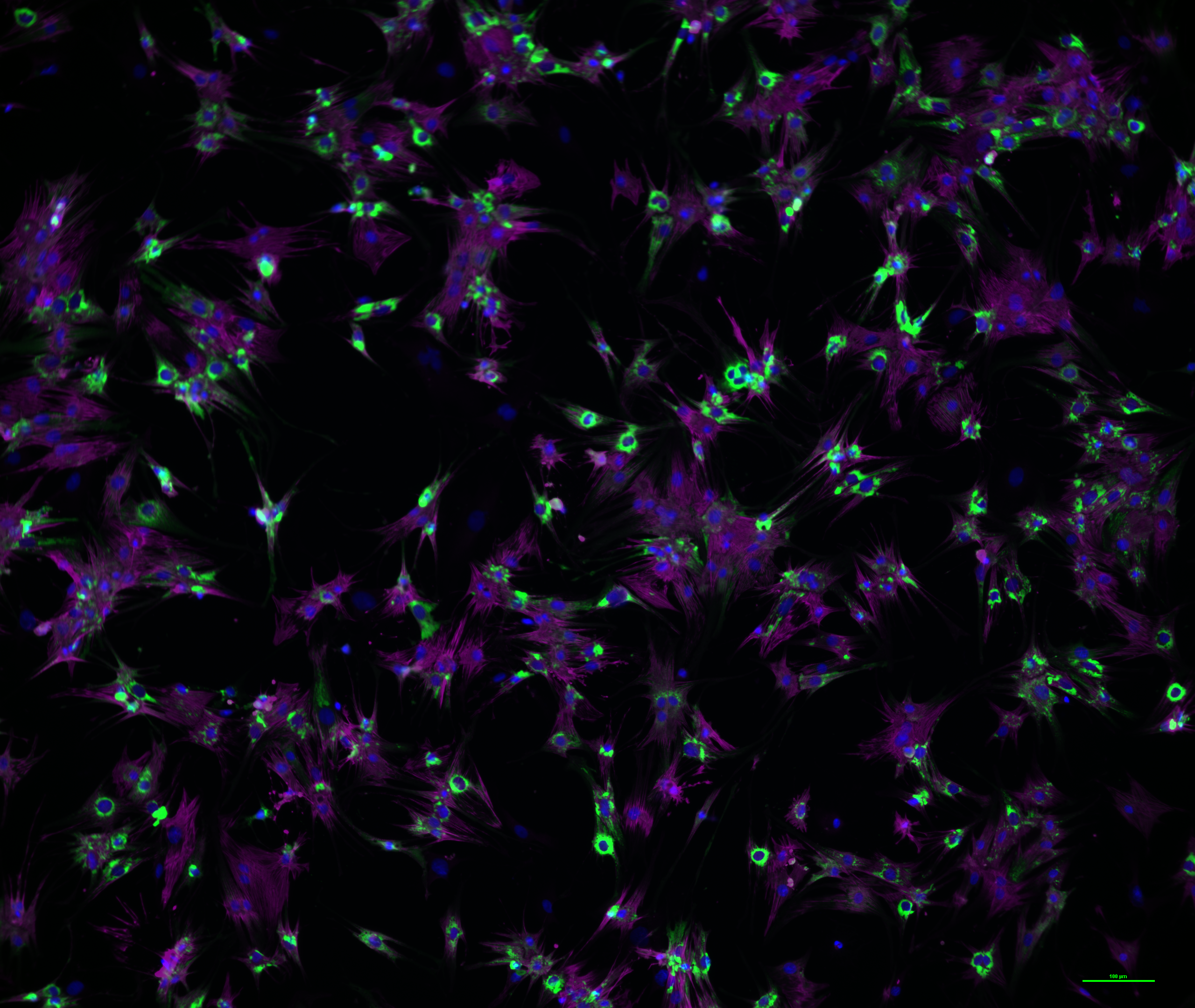

Supplement: Supplementary file 13 — Source data Fig. 6 [file 44321_2026_411_MOESM13_ESM.zip › Figure 6/6A/Ruxo_siPsmd1.tif]

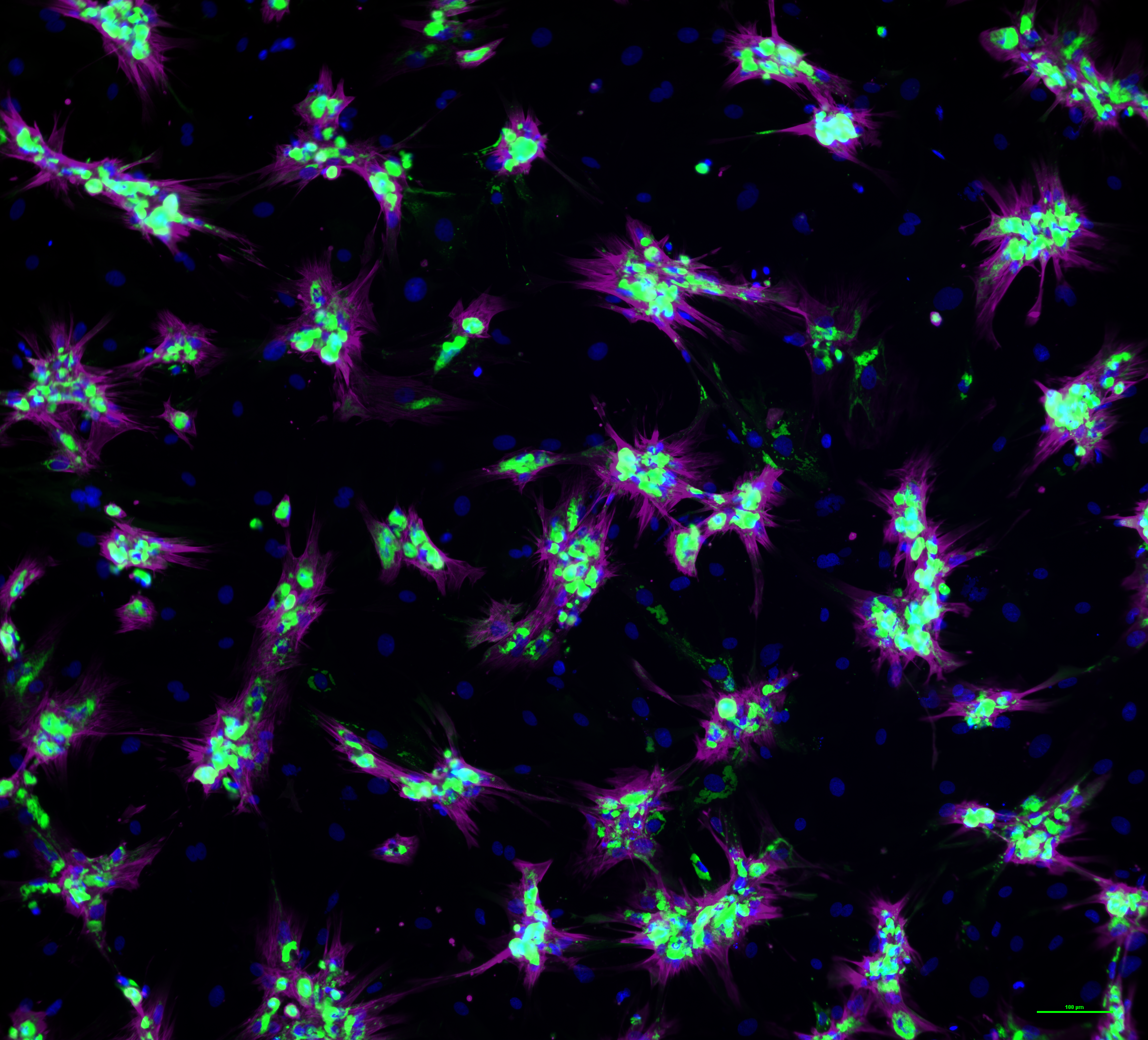

Supplement: Supplementary file 13 — Source data Fig. 6 [file 44321_2026_411_MOESM13_ESM.zip › Figure 6/6A/Scr.tif]

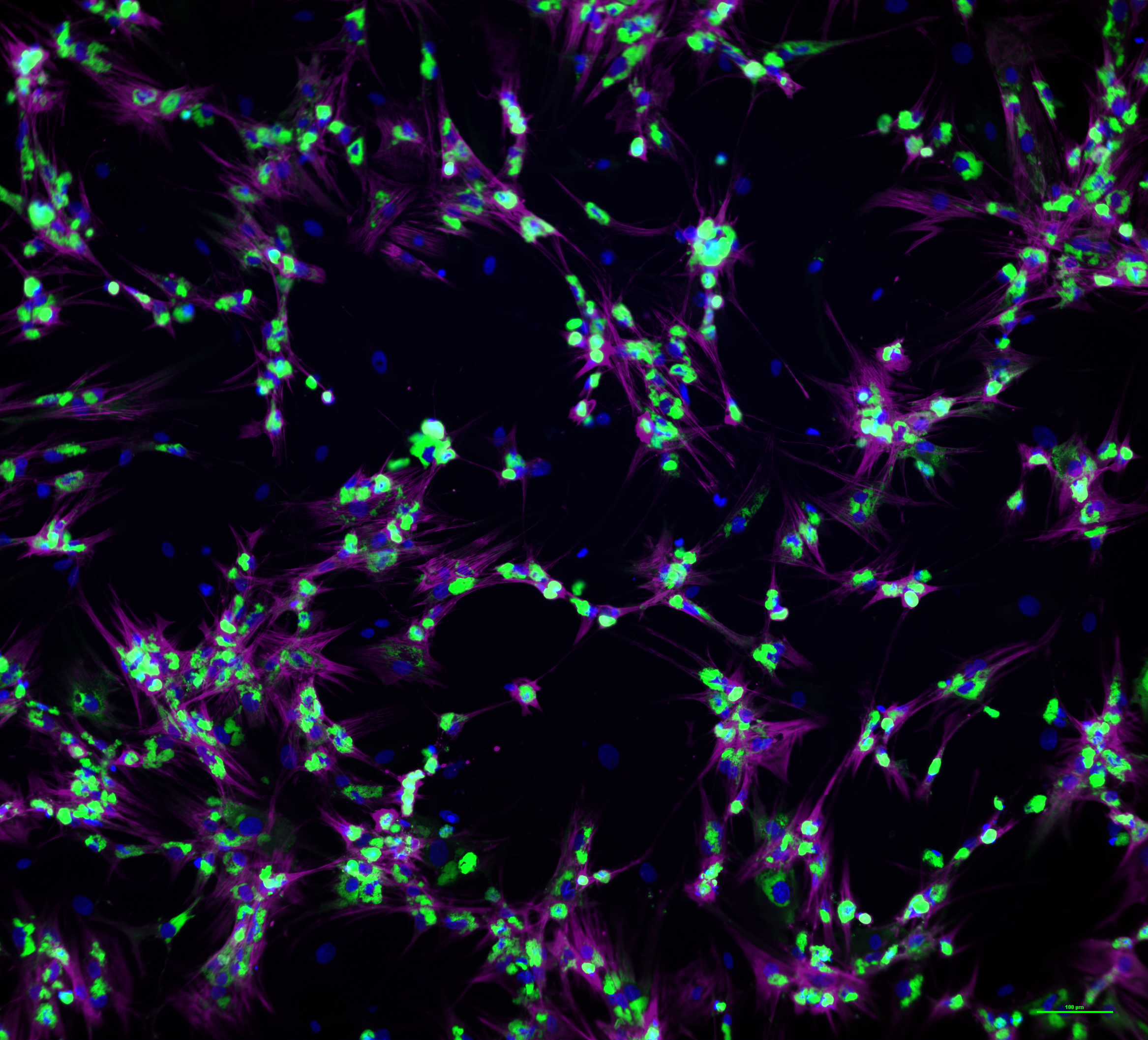

Supplement: Supplementary file 13 — Source data Fig. 6 [file 44321_2026_411_MOESM13_ESM.zip › Figure 6/6A/Scr_siPsmd1.tif]

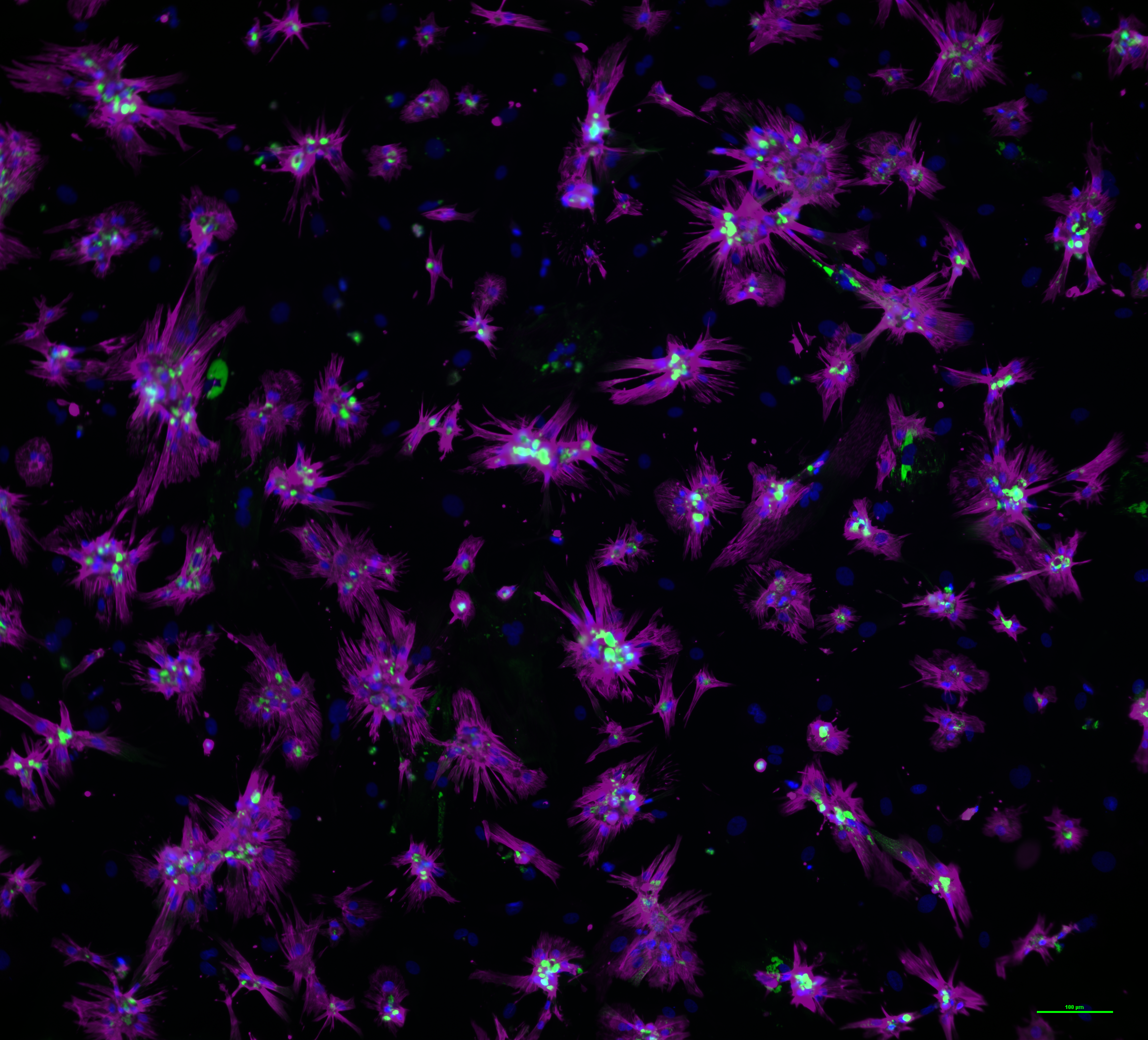

Supplement: Supplementary file 13 — Source data Fig. 6 [file 44321_2026_411_MOESM13_ESM.zip › Figure 6/6A/siJak1.tif]

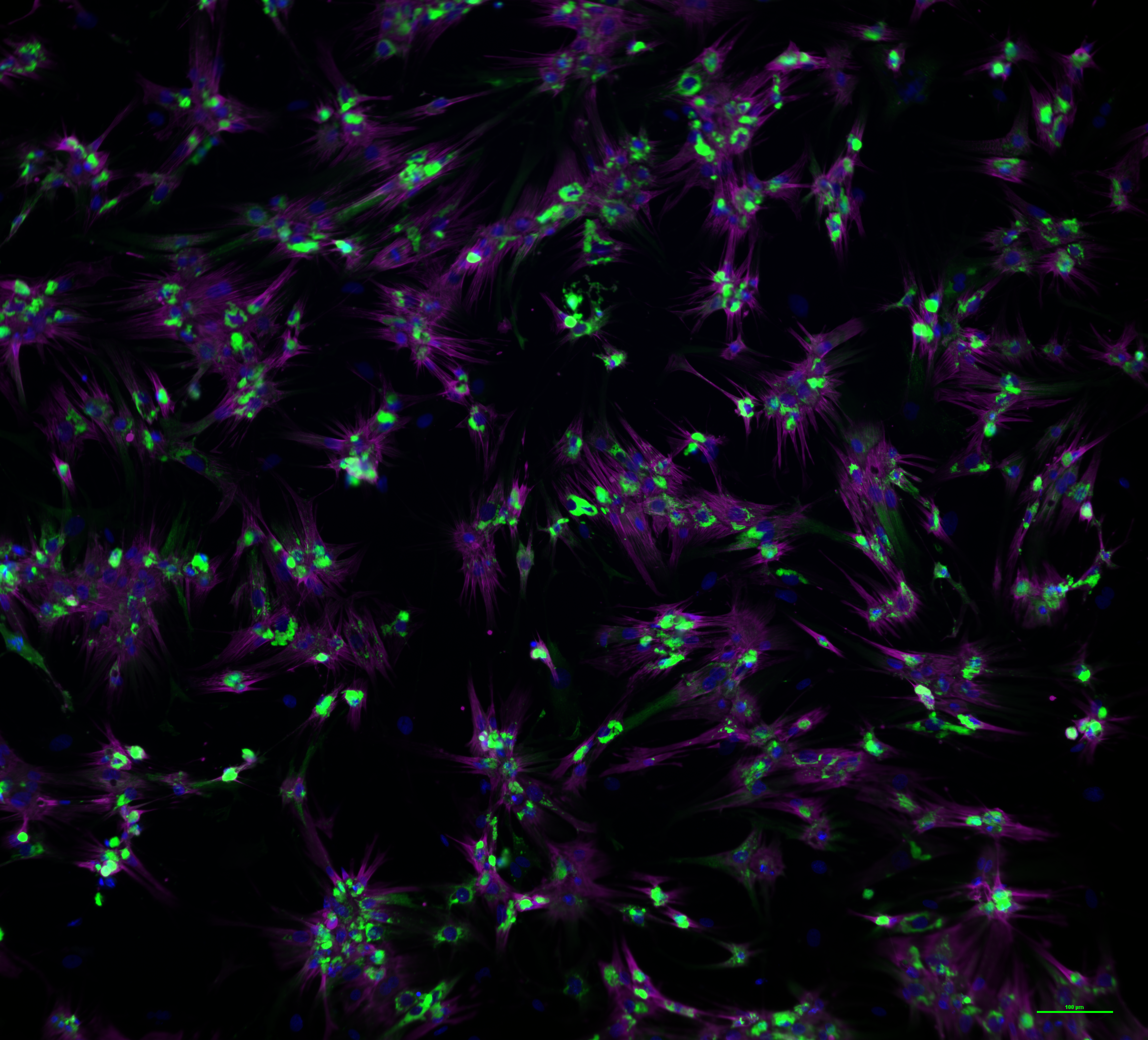

Supplement: Supplementary file 13 — Source data Fig. 6 [file 44321_2026_411_MOESM13_ESM.zip › Figure 6/6A/siJak1_siPsmd1.tif]

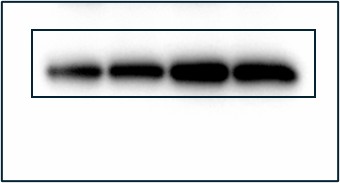

Supplement: Supplementary file 13 — Source data Fig. 6 [file 44321_2026_411_MOESM13_ESM.zip › Figure 6/6B/6B Western CRYAB.jpg]

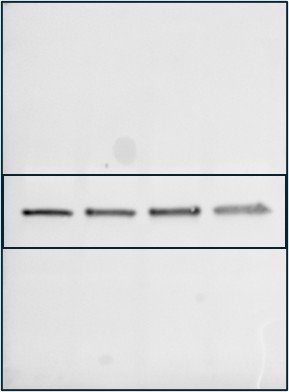

Supplement: Supplementary file 13 — Source data Fig. 6 [file 44321_2026_411_MOESM13_ESM.zip › Figure 6/6B/6B Western GAPDH 2.jpg]

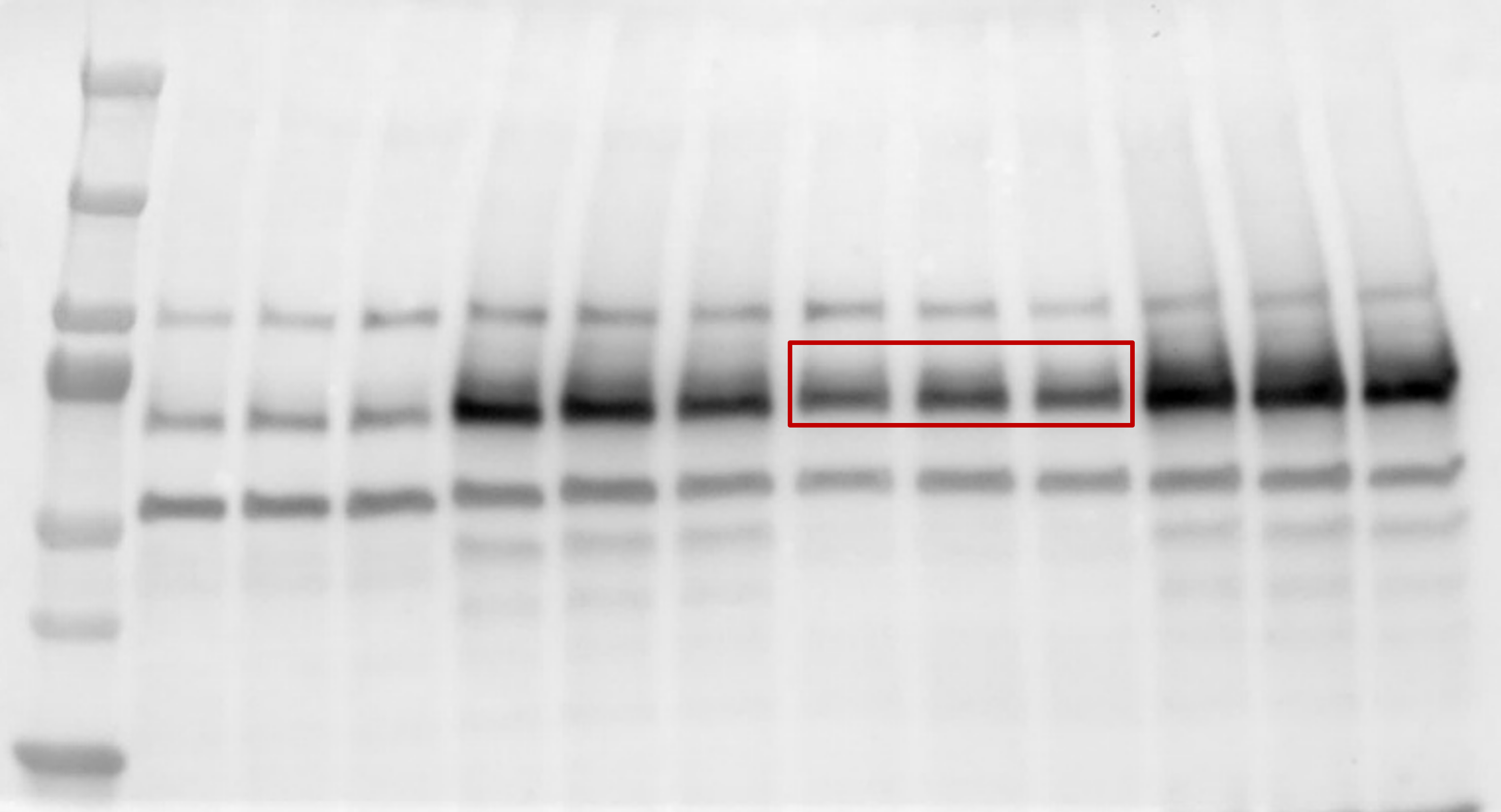

Supplement: Supplementary file 13 — Source data Fig. 6 [file 44321_2026_411_MOESM13_ESM.zip › Figure 6/6C/western Insoluble Asb2B 1.tif]

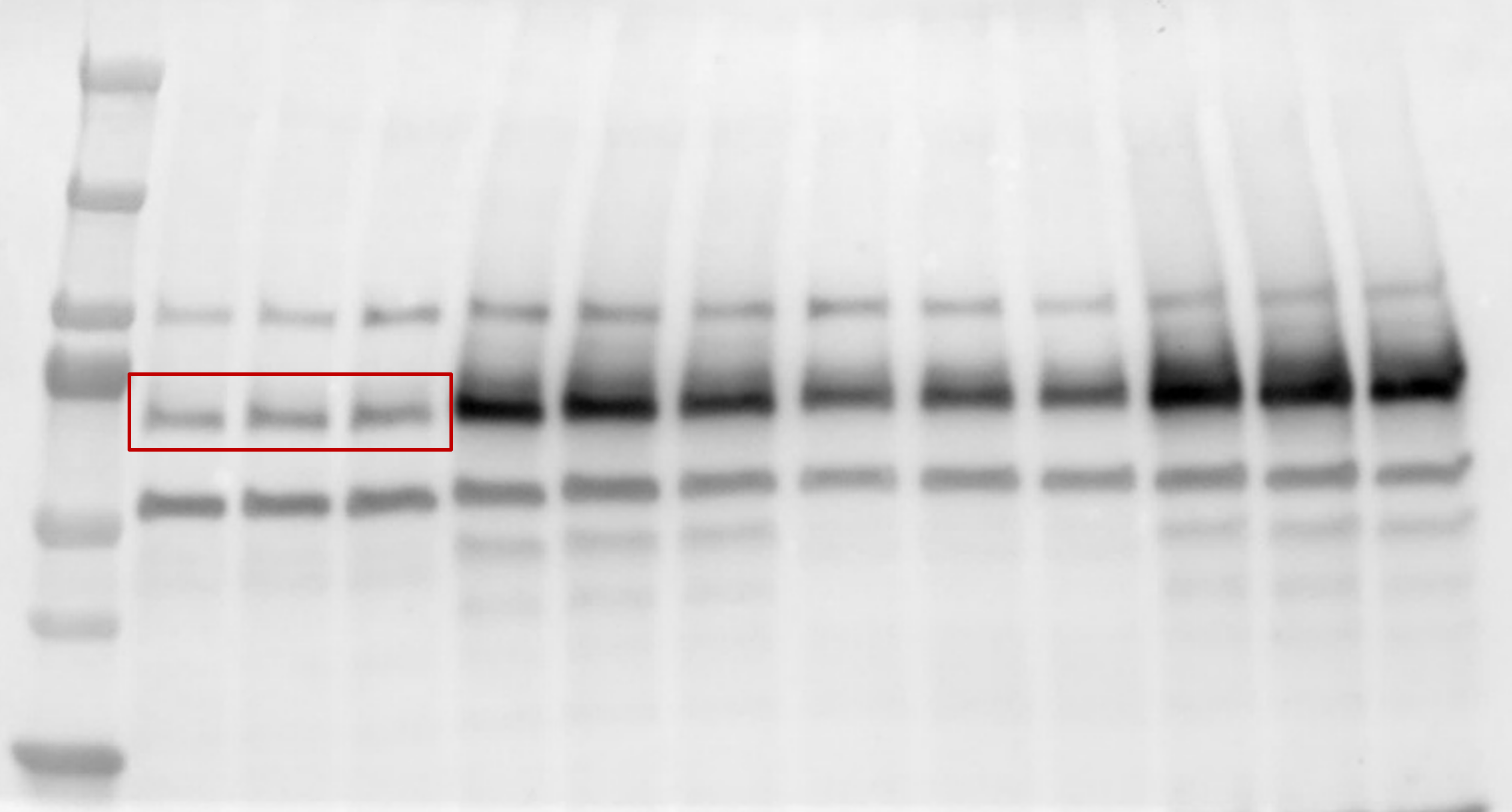

Supplement: Supplementary file 13 — Source data Fig. 6 [file 44321_2026_411_MOESM13_ESM.zip › Figure 6/6C/western Insoluble Asb2B.tif]

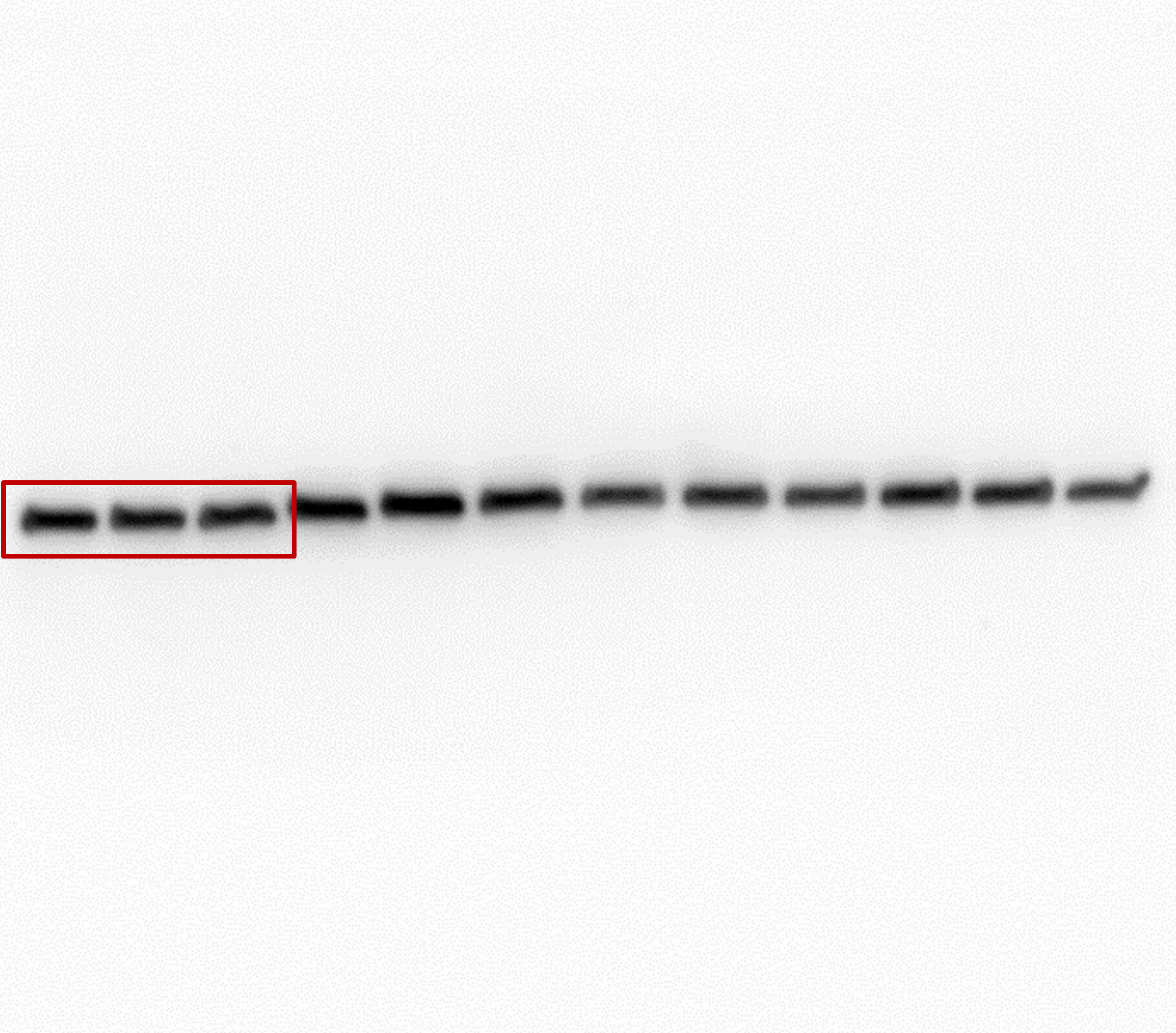

Supplement: Supplementary file 13 — Source data Fig. 6 [file 44321_2026_411_MOESM13_ESM.zip › Figure 6/6C/western Insoluble DES.tif]

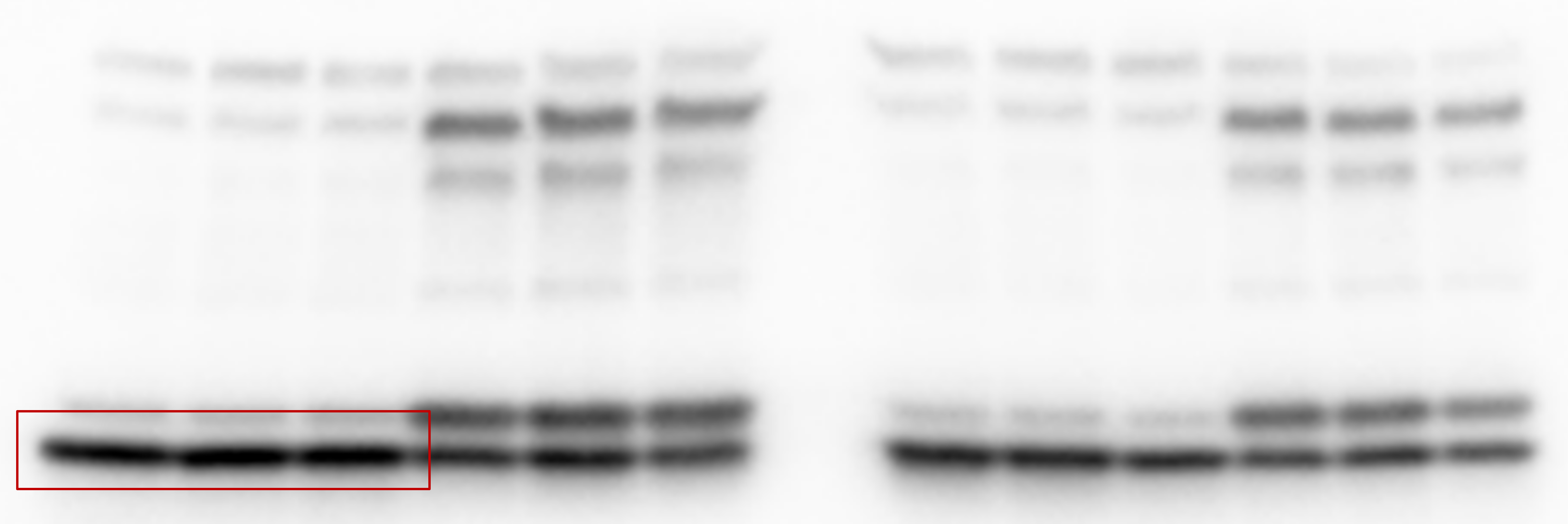

Supplement: Supplementary file 13 — Source data Fig. 6 [file 44321_2026_411_MOESM13_ESM.zip › Figure 6/6C/western Insoluble H3 2.tif]

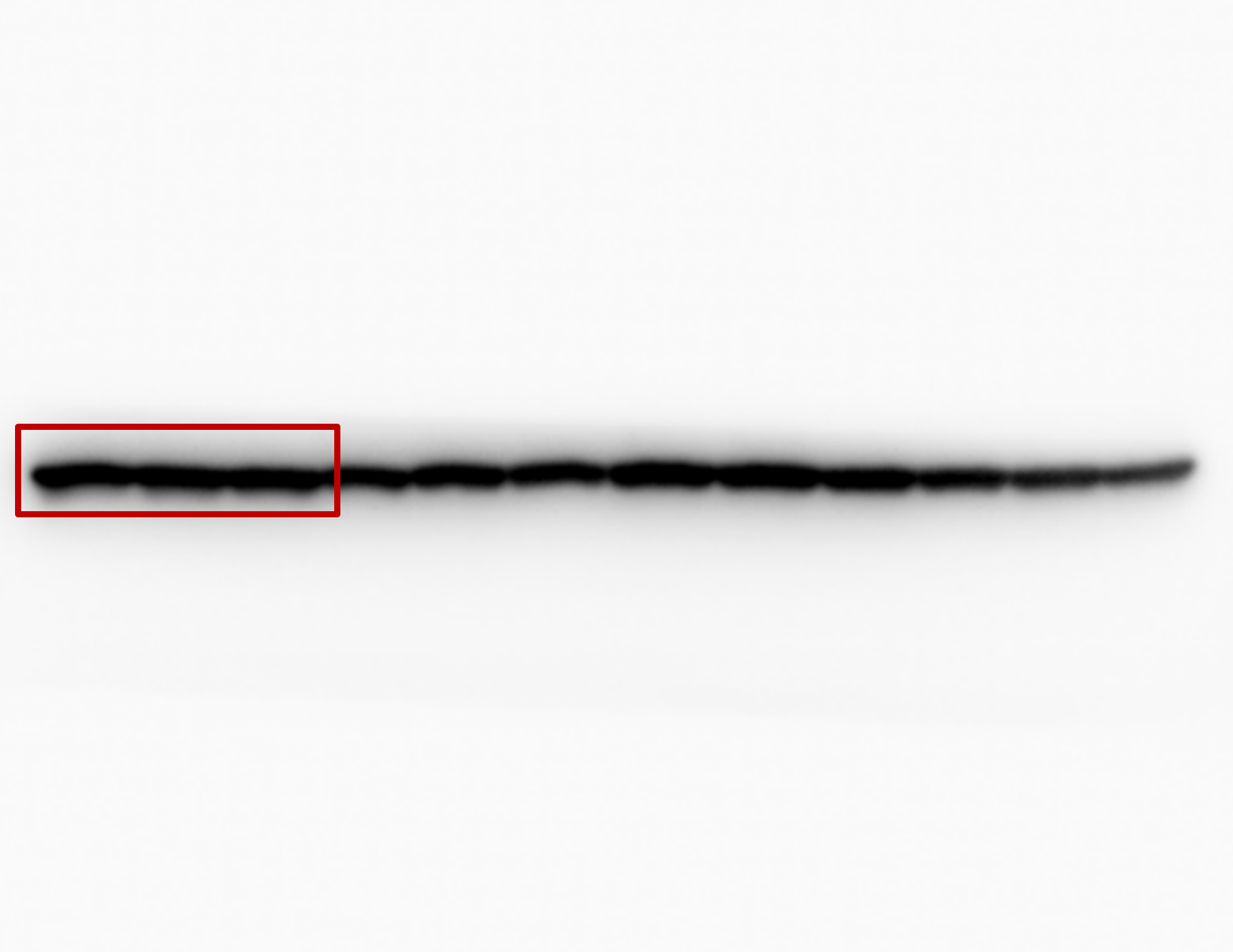

Supplement: Supplementary file 13 — Source data Fig. 6 [file 44321_2026_411_MOESM13_ESM.zip › Figure 6/6C/western Insoluble H3.tif]

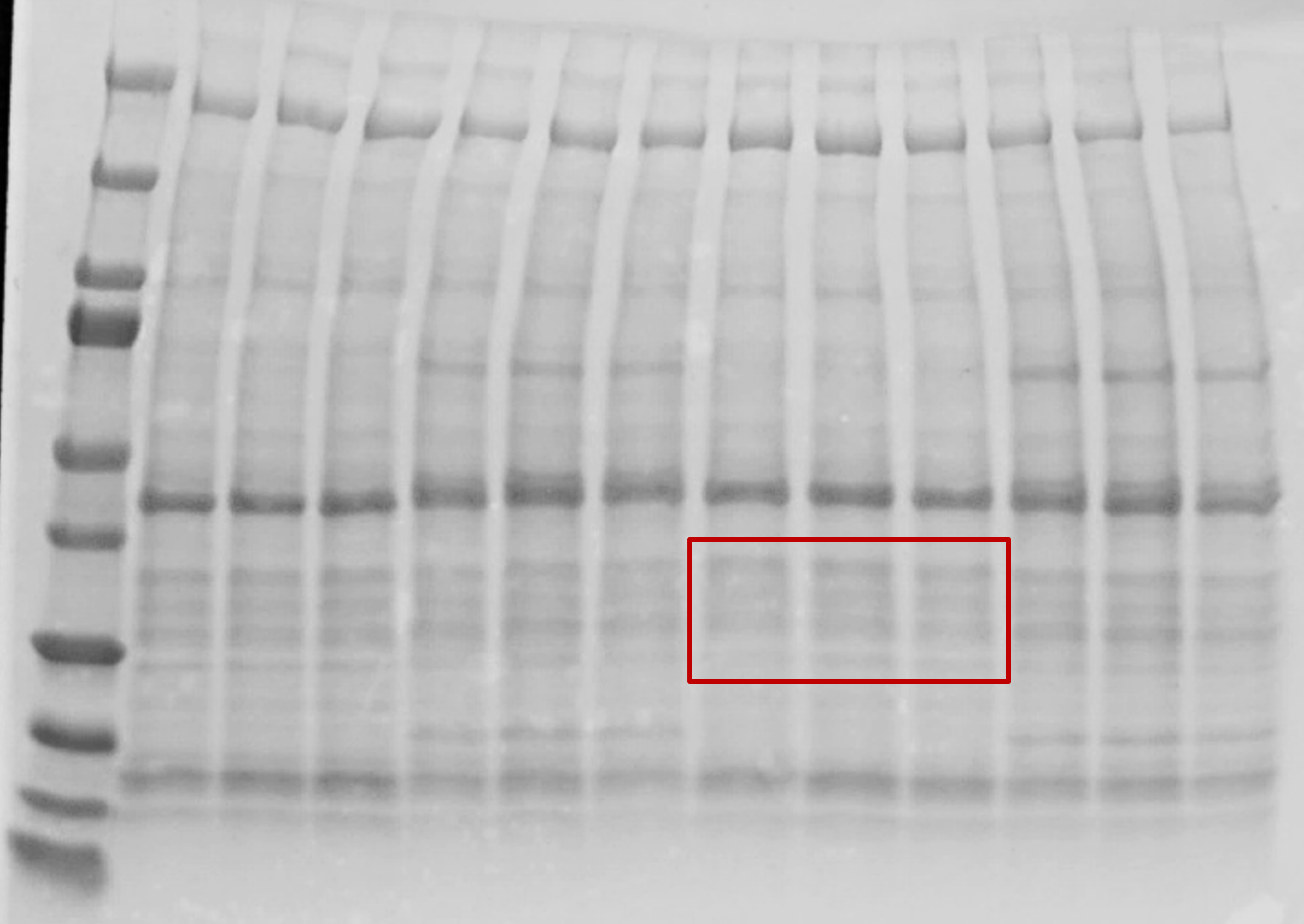

Supplement: Supplementary file 13 — Source data Fig. 6 [file 44321_2026_411_MOESM13_ESM.zip › Figure 6/6C/western Insoluble Ponceau 1.tif]

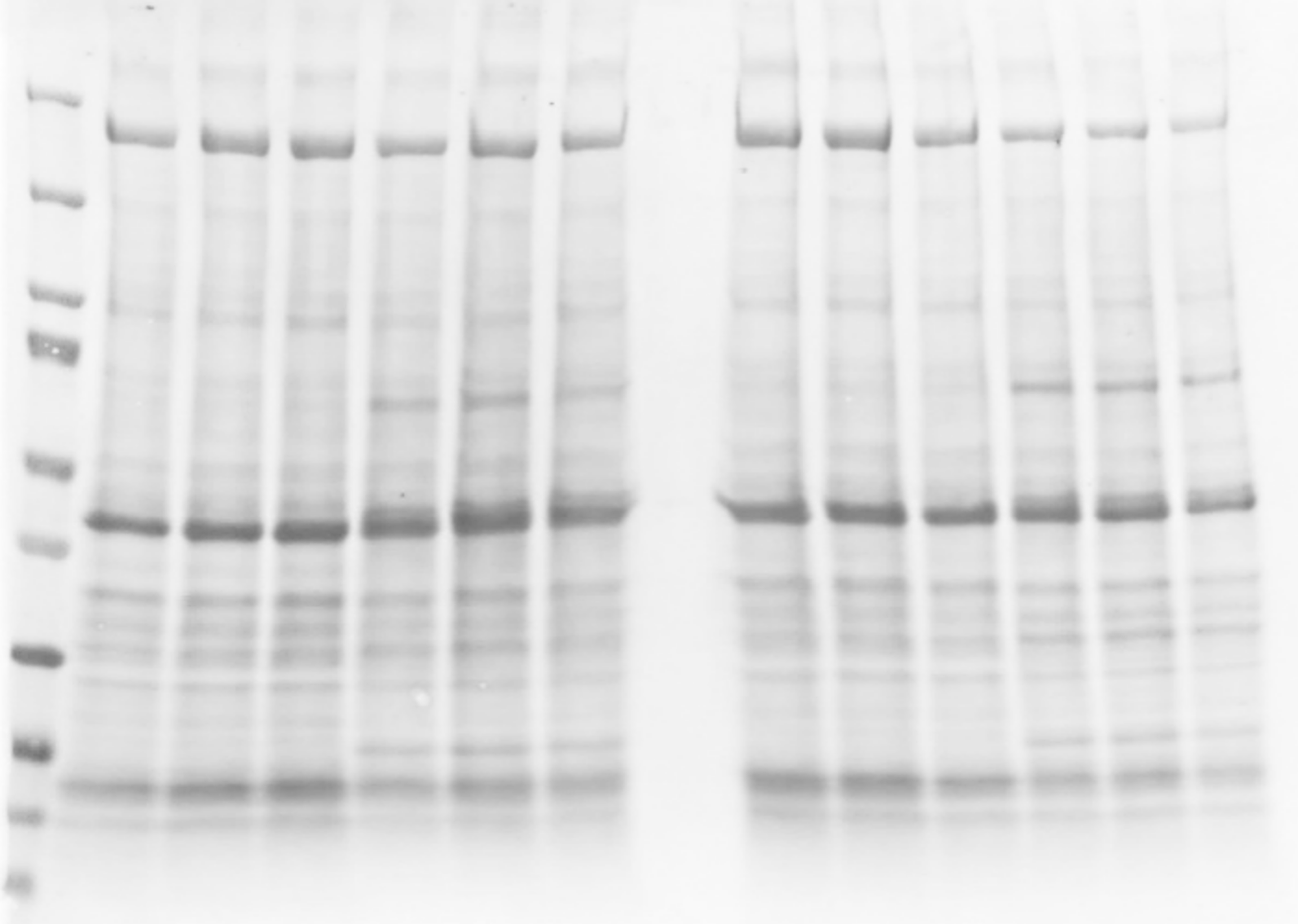

Supplement: Supplementary file 13 — Source data Fig. 6 [file 44321_2026_411_MOESM13_ESM.zip › Figure 6/6C/western Insoluble Ponceau 2.tif]

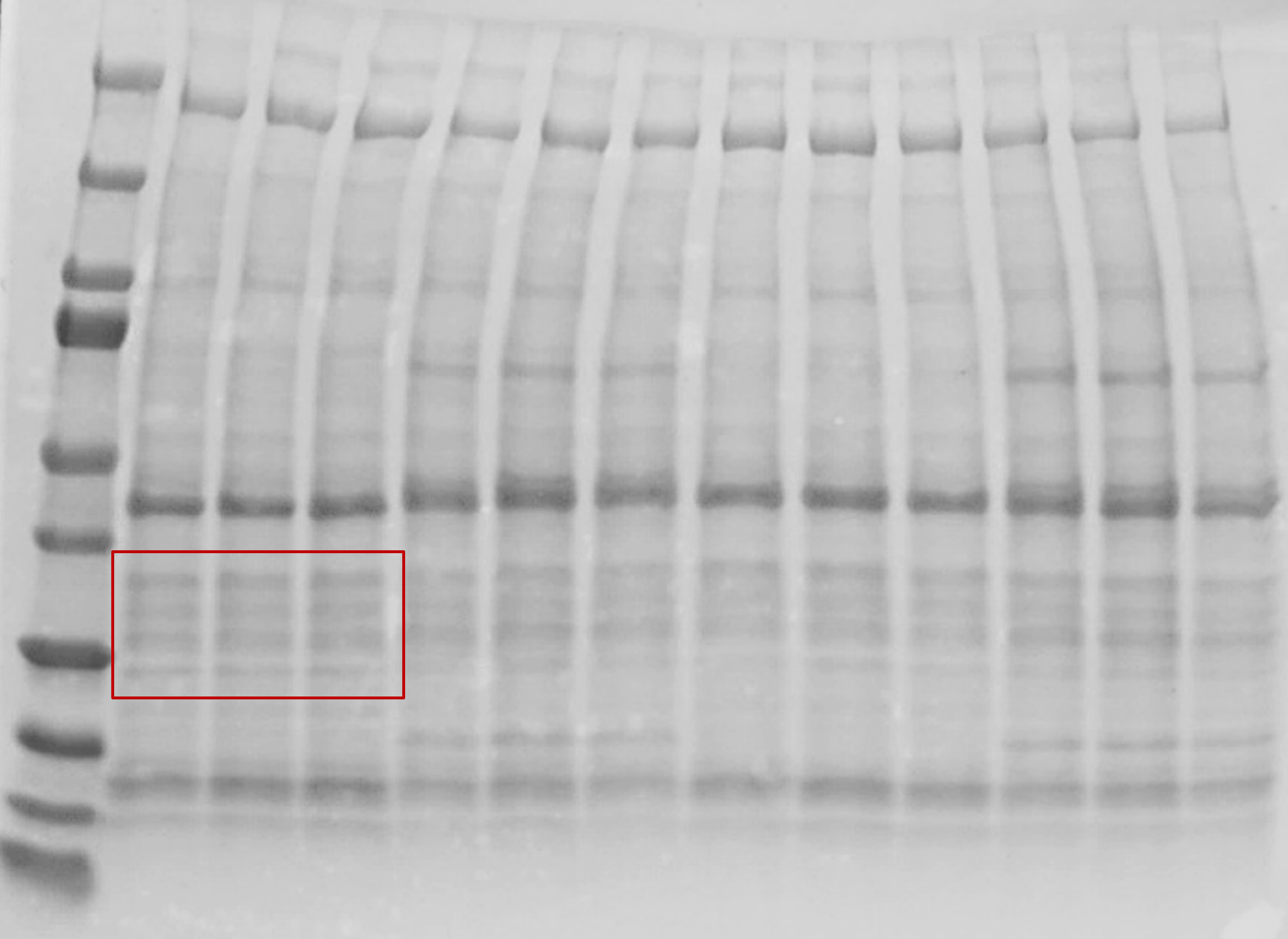

Supplement: Supplementary file 13 — Source data Fig. 6 [file 44321_2026_411_MOESM13_ESM.zip › Figure 6/6C/western Insoluble Ponceau.tif]

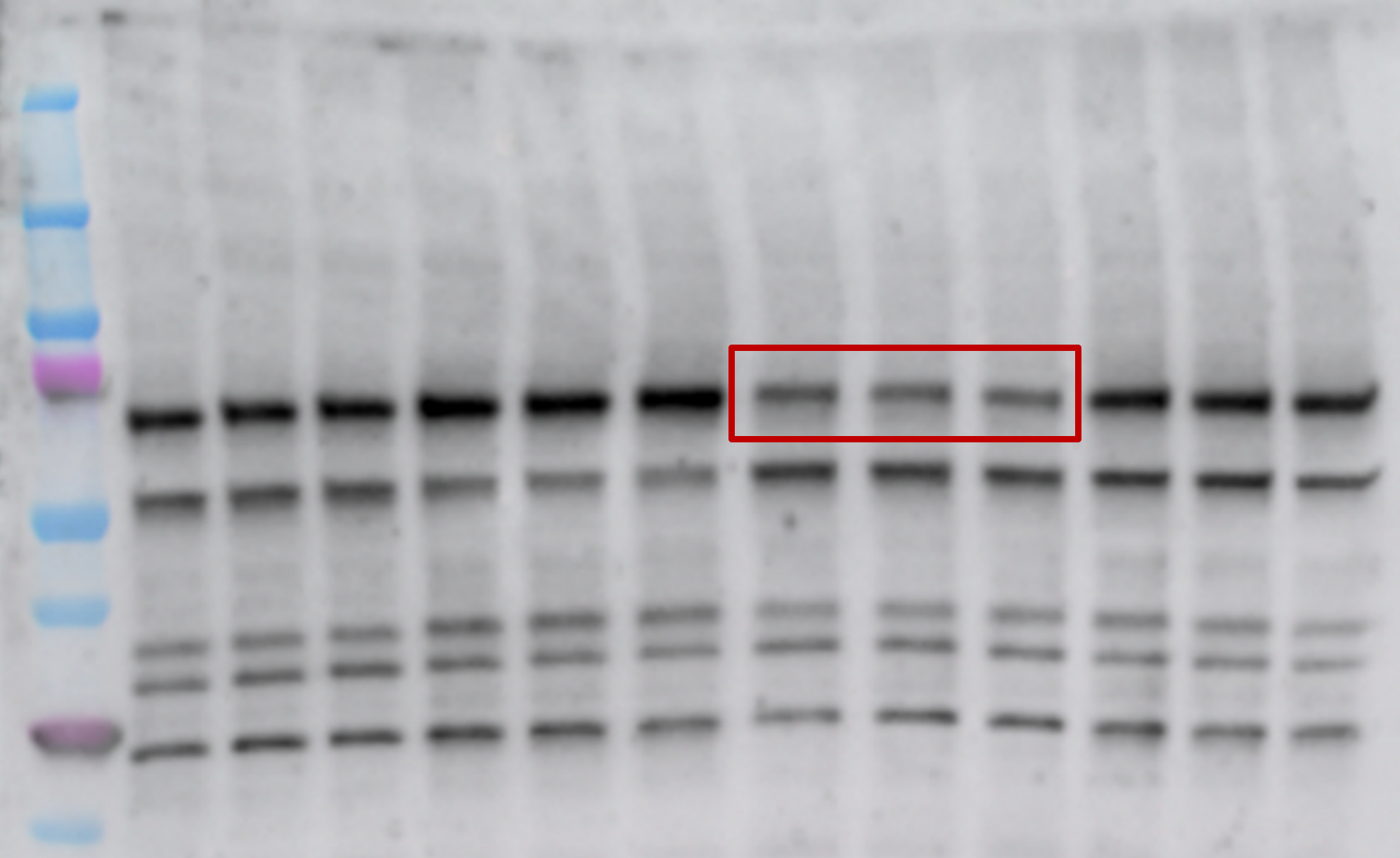

Supplement: Supplementary file 13 — Source data Fig. 6 [file 44321_2026_411_MOESM13_ESM.zip › Figure 6/6C/western Soluble Asb2B 1.tif]

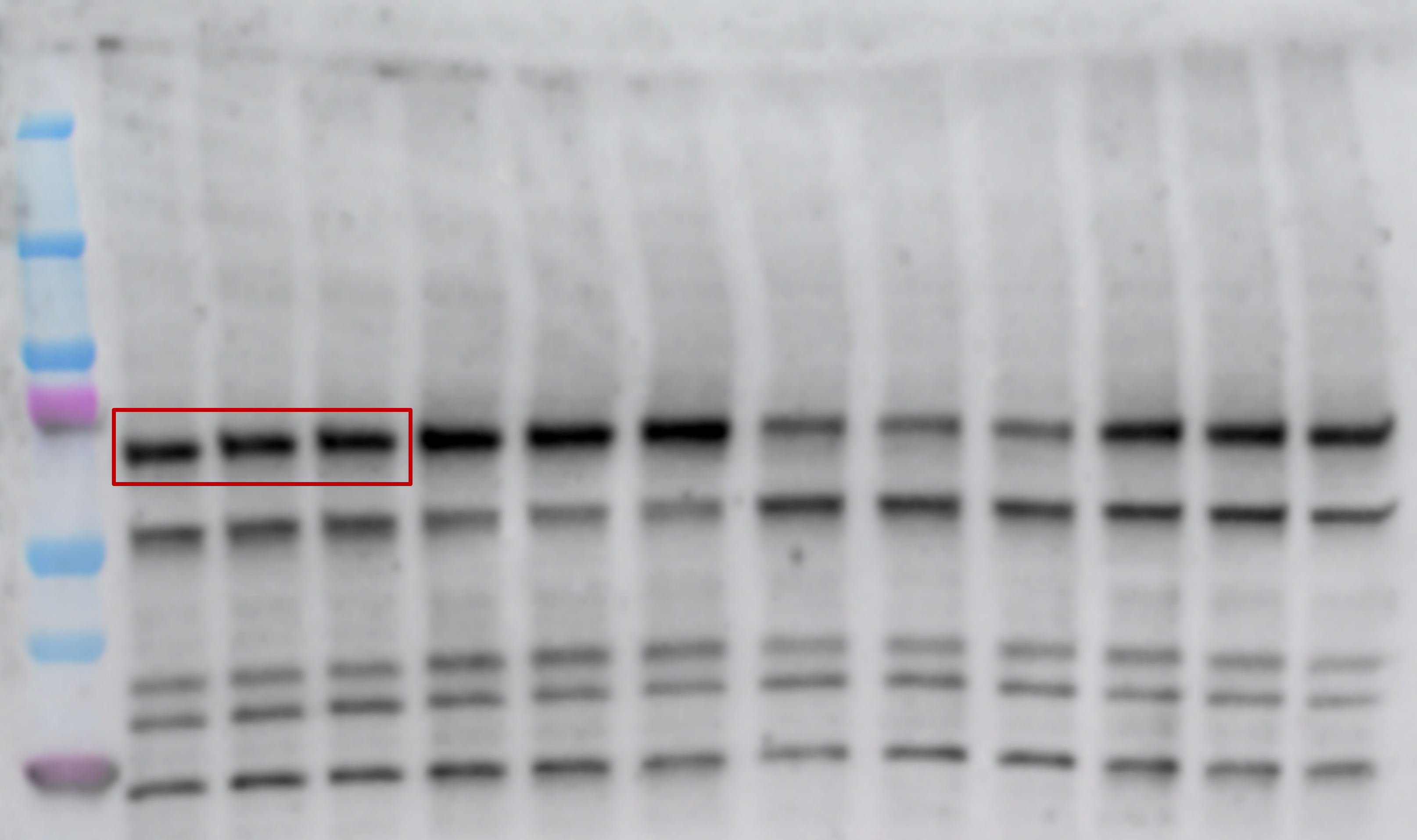

Supplement: Supplementary file 13 — Source data Fig. 6 [file 44321_2026_411_MOESM13_ESM.zip › Figure 6/6C/western Soluble Asb2B.tif]

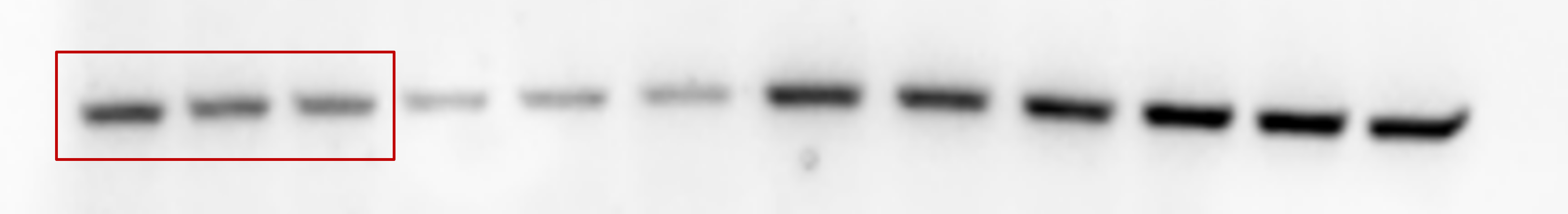

Supplement: Supplementary file 13 — Source data Fig. 6 [file 44321_2026_411_MOESM13_ESM.zip › Figure 6/6C/western Soluble DES.tif]

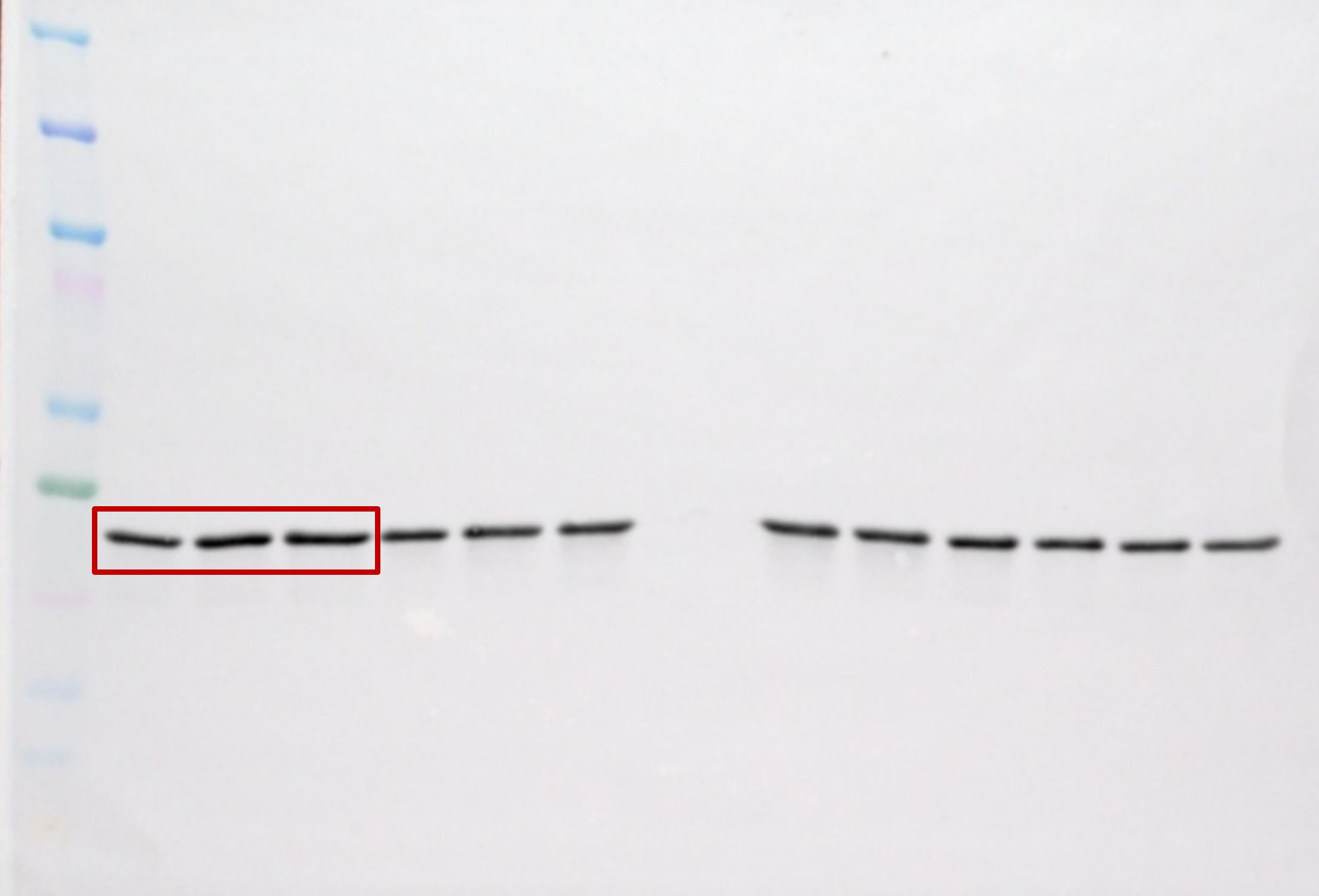

Supplement: Supplementary file 13 — Source data Fig. 6 [file 44321_2026_411_MOESM13_ESM.zip › Figure 6/6C/western Soluble GAPDH 2.tif]

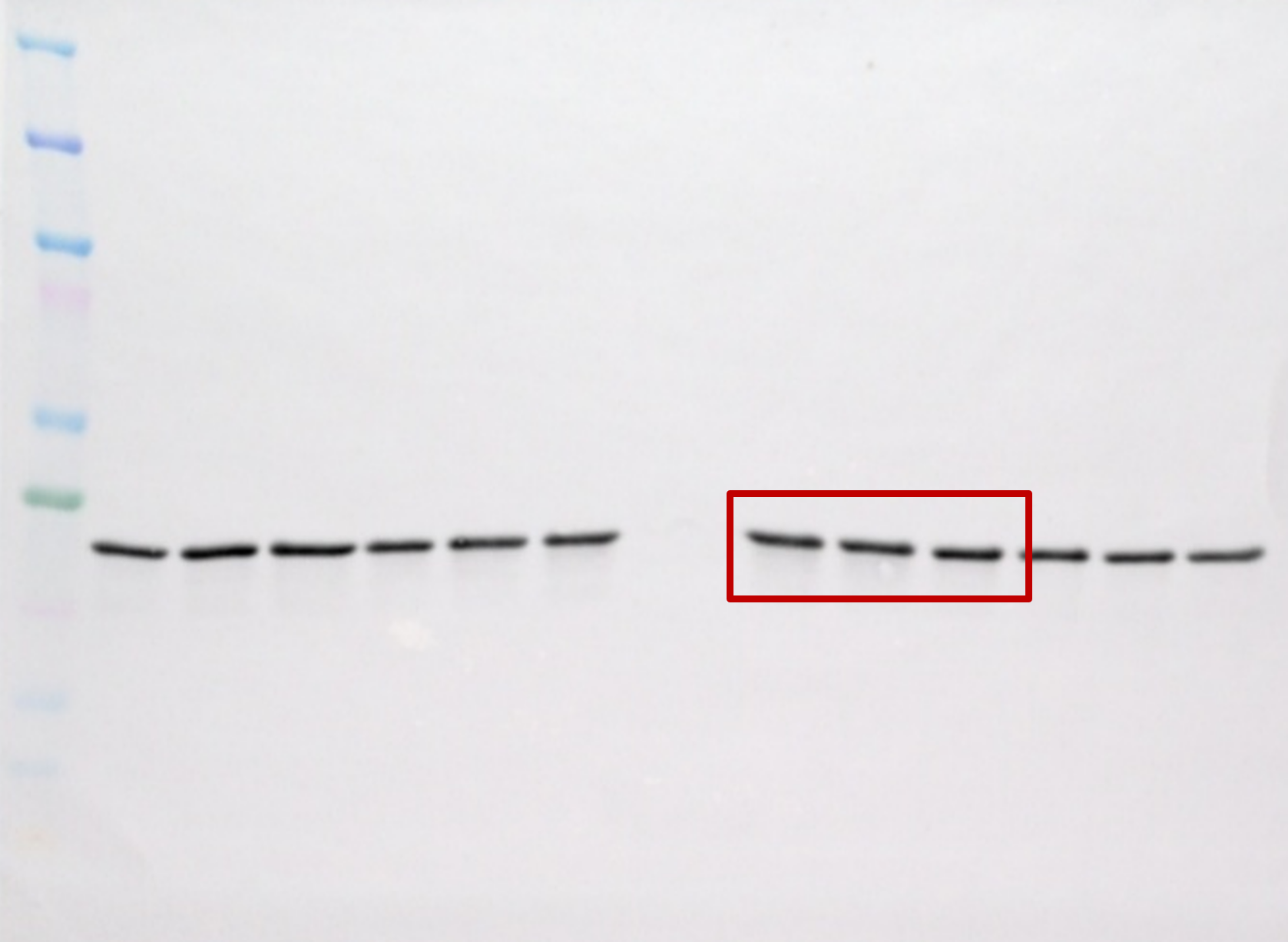

Supplement: Supplementary file 13 — Source data Fig. 6 [file 44321_2026_411_MOESM13_ESM.zip › Figure 6/6C/western Soluble GAPDH 3.tif]

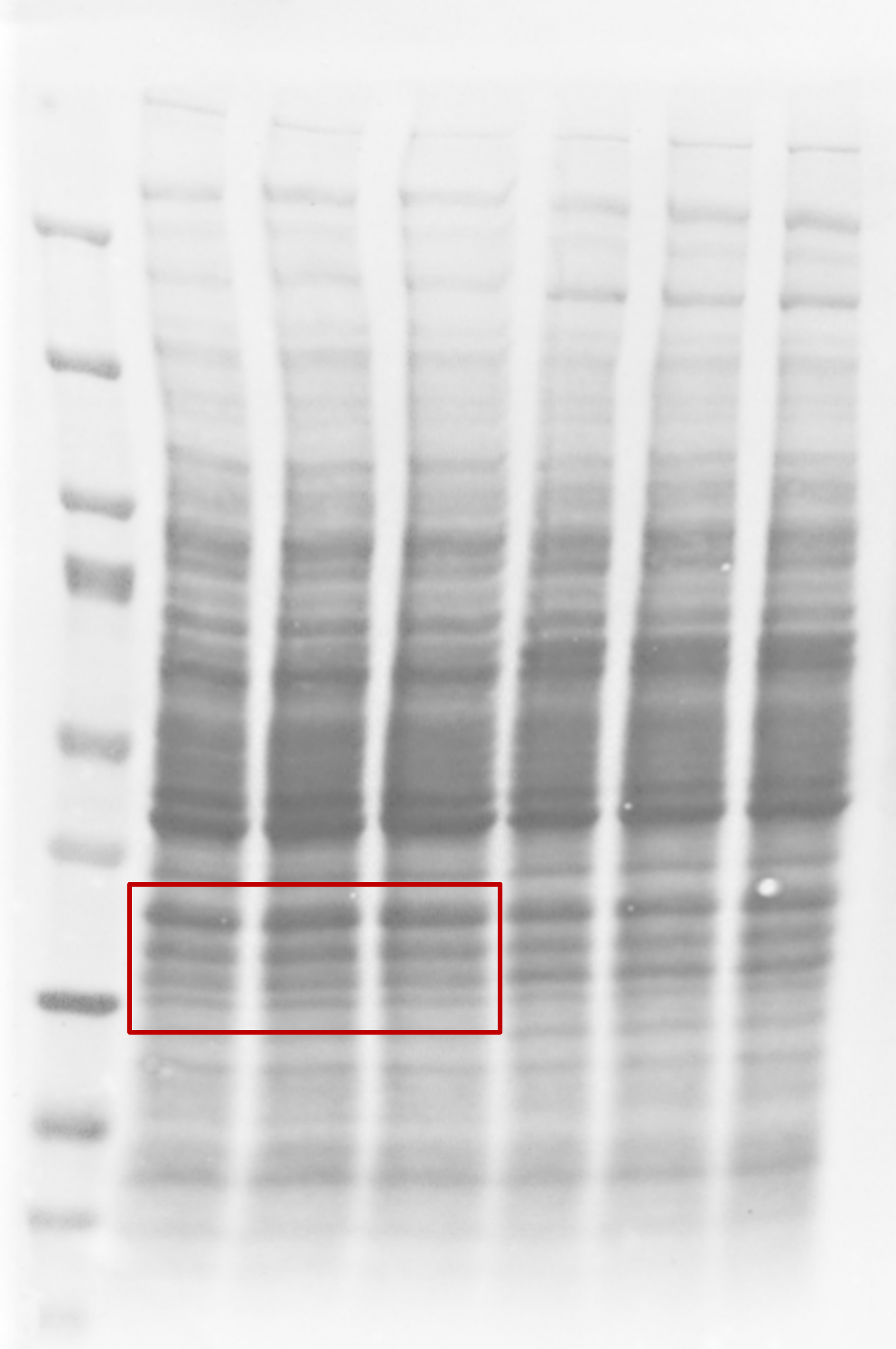

Supplement: Supplementary file 13 — Source data Fig. 6 [file 44321_2026_411_MOESM13_ESM.zip › Figure 6/6C/western Soluble Ponceau 2.tif]

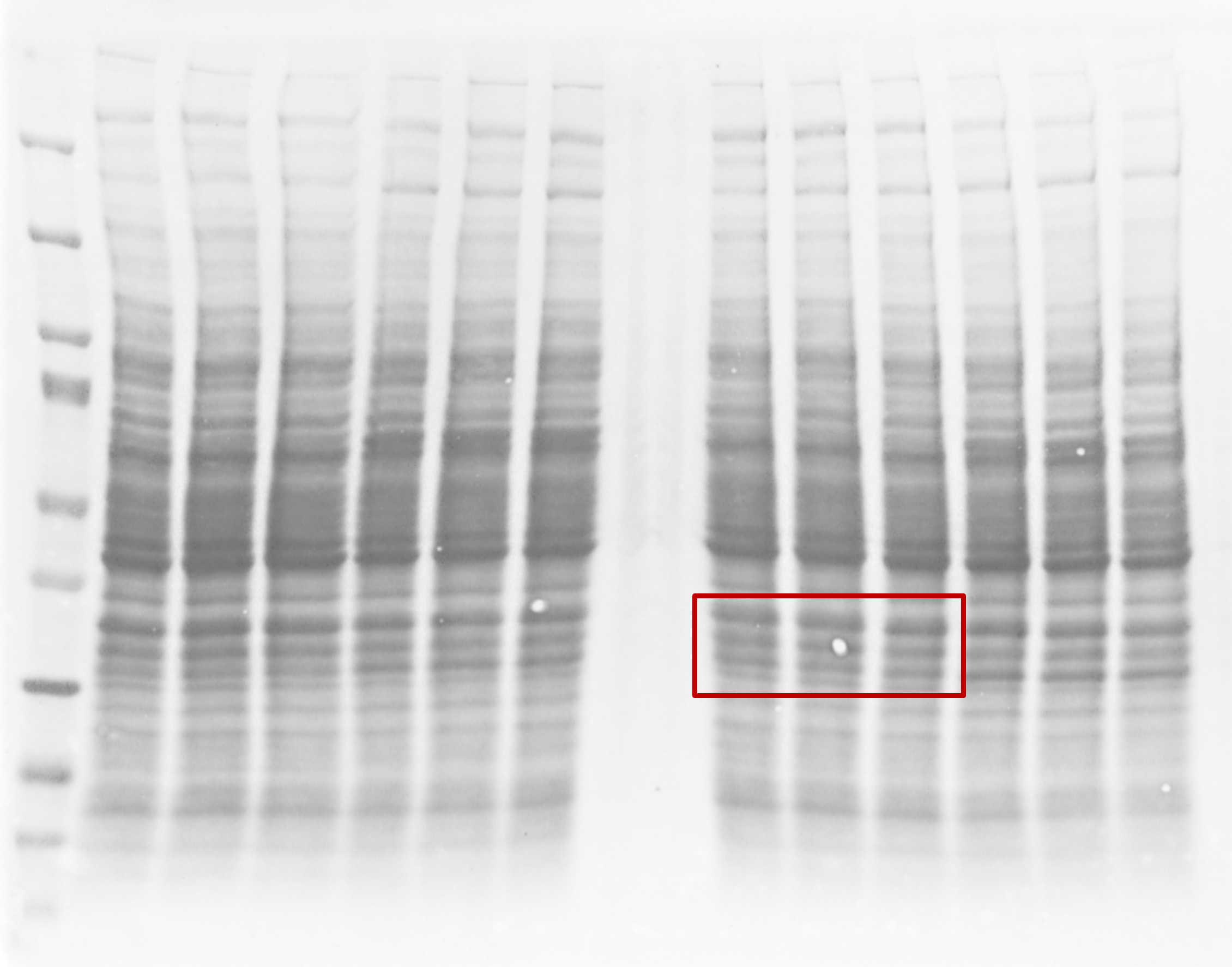

Supplement: Supplementary file 13 — Source data Fig. 6 [file 44321_2026_411_MOESM13_ESM.zip › Figure 6/6C/western Soluble Ponceau 3.tif]

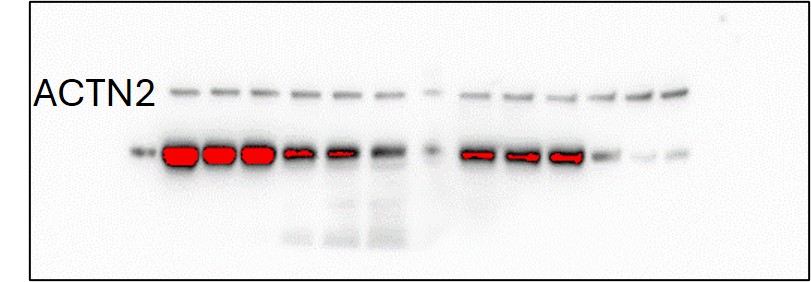

Supplement: Supplementary file 13 — Source data Fig. 6 [file 44321_2026_411_MOESM13_ESM.zip › Figure 6/6D/6D Western Insoluble ACTN2.jpg]

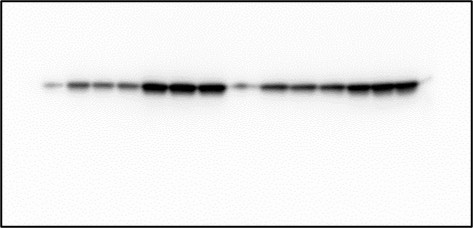

Supplement: Supplementary file 13 — Source data Fig. 6 [file 44321_2026_411_MOESM13_ESM.zip › Figure 6/6D/6D Western Insoluble CRYAB.jpg]

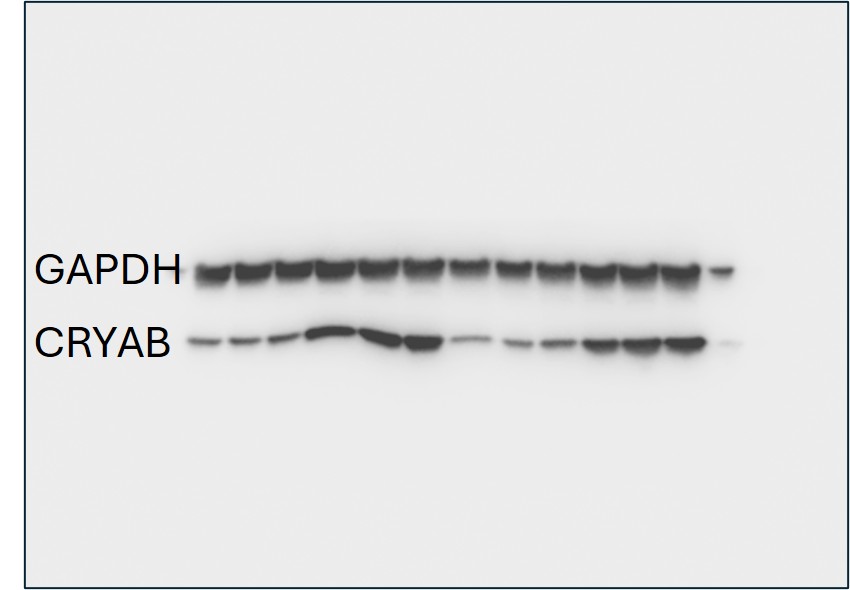

Supplement: Supplementary file 13 — Source data Fig. 6 [file 44321_2026_411_MOESM13_ESM.zip › Figure 6/6D/6D Western Soluble CRYAB GAPDH.jpg]

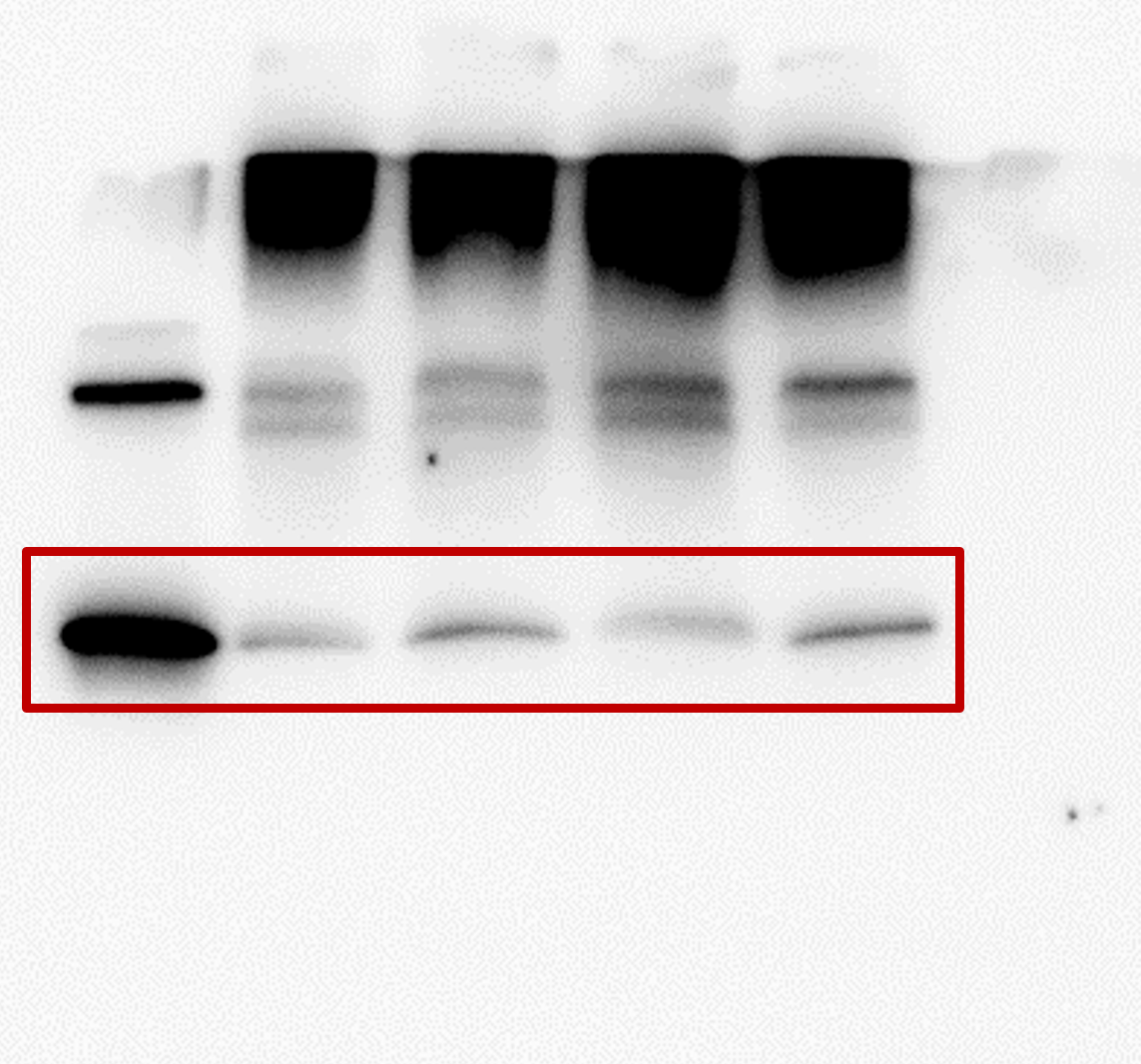

Supplement: Supplementary file 13 — Source data Fig. 6 [file 44321_2026_411_MOESM13_ESM.zip › Figure 6/6E/western CRYAB 1.tif]

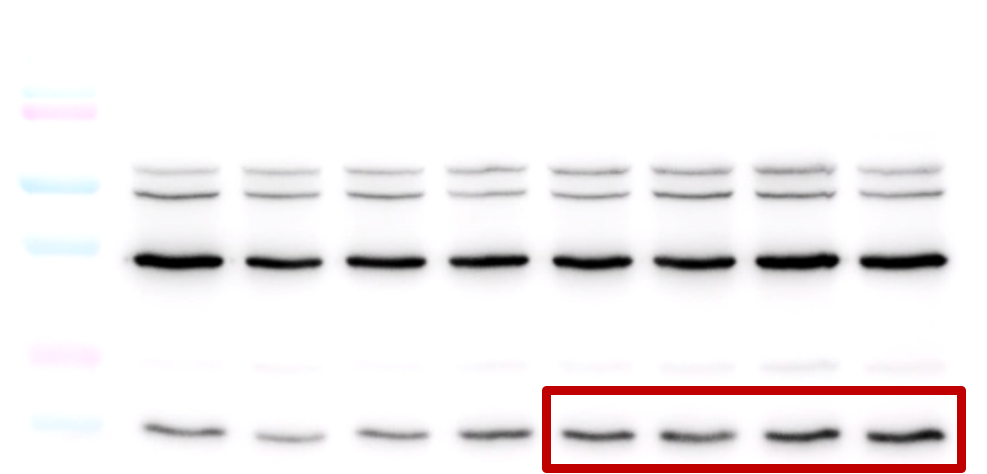

Supplement: Supplementary file 13 — Source data Fig. 6 [file 44321_2026_411_MOESM13_ESM.zip › Figure 6/6E/western CRYAB 2.tif]

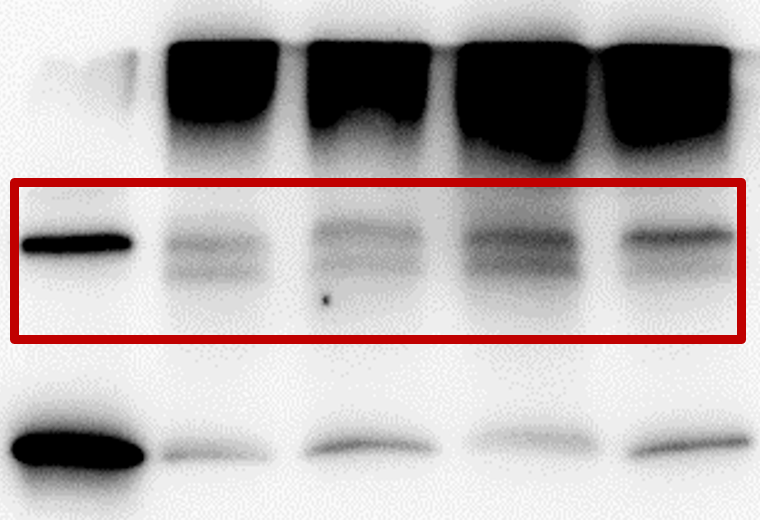

Supplement: Supplementary file 13 — Source data Fig. 6 [file 44321_2026_411_MOESM13_ESM.zip › Figure 6/6E/western CRYAB-GFP 1.tif]

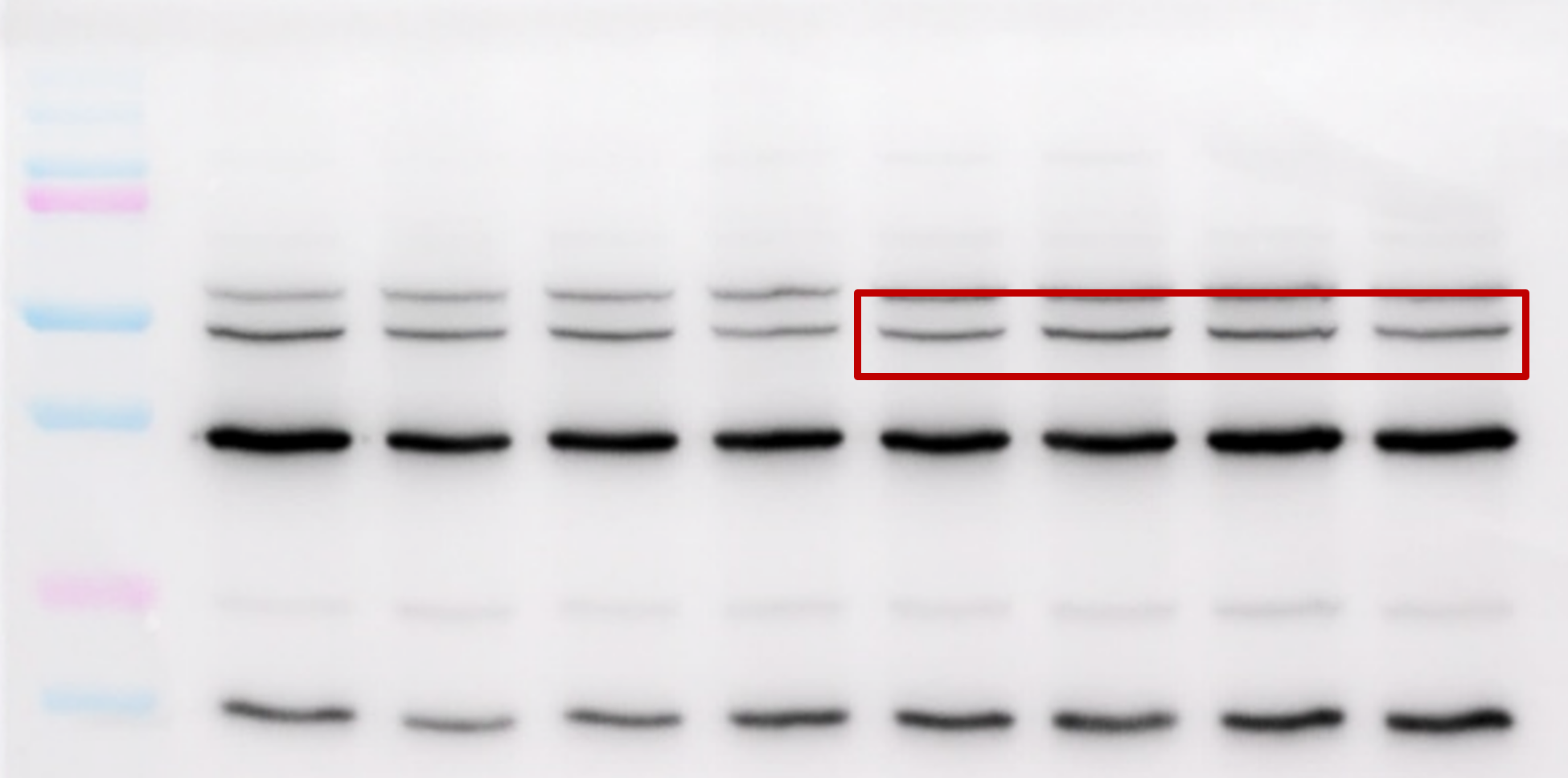

Supplement: Supplementary file 13 — Source data Fig. 6 [file 44321_2026_411_MOESM13_ESM.zip › Figure 6/6E/western CRYAB-GFP 2.tif]

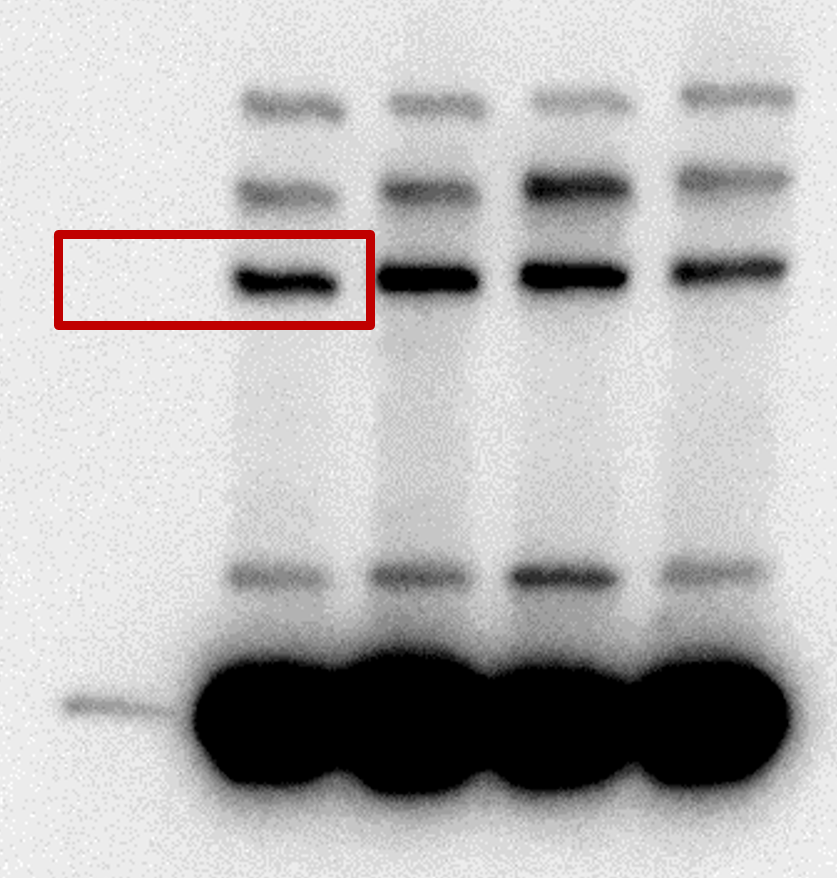

Supplement: Supplementary file 13 — Source data Fig. 6 [file 44321_2026_411_MOESM13_ESM.zip › Figure 6/6E/western CRYAB-GFP.tif]

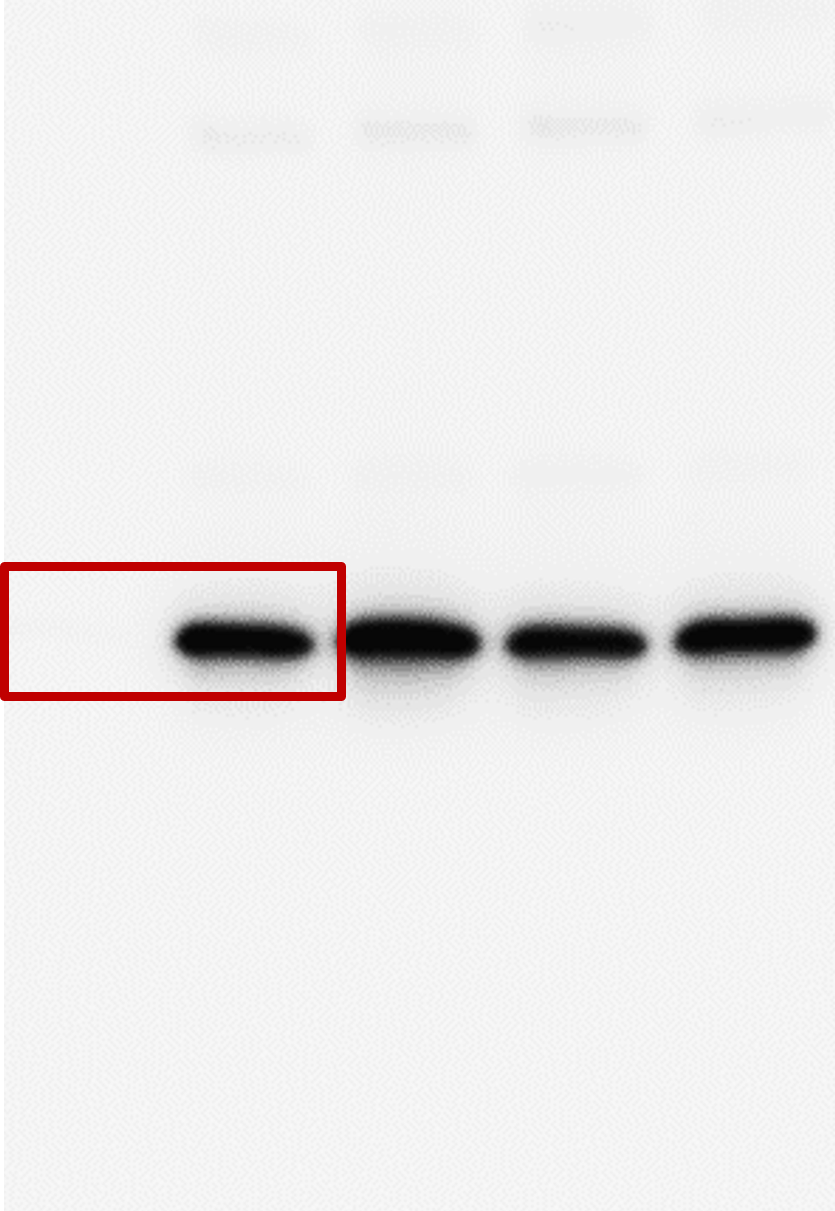

Supplement: Supplementary file 13 — Source data Fig. 6 [file 44321_2026_411_MOESM13_ESM.zip › Figure 6/6E/western CRYAB.tif]

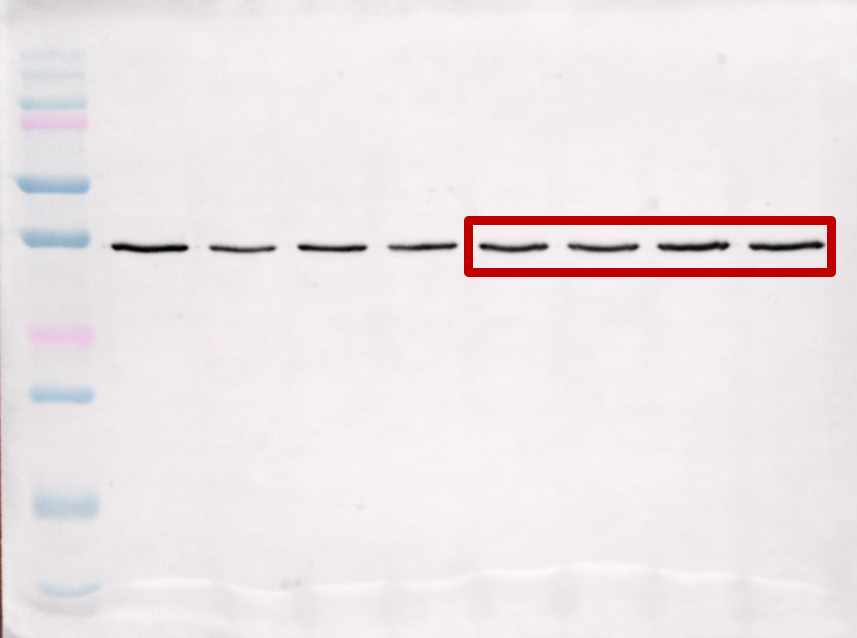

Supplement: Supplementary file 13 — Source data Fig. 6 [file 44321_2026_411_MOESM13_ESM.zip › Figure 6/6E/western GAPDH.tif]

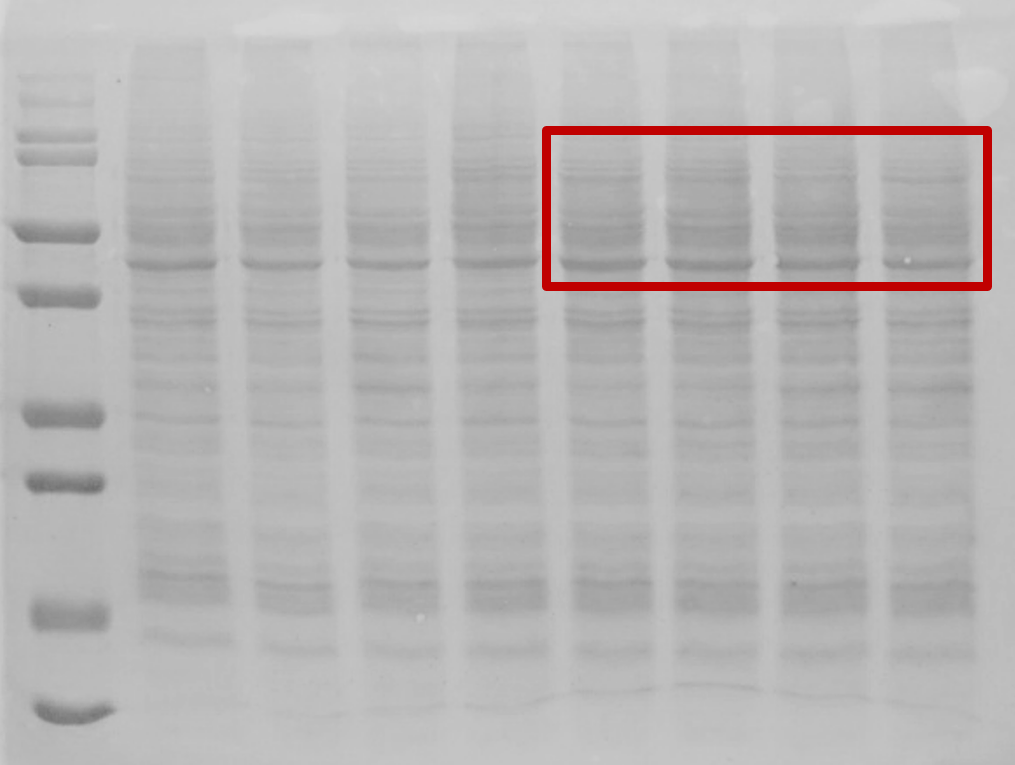

Supplement: Supplementary file 13 — Source data Fig. 6 [file 44321_2026_411_MOESM13_ESM.zip › Figure 6/6E/western Ponceau.tif]

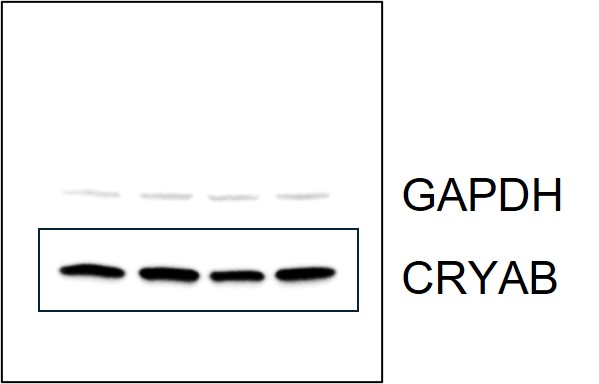

Supplement: Supplementary file 13 — Source data Fig. 6 [file 44321_2026_411_MOESM13_ESM.zip › Figure 6/6F/6F Input CRYAB.jpg]

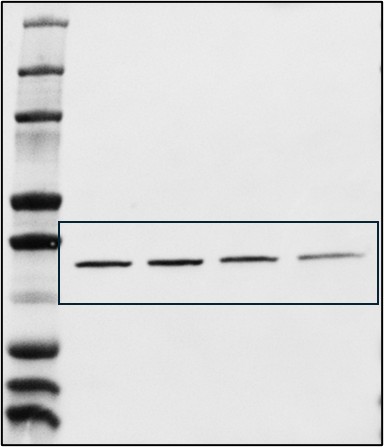

Supplement: Supplementary file 13 — Source data Fig. 6 [file 44321_2026_411_MOESM13_ESM.zip › Figure 6/6F/6F Input GAPDH 1.jpg]

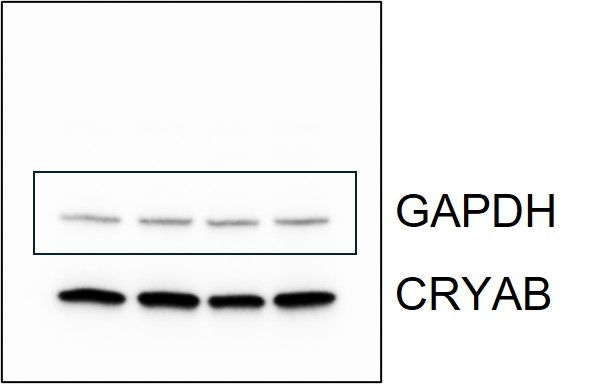

Supplement: Supplementary file 13 — Source data Fig. 6 [file 44321_2026_411_MOESM13_ESM.zip › Figure 6/6F/6F Input GAPDH.jpg]

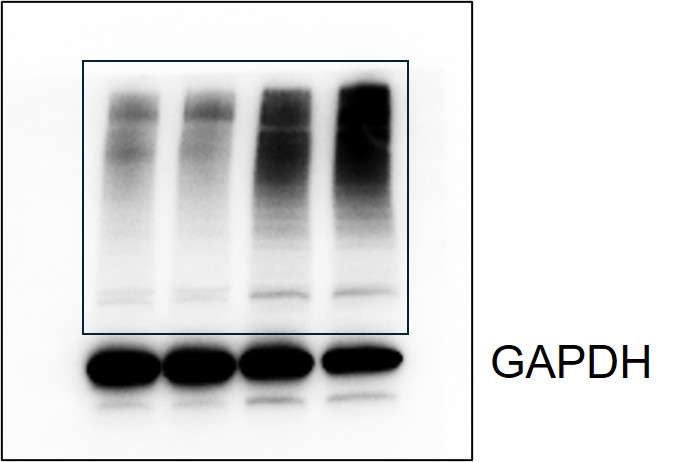

Supplement: Supplementary file 13 — Source data Fig. 6 [file 44321_2026_411_MOESM13_ESM.zip › Figure 6/6F/6F Input Ubiquitin.jpg]

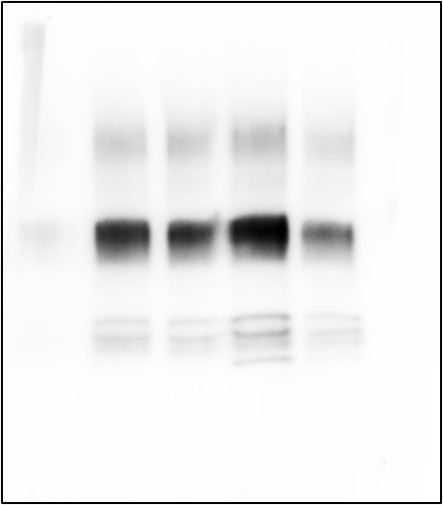

Supplement: Supplementary file 13 — Source data Fig. 6 [file 44321_2026_411_MOESM13_ESM.zip › Figure 6/6F/6F IP CRYAB-Ub.jpg]

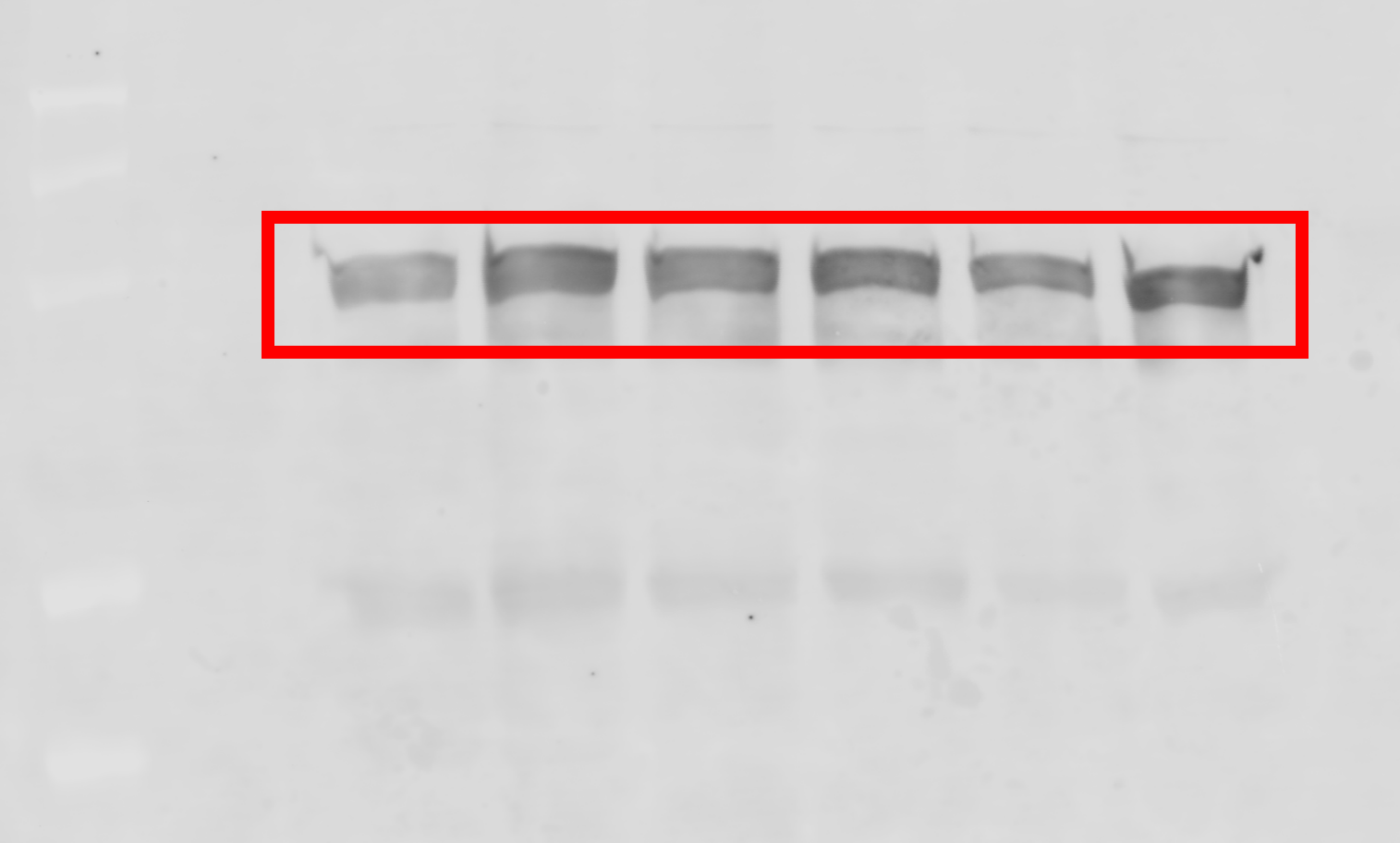

Supplement: Supplementary file 14 — Source data Fig. 7 [file 44321_2026_411_MOESM14_ESM.zip › Figure 7/7C/western ACTN2 7 months.tif]

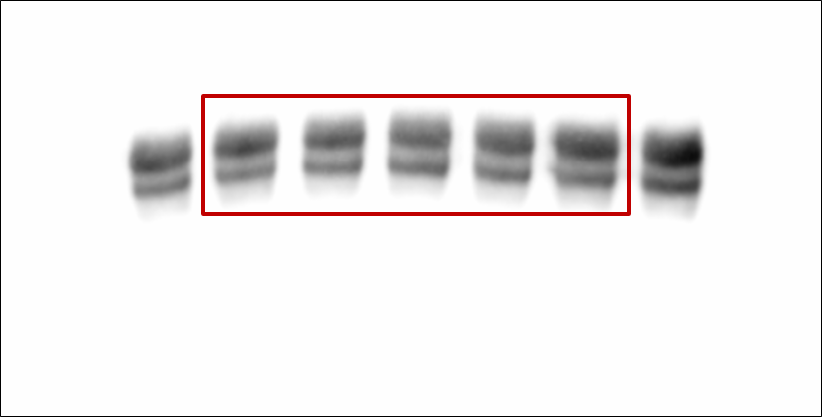

Supplement: Supplementary file 14 — Source data Fig. 7 [file 44321_2026_411_MOESM14_ESM.zip › Figure 7/7C/western GAPDH 1 month.tif]

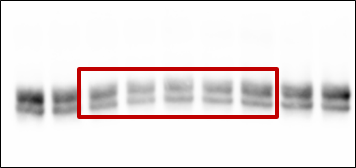

Supplement: Supplementary file 14 — Source data Fig. 7 [file 44321_2026_411_MOESM14_ESM.zip › Figure 7/7C/western GAPDH 4 months.tif]

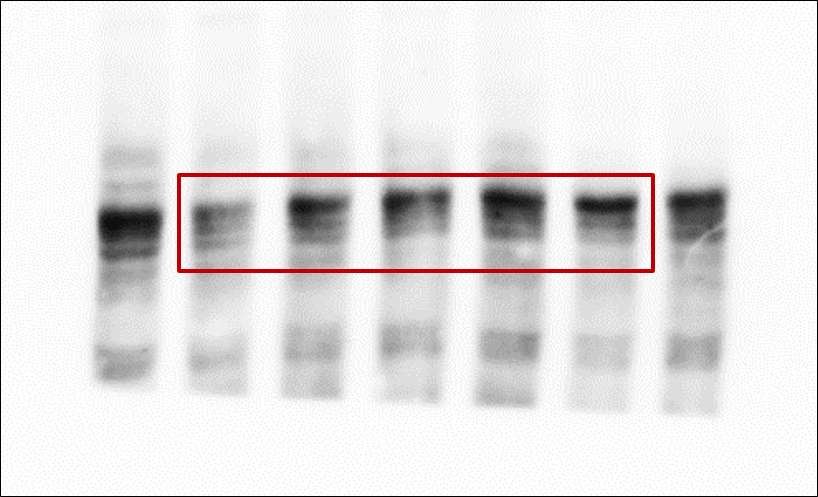

Supplement: Supplementary file 14 — Source data Fig. 7 [file 44321_2026_411_MOESM14_ESM.zip › Figure 7/7C/western P-STAT3 1 month.tif]

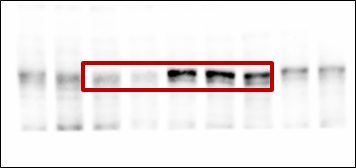

Supplement: Supplementary file 14 — Source data Fig. 7 [file 44321_2026_411_MOESM14_ESM.zip › Figure 7/7C/western P-STAT3 4 months.tif]

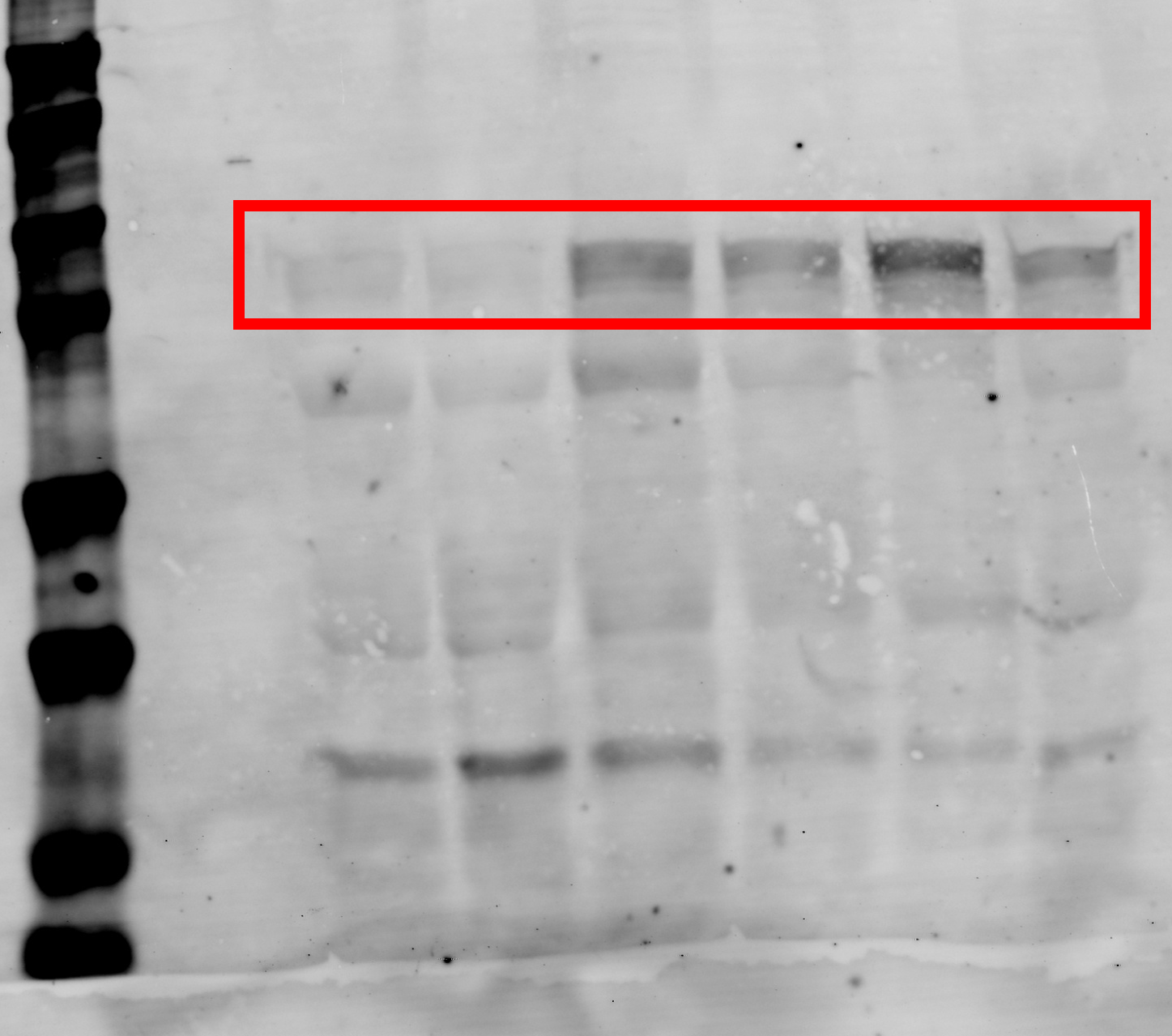

Supplement: Supplementary file 14 — Source data Fig. 7 [file 44321_2026_411_MOESM14_ESM.zip › Figure 7/7C/western P-STAT3 7 months.tif]

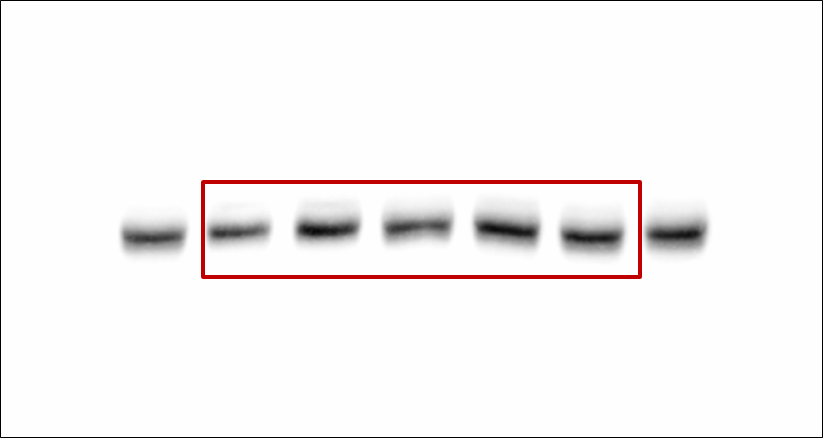

Supplement: Supplementary file 14 — Source data Fig. 7 [file 44321_2026_411_MOESM14_ESM.zip › Figure 7/7C/western STAT3 1 month.tif]

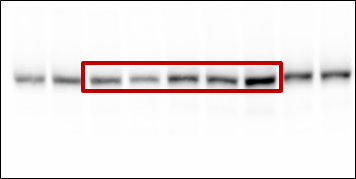

Supplement: Supplementary file 14 — Source data Fig. 7 [file 44321_2026_411_MOESM14_ESM.zip › Figure 7/7C/western STAT3 4 months.tif]

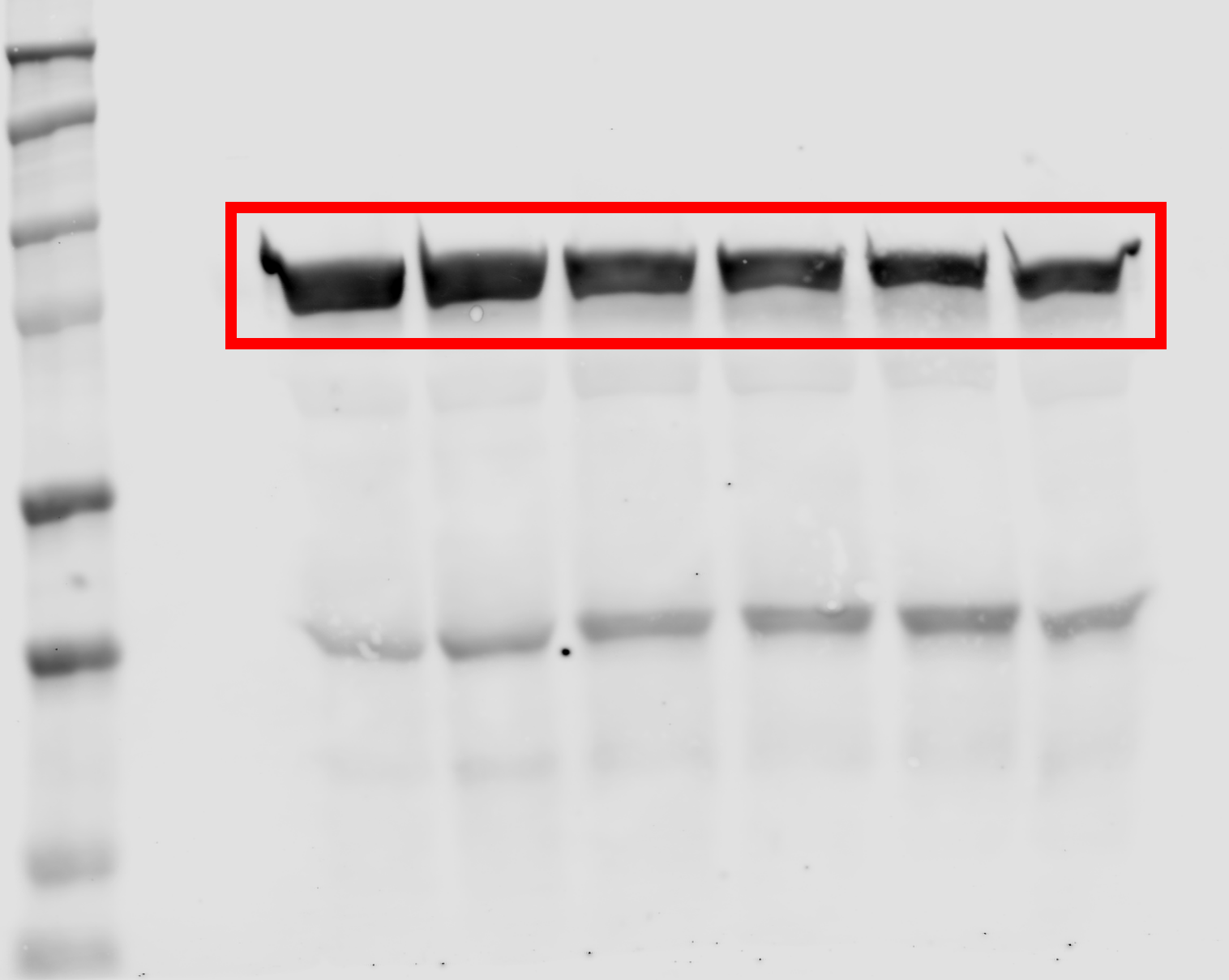

Supplement: Supplementary file 14 — Source data Fig. 7 [file 44321_2026_411_MOESM14_ESM.zip › Figure 7/7C/western STAT3 7 months.tif]

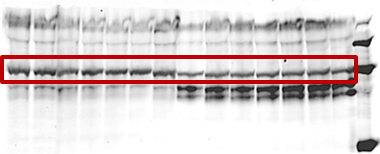

Supplement: Supplementary file 14 — Source data Fig. 7 [file 44321_2026_411_MOESM14_ESM.zip › Figure 7/7F/western Insoluble ACTN2 1.tif]

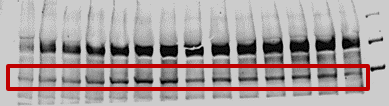

Supplement: Supplementary file 14 — Source data Fig. 7 [file 44321_2026_411_MOESM14_ESM.zip › Figure 7/7F/western Insoluble ACTN2.tif]

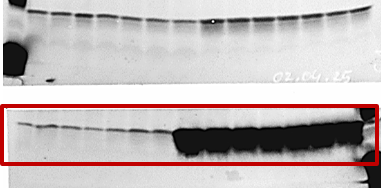

Supplement: Supplementary file 14 — Source data Fig. 7 [file 44321_2026_411_MOESM14_ESM.zip › Figure 7/7F/western Insoluble CRYAB.tif]

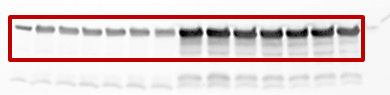

Supplement: Supplementary file 14 — Source data Fig. 7 [file 44321_2026_411_MOESM14_ESM.zip › Figure 7/7F/western Insoluble Desmin.tif]

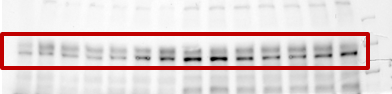

Supplement: Supplementary file 14 — Source data Fig. 7 [file 44321_2026_411_MOESM14_ESM.zip › Figure 7/7F/western Insoluble JAK1.tif]

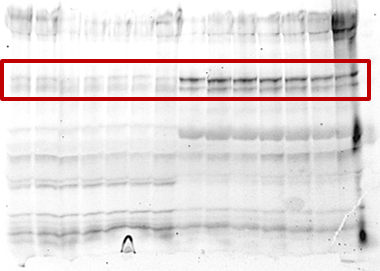

Supplement: Supplementary file 14 — Source data Fig. 7 [file 44321_2026_411_MOESM14_ESM.zip › Figure 7/7F/western Insoluble P-STAT3.tif]

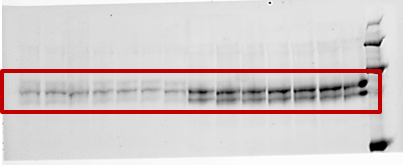

Supplement: Supplementary file 14 — Source data Fig. 7 [file 44321_2026_411_MOESM14_ESM.zip › Figure 7/7F/western Insoluble STAT3.tif]

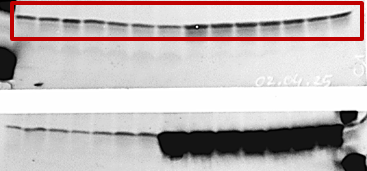

Supplement: Supplementary file 14 — Source data Fig. 7 [file 44321_2026_411_MOESM14_ESM.zip › Figure 7/7F/western Soluble CRYAB.tif]

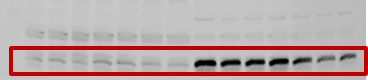

Supplement: Supplementary file 14 — Source data Fig. 7 [file 44321_2026_411_MOESM14_ESM.zip › Figure 7/7F/western Soluble Desmin.tif]

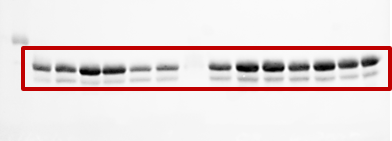

Supplement: Supplementary file 14 — Source data Fig. 7 [file 44321_2026_411_MOESM14_ESM.zip › Figure 7/7F/western Soluble GAPDH 1.tif]

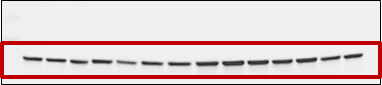

Supplement: Supplementary file 14 — Source data Fig. 7 [file 44321_2026_411_MOESM14_ESM.zip › Figure 7/7F/western Soluble GAPDH.tif]

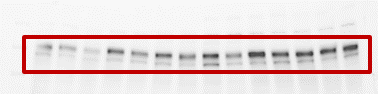

Supplement: Supplementary file 14 — Source data Fig. 7 [file 44321_2026_411_MOESM14_ESM.zip › Figure 7/7F/western Soluble JAK1.tif]

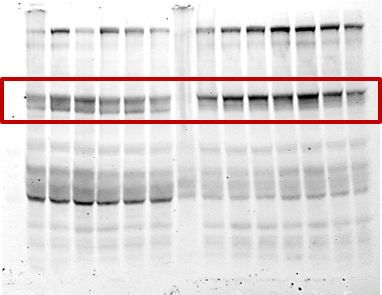

Supplement: Supplementary file 14 — Source data Fig. 7 [file 44321_2026_411_MOESM14_ESM.zip › Figure 7/7F/western Soluble P-STAT3.tif]

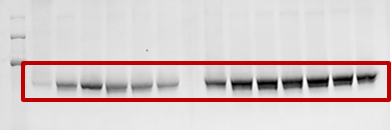

Supplement: Supplementary file 14 — Source data Fig. 7 [file 44321_2026_411_MOESM14_ESM.zip › Figure 7/7F/western Soluble STAT3.tif]

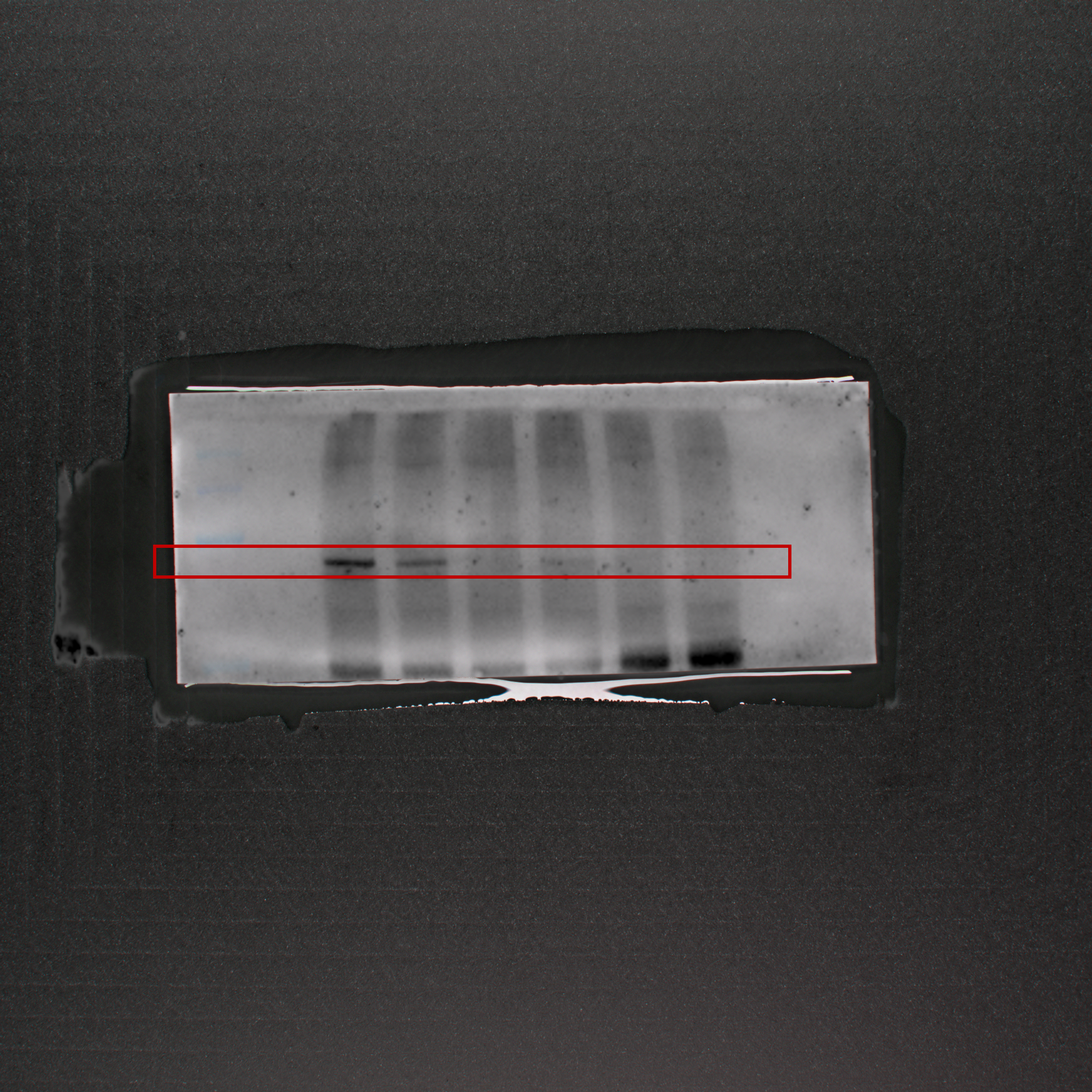

Supplement: Supplementary file 15 — Source data Fig. 8 [file 44321_2026_411_MOESM15_ESM.zip › Figure 8/8A/p-STAT3.tif]

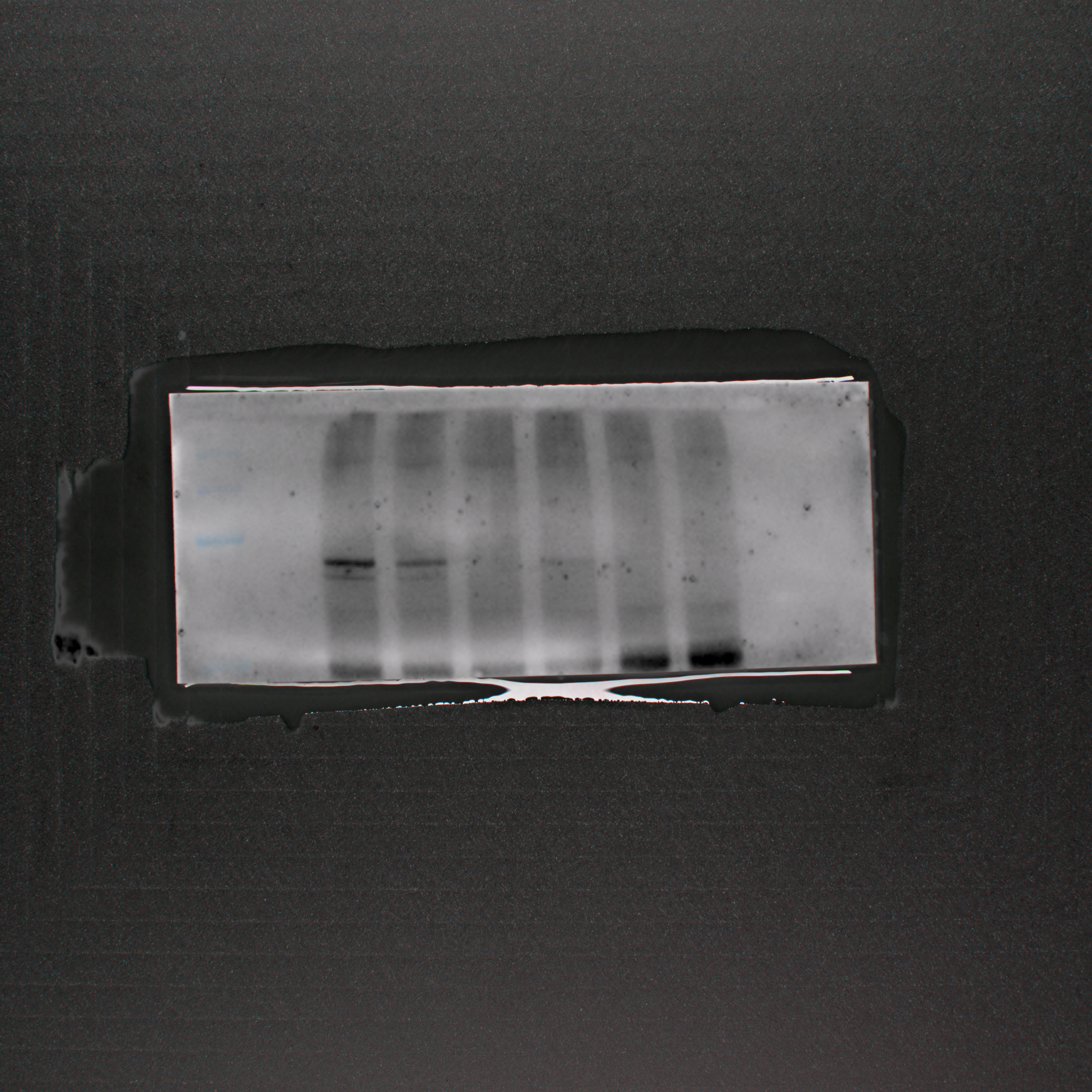

Supplement: Supplementary file 15 — Source data Fig. 8 [file 44321_2026_411_MOESM15_ESM.zip › Figure 8/8A/pSTAT3/20_02_2025_18_54_41.Tif]

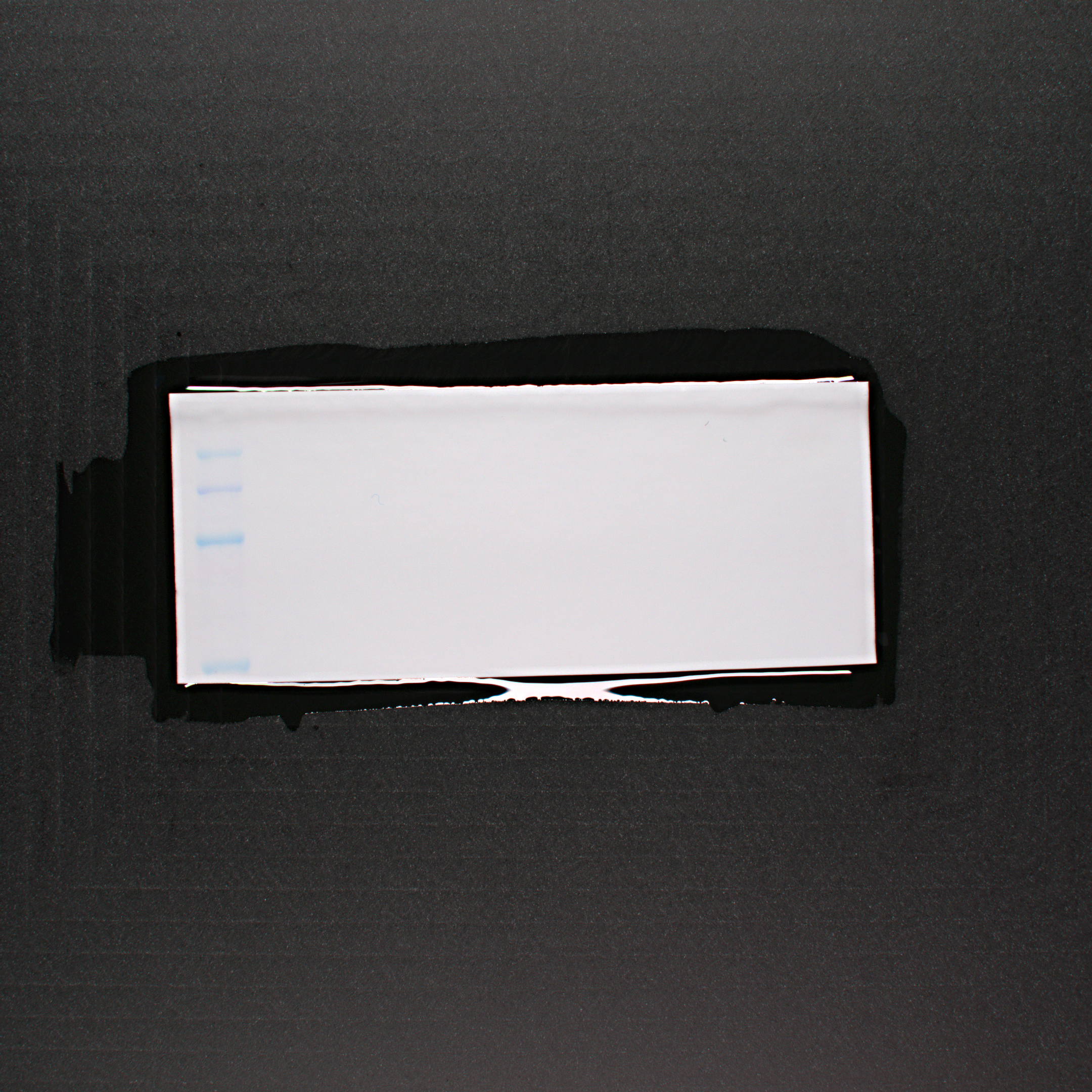

Supplement: Supplementary file 15 — Source data Fig. 8 [file 44321_2026_411_MOESM15_ESM.zip › Figure 8/8A/pSTAT3/20_02_2025_18_54_47.Tif]

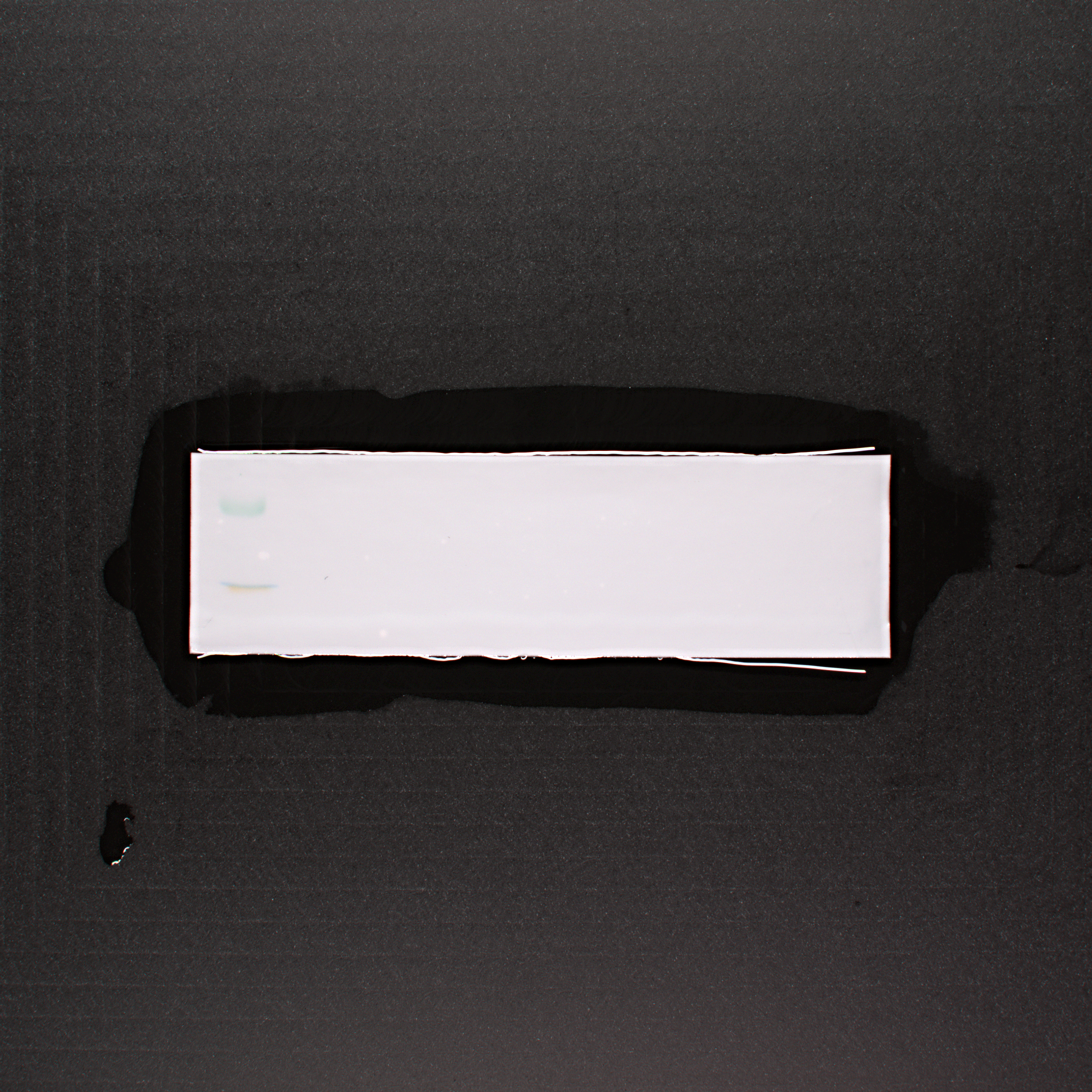

Supplement: Supplementary file 15 — Source data Fig. 8 [file 44321_2026_411_MOESM15_ESM.zip › Figure 8/8A/S6/20_02_2025_18_54_34.Tif]

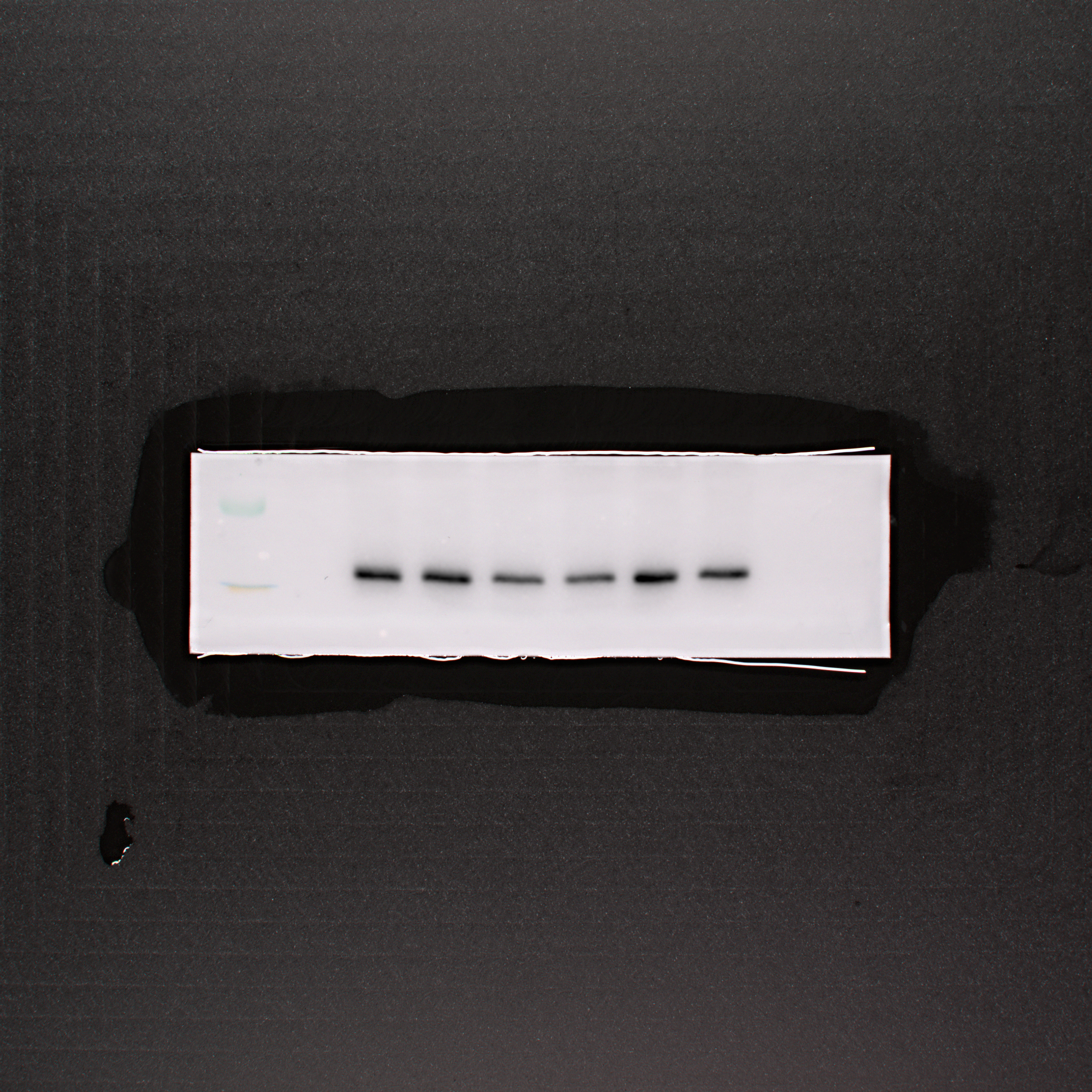

Supplement: Supplementary file 15 — Source data Fig. 8 [file 44321_2026_411_MOESM15_ESM.zip › Figure 8/8A/S6/Merge.Tif]

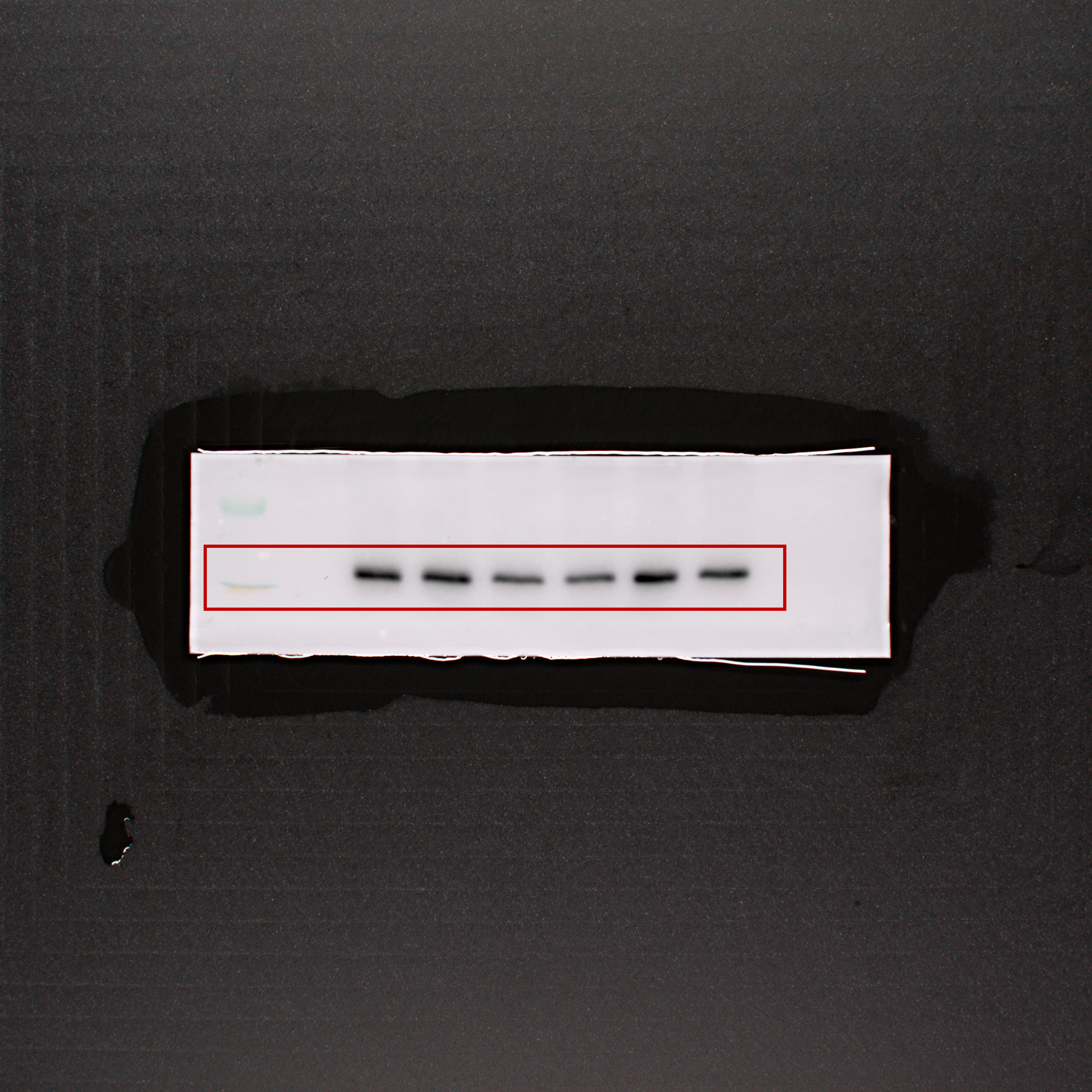

Supplement: Supplementary file 15 — Source data Fig. 8 [file 44321_2026_411_MOESM15_ESM.zip › Figure 8/8A/S6/S6.tif]

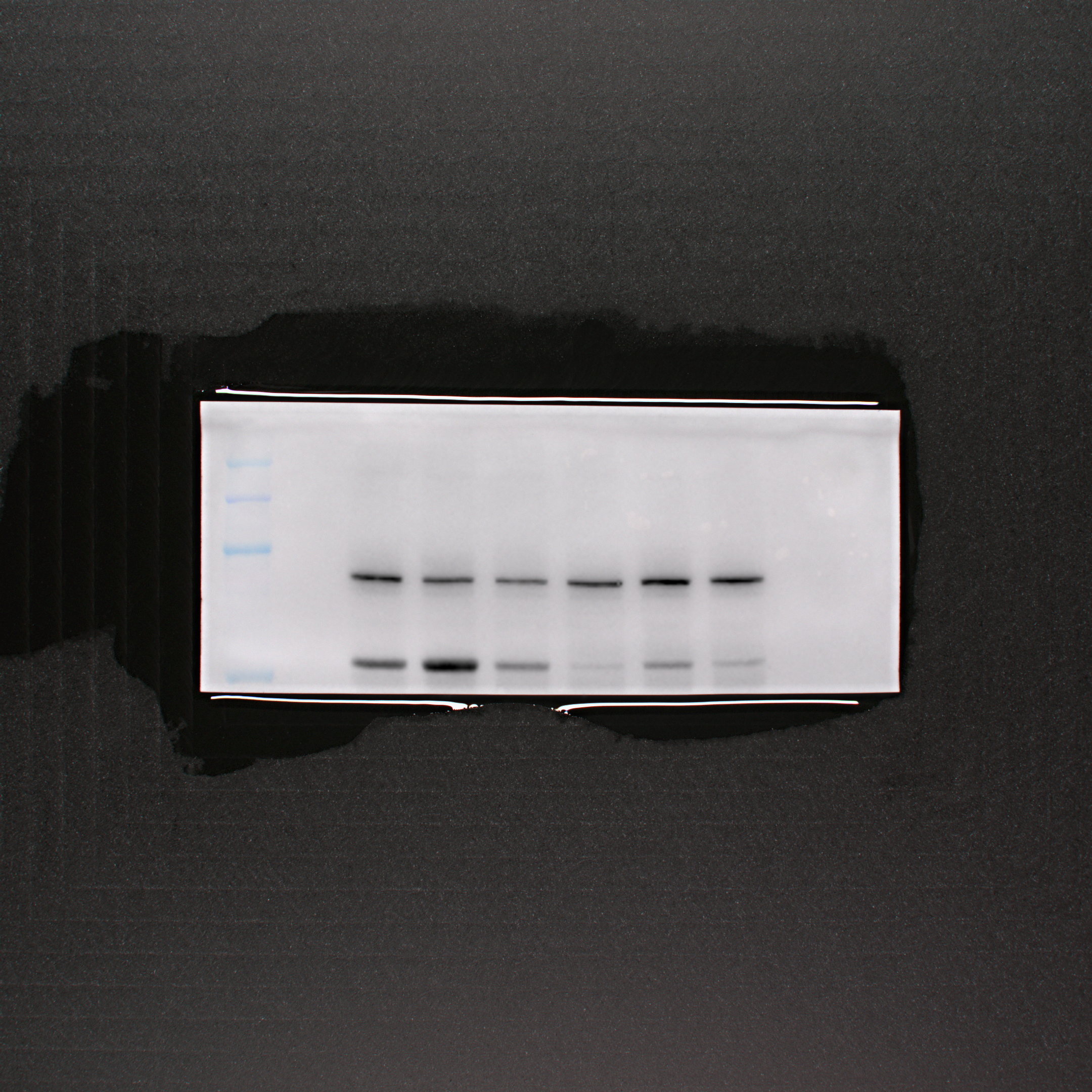

Supplement: Supplementary file 15 — Source data Fig. 8 [file 44321_2026_411_MOESM15_ESM.zip › Figure 8/8A/STAT3/24_02_2025_18_43_32.Tif]

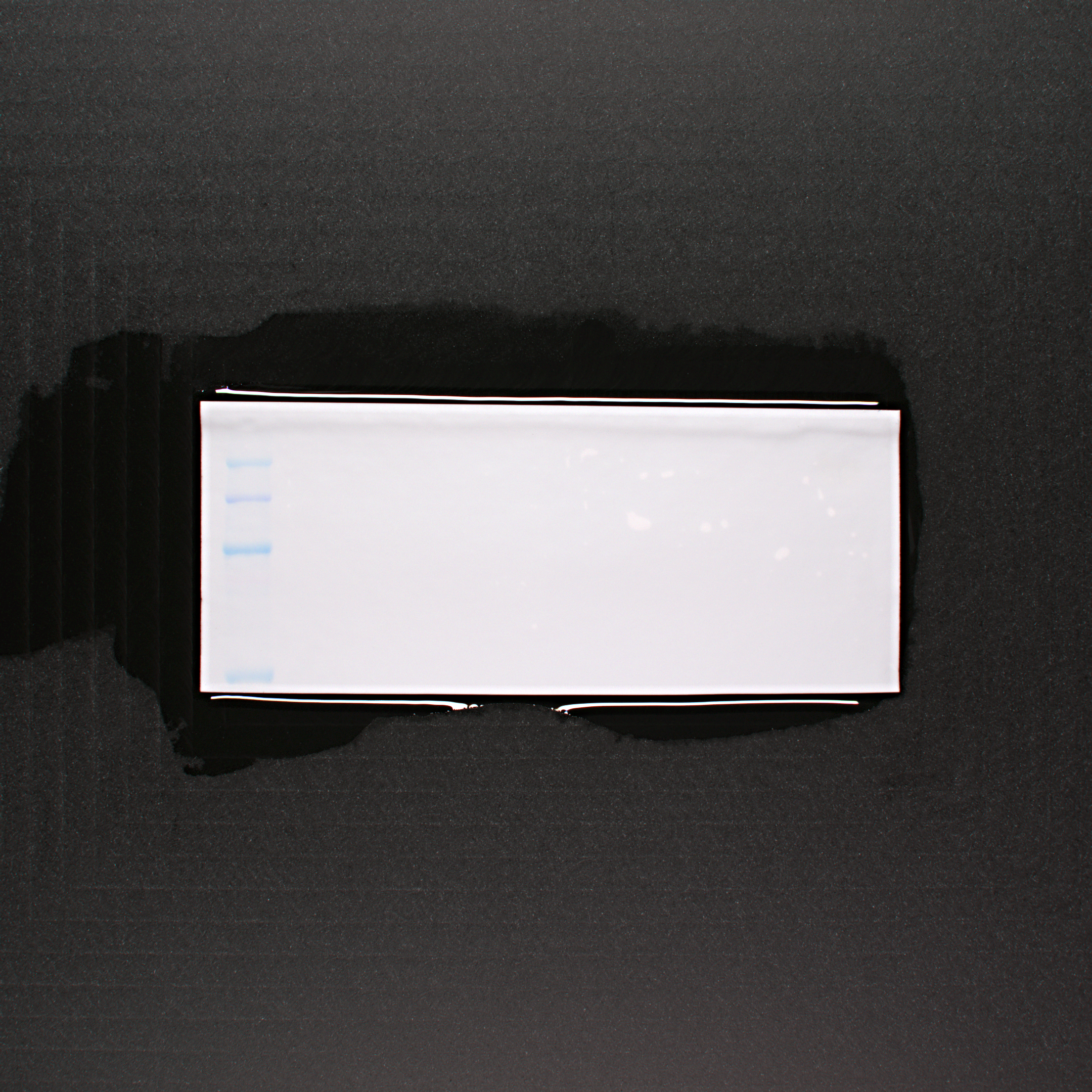

Supplement: Supplementary file 15 — Source data Fig. 8 [file 44321_2026_411_MOESM15_ESM.zip › Figure 8/8A/STAT3/24_02_2025_18_43_54.Tif]

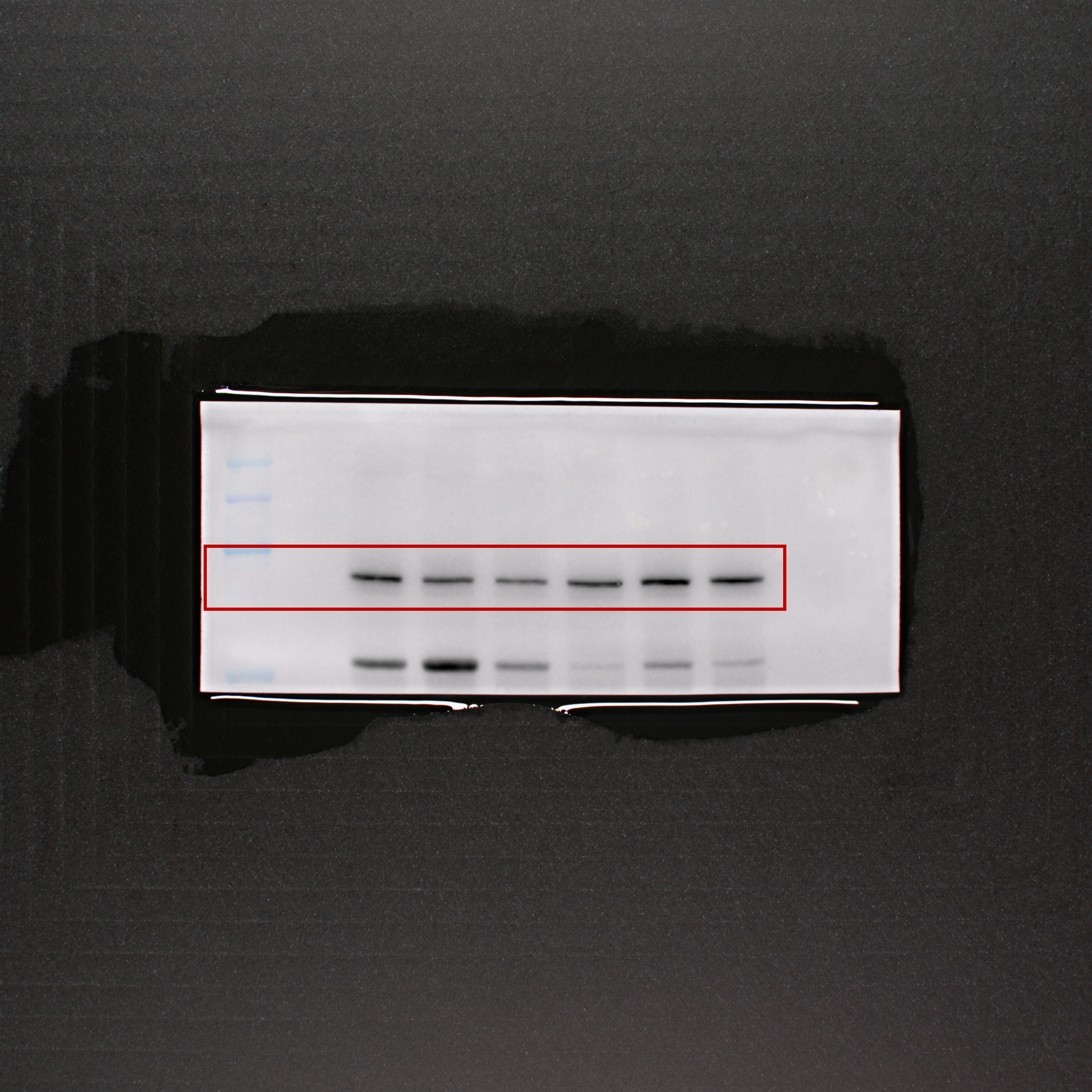

Supplement: Supplementary file 15 — Source data Fig. 8 [file 44321_2026_411_MOESM15_ESM.zip › Figure 8/8A/STAT3/STAT3.tif]

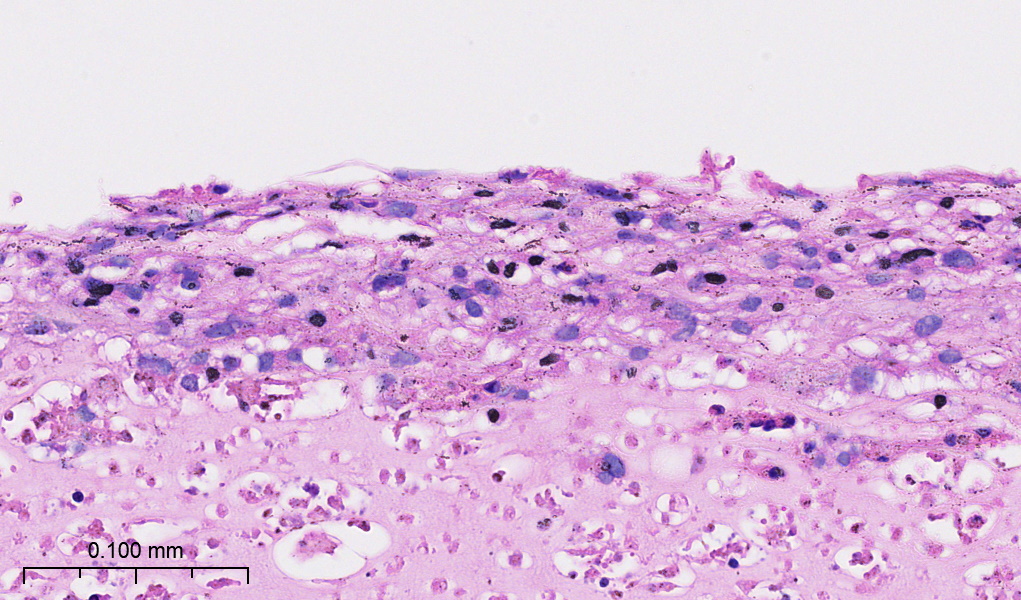

Supplement: Supplementary file 15 — Source data Fig. 8 [file 44321_2026_411_MOESM15_ESM.zip › Figure 8/8E/HE 1 (30 μM) .ndpi_14.0x.jpg]
