# Supplementary figures and images for: JAK1/2 inhibitor ruxolitinib reduces aggregates in cardiac proteinopathy (part 3 of 3)
Source: EMBO Mol Med. 2026 Mar 31;18(5):1836–65. doi: 10.1038/s44321-026-00411-x (PMC13179346; doi:10.1038/s44321-026-00411-x)

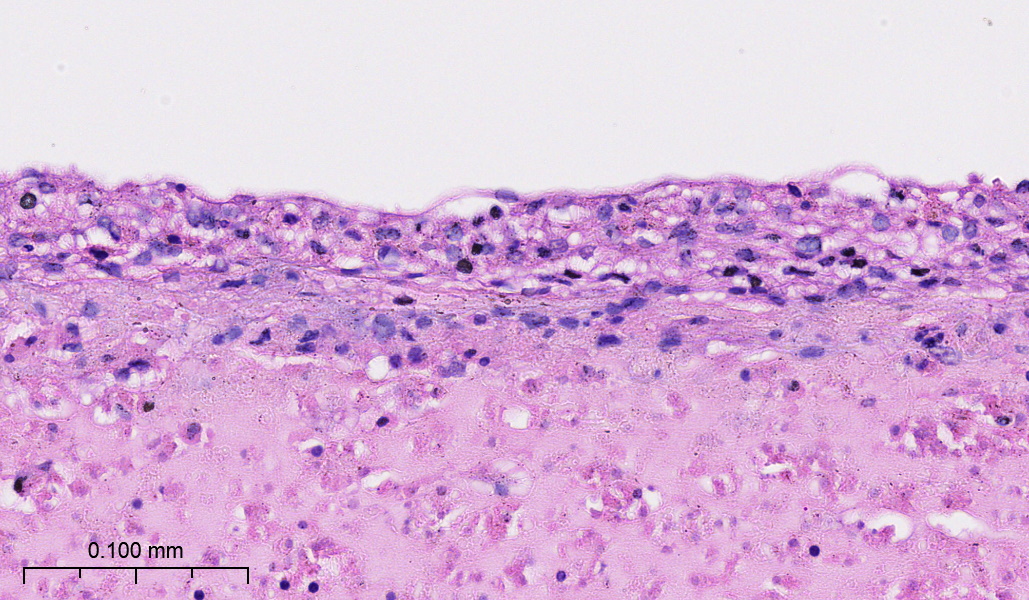

Supplement: Supplementary file 15 — Source data Fig. 8 [file 44321_2026_411_MOESM15_ESM.zip › Figure 8/8E/HE 2 (10 μM) .ndpi_14.0x.jpg]

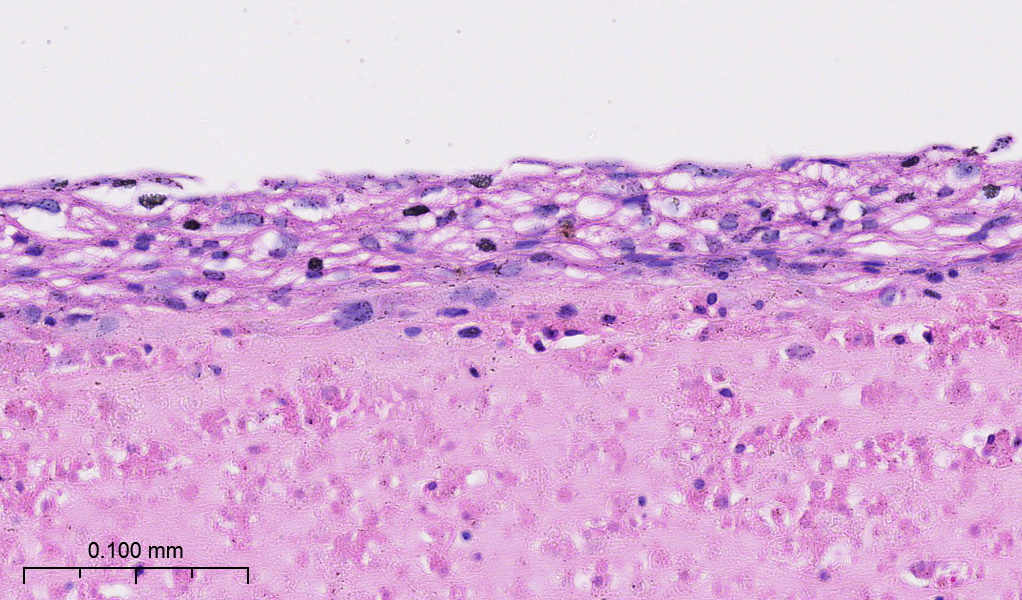

Supplement: Supplementary file 15 — Source data Fig. 8 [file 44321_2026_411_MOESM15_ESM.zip › Figure 8/8E/HE 3 (3 μM) .ndpi_14.0x.jpg]

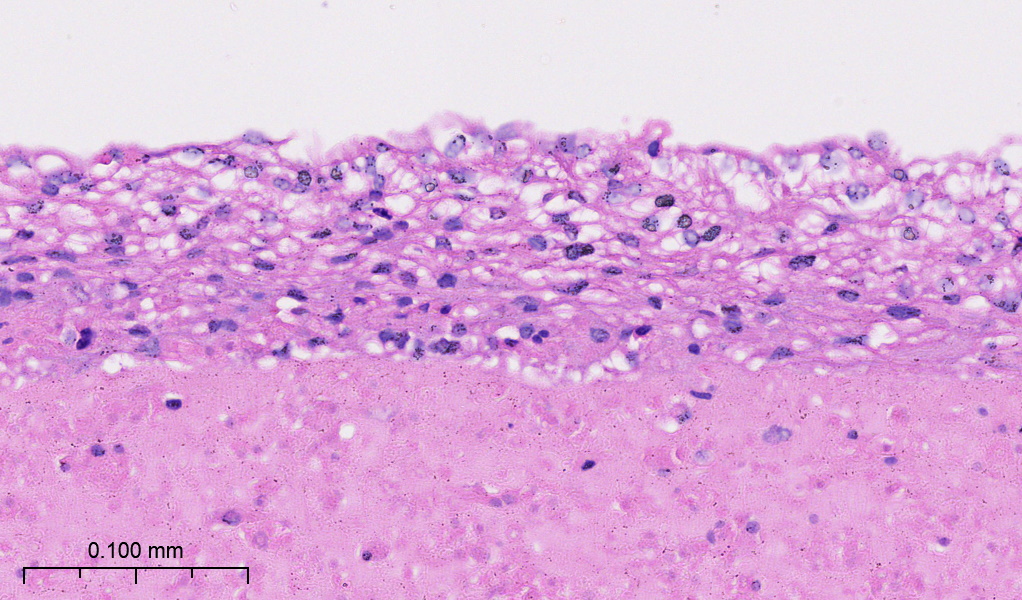

Supplement: Supplementary file 15 — Source data Fig. 8 [file 44321_2026_411_MOESM15_ESM.zip › Figure 8/8E/HE 4 (1 μM) .ndpi_14.0x.jpg]

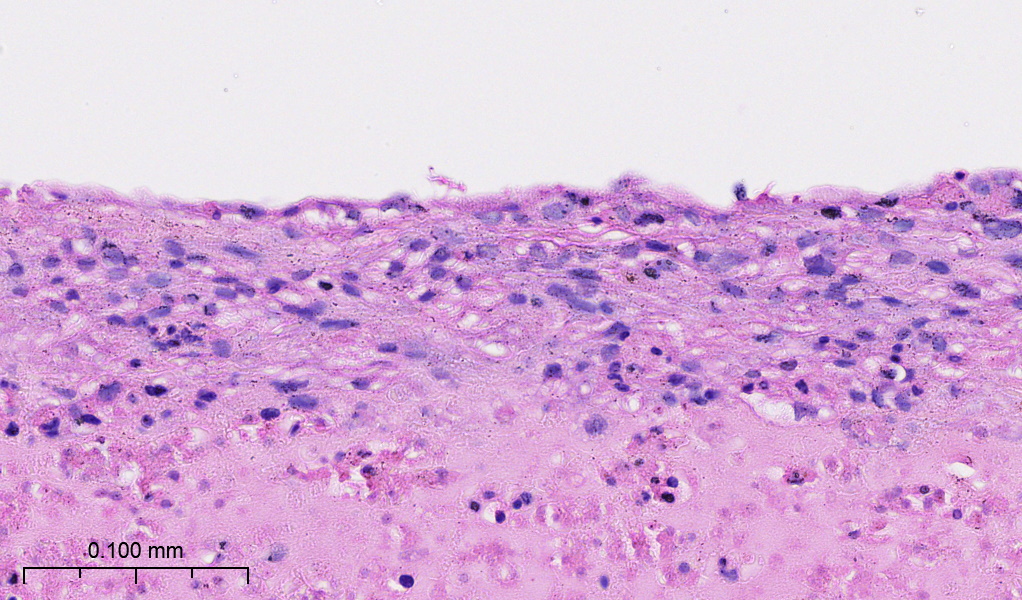

Supplement: Supplementary file 15 — Source data Fig. 8 [file 44321_2026_411_MOESM15_ESM.zip › Figure 8/8E/HE 5 (0.3 μM) .ndpi_14.0x.jpg]

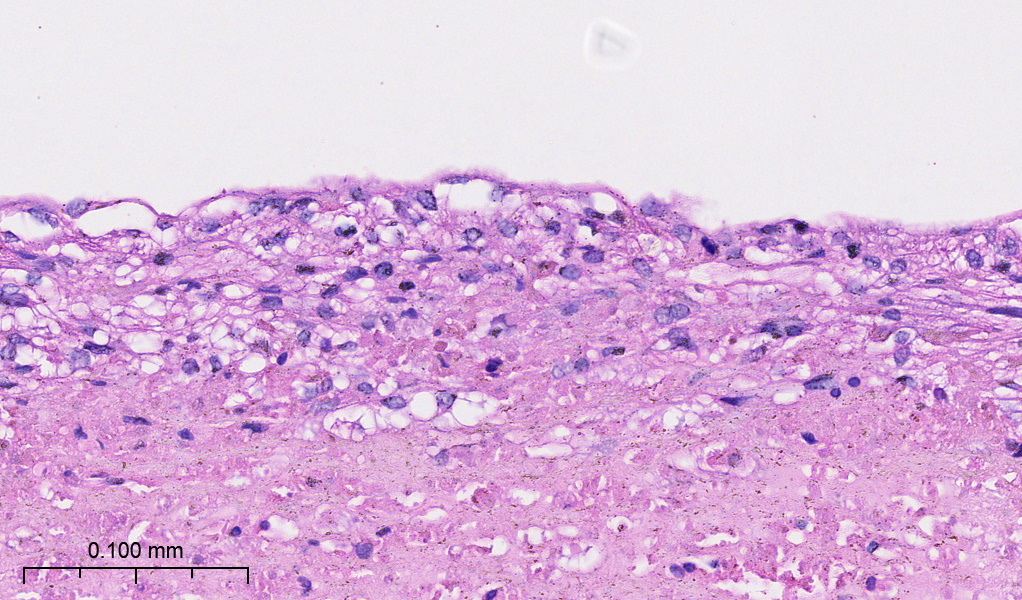

Supplement: Supplementary file 15 — Source data Fig. 8 [file 44321_2026_411_MOESM15_ESM.zip › Figure 8/8E/HE 6 (solvent DMSO) .ndpi_14.0x.jpg]

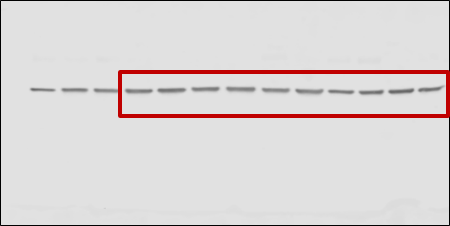

Supplement: Supplementary file 16 — Source data Fig. 9 [file 44321_2026_411_MOESM16_ESM.zip › Figure 9/9B/western GAPDH.tif]

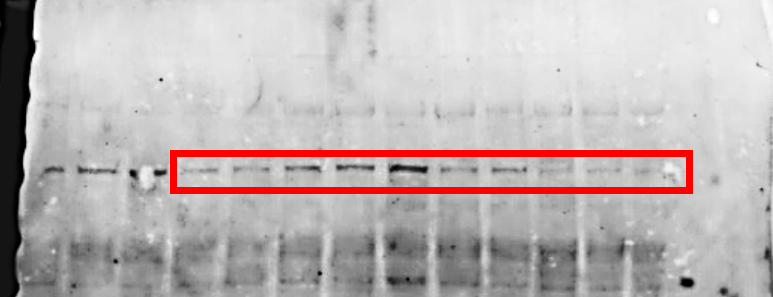

Supplement: Supplementary file 16 — Source data Fig. 9 [file 44321_2026_411_MOESM16_ESM.zip › Figure 9/9B/western P-STAT3.tif]

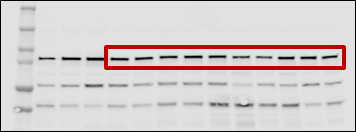

Supplement: Supplementary file 16 — Source data Fig. 9 [file 44321_2026_411_MOESM16_ESM.zip › Figure 9/9B/western STAT3.tif]

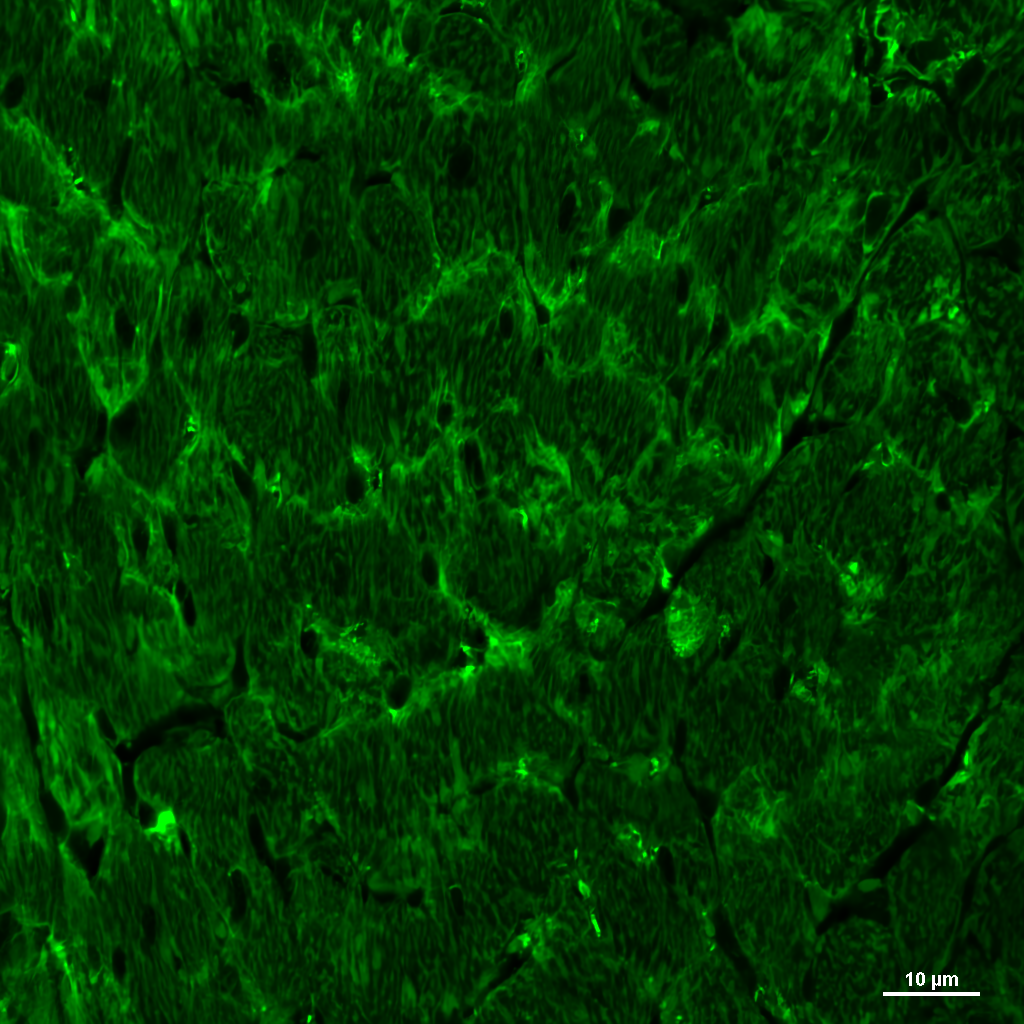

Supplement: Supplementary file 16 — Source data Fig. 9 [file 44321_2026_411_MOESM16_ESM.zip › Figure 9/9E/NTG_Veh_CRYAB.tif]

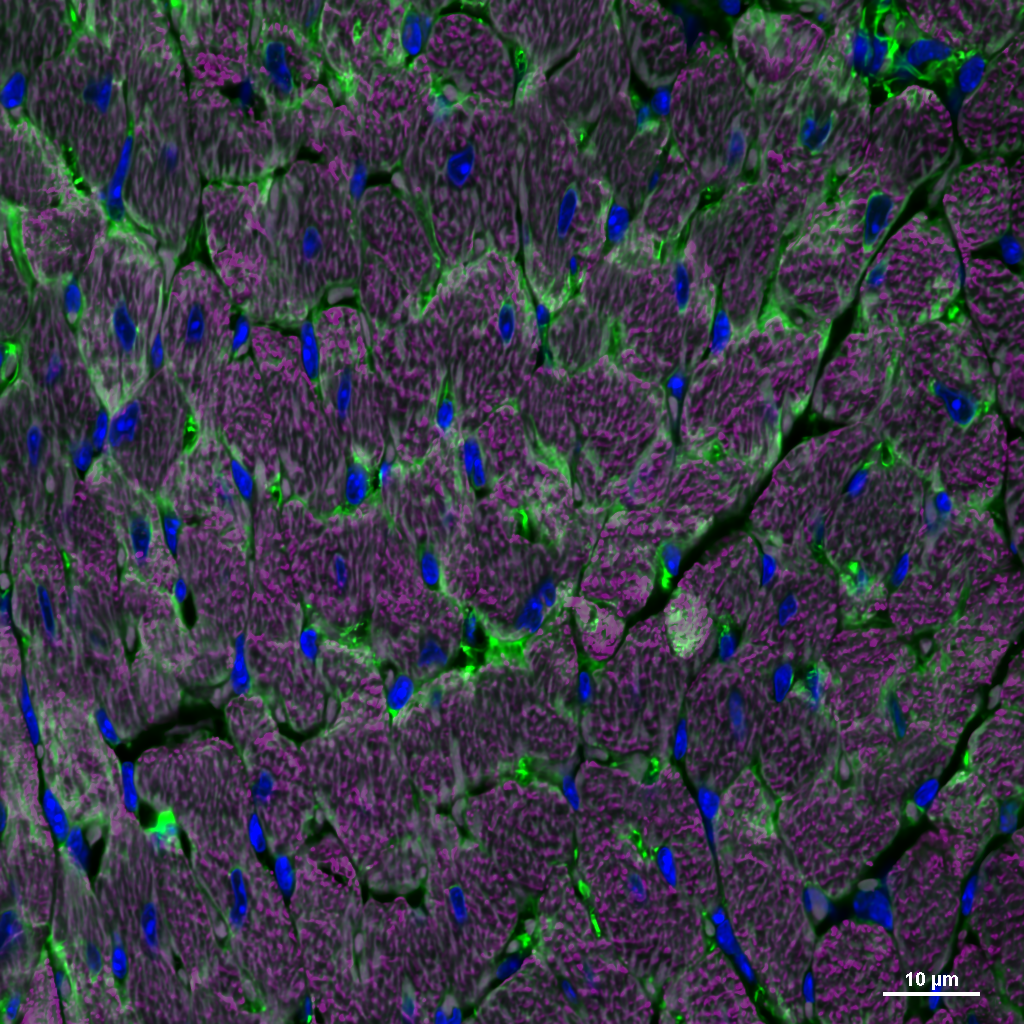

Supplement: Supplementary file 16 — Source data Fig. 9 [file 44321_2026_411_MOESM16_ESM.zip › Figure 9/9E/NTG_Veh_RGB.tif]

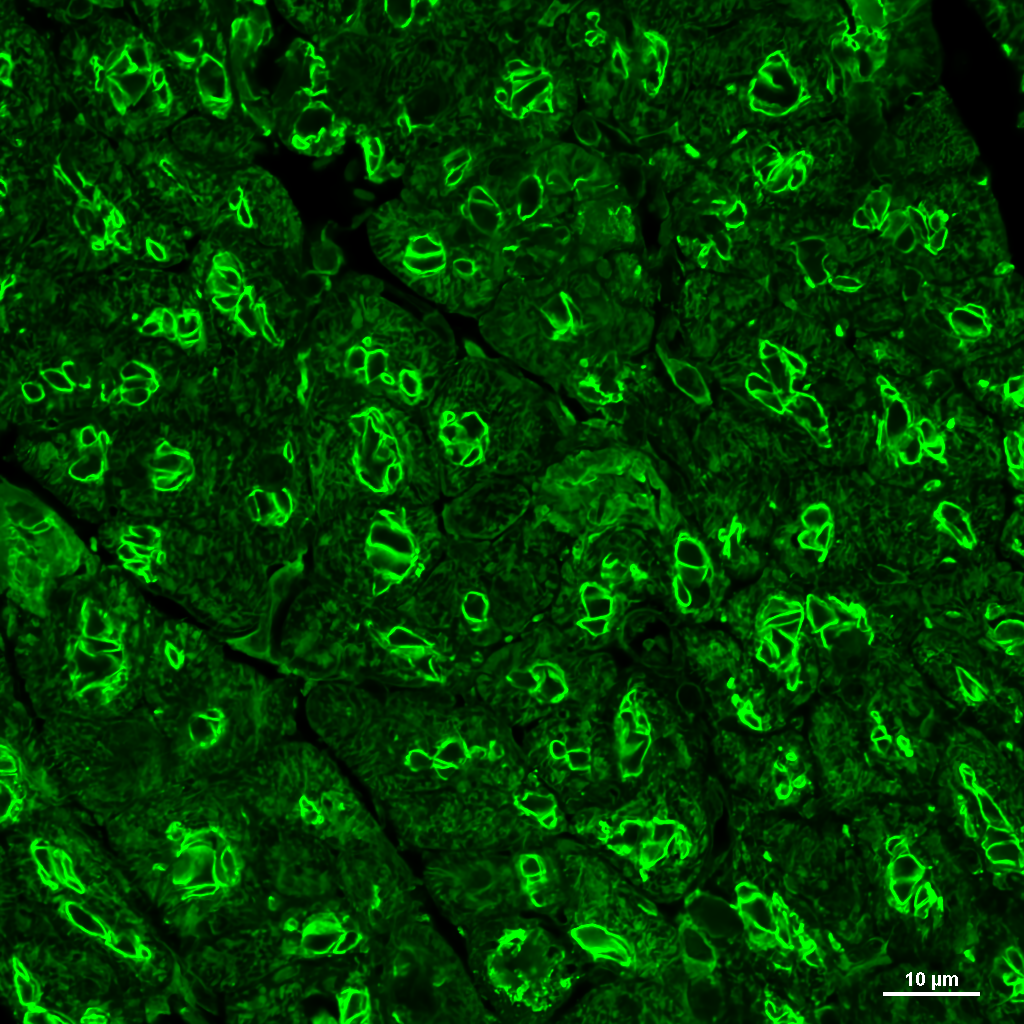

Supplement: Supplementary file 16 — Source data Fig. 9 [file 44321_2026_411_MOESM16_ESM.zip › Figure 9/9E/R120G_Ruxo_CRYAB.tif]

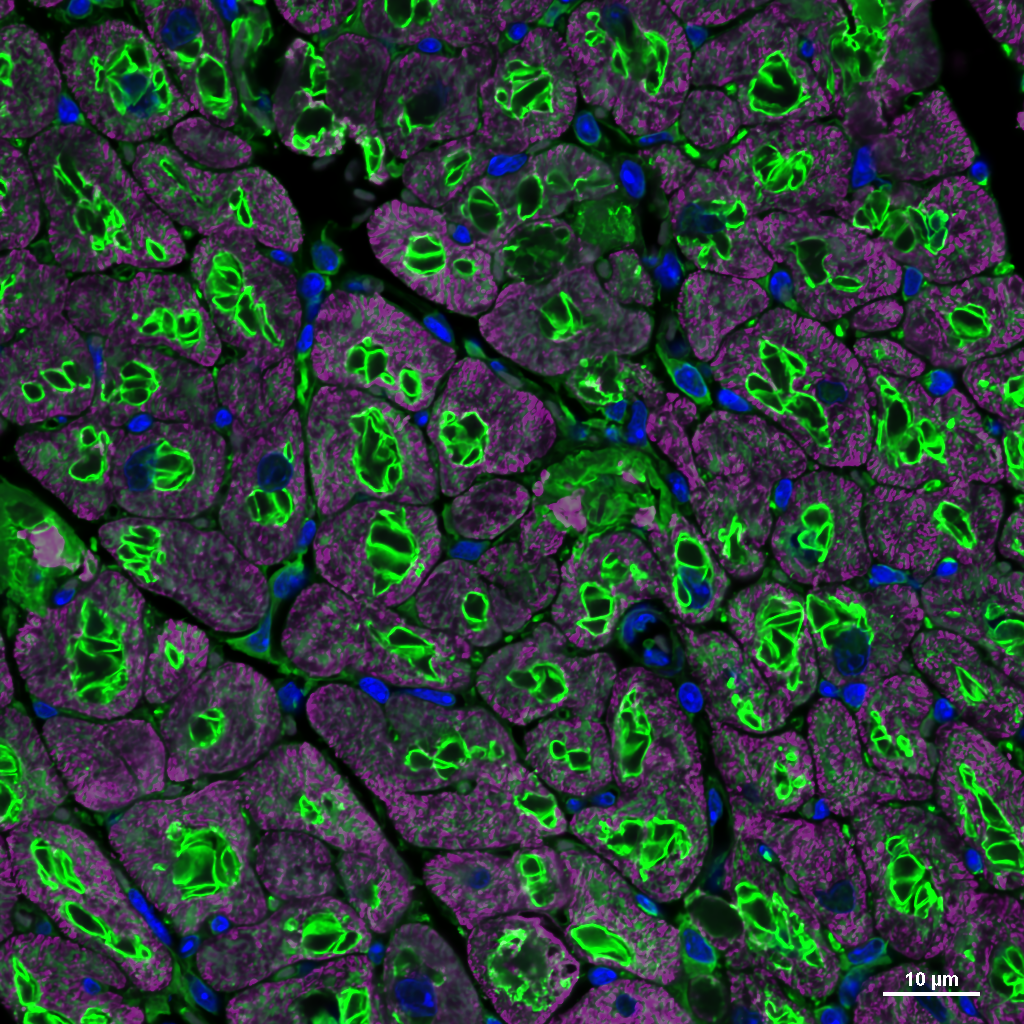

Supplement: Supplementary file 16 — Source data Fig. 9 [file 44321_2026_411_MOESM16_ESM.zip › Figure 9/9E/R120G_Ruxo_RGB.tif]

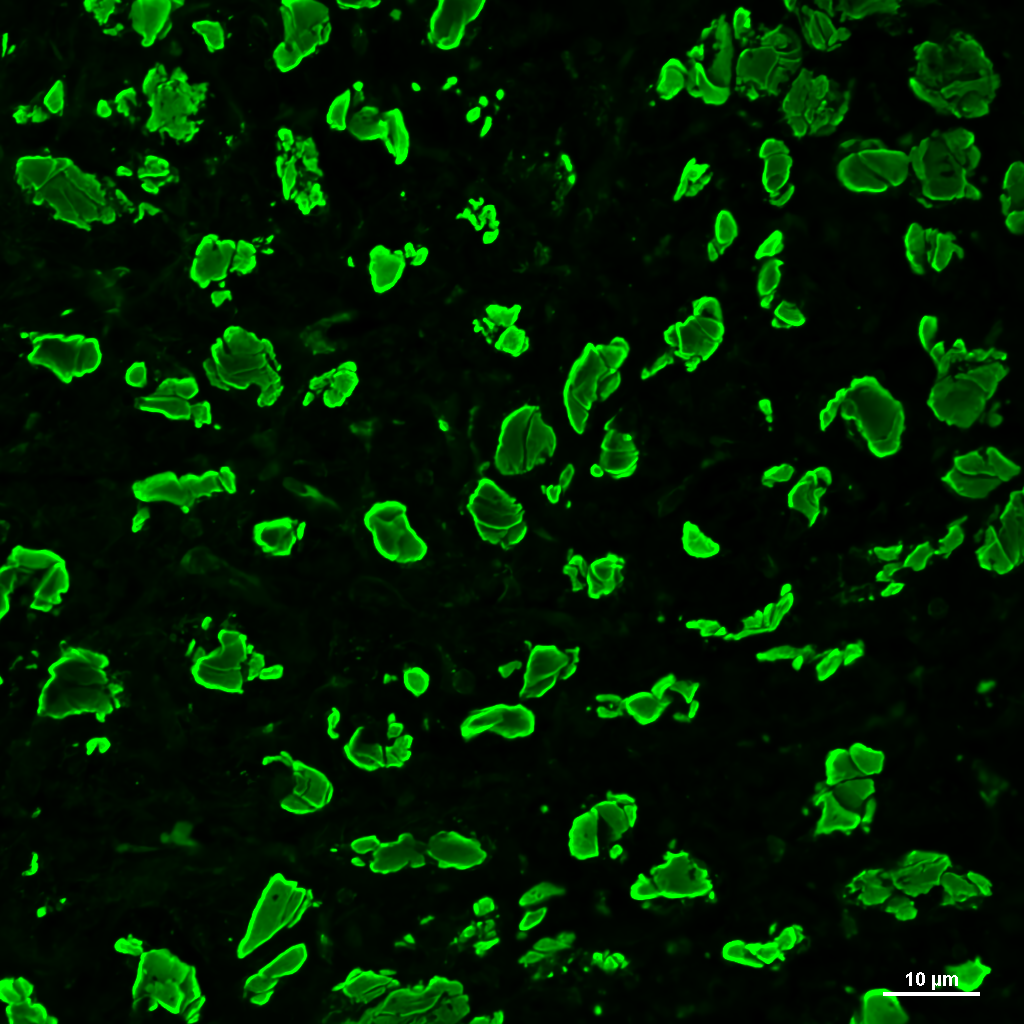

Supplement: Supplementary file 16 — Source data Fig. 9 [file 44321_2026_411_MOESM16_ESM.zip › Figure 9/9E/R120G_Veh_CRYAB.tif]

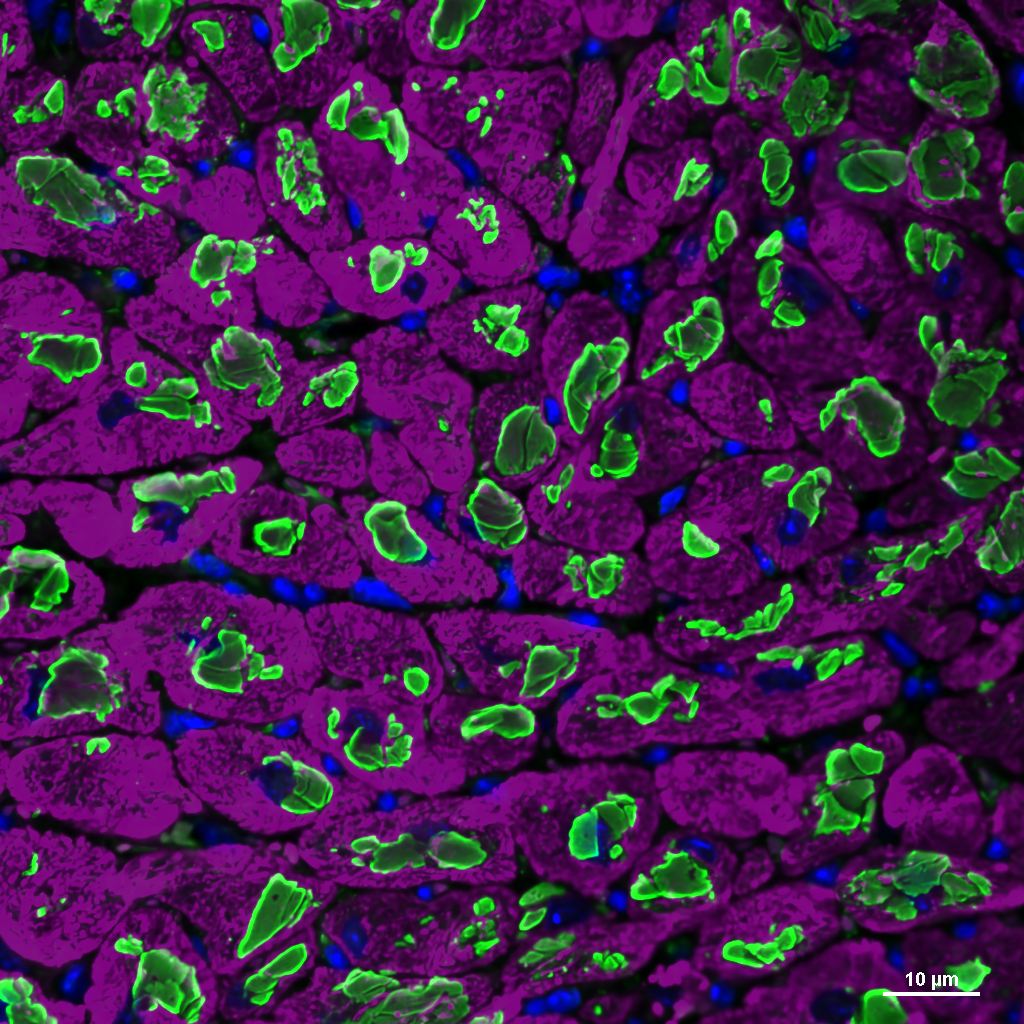

Supplement: Supplementary file 16 — Source data Fig. 9 [file 44321_2026_411_MOESM16_ESM.zip › Figure 9/9E/R120G_Veh_RGB.tif]

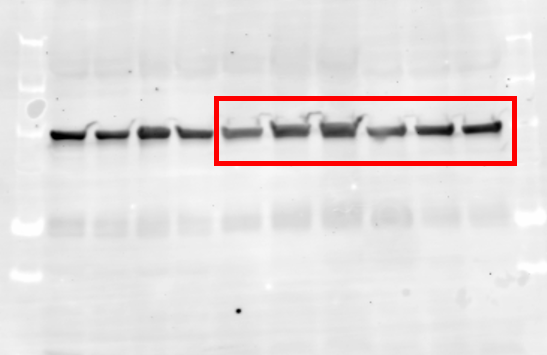

Supplement: Supplementary file 16 — Source data Fig. 9 [file 44321_2026_411_MOESM16_ESM.zip › Figure 9/9G/ACTN2_Insoluble.tif]

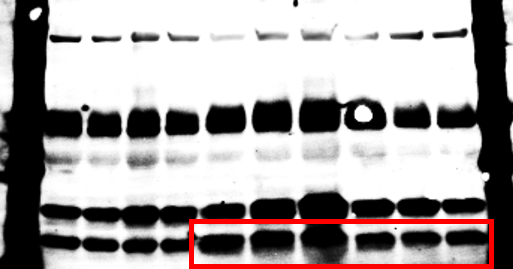

Supplement: Supplementary file 16 — Source data Fig. 9 [file 44321_2026_411_MOESM16_ESM.zip › Figure 9/9G/CRYAB_Insoluble.tif]

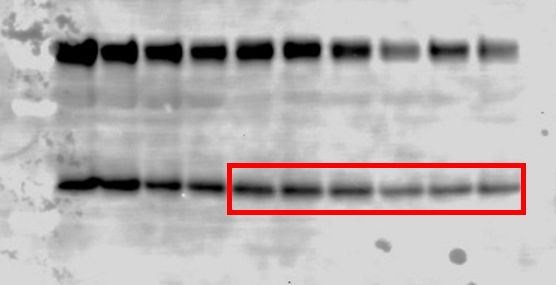

Supplement: Supplementary file 16 — Source data Fig. 9 [file 44321_2026_411_MOESM16_ESM.zip › Figure 9/9G/CRYAB_Soluble.tif]

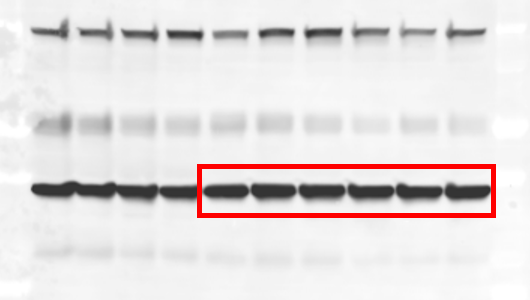

Supplement: Supplementary file 16 — Source data Fig. 9 [file 44321_2026_411_MOESM16_ESM.zip › Figure 9/9G/GAPDH_Soluble.tif]

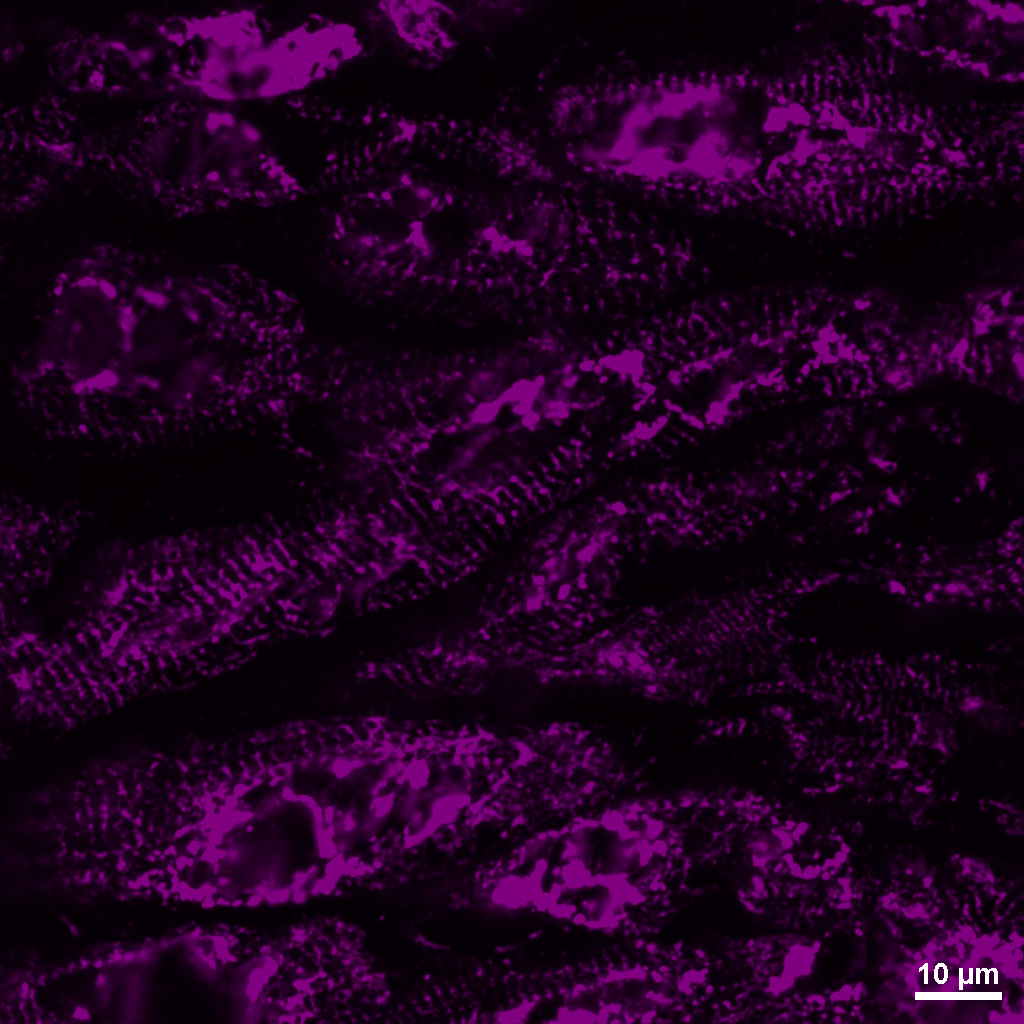

Supplement: Supplementary file 16 — Source data Fig. 9 [file 44321_2026_411_MOESM16_ESM.zip › Figure 9/9H/R120G-Ruxo_Desmin.tif]

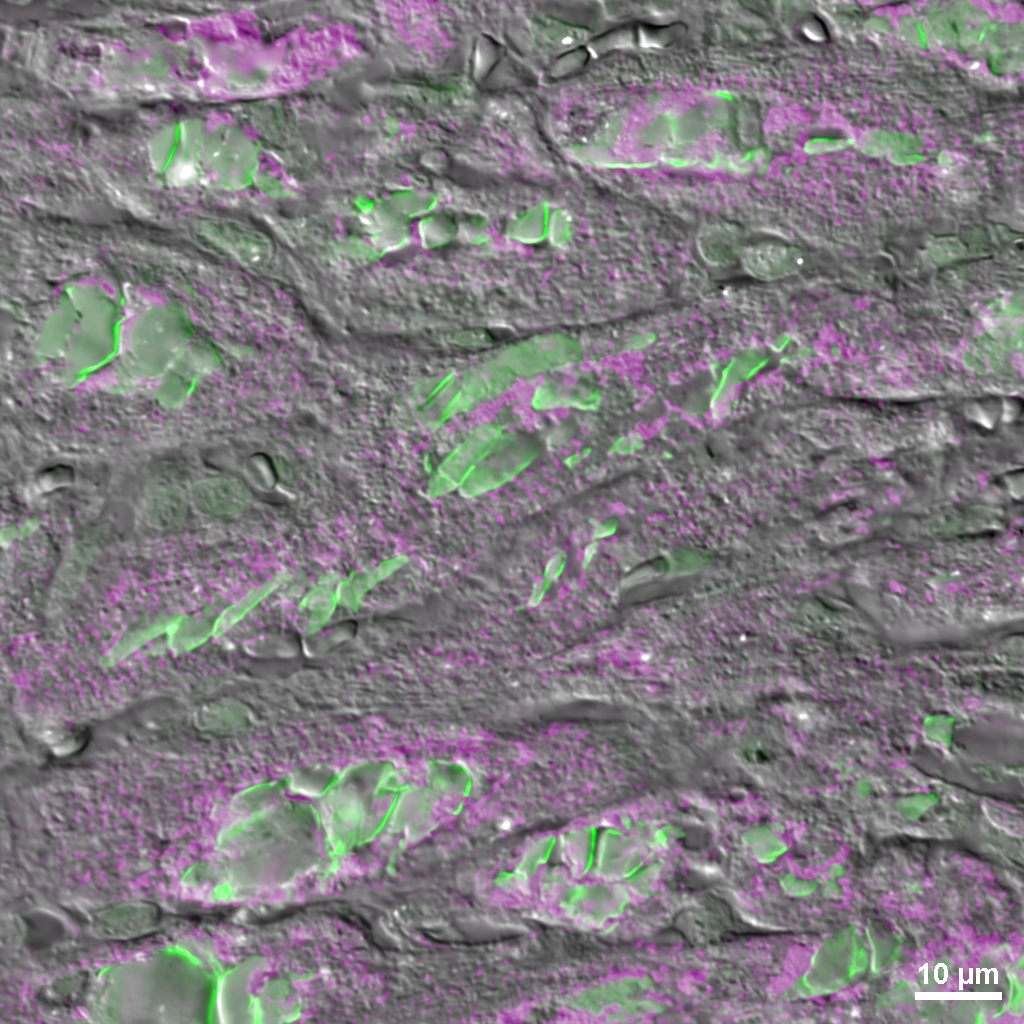

Supplement: Supplementary file 16 — Source data Fig. 9 [file 44321_2026_411_MOESM16_ESM.zip › Figure 9/9H/R120G-Ruxo_Overlay.tif]

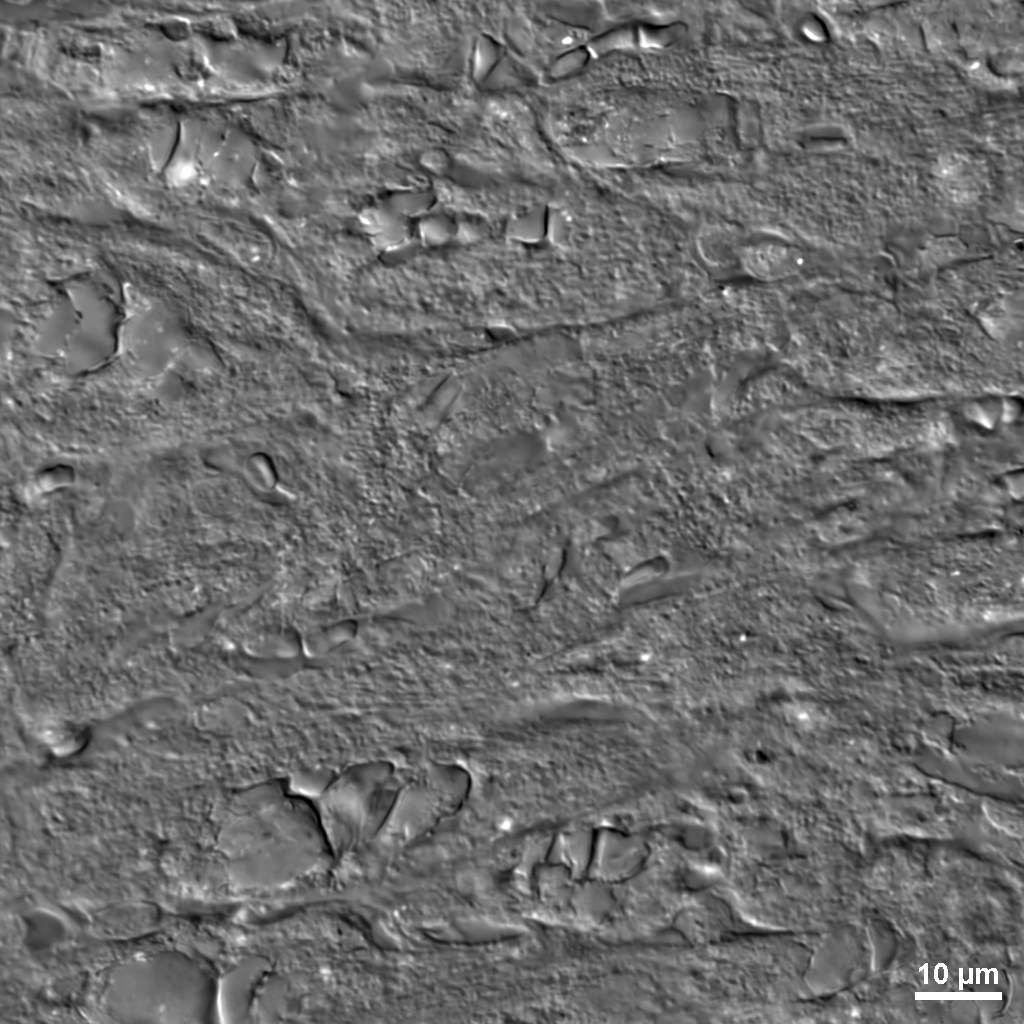

Supplement: Supplementary file 16 — Source data Fig. 9 [file 44321_2026_411_MOESM16_ESM.zip › Figure 9/9H/R120G-Ruxo_TD.tif]

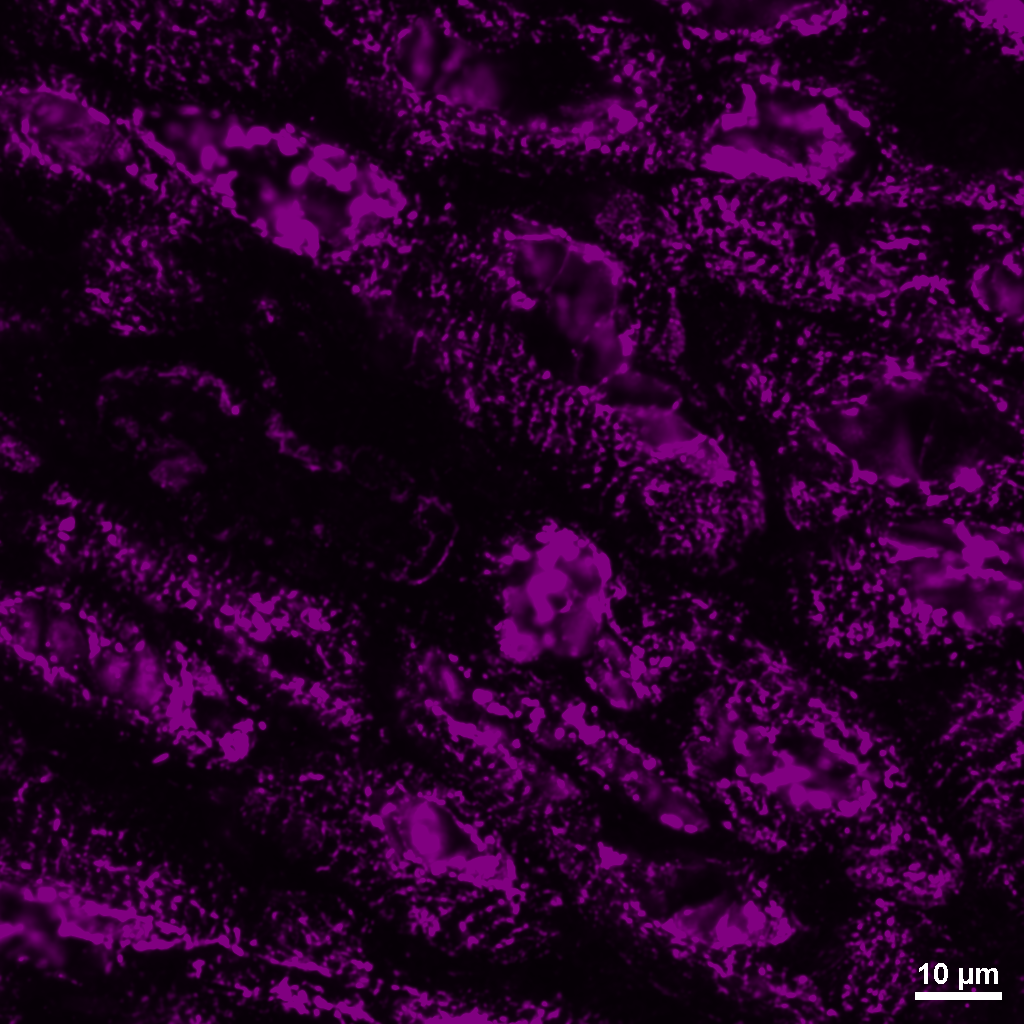

Supplement: Supplementary file 16 — Source data Fig. 9 [file 44321_2026_411_MOESM16_ESM.zip › Figure 9/9H/R120G-Veh_Desmin.tif]

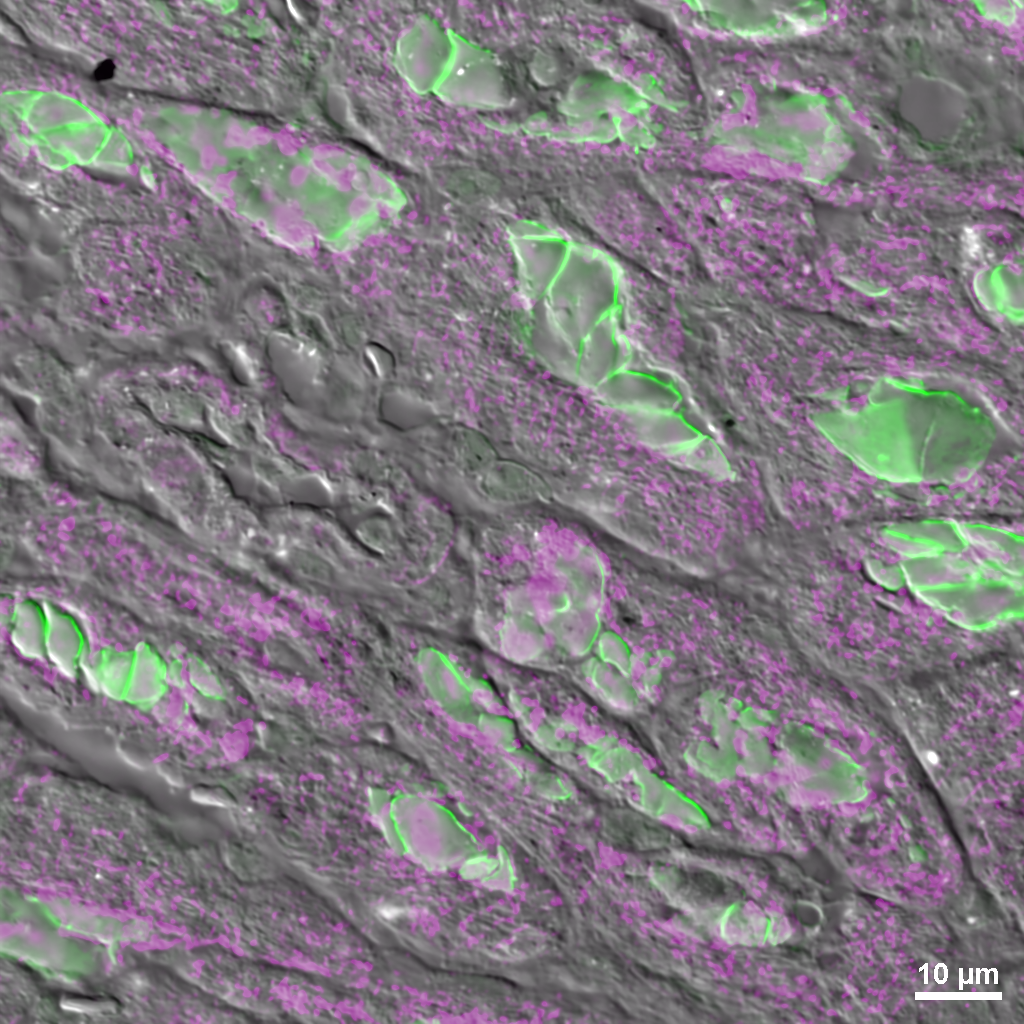

Supplement: Supplementary file 16 — Source data Fig. 9 [file 44321_2026_411_MOESM16_ESM.zip › Figure 9/9H/R120G-Veh_Overlay.tif]

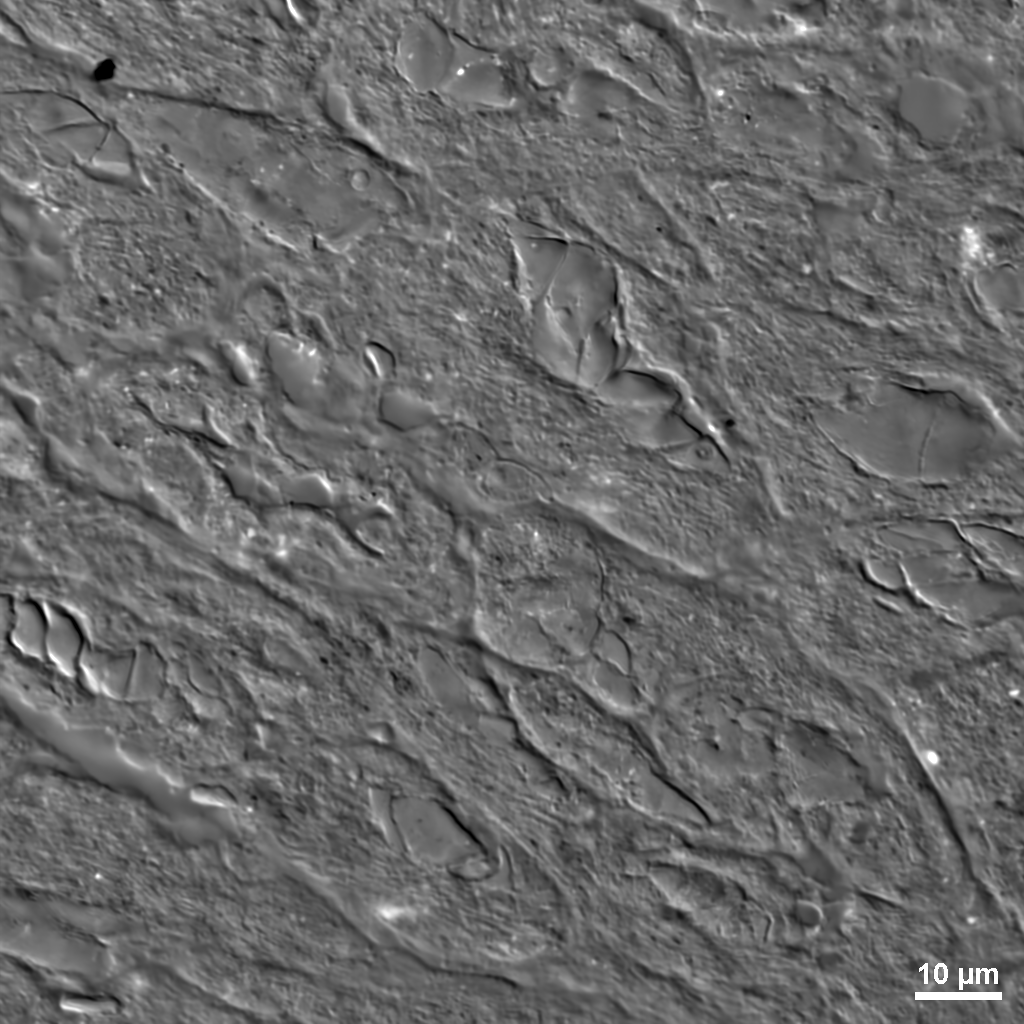

Supplement: Supplementary file 16 — Source data Fig. 9 [file 44321_2026_411_MOESM16_ESM.zip › Figure 9/9H/R120G-Veh_TD.tif]

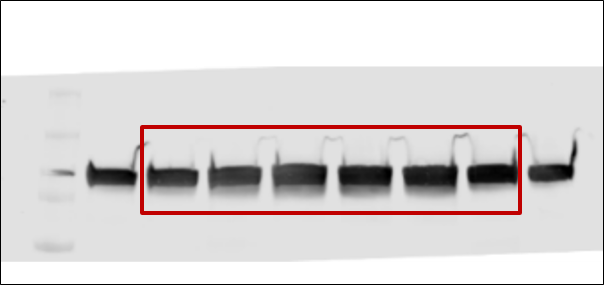

Supplement: Supplementary file 17 — Source data Fig. 10 [file 44321_2026_411_MOESM17_ESM.zip › Figure 10/10C/western ACTN2.tif]

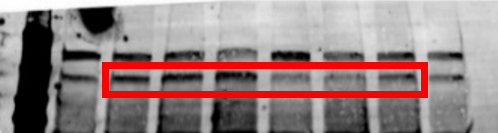

Supplement: Supplementary file 17 — Source data Fig. 10 [file 44321_2026_411_MOESM17_ESM.zip › Figure 10/10C/western JAK1.tif]

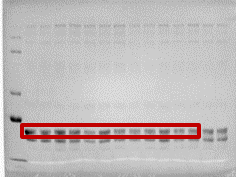

Supplement: Supplementary file 17 — Source data Fig. 10 [file 44321_2026_411_MOESM17_ESM.zip › Figure 10/10F/western GAPDH.tif]

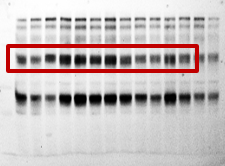

Supplement: Supplementary file 17 — Source data Fig. 10 [file 44321_2026_411_MOESM17_ESM.zip › Figure 10/10F/western P-STAT3.tif]

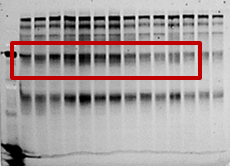

Supplement: Supplementary file 17 — Source data Fig. 10 [file 44321_2026_411_MOESM17_ESM.zip › Figure 10/10F/western STAT3.tif]

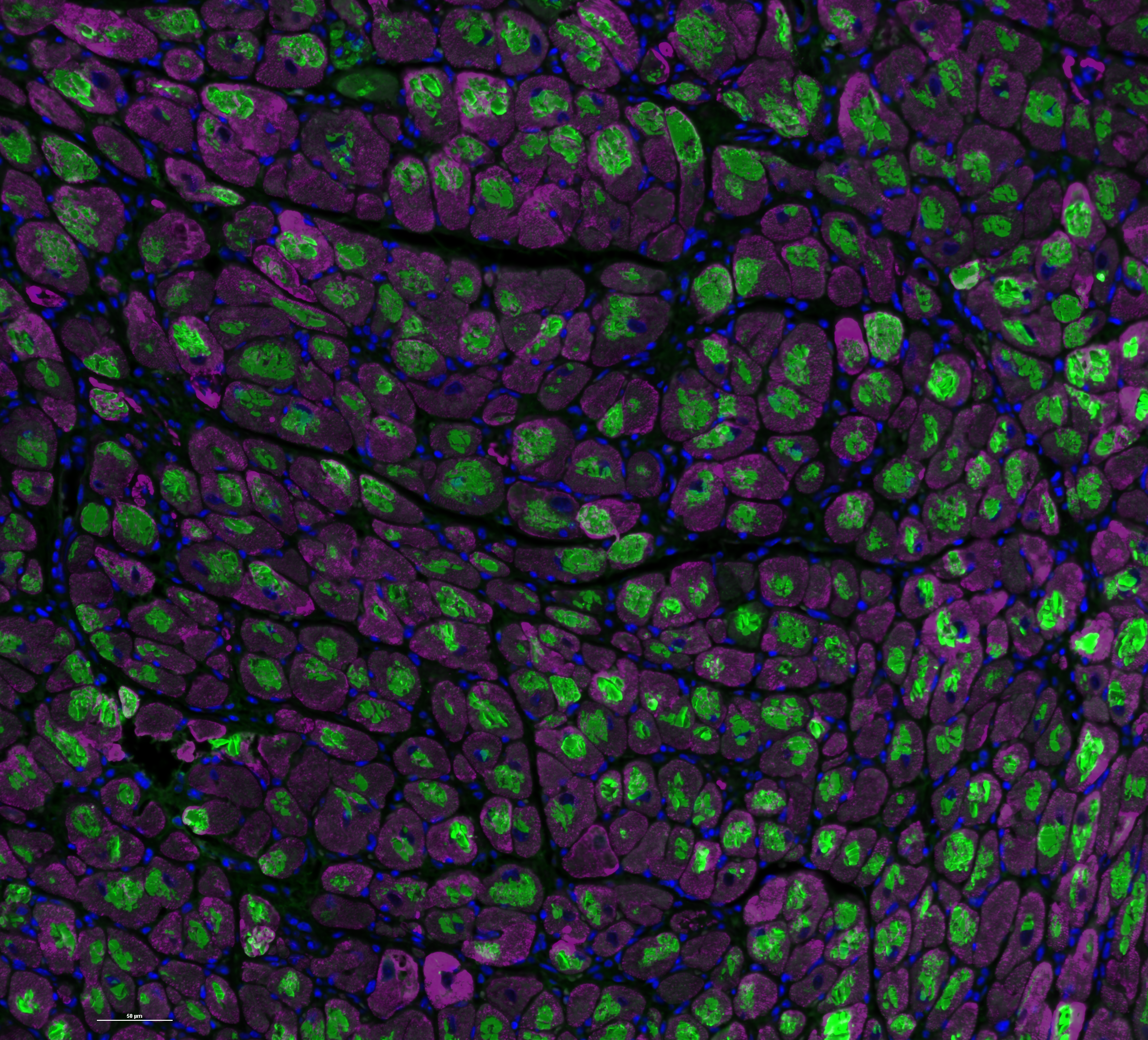

Supplement: Supplementary file 17 — Source data Fig. 10 [file 44321_2026_411_MOESM17_ESM.zip › Figure 10/10G/R120G-Jak1 hom KO.tif]

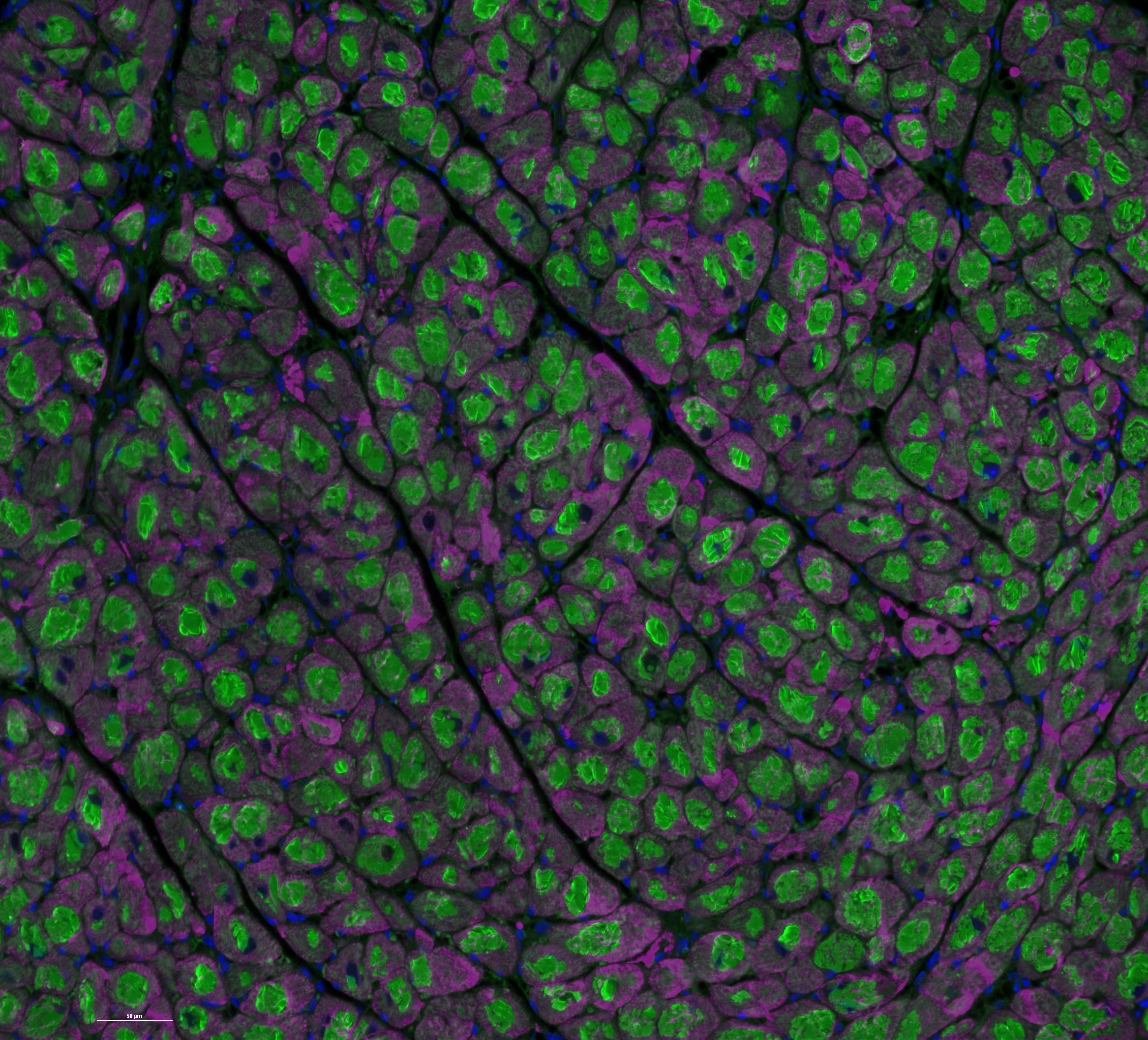

Supplement: Supplementary file 17 — Source data Fig. 10 [file 44321_2026_411_MOESM17_ESM.zip › Figure 10/10G/R120G-Jak1 wt.tif]

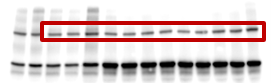

Supplement: Supplementary file 17 — Source data Fig. 10 [file 44321_2026_411_MOESM17_ESM.zip › Figure 10/10H/western ACTN2.png]

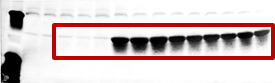

Supplement: Supplementary file 17 — Source data Fig. 10 [file 44321_2026_411_MOESM17_ESM.zip › Figure 10/10H/western CRYAB 1.png]

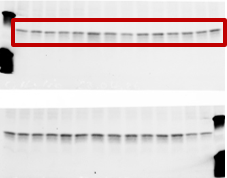

Supplement: Supplementary file 17 — Source data Fig. 10 [file 44321_2026_411_MOESM17_ESM.zip › Figure 10/10H/western CRYAB.png]

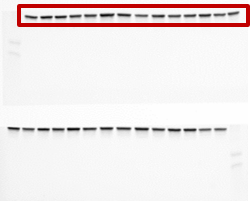

Supplement: Supplementary file 17 — Source data Fig. 10 [file 44321_2026_411_MOESM17_ESM.zip › Figure 10/10H/western GAPDH.png]

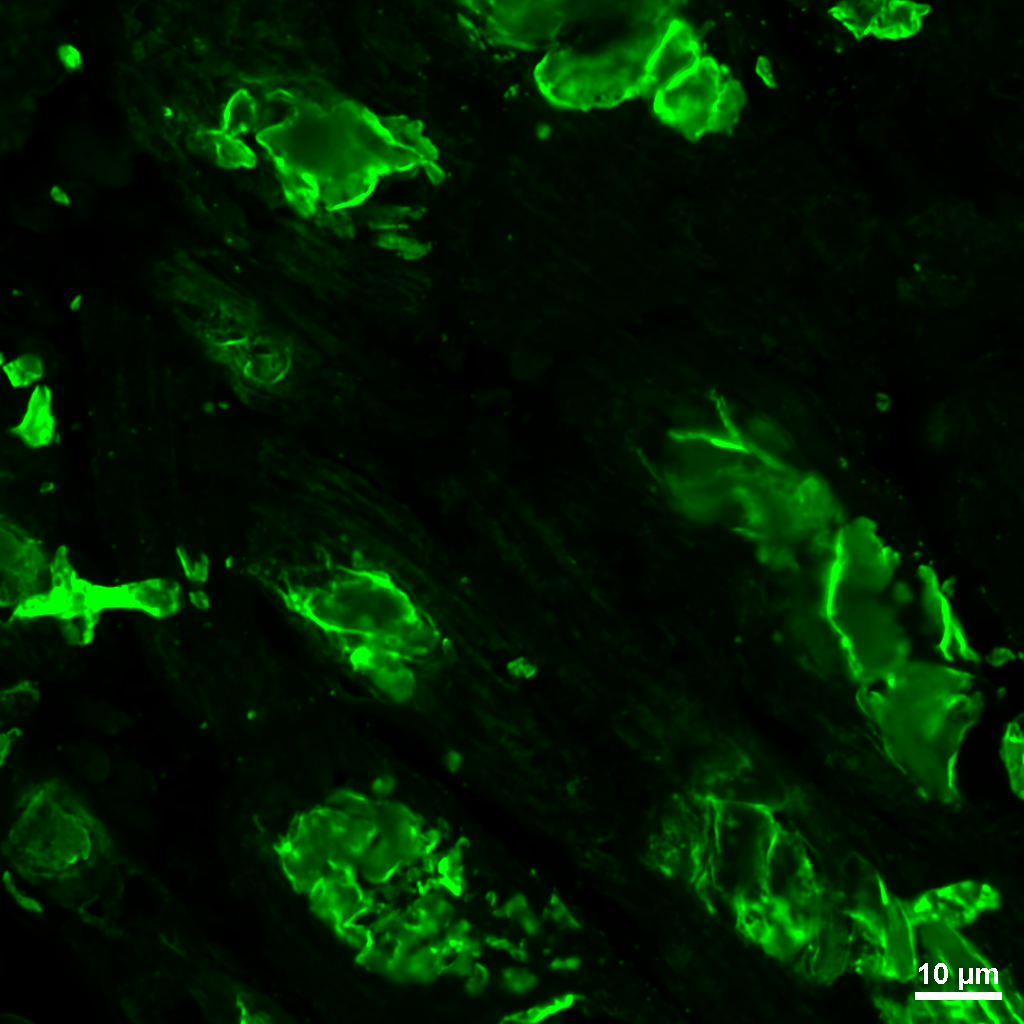

Supplement: Supplementary file 17 — Source data Fig. 10 [file 44321_2026_411_MOESM17_ESM.zip › Figure 10/10I/R120G-Jak1 hom KO_CRYAB.tif]

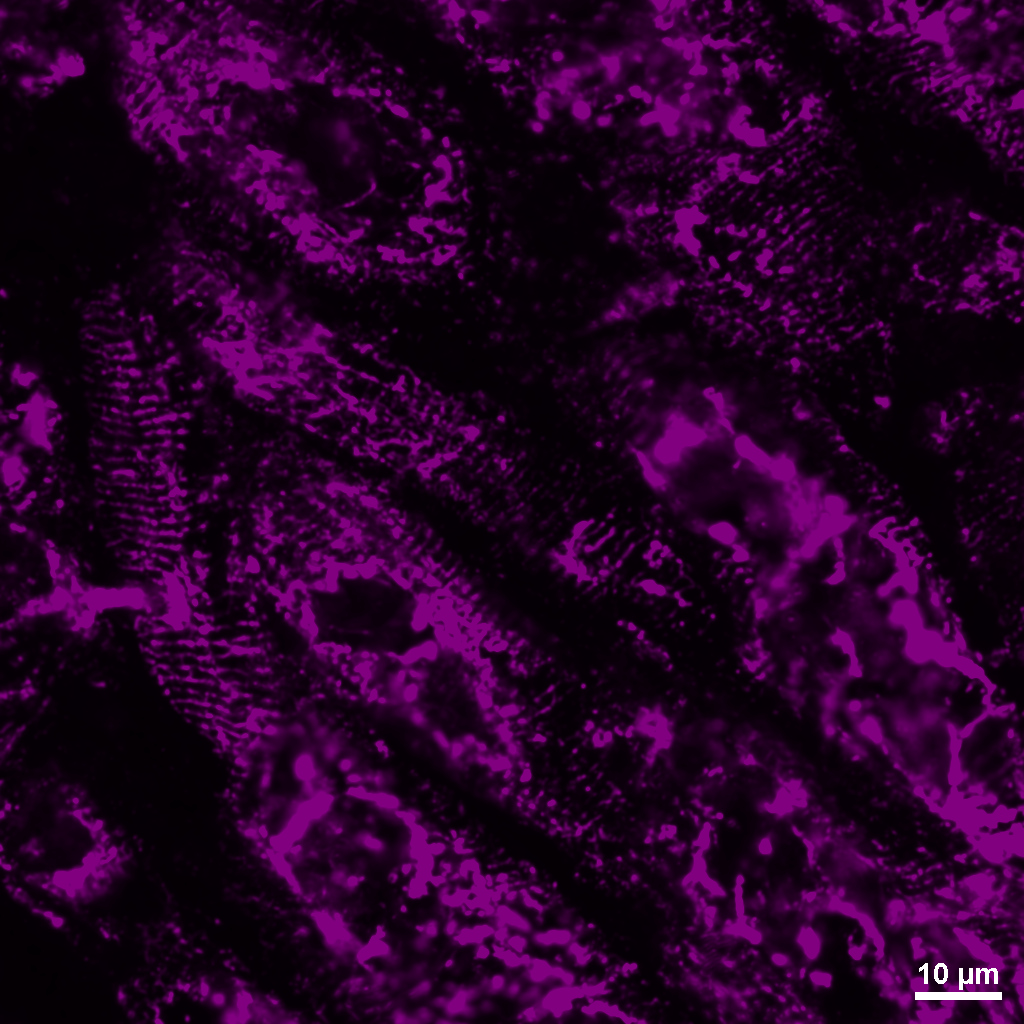

Supplement: Supplementary file 17 — Source data Fig. 10 [file 44321_2026_411_MOESM17_ESM.zip › Figure 10/10I/R120G-Jak1 hom KO_Desmin.tif]

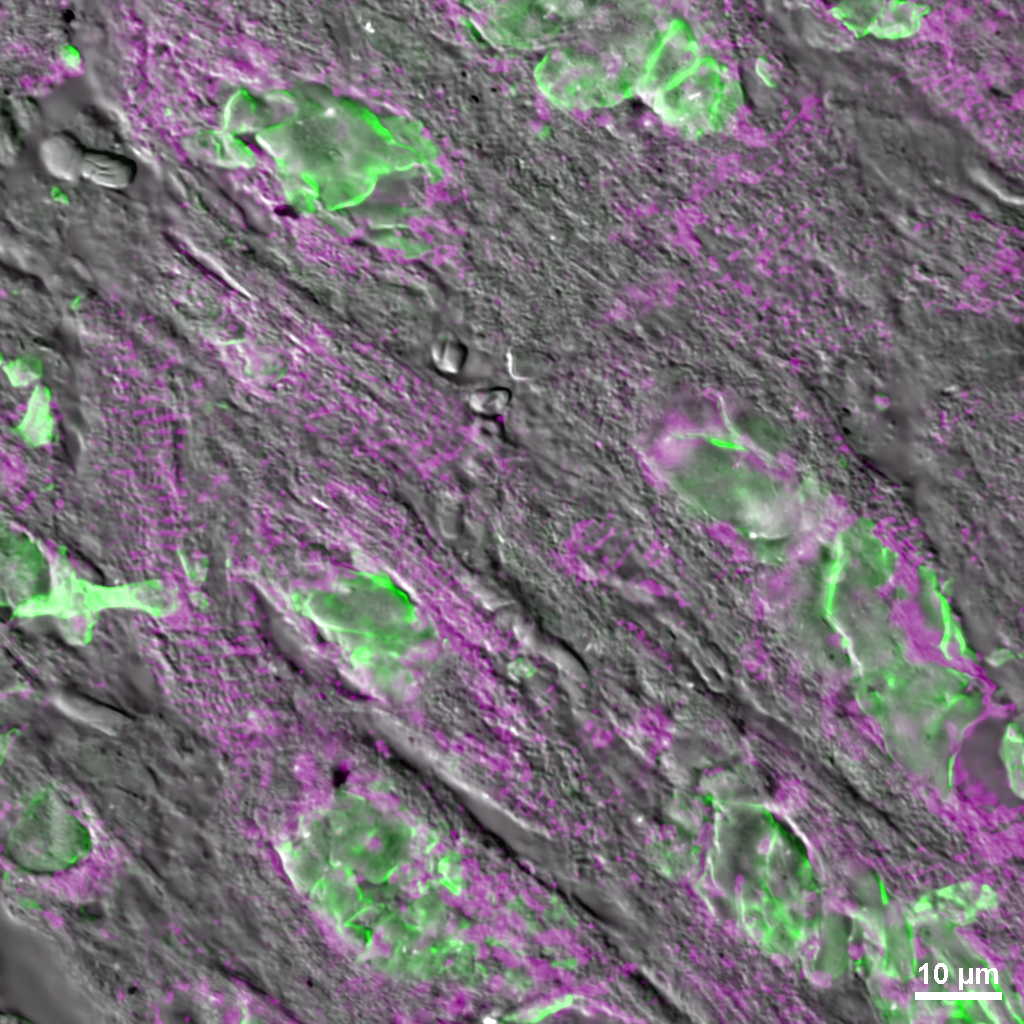

Supplement: Supplementary file 17 — Source data Fig. 10 [file 44321_2026_411_MOESM17_ESM.zip › Figure 10/10I/R120G-Jak1 hom KO_Overlay.tif]

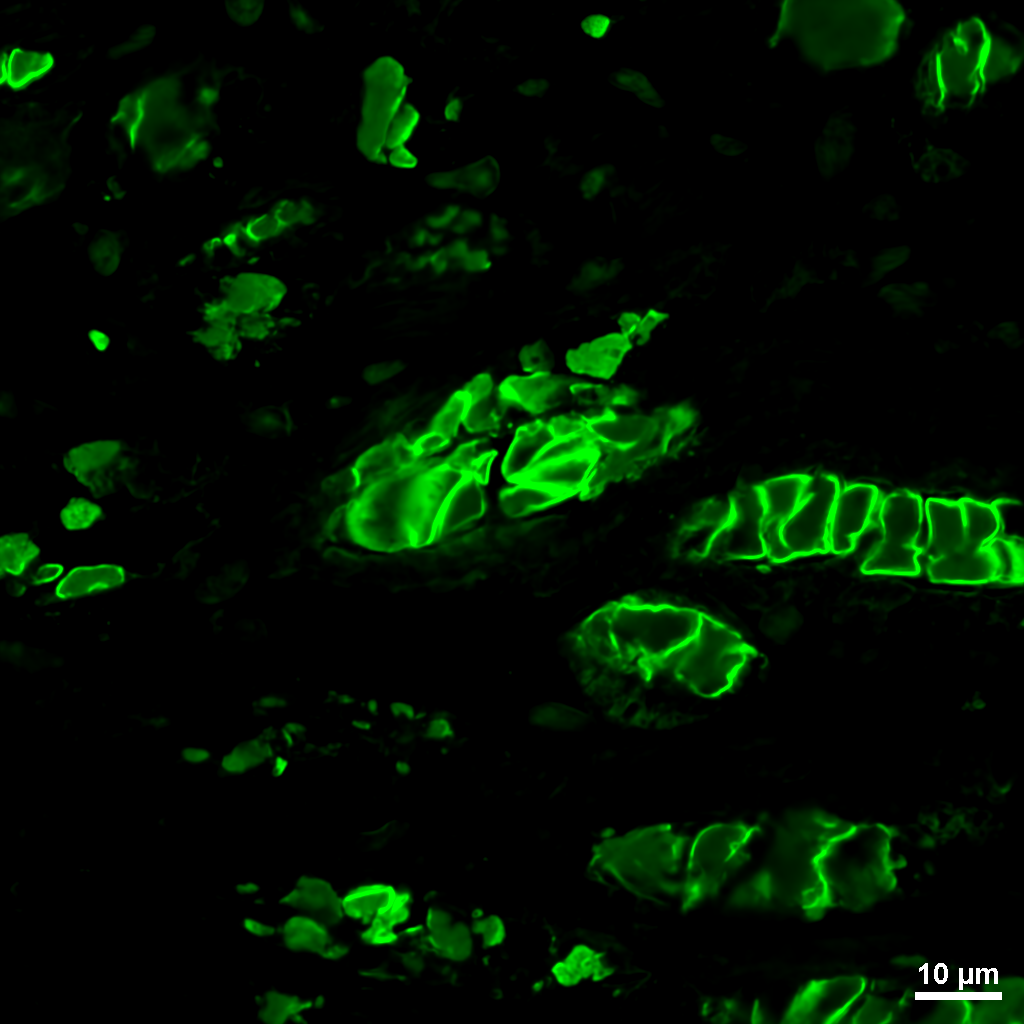

Supplement: Supplementary file 17 — Source data Fig. 10 [file 44321_2026_411_MOESM17_ESM.zip › Figure 10/10I/R120G-Jak1 wt_CRYAB.tif]

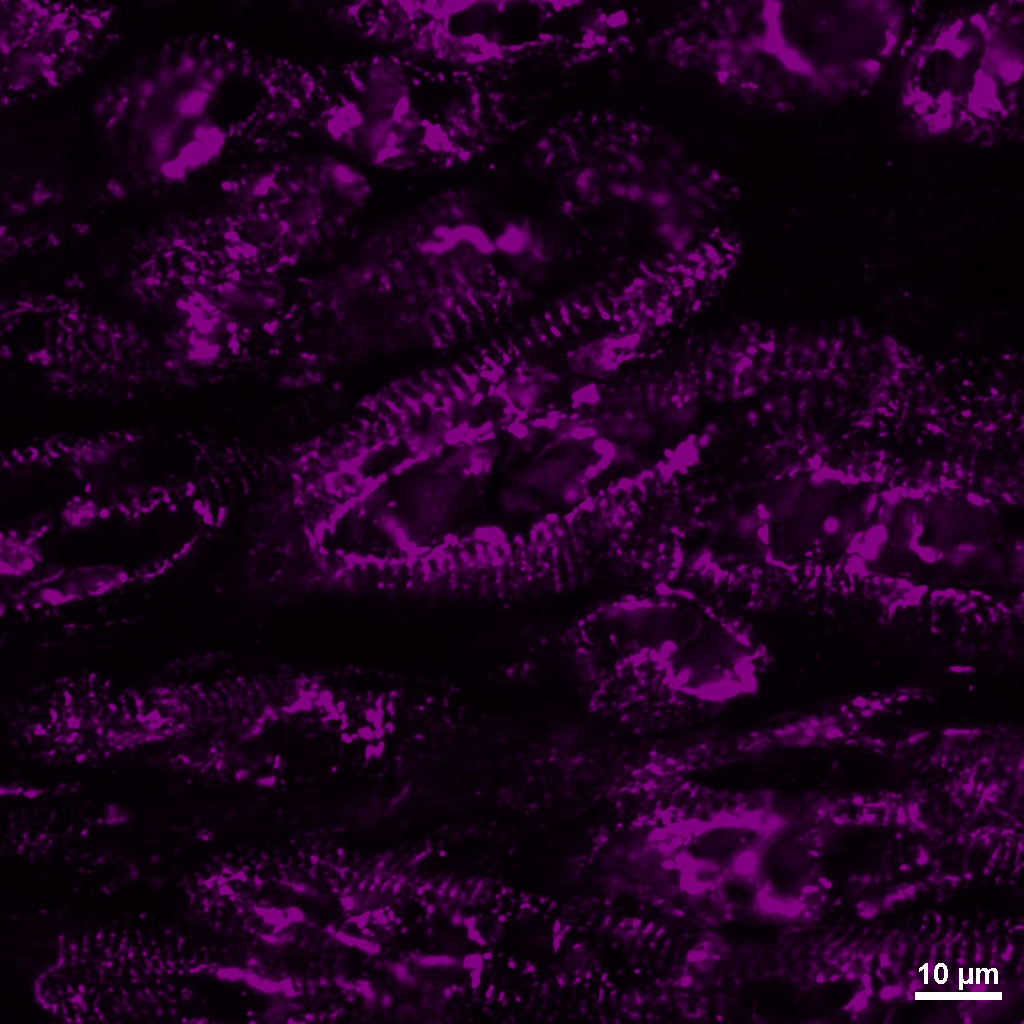

Supplement: Supplementary file 17 — Source data Fig. 10 [file 44321_2026_411_MOESM17_ESM.zip › Figure 10/10I/R120G-Jak1 wt_Desmin.tif]

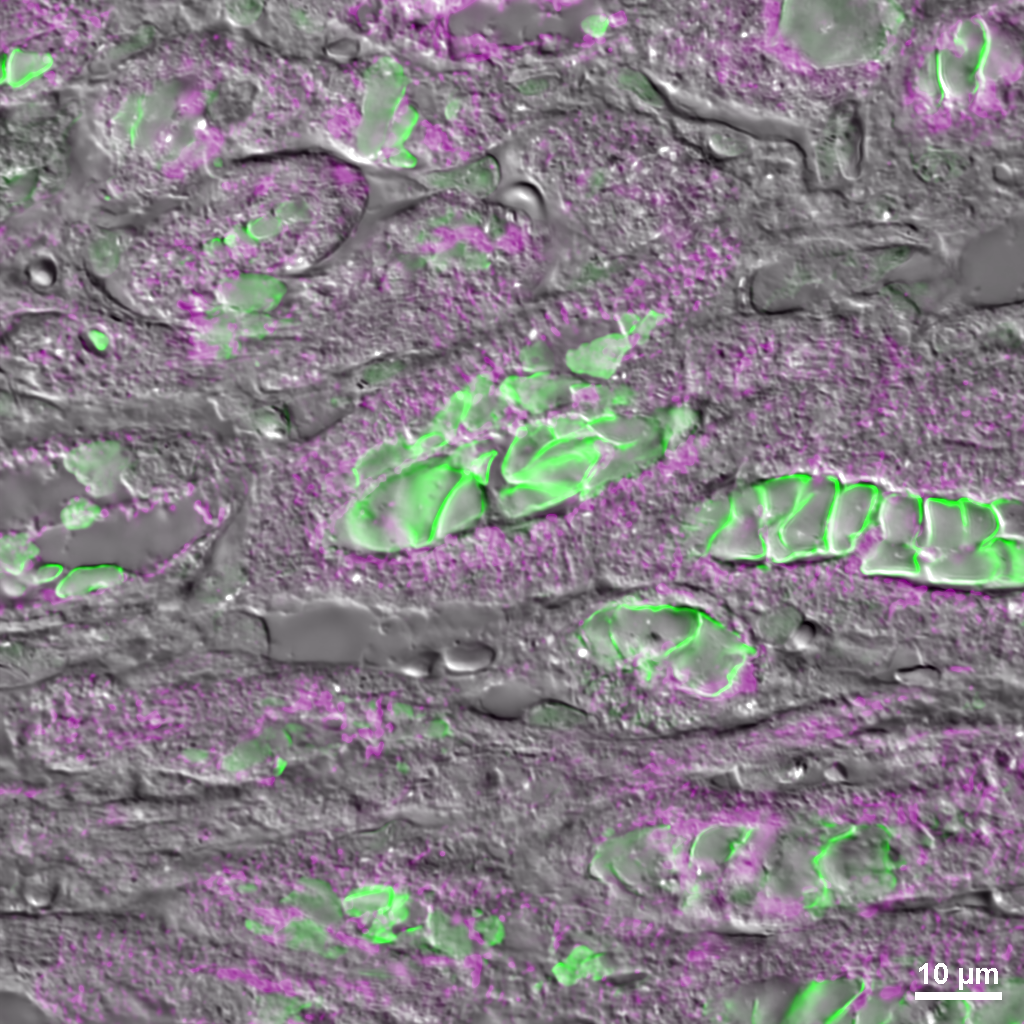

Supplement: Supplementary file 17 — Source data Fig. 10 [file 44321_2026_411_MOESM17_ESM.zip › Figure 10/10I/R120G-Jak1 wt_Overlay.tif]
