# Supplementary material for: JAK1/2 inhibitor ruxolitinib reduces aggregates in cardiac proteinopathy
Source: EMBO Mol Med. 2026 Mar 31;18(5):1836–65. doi: 10.1038/s44321-026-00411-x (PMC13179346; doi:10.1038/s44321-026-00411-x)
Supplement: Supplementary file 18 — Expanded View Figures [file 44321_2026_411_MOESM18_ESM.pdf]

Expanded View Figures

Figure EV1. Ruxolitinib treatment prevents CRYAB^{R120G} aggregate formation at early and late stages and partially clears pre-existing aggregates.

(**A**) Ruxolitinib concentration-response curve on phosphorylated STAT3 levels in NRVMs. NRVMs were treated with indicated concentrations of ruxolitinib or 0.1% DMSO. Western blot of protein extracts from treated NRVMs was stained with antibodies directed against phosphorylated (P-) STAT3 and STAT3. (**B**, **C**) NRVMs were transduced with AdV5-CMV-CRYAB^{R120G}-GFP and treated with either 3 μM ruxolitinib or DMSO on the indicated day. Medium change with ruxolitinib or DMSO was performed every other day. (**B**) Quantification of aggregates in cardiomyocytes with NIS Elements software. Data were obtained from 2 independent NRVM preparations (prep) with 3 wells per prep, 7–8 images per well and are depicted as mean ± SEM, one-way ANOVA, Dunnett's post-hoc analysis for the same time points or unpaired Student's t-test for comparison of start and end of the experiment within one group. (**C**) Representative IF images. Aggregates are depicted in magenta (CRYAB^{R120G}-GFP), cardiomyocytes in yellow (anti-cardiac troponin I), and nuclei in blue (DAPI). Scale bar = 100 μm.

© The Author(s)

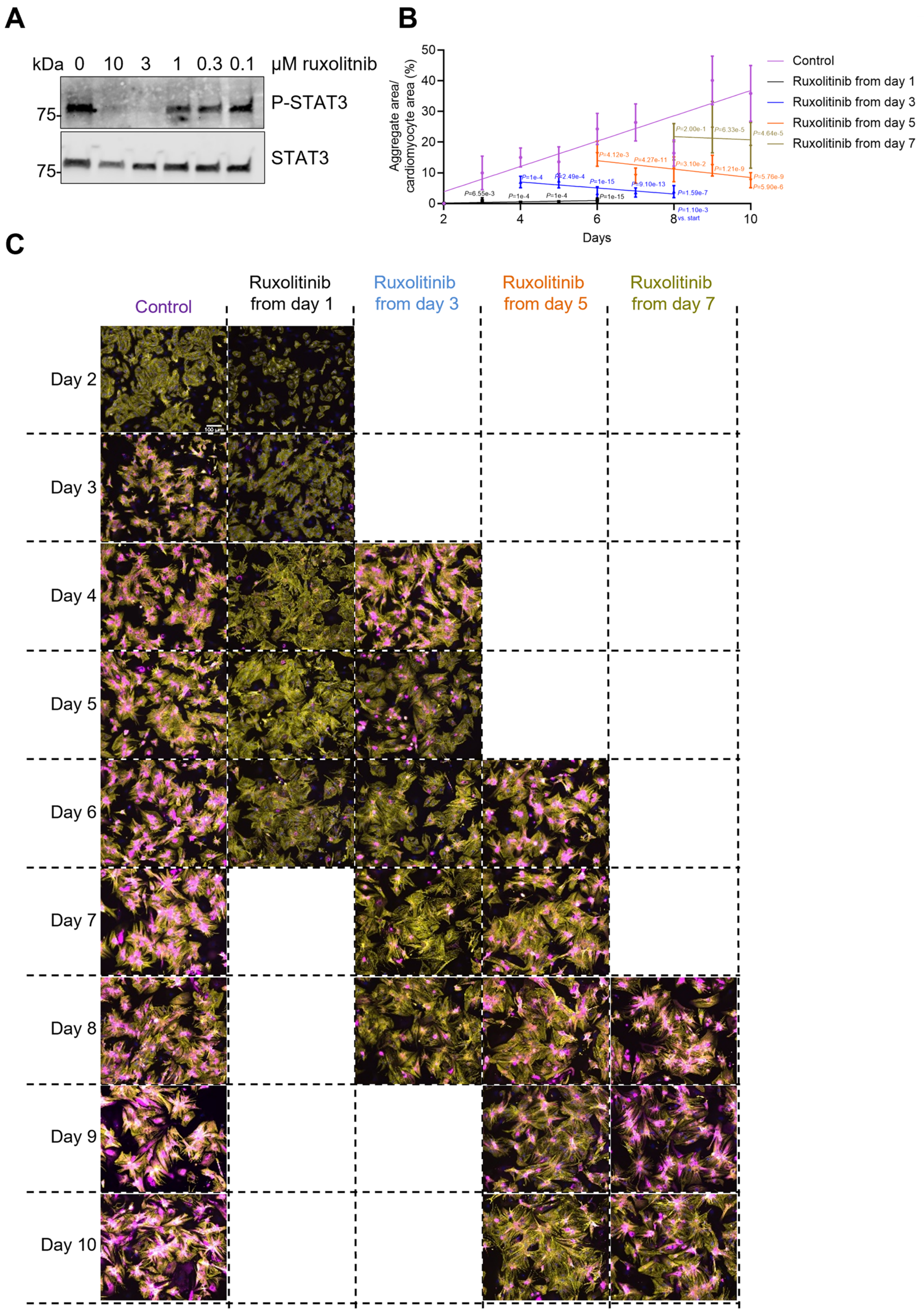
A
kDa 0 10 3 1 0.3 0.1 µM ruxolitnib
75-
P-STAT3
75-
STAT3
B
Aggregate area/ cardiomyocyte area (%)
Days
Control
Ruxolitinib from day 1
Ruxolitinib from day 3
Ruxolitinib from day 5
Ruxolitinib from day 7
P=6.55e-3
P=1e-4
P=1e-4
P=1e-15
P=1e-4
P=2.49e-4
P=1e-15
P=9.10e-13
P=1.59e-7
P=1.10e-3 vs. start
P=4.12e-3
P=4.27e-11
P=3.10e-2
P=1.21e-9
P=5.76e-9
P=5.90e-6
P=2.00e-1
P=6.33e-5
P=4.64e-5
C
Control
Ruxolitinib from day 1
Ruxolitinib from day 3
Ruxolitinib from day 5
Ruxolitinib from day 7
Day 2
Day 3
Day 4
Day 5
Day 6
Day 7
Day 8
Day 9
Day 10

 © The Author(s)

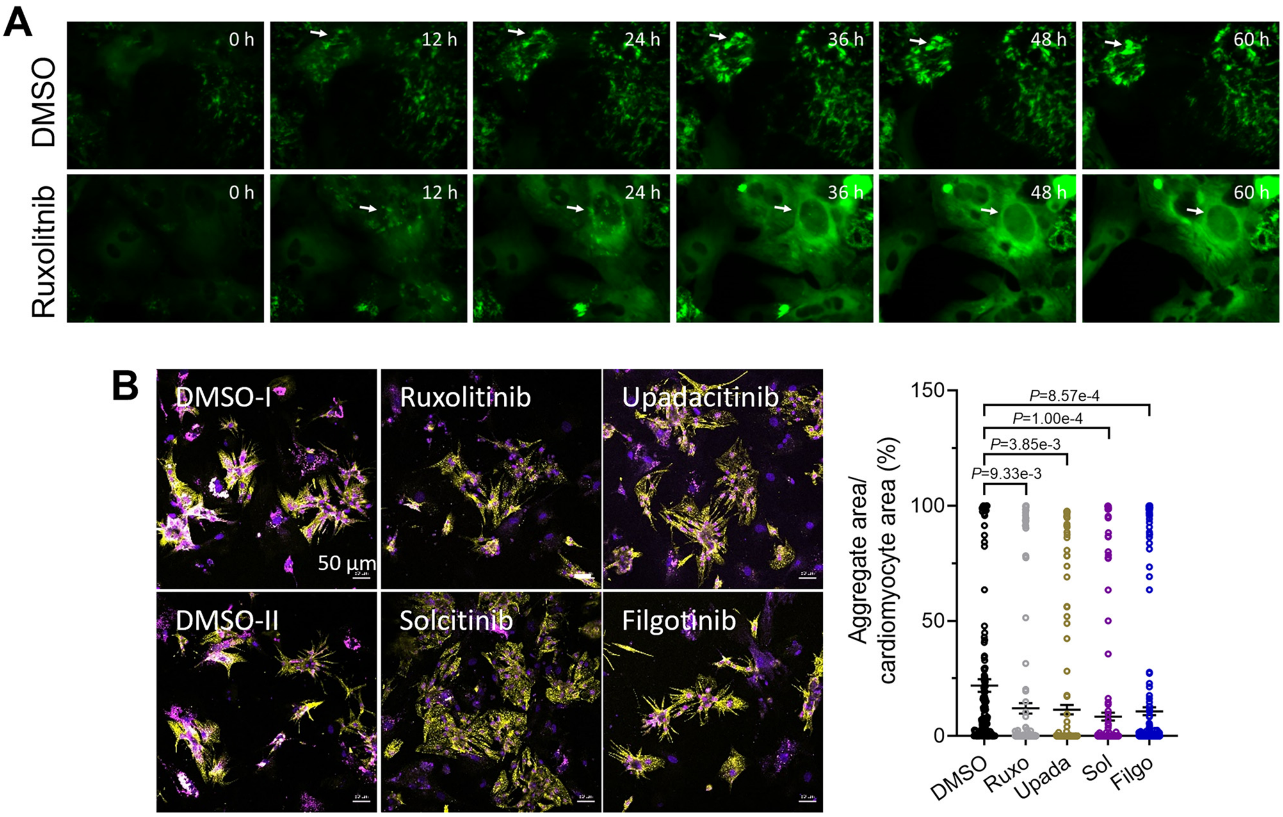

Figure EV2. Ruxolitinib and selective JAK1 inhibitors reduce CRYAB^{R120G} aggregates in hiPSC-CMs.

(**A**) Live cell imaging of hiPSC-CMs transduced with AdV5-CMV-CRYAB^{R120G}. Representative fluorescence images of videos from hiPSC-CMs treated with 3 µM ruxolitinib or 0.1% DMSO were taken with a Nikon Biostation IM-Q time-lapse with a Nikon Biostation IM-Q time lapse imaging system (widefield). Aggregates are depicted in green (CRYAB^{R120G}-GFP). Arrows mark growing or dissolving aggregates. Corresponding videos can be found in online supplements. (**B**) Representative immunofluorescence images of NRVMs treated with 3 µM ruxolitinib or 1 µM upadacitinib, solcitinib or filgotinib or 0.1% DMSO, transduced with AdV5-CMV-CRYAB^{R120G}-GFP and fixed after 4–6 days. Aggregates are depicted in magenta (CRYAB^{R120G}-GFP), cardiomyocytes in yellow (anti-cardiac troponin I), and nuclei in blue (DAPI). Quantification of aggregates in cardiomyocytes (NRVMs) with ImageJ software. Scale bar = 50 µm. Data were obtained from 2 independent NRVM preparations with at least 4 wells per condition and 5–8 images per well and are depicted as mean ± SEM, with *p*-values obtained with the one-way ANOVA and Dunnett's post-hoc analysis.

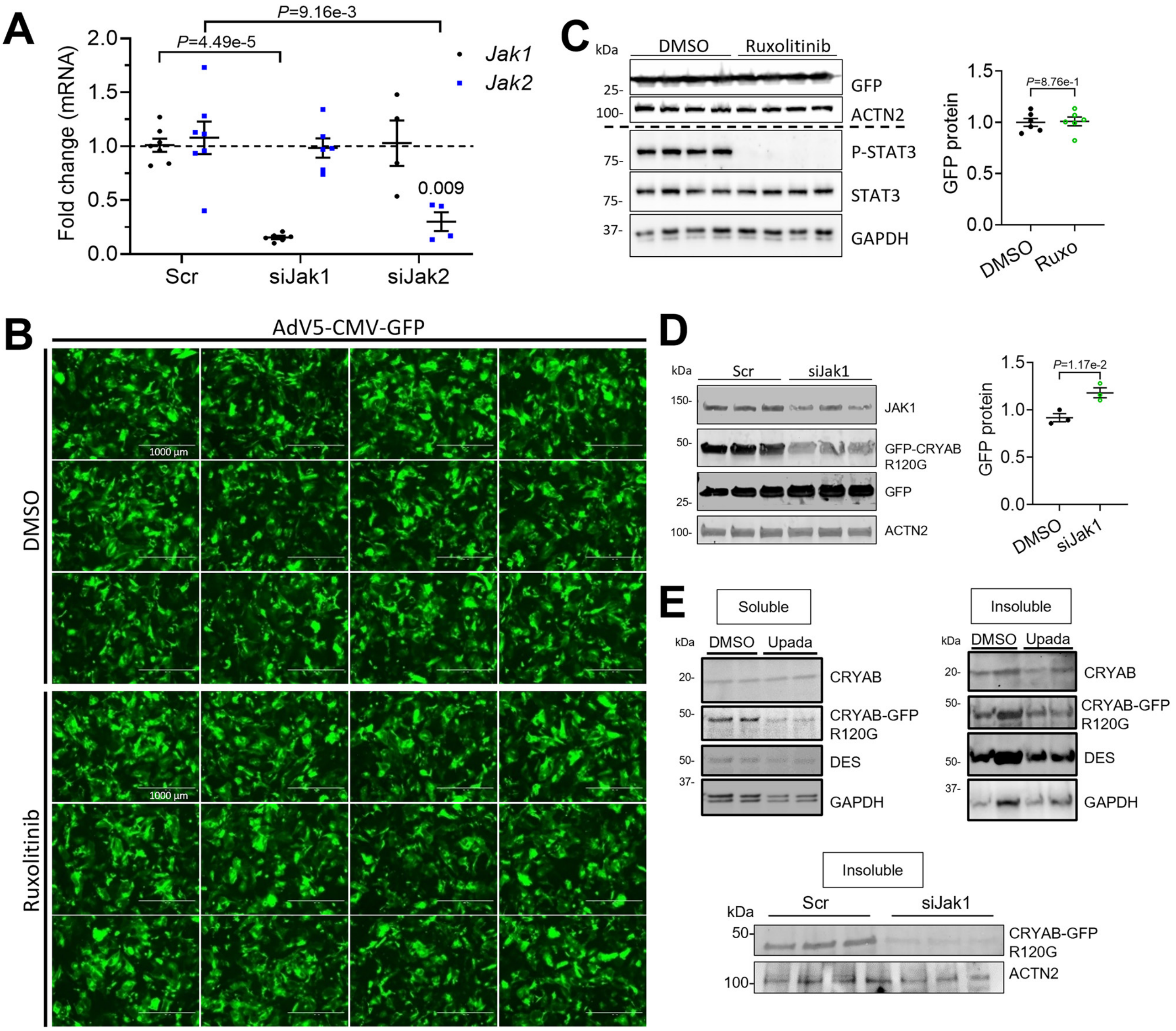

Figure EV3. Ruxolitinib or siJak1 treatment does not result in lower GFP levels in immunofluorescence or blot.

NRVMs were transduced with AdV5-CMV-GFP (**A–C**) and AdV5-CMV-CRYAB^{R120G}-GFP (**C**) and treated with either 3 µM ruxolitinib or DMSO directly after transduction (**A**, **B**) or transfected with siRNA targeting *Jak1* or *Jak2* (siJak1, siJak2), or scramble siRNA (scr) directly before transduction (**C**). Medium change with ruxolitinib or DMSO was performed every other day and cells were fixed or harvested 5 days after transduction. (**A**) mRNA levels of GFP after siRNA treatment. (**B**) IF images. Scale bar = 1000 µm. (**C–E**) Western blots of protein extracts from treated NRVM were stained with antibodies directed against indicated proteins. Quantification was performed with Image Lab (**B**) or Image Studio (**C**) software. Data are depicted as mean ± SEM, and *p*-value was obtained with the unpaired Student's t-test. ns, non-significant.

 © The Author(s)

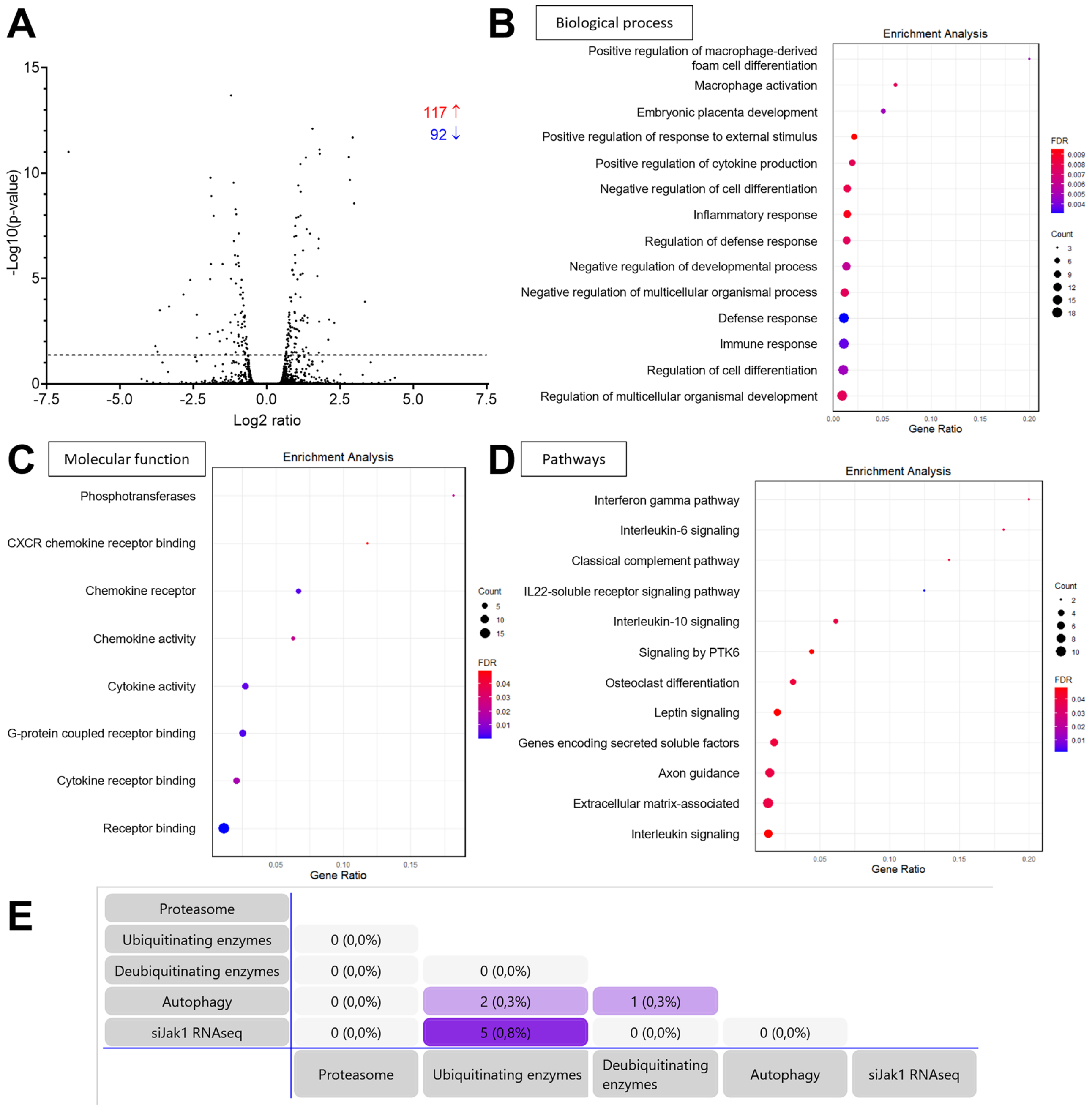

Figure EV4. Effects of *Jak1* knockdown on RNA levels in NRVMs.

NRVMs were transfected with 100 nM siRNA targeting *Jak1* or scramble siRNA and extracted RNA was subjected to paired-end RNA sequencing. Data were obtained from 3 samples of 3 independent NRVM preparations ($n = 3$; each replicate was pooled from 3 samples of 1 NRVM preparation). (**A**) Volcano plots show the −log10 of *P*-value vs. the magnitude of change (log2 ratio) of mRNA levels in siJak1/scr. Differential gene expression analysis was used. Dot plots of enrichment analysis of (**B**) biological processes, (**C**) molecular function, and (**D**) pathways. (**E**) Mapping of significantly up- or down-regulated RNAs to gene identifiers related to proteasome, ubiquitinating enzymes, deubiquitinating enzymes and autophagy.

© The Author(s)

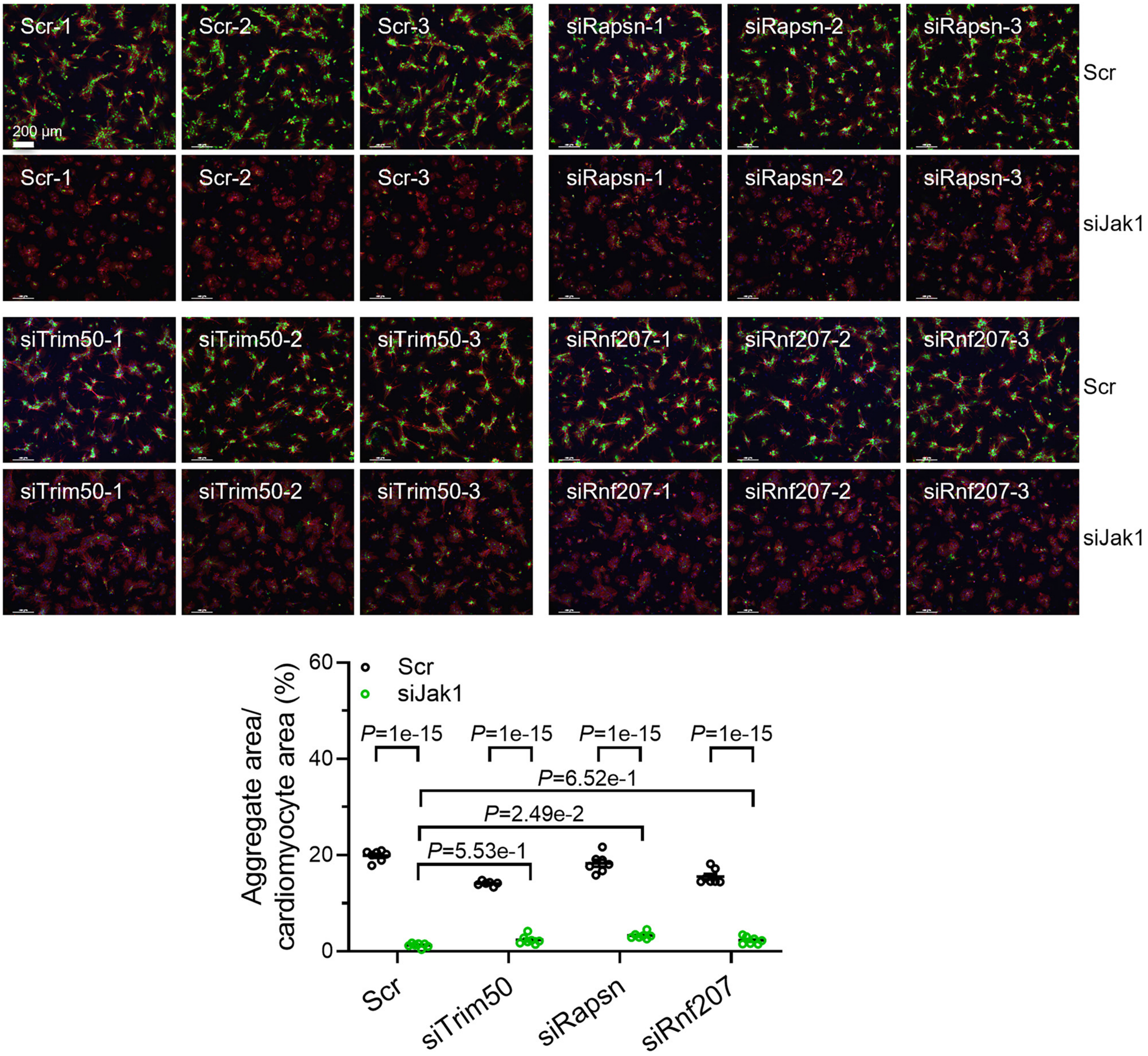

Figure EV5. Knockdown of E3 ligase *Trim50*, *Rapsn* or *Rnf207* has no major effect on CRYAB^{R120G} aggregates after *Jak1* knockdown.

NRVMs transfected with 100 nM siTrim50, siRapsn, siRnf207 or scramble siRNA (scr), transduced with AdV5-CMV-CRYAB^{R120G}, treated with 3 µM ruxolitinib (ruxo) or DMSO and fixed after 5 days. Representative immunofluorescence images. Scale bar = 200 µm. Aggregates are depicted in green (CRYAB^{R120G}-GFP), cells in red (anti-cardiac troponin I) and nuclei in blue (DAPI). Quantification of aggregates in cardiomyocytes with NIS Elements software. Data were obtained from 1 NRVM preparation with 2 wells per condition and 3 images per well, and are depicted as mean ± SEM, and *p*-values were obtained with the two-way ANOVA with Tukey's multiple comparisons post-hoc analysis. Dots represent images.

 © The Author(s)
